# Copper-Catalyzed Azide–Alkyne Cycloaddition of Hydrazoic Acid Formed *In Situ* from Sodium Azide Affords 4-Monosubstituted-1,2,3-Triazoles

**DOI:** 10.1021/acs.joc.1c02775

**Published:** 2022-02-11

**Authors:** Dominik Jankovič, Miha Virant, Martin Gazvoda

**Affiliations:** Faculty of Chemistry and Chemical Technology, University of Ljubljana, Večna pot 113, SI-1000 Ljubljana, Slovenia

## Abstract

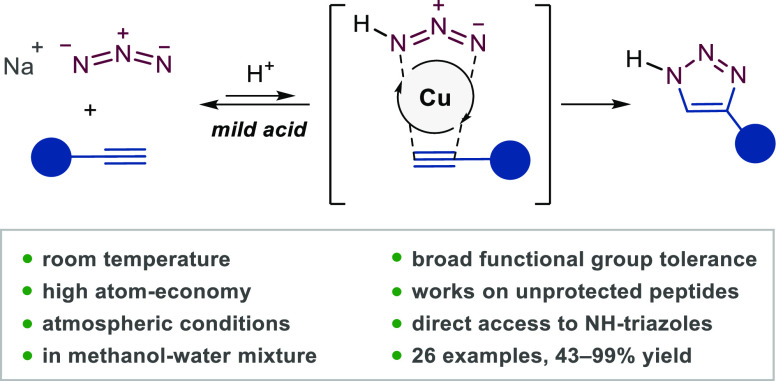

We report a copper-catalyzed cycloaddition of hydrogen azide (hydrazoic acid, HN_3_) with terminal alkynes to form 4-substituted-1*H*-1,2,3-triazoles in a sustainable manner. Hydrazoic acid was formed *in situ* from sodium azide under acidic conditions to react with terminal alkynes in a copper-catalyzed reaction. Using polydentate N-donor chelating ligands and mild organic acids, the reactions were realized to proceed at room temperature under aerobic conditions in a methanol–water mixture and with 5 mol % catalyst loadings to afford 4-substituted-1,2,3-triazoles in high yields. This method is amenable on a wide range of alkyne substrates, including unprotected peptides, showing diverse functional group tolerance. It is applicable for late-stage functionalization synthetic strategies, as demonstrated in the synthesis of the triazole analogue of losartan. The preparation of orthogonally protected azahistidine from Fmoc-l-propargylglycine was realized on a gram scale. The hazardous nature of hydrazoic acid has been diminished as it forms *in situ* in <6% concentrations at which it is safe to handle. Reactions of distilled solutions of hydrazoic acid indicated its role as a reactive species in the copper-catalyzed reaction.

## Introduction

**Figure 1:**
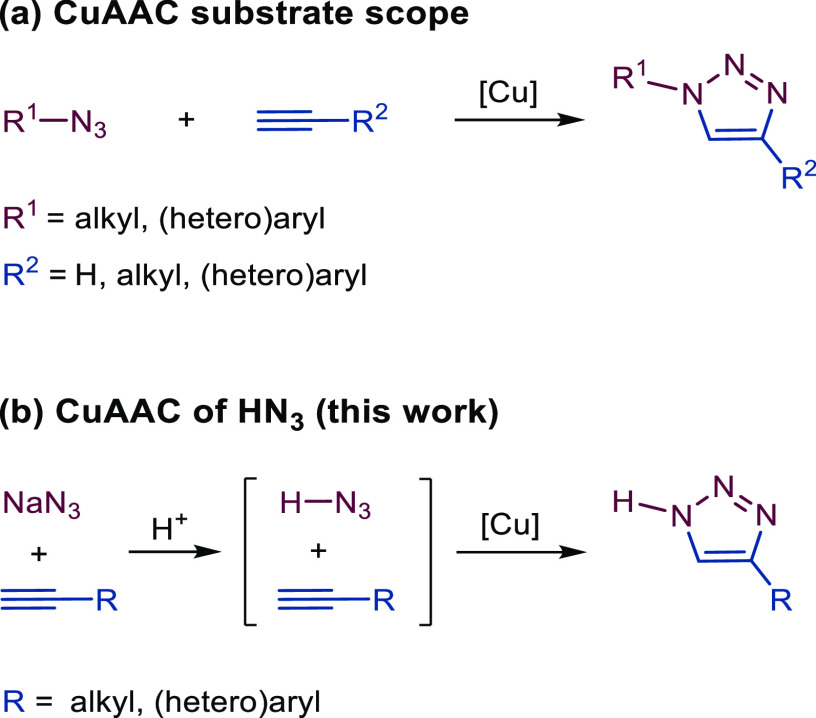
(a) Substrate scope of CuAAC. R^1^, R^2^, and R are defined by the substituent at the end group that most affects the reactivity of the respective reagent. Otherwise, the substrate scope of CuAAC has been extended to complex structures such as biomolecules and polymers with various functional groups in their structure. (b) Extending the substrate scope of CuAAC to the simplest azide, hydrogen azide (hydrazoic acid, HN_3_).

Copper-catalyzed azide–alkyne cycloaddition (CuAAC), the prototypical example of “click” chemistry, has become one of the most widely used reactions and has been extended to various fields of science.^1^ As for the substrate scope, the reaction was extended to all types of starting alkyne reagents and almost all types of organic azide reagents (Figure 1a), and the resulting 1,2,3-triazoles are found in a variety of applications.^2^ However, the CuAAC reaction of hydrogen azide (HN_3_, hydrazoic acid), leading to 4-substituted-1*H*-1,2,3-triazoles, has not yet been realized. Herein, the development of the CuAAC of hydrogen azide is described (Figure 1b).

4-Substituted-1*H*-1,2,3-triazoles (also NH-1,2,3-triazoles or NH-triazoles) are a subclass of 1,2,3-triazoles that have shown interesting biological applications.^3^ Among others, the 4-substituted-1*H*-1,2,3-triazoles moiety is a part of a β-lactam cephalosporin antibiotic cefatrizine (BL-S640),^3e^ noncanonical amino acid azahistidine,^3f,3g^ which can be incorporated into proteins *in vivo*.^3h^ 4-Substituted-1*H*-1,2,3-triazoles have been shown to be useful in coordination chemistry,^4^ e.g., the bis(1,2,3-triazol-4-yl)pyridine motif was demonstrated as a versatile tridentate ligand for supramolecular and coordination chemistry^4a^ and as a ligand for ruthenium in dye-sensitized solar cells.^4b^ NH-triazole is also a convenient scaffold for further modifications, e.g., *N*^2^-selective modifications into 2,4-disubstituted triazoles,^5^ halogenation at the C-5 position of the triazole,^6^*N*^1^-oxidative C–N bond coupling with quinoxalinone^7^ and pyrroles,^8^ gold-catalyzed *N*^1^-selective alkenylation,^9^ and for the preparation of 1,2,3-triazolide ionic liquids.^10^

Current methods for the preparation of 4-substituted-1*H*-1,2,3-triazoles include acid-mediated cycloaddition of nitroolefins and sodium azide,^11^ Pd or Cu-catalyzed reaction of bromoolefins, *anti*-2,3-dibromopropanoic acids, or acetylenes with sodium azide,^12^ reactions of *in situ* formed propargyl azides via the Banert reaction,^13^ multicomponent reactions of aldehydes, nitroalkanes, and sodium azide,^14^ or enolizable ketones, ammonium acetate, and 1-azido-4-nitrobenzene,^15^ N-deallylation of allyltriazoles,^16^ reactions of β-azolylenamines with sulfonyl azides,^17a^ and *N*,*N*-dimethyl enaminones and tosyl azide,^17b^ as well as iodine-promoted cyclization of aryl-methyl ketones, *p*-toluenesulfonyl hydrazide, and 1-aminopyridinium iodide.^18^

4-Substituted-1*H*-1,2,3-triazoles are also putative reaction products of cycloaddition between hydrogen azide (hydrazoic acid, HN_3_) and terminal alkynes. The thermal reaction of hydrazoic acid and phenylacetylene proceeds sluggishly at elevated temperatures.^19^ The CuAAC variant of this reaction is not readily performed, and methods based on transiently protected azides have been developed. To enable this reaction under the CuAAC reaction protocol and to avoid handling hazardous hydrazoic acid, *N*-protected organic azides, such as azidomethyl pivalate, azidomethyl morpholine-4-carboxylate, azidomethyl *N*,*N*-diethylcarbamate,^20^ trimethylsilyl azide,^21^ and α-azidoacetophenone,^22^ were demonstrated as viable substrates for the stepwise synthesis of 4-substituted-1*H*-1,2,3-triazoles via CuAAC, followed by a deprotection sequence. In the case of the trimethylsilyl azide reaction, TMS-*N*^1^-protected triazoles were not isolated, suggesting that hydrazoic acid may be involved in the reaction as a reactive species.^21^ Similarly, the reported reactions of sodium azide and terminal alkynes to NH-triazoles in the presence of a stoichiometric amount of copper, carried out under reflux of methanol for 2 days,^12e^ and on water reaction employing Cu@g-C_3_N_4_,^12f^ could also proceed via *in situ* formed hydrazoic acid. The three-component CuAAC reaction of alkyne, sodium azide, and formaldehyde gave 2-hydroxymethyl-2*H*-1,2,3-triazoles in which the hydroxymethyl group can be removed, providing access to NH-1,2,3-triazoles.^23^ An acid-labile azido linker was developed for the solid-phase synthesis of NH-1,2,3-triazoles, which reacted efficiently in CuAAC and afforded NH-1,2,3-triazoles after TFA cleavage of the resin.^24^

## Results and Discussion

We envisioned to develop a synthetic protocol for the CuAAC of hydrazoic acid to afford 4-substituted-1*H*-1,2,3-triazoles in an atom-economic reaction that would not require reagent preparation, inert atmosphere, anhydrous conditions, high temperatures, multiple synthetic steps, or difficult work-up (Figure 1b).

It was reported that the reactivity of the azide reagent in CuAAC depends on both the electronic properties of the substituent and steric congestion around the reactive group.^25^ In coordination chemistry, organic azides usually behave as neutral n-donors via its *N*^1^ nitrogen atom,^26^ which is also proposed for the azide addition to the activated acetylene in the CuAAC mechanism.^27^ Based on the previous reports of the thermal cyclization reaction of hydrazoic acid and considering its electronic and acidic nature, we expected challenging optimization of the copper-catalyzed reaction. Hydrazoic acid (HN_3_) is a useful synthetic tool,^28^ although commonly avoided because of its hazardous nature. It is toxic if inhaled^29^ and explosive if concentrated.^30^ Pure hydrazoic acid is extremely dangerous and should be omitted, whereas diluted solutions (<10%, w/w) can be safely stored and handled.^30b,31^ Noteworthily, reports of catalytic transformations employing hydrazoic acid as a substrate are scarce.^32^ Our goal was to avoid the direct handling of hydrazoic acid and, therefore, we wanted to form it *in situ* from accessible sodium azide. Nevertheless, when handling HN_3_ solutions, all work should be performed in a fume hood. With safety in mind, we designed the experiments to be as safe as possible.^33^

We began our study on the model reaction of phenylacetylene (**1a**) using modified CuAAC seminal reaction conditions (Table 1), i.e., CuSO_4_ × 5H_2_O, sodium ascorbate (Na(asc)) as a reducing agent for the (re)formation of reactive Cu(I) species,^1b,1d^ THF/H_2_O/EtOH 2:2:1 (v/v/v) as the solvent system to ensure adequate solubility of both the inorganic and organic components. For comparison and to ensure triazole formation, an elevated temperature (100 °C) was used based on a previously reported noncatalyzed thermal reaction. High loadings of the CuSO_4_ catalyst (20 mol %) and sodium ascorbate (Na(asc), 1 equiv) were employed for the initial screening, which was carried out under aerobic conditions. Hydrazoic acid is a moderately weak acid (p*K*_a_ 4.69 at 25 °C),^31^ and to ensure its sufficient formation from sodium azide, strong acids, i.e., sulfuric acid (p*K*_a_ −2.8, 1.99) and *p*-toluenesulfonic acid (p*K*_a_ −2.8 in water^34^), were selected for initial screening. The reaction of phenylacetylene (**1a**) with sodium azide (**2**) proceeded with lower yield in the absence of the acid than in its presence (Table 1, entries 1–3). Replacement of CuSO_4_ × 5H_2_O with CuCl resulted in a slightly reduced yield, while the absence of a reducing additive sodium ascorbate to regenerate Cu(I) from Cu(II) significantly decreased the yield of the reaction (Table 1, entries 4, 5). It is known that Cu(I) species are oxidized to Cu(II) under an ambient atmosphere in the presence of oxygen and that sodium ascorbate converts oxidized copper(II) species back to the catalytically active +1 oxidation state.^1b,1d^ Our goal was to develop the reaction under an ambient atmosphere, and the addition of Na(asc) indeed proved to be crucial. The reaction without the copper cocatalyst was sluggish (Table 1, entry 6), comparable to the reported thermal reaction of hydrazoic acid with phenylacetylene (**1a**). The reported thermal reaction of **1a** with distilled hydrazoic acid in benzene gave the triazole product with 48% yield after 40 h at 110–115 °C.^19b^ Lowering the Cu catalyst loading to 5 mol %, the reaction temperature to 60 °C or the reaction time to 6 h resulted in a significant decrease in yield (Table 1, entries 7–9). The DMF/MeOH 5:1 (v/v) solvent system with CuI/Na(asc) worked similarly (Table 1, entry 10), and increasing the excess of sodium azide (**2**) from 1.5 to 5 equiv did not prove beneficial (Table 1, entry 11). For more details on the initial optimization of the reaction conditions, see Tables S1–S6.

**Table 1:**
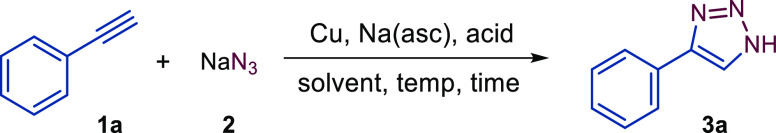
Initial Optimization of Reaction Conditions^a^

no.	acid	catalyst(s)	solvent [ratio v/v/v]	*T* [°C]	*t* [h]	3a [%]^b^
		CuSO_4_/Na(asc)	THF/H_2_O/EtOH [2:2:1]	100	24	25
2	*p*-TsOH	CuSO_4_/Na(asc)	THF/H_2_O/EtOH [2:2:1]	100	24	38
3	H_2_SO_4_	CuSO_4_/Na(asc)	THF/H_2_O/EtOH [2:2:1]	100	24	76
4	H_2_SO_4_	CuCl	THF/H_2_O/EtOH [2:2:1]	100	24	39
5	H_2_SO_4_	CuCl/Na(asc)	THF/H_2_O/EtOH [2:2:1]	100	24	64
6	H_2_SO_4_		THF/H_2_O/EtOH [2:2:1]	100	24	14
7^c^	H_2_SO_4_	CuSO_4_/Na(asc)	THF/H_2_O/EtOH [2:2:1]	100	24	42
8	H_2_SO_4_	CuSO_4_/Na(asc)	THF/H_2_O/EtOH [2:2:1]	60	24	35
9	H_2_SO_4_	CuSO_4_/Na(asc)	THF/H_2_O/EtOH [2:2:1]	100	6	53
10	H_2_SO_4_	CuI/Na(asc)	DMF/MeOH [5:1]	100	24	84
11^d^	H_2_SO_4_	CuSO_4_/Na(asc)	THF/H_2_O/EtOH [2:2:1]	100	24	79^e^

a Reaction conditions: phenylacetylene (**1a**, 1 mmol), NaN_3_ (**2**, 1.5 mmol), acid (1.6 mmol), Cu (20 mol %), sodium ascorbate (1 mmol), and THF/H_2_O/EtOH 2:2:1 (v/v/v, 2.5 mL).

b Yield of purified product **3a** after column chromatography.

c 5 mol % Cu catalyst.

d Reaction with 5 equiv of NaN_3_.

e Conversion into product **3a** was determined by ^1^H NMR spectroscopy using 1,3,5-trimethoxybenzene (TMB) as an internal standard.

**Figure 2:**
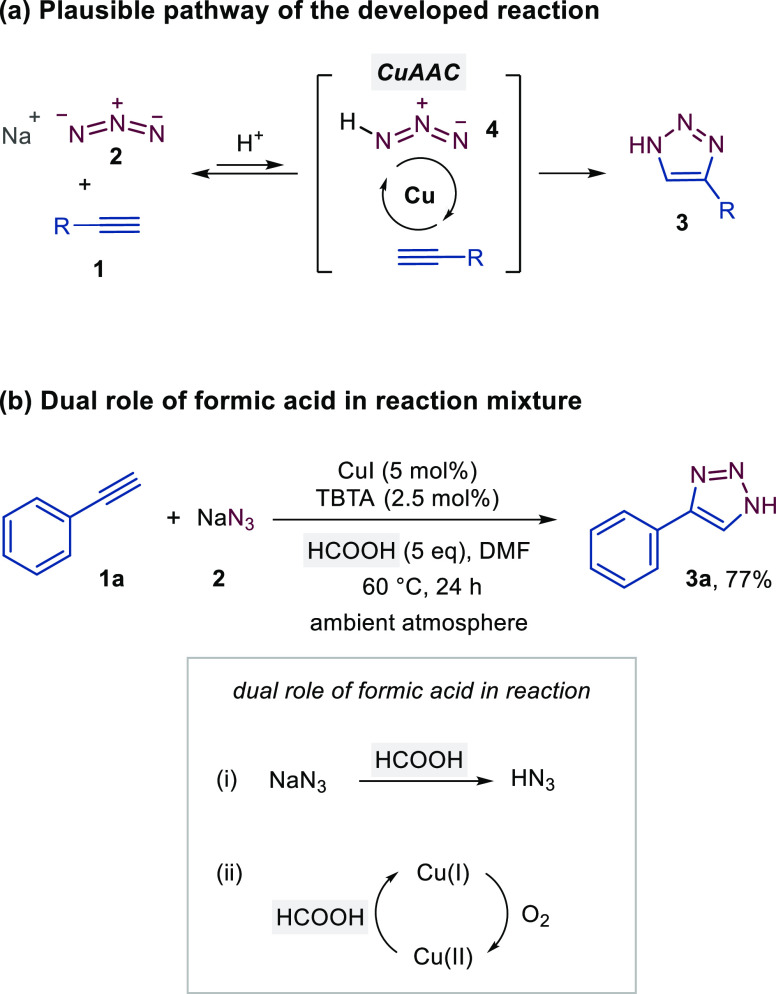
(a) Proposed pathway of the developed Cu(I)-catalyzed reaction. Acid-mediated *in situ* formation of hydrazoic acid **4** from sodium azide **2** is followed by CuAAC of **1** and **4** to give 4-substituted triazoles **3**. (b) Example of a reaction with the CuI/TBTA system with a dual role of formic acid, i.e., for the formation of hydrazoic acid from sodium azide and for the regeneration of Cu(I) from Cu(II).

These results encouraged us to further optimize the reaction conditions, especially with respect to the loading of the copper catalyst, the reaction temperature, and the strength of the employed acid. It is known that N-donor chelating ligands increase the efficiency of the copper catalyst for the CuAAC reaction.^35^ In addition, the use of milder organic acids should also have an overall beneficial effect on the protocol. Based on the result of the initial screening (Table 1, entry 10), we first investigated the effect of the ligand tris(benzyltriazolylmethyl)amine (TBTA) (Table 2, entry 1). The reaction of **1a** with sodium ascorbate (1 equiv) in the presence of 20 mol % of CuI and 10 mol % of TBTA proceeded with 96% conversion to **3a** at 60 °C. Lowering the catalyst and TBTA loadings to 5 and 2.5 mol %, respectively, did not drastically affect the outcome (Table 2, entry 2). However, lowering the temperature to room temperature (22 °C) with 20 mol % loading of the CuI precatalyst and 10 mol % of TBTA resulted in a decrease of conversion to 51% (Table 2, entry 3). Concentrating the reaction mixture from 0.40 to 0.83 M increased the conversion from 51 to 85% (Table 2, entry 4). However, decreasing the loading of CuI to 5 mol % and TBTA to 2.5 mol % caused the conversion to drop to 35% (Table 2, entry 5). Assuming that methanoic acid can act as a reducing agent,^36^ we hypothesized that it might play a dual role in our case: as an acid to provide hydrazoic acid from sodium azide and as a mild reducing agent for the regeneration of Cu(I). Indeed, the reaction with formic acid in the absence of sodium ascorbate proceeded to **3a** at 60 °C with 77% conversion (Table 2, entry 6; Figure 2b). Introducing a substoichiometric amount (20 mol %) of sodium ascorbate and running the reaction at 40 °C further improved the protocol and gave 90% of the product **3a** (Table 2, entry 7). We investigated another CuAAC accelerating ligand, tris(2-benzimidazolylmethyl)amine (BimH)_3_. Using various mild organic acids, i.e., methanoic acid, lactic acid, trifluoroacetic acid, and acetic acid, in combination with (BimH)_3_, we were able to obtain quantitative conversions of phenylacetylene (**1a**) to the triazole product **3a** at room temperature with 5 mol % of CuSO_4_ and 5 mol % of (BimH)_3_ (Table 2, entries 12–15). We have also investigated other ligands such as triphenylphosphine (PPh_3_), 1,4-diazabicyclo[2.2.2]octane (DABCO), Bipy (2,2′-bipyridine), and phenanthroline (Phen), which were found to be less efficient than TBTA and (BimH)_3_ (Table 2, entries 8–11). To ensure sufficient solubility of all components, DMF was used as a solvent for the initial experiments to optimize the reaction conditions. However, with the optimized protocol, we demonstrated that the reactions could be carried out with the same efficiency in the MeOH/H_2_O solvent system (Table 2, entries 12–15), as well as in EtOH,^33^ recommended by CHEM21 to be optimal based on safety, health, and environmental criteria.^37^ More details on catalytic system optimizations can be found in Tables S7–S9.^33^

**Table 2:**
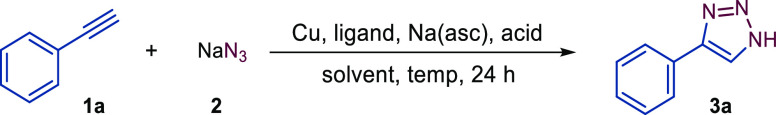
Tuning of the Catalytic System with Appropriate Ligand and Organic Acid Selection^a^

no.	acid (equiv)	Cu (mol %)	Na(asc) (equiv)	ligand (mol %)	solvent (conc., M)	*T* (°C)	3a (%)^b^
1	H_2_SO_4_ [1.6]	CuI [20]	1	TBTA [10]	DMF [0.40]	60	96
2	H_2_SO_4_ [1.6]	CuI [5]	1	TBTA [2.5]	DMF [0.40]	60	89
3	H_2_SO_4_ [1.6]	CuI [20]	1	TBTA [10]	DMF [0.40]	RT	51
4	H_2_SO_4_ [1.6]	CuI [20]	1	TBTA [10]	DMF [0.83]	RT	85
5	H_2_SO_4_ [1.6]	CuI [5]	1	TBTA [2.5]	DMF [0.83]	RT	35
6	HCOOH [5]	CuI [5]	0	TBTA [2.5]	DMF [0.83]	60	77
7	HCOOH [5]	CuI [5]	0.2	TBTA [2.5]	DMF [0.83]	40	90 (89)^c^
8	acetic acid [3]	CuI [5]	0.2	PPh_3_ [5]	DMF [0.83]	40	43
9	acetic acid [2]	CuI [5]	0.2	DABCO [5]	DMF [0.83]	40	63
10	acetic acid [3]	CuI [5]	0.2	Bipy [10]	DMF [0.83]	40	67
11	acetic acid [3]	CuI [5]	0.2	Phen [5]	DMF [0.83]	40	73 (72)^c^
12	HCOOH [3]	CuSO_4_ [5]	0.25	(BimH)_3_ [5]	MeOH/H_2_O [0.83]^d^	RT	99
13	lactic acid [2]	CuSO_4_ [5]	0.25	(BimH)_3_ [5]	MeOH/H_2_O [0.83]^d^	RT	90
14	TFA [2]	CuSO_4_ [5]	0.25	(BimH)_3_ [5]	MeOH/H_2_O [0.83]^d^	RT	95
15	acetic acid [6.6]	CuSO_4_ [5]	0.25	(BimH)_3_ [5]	MeOH/H_2_O [0.83]^d^	RT	100 (99)^c^

a Reaction conditions: phenylacetylene (**1a**, 0.5 mmol), NaN_3_ (**2**, 0.75 mmol). TBTA: Tris[(1-benzyl-1*H*-1,2,3-triazol-4-yl)methyl]amine, (BimH)_3_: tris(2-benzimidazolylmethyl)amine, and TFA: trifluoroacetic acid. RT (22 °C).

b Conversion was determined by ^1^H NMR spectroscopy using 1,3,5-trimethoxybenzene (TMB) as an internal standard.

c Yield of **3a** after purification by column chromatography.

d MeOH/H_2_O 3:1 (v/v) solvent mixture.

To investigate whether the developed copper-catalyzed reaction indeed proceeds with hydrazoic acid as the reactive species, its aqueous solution was independently prepared^33^ and used in test experiments under similar reaction conditions as described above. The conversions to the triazole product **3a** were comparable for reactions with a distilled hydrazoic acid solution (Table 3) and experiments in which hydrazoic acid was formed *in situ* from sodium azide (Tables 1 and 2). In the catalytic system with CuSO_4_ and Na(asc), the temperature-depended formation of **3a** was observed (Table 3, entries 1–6), and the conversions were aligned with the results in which hydrazoic acid was formed *in situ* (see Table 1, entries 3, 8). The reaction without copper resulted in low conversion, again demonstrating the crucial role of the Cu(I) catalyst (Table 3, entry 7). Using the developed protocol with the (BimH)_3_ ligand, the reaction with distilled hydrazoic acid proceeded at room temperature with near-quantitative conversion (Table 3, entry 9), similar to the example where hydrazoic acid was formed *in situ* (Table 2, entry 15).

**Table 3:**
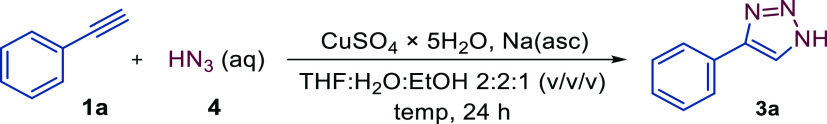
Reactions with Solutions of Distilled Hydrazoic Acid^a^

no.	catalytic system	*T* (°C)	3a (%)^b^
1	CuSO_4_/Na(asc)	RT	1
2	CuSO_4_/Na(asc)	40	34
3	CuSO_4_/Na(asc)	40	34^a^
4	CuSO_4_/Na(asc)	60	38
5	CuSO_4_/Na(asc)	80	50
6	CuSO_4_/Na(asc)	100	78
7		100	14
8^c^	CuSO_4_/Na(asc)	100	72 (70)^d^
9^e^	CuSO_4_/(BimH)_3_, Na(asc)	RT	92

a Reaction conditions: phenylacetylene (**1a**, 1 mmol), HN_3_ (**4**, 0.5 mmol, 1 mL of 0.5 M aqueous solution), CuSO_4_ × 5H_2_O (20 mol %), sodium ascorbate (1 mmol), THF/H_2_O/EtOH 2:2:1 (v/v/v, 2.5 mL), and reaction time 72 h.

b Conversion was determined by ^1^H NMR spectroscopy using 1,3,5-trimethoxybenzene (TMB) as an internal standard.

c Phenylacetylene (**1a**, 0.17 mmol), HN_3_ (**4**, 0.5 mmol, 1 mL of 0.5 M aqueous solution), CuSO_4_ × 5H_2_O (20 mol %), and sodium ascorbate (0.17 mmol).

d Yield after purification by column chromatography.

e Phenylacetylene (**1a**, 0.16 mmol), HN_3_ (**4**, 0.31 mmol, 0.5 mL of 0.62 M aqueous solution), CuSO_4_ × 5H_2_O (5 mol %), (BimH)_3_ (5 mol %), sodium ascorbate (0.04 mmol), and solvent MeOH/H_2_O 3:1 (v/v, 2 mL).

**Figure 3:**
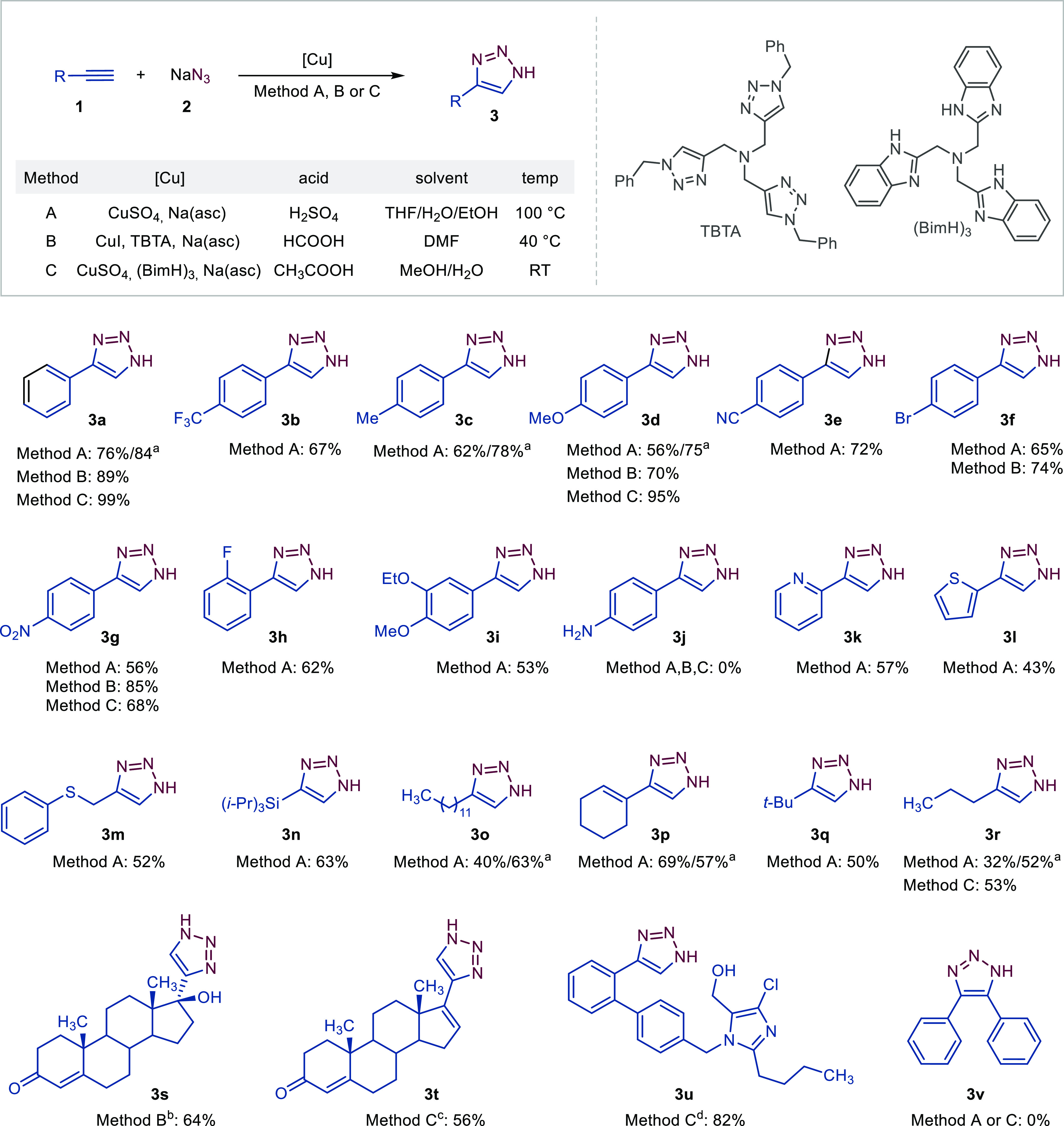
Conditions: Method A: alkyne (1 mmol), NaN_3_ (1.5 mmol), H_2_SO_4_ (1.6 mmol), Cu (20 mol %), Na(asc) (1 mmol), and THF/H_2_O/EtOH 2:2:1 (v/v/v). ^a^CuI as a copper catalyst and DMF/MeOH 5:1 (v/v) as a solvent system. Method B: alkyne (0.5 mmol), NaN_3_ (0.75 mmol), HCOOH (2.5 mmol), CuI (5 mol %), TBTA (2.5 mol %), Na(asc) (0.1 mmol), and DMF. Method C: alkyne (0.5 mmol), NaN_3_ (0.75 mmol), CH_3_COOH (3.3 mmol), Cu (5 mol %), (BimH)_3_ (5 mol %), Na(asc) (0.125 mmol), and MeOH/H_2_O 3:1 (v/v). ^b^Slightly modified Method B with a reaction temperature of 60 °C and a reaction time of 120 h. ^c^MeOH as the only solvent. ^d^Slightly modified Method C with 20 mol % of Cu catalyst and (BimH)_3_ ligand, 2 equiv NaN_3_, 0.5 equiv Na(asc), reaction time of 72 h at 40 °C, see the Supporting Information for more details.

**Figure 4:**
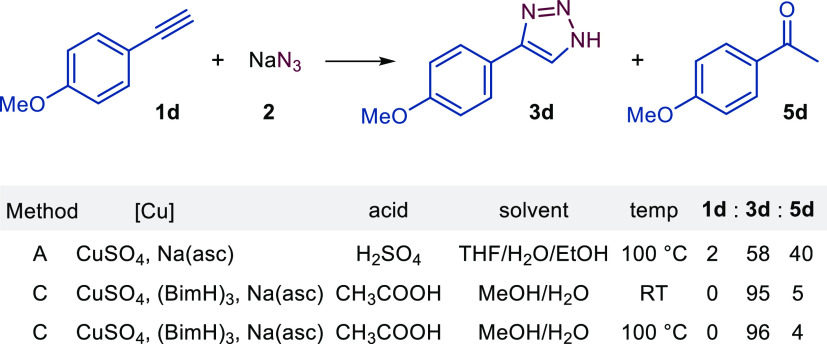
Diminishing competitive ketone formation **5d** over triazole formation **3d** in the case of alkyne **1d** by employing optimized reaction conditions. The ratio of **1d/3d/5d** is given in %.

Three methods with different reaction conditions derived from the optimization experiments, i.e., ligandless Method A (Table 1, entry 3) that employs H_2_SO_4_, Method B with the Cu/TBTA system and formic acid (Table 2, entry 7), and Method C with the Cu/(BimH)_3_ system and acetic acid (Table 2, entry 15), were used for substrate scope screening as shown in Figure 3. Although ligandless Method A employs harsh reaction conditions, it was used for the initial screening on simple, robust substrates, whereas Methods B and C were used for more complex molecular structures with acid- and temperature-sensitive functional groups and when Method A did not provide sufficient conversion. Noteworthily, the starting concentration of hydrazoic acid in solution for reaction employing acetic acid, for example, that from Table 2, entry 15, was estimated to be ∼3%.^33^ When preparing 4-aryl-1*H*-1,2,3-triazoles **3** by Method A, we observed no drastic effect of *para*-substituents at the phenyl ring of acetylenes **1** on the yield of the products **3b**–**3g**. However, the reactions of acetylenes **1d** and **1i** with a strongly electron-donating methoxy group resulted in ketone side products when using the method with H_2_SO_4_. In the case of **1d** (Figure 4) and **1i**, the ketone side product was formed in 40 and 38%, respectively, as evident from the ^1^H NMR spectra of the crude reaction mixtures.^33^ The acid-catalyzed oxidation of terminal alkynes in aqueous media is an expected competitive reaction.^38^ We were able to minimize this oxidation process using a room-temperature protocol and a mild organic acid with the Cu/(BimH)_3_ catalytic system (Method C), which provided **3d** in 95% yield. A similar result was obtained with Method C also at 100 °C (Figure 4). In contrast, 4-aminophenylacetylene (**1j**) did not react to form **3j** under any of the developed conditions and the ketone product 1-(4-aminophenyl)ethan-1-one was obtained in 99% yield by all methods. Reactions of heteroaryl- and alkyl-substituted alkynes proceeded with lower but still acceptable yields (43–63%) to the corresponding triazoles **3k**–**3r**. We attempted the reactions of hydrophobic steroid substrates **1s** and **1t**. The reaction of ethisterone **1s** was troublesome due to the low solubility (≈1 mg/mL); therefore, the temperature had to be increased and the time prolonged to achieve sufficient conversion to the product **3s**, which was eventually isolated in 64% yield. On the other hand, the reaction of **1t** in methanol as the only reaction solvent afforded **3t** with 56% isolated yield. We have attempted the synthesis of the triazole analogue of losartan, a drug used to treat hypertension. From the alkyne substrate **1u**,^33^ we were able to prepare the losartan triazole analogue **3u** in 82% yield with slightly modified reaction conditions. On the other hand, the reaction with diphenylacetylene **1v** did not proceed to triazole **3v**, further indicating the CuAAC reaction pathway of the developed method (Figure 2a).

In all cases, the products **3** were purified by simple silica gel column chromatography since the resulting triazoles have distinctive retention factors than the residual ligand and starting acetylene **1** when the conversions were not complete. In many cases, especially when the more efficient Methods B or C were used, yielding **3** with higher conversions, the crude products **3** were almost in the pure form after ethyl acetate extraction (Method A) or filtration through a silica pad and evaporation (Methods B and C), with crystallization also available as a means of purification, as shown in the case of synthesis **3a** by Method C.^33^

**Figure 5:**
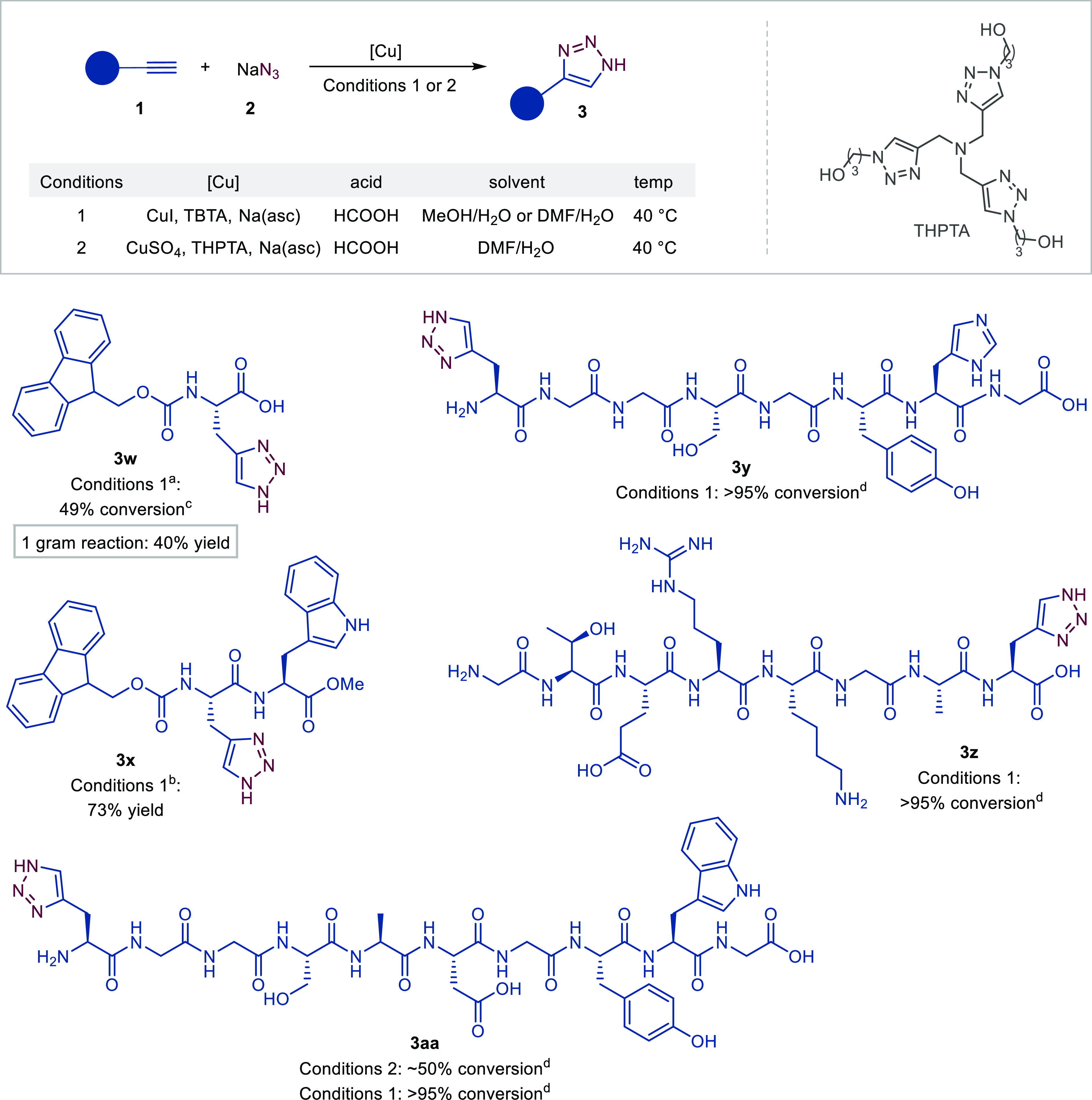
Conditions: Conditions 1 for **3w** and **3x**: alkyne **1w** or **1x** (1 equiv, 500 mM), NaN_3_ (1.5 equiv), HCOOH (5 equiv), Cu (20 mol %), TBTA (10 mol %), Na(asc) (0.2 equiv), 40 °C, 24 h. ^a^MeOH/H_2_O 5:1 (v/v). ^b^DMF as the only solvent. Conditions 1 for **3y**, **3z** and **3aa**: peptide (100 mM), NaN_3_ (200 mM), HCOOH (500 mM), Cu (50 mM), TBTA (50 mM), Na(asc) (100 mM), DMF/H_2_O 7:3 (v/v), 40 °C. Conditions 2: peptide (50 mM), NaN_3_ (100 mM), HCOOH (500 mM), Cu (25 mM), THPTA (125 mM), Na(asc) (200 mM), DMF/H_2_O 3:7 (v/v), 40 °C. ^c^Conversion was determined by ^1^H NMR using 1,3,5-trimethoxybenzene as an internal standard. ^d^Conversion was determined by HPLC analysis, see the Supporting Information for more details.

Encouraged by these results, we evaluated the developed method on peptides with an alkynyl handle (Figure 5). Modified reaction conditions of Method B from Figure 3 by employing the MeOH/H_2_O solvent mixture instead of DMF and higher CuI (20 mol %) and TBTA (10 mol %) loadings were found to be applicable for the reaction of Fmoc-L-propargylglycine **1w**. The reaction of **1w** to Fmoc-protected azahistidine **3w** proceeded with 49% conversion. The described synthesis simplifies the preparation of azahistidine, an interesting noncanonical amino acid that can be incorporated into peptides and proteins and that has previously been accessible only in a stepwise manner.^3f,3h^ We have demonstrated that the protocol can be scaled-up and 1 gram-scale reaction can be performed that resulted in **3w** in 40% yield. Similar reaction conditions were successfully used to prepare dipeptide **3x**, which was isolated in 73% yield.

Modified reaction conditions were developed for more complex peptides. A catalytic system using CuI, TBTA, Na(asc), and HCOOH in a H_2_O/DMF 3:7 (v/v) solvent mixture worked well for propargylglycine-containing 8-mers **1y**, **1z**, and 10-mer **1aa**, with the corresponding triazole peptide derivatives **3y**, **3z**, and **3aa** obtained with >95% conversion in all cases. The reactions were carried out in a 100 mM peptide concentration at 40 °C and monitored by HPLC.^33^ The water-soluble *tris*-hydroxypropyltriazolyl-methylamine (THPTA) ligand in combination with CuSO_4_, Na(asc), and HCOOH in an aqueous solvent mixture, H_2_O/DMF 7:3 (v/v) (Figure 5, Conditions 2), afforded the triazole peptide **3aa** in ∼50% conversion. The reaction was carried out in a 50 mM concentration of peptide **1aa** at 40 °C. The reaction protocols were found to be tolerant to various functional groups and moieties, e.g., hydroxy, phenol, amine, imidazole, carboxylic acid, and guanidino groups, of serine, tyrosine, lysine, histidine, glutamic acid, and arginine amino acid residues, respectively.

Noteworthily, NH-triazole derivatives of peptides or other biomolecules were not reported yet. Biomolecules typically require the use of aqueous conditions that are incompatible with many common methods for the preparation of 4-substituted-1*H*-1,2,3-triazoles. Similarly, these methods typically require the synthesis of starting reagents whose synthesis is incompatible with the biomolecule substrates. Methods involving the stepwise synthesis of NH-triazoles via CuAAC with transiently protected azides, followed by base-mediated deprotection, require isolation of 1,4-substituted-triazole intermediates and/or basic conditions, neither of which is suitable for the synthesis of biomolecules.

## Conclusions

We report a copper-catalyzed reaction for the preparation of 4-substituted-1*H*-1,2,3-triazoles from terminal alkynes and sodium azide. In most aspects, the method follows the principles of green chemistry.^39^ The reactions proceed with *in situ* formed hydrazoic acid that is present in solutions in low (<6%, w/w) concentrations. By employing tetradentate N-donor ligands, such as TBTA and (BimH)_3_, the reactions were realized at room temperature with 5 mol % copper catalyst loading, had high atom economy, and produced triazoles in good yields with little waste and by-products. The reactions could be carried out in environmentally friendly solvents and could afford 4-substituted-1,2,3-triazoles from the readily available alkynes and sodium azide, with almost complete incorporation of the starting materials into the final products. The method was demonstrated on a wide range of substrates, and the resulting NH-triazoles could be easily isolated. Noteworthily, 4-substituted-1*H*-1,2,3-triazoles, which were previously synthesized in a stepwise manner and required the preparation of starting reagents and/or an inert atmosphere and anhydrous solvents, are now accessible in one step from terminal alkynes under an ambient atmosphere. The developed method gives access to NH-triazole derivatives of peptides and potentially of other biomolecules. We have demonstrated the dual role of formic acid in the reported system, i.e., as an acid for the formation of hydrazoic acid from sodium azide and as a mild reducing agent for the regeneration of Cu(I) from Cu(II). It is noteworthy that hydrazoic acid was recently used for the large-scale synthesis of an early aryltetrazole intermediate in the synthesis of a drug candidate, giving the developed method the potential for scaling-up.^40^

## Experimental Section

### Hydrazoic Acid Caution!

Hydrazoic acid (HN_3_) is toxic if inhaled^29^ and can be explosive if concentrated. It was reported that hydrazoic acid at a concertation above 20% (w/w) can be explosive,^30a^ although an ignition source (e.g., spark) is required for detonation of its vapors.^30b^ Hydrazoic acid solutions with concentrations under 10% (w/w) are safe to store and handle.^31^ Therefore, to minimize its potential hazards, reactions were performed on the low scale (0.5–1.5 mmol) and at low concentrations of NaN_3_/hydrazoic acid (max. 6%, w/w) in closed systems inside the fume hood.

### General Procedure for Method A (Figure 3)

Into an 8 or 21 mL ACE pressure tube stirring bar, alkyne (1 mmol, 1 equiv), CuSO_4_ × 5H_2_O (50 mg, 0.2 mmol, 20 mol %), sodium ascorbate (198 mg, 1 mmol), NaN_3_ (98 mg, 1.5 mmol), a THF/H_2_O/EtOH 2:2:1 (v/v/v) solvent mixture, and 96% H_2_SO_4_ (89 μL, 164 mg, 1.6 mmol) were added in that order. After the addition of sulfuric acid, the ACE tube was sealed with a screw cap and the reaction mixture was stirred for 24 h at 100 °C in a preheated aluminum heating block. After that time, the reaction mixture was allowed to cool to room temperature in a closed ACE pressure tube. Prior to work-up, the ACE tube was uncapped and left open for a few minutes in the fume hood to allow any excess hydrazoic acid in the vapor phase to evaporate. To the reaction mixture were added ethyl acetate or dichloromethane (30 mL) and ammonium chloride solution (20 mL sat. NH_4_Cl + 20 mL H_2_O), and phases were separated in a separatory funnel. The aqueous phase was additionally extracted with ethyl acetate (2 × 30 mL). Organic phases were combined and dried over anhydrous Na_2_SO_4_, filtered, and the solvent was removed with the aid of a rotary evaporator. The crude product was purified by silica column chromatography.

### General Procedure for Method A with the CuI Catalyst and DMF/MeOH Solvent System (Figure 3)

Into an 8 or 21 mL ACE pressure tube stirring bar, alkyne (1 mmol or 0.5 mmol scale, 1 equiv), CuI (20 mol %), sodium ascorbate (1 equiv), NaN_3_ (1.5 equiv), a DMF/MeOH 5:1 (v/v) solvent mixture, and 96% H_2_SO_4_ (1.6 equiv) were added in that order. After the addition of sulfuric acid, the ACE tube was sealed with a screw cap and the reaction mixture was stirred for 24 h at 100 °C in a preheated aluminum heating block. After that time, the reaction mixture was allowed to cool to room temperature in a closed ACE pressure tube. Prior to work-up, the ACE tube was uncapped and left open for a few minutes in the fume hood to allow any excess hydrazoic acid in the vapor phase to evaporate. The reaction mixture was filtered through a short pad of silica gel using ethyl acetate as an eluent. The resulting filtrate was concentrated with the aid of a rotary evaporator, and the crude product was purified by silica gel column chromatography.

### General Procedure for Method B (Figure 3)

Into an 8 mL ACE pressure tube stirring bar, alkyne (0.5 mmol, 1 equiv), CuI (4.8 mg, 0.025 mmol, 5 mol %), sodium ascorbate (20 mg, 0.1 mmol, 0.2 equiv), tris[(1-benzyl-1*H*-1,2,3-triazol-4-yl)methyl]amine (TBTA) (6.6 mg, 0.0125 mmol, 2.5 mol %), NaN_3_ (49 mg, 0.75 mmol, 1.5 equiv), DMF (0.6 mL), and HCOOH (94 μL, 115 mg, 2.5 mmol, 5 equiv) were added in that order. After the addition of methanoic acid, the ACE tube was sealed with a screw cap and the reaction mixture was stirred for 24 h at 40 °C in a preheated aluminum heating block. Then, the reaction tube was uncapped and the reaction mixture was filtered through a short pad of silica gel using ethyl acetate as an eluent. The resulting filtrate was concentrated with the aid of a rotary evaporator, and the crude product was purified by silica gel column chromatography.

### General Procedure for Method C (Figure 3)

Into a 5 mL round bottom flask with a stirring bar, alkyne (0.5 mmol, 1 equiv), CuSO_4_ × 5H_2_O (6.2 mg, 0.025 mmol, 5 mol %), sodium ascorbate (25 mg, 0.125 mmol, 0.25 equiv), tris(2-benzimidazolylmethyl)amine ((BimH)_3_) (10 mg, 0.025 mmol, 5 mol %), NaN_3_ (49 mg, 0.75 mmol, 1.5 equiv), a MeOH/H_2_O 3:1 (v/v, 0.6 mL) solvent, and CH_3_COOH (189 μL, 198 mg, 3.3 mmol, 6.6 equiv) were added in that order. After the addition of acetic acid, the flask was capped with a glass stopper and the reaction mixture was stirred for 24 h at room temperature. Then, the reaction flask was uncapped and the reaction mixture was filtered through a short pad of silica gel using ethyl acetate as an eluent. The resulting filtrate was concentrated with the aid of a rotary evaporator, and the crude product was purified by silica gel column chromatography.

### Reactions of Peptides (Figure 5)

#### Conditions 1

The following stock solutions were prepared: peptide stock solution (250 mM in DMF/H_2_O 1:1, v/v) and reagent stock solution (TBTA (100 mM), CuI (100 mM), Na(asc) (200 mM), HCOOH (1.0 M), internal standard (100 mM) in DMF), and NaN_3_ (2.0 M in H_2_O) stock solution. The reaction was performed in a 200 μL Eppendorf tube. Stock solutions were added in the following order: 20 μL of peptide stock solution, 25 μL of reagent stock solution, and 5 μL of NaN_3_ stock solution. After the addition of each stock solution, the mixture was mixed by pipetting up and down 15 times. The reaction was set at 40 °C in a preheated oil bath. Final concentrations of reagents were: peptide (100 mM), NaN_3_ (200 mM), HCOOH (500 mM), Cu (50 mM), TBTA (50 mM), Na(asc) (100 mM), and solvent: DMF/H_2_O 7:3 (v/v). For analysis, 5 μL aliquot of the reaction mixture was taken, diluted with 250 μL of DMF + 250 μL H_2_O, and analyzed by HPLC and HPLC-HRMS.

#### Conditions 2

The following stock solutions were prepared: peptide stock solution (125 mM in DMF/H_2_O 1:1, v/v), reagent stock solution (THPTA (250 mM), CuSO_4_ (50 mM), Na(asc) (400 mM), HCOOH (1.0 M) in DMF/H_2_O 1:4, v/v), and NaN_3_ (1.0 M in H_2_O) stock solution. The reaction was performed in a 200 μL Eppendorf tube. Stock solutions were added in the following order: 40 μL of peptide stock solution, 50 μL of reagent stock solution, and 10 μL of sodium azide stock solution. After the addition of each stock solution, the reaction mixture was mixed by pipetting up and down 15 times. The final concentrations of reagents were: peptide (50 mM), NaN_3_ (100 mM), HCOOH (500 mM), Cu (25 mM), THPTA (125 mM), Na(asc) (200 mM), and DMF/H_2_O 3:7 (v/v). The reaction was set at 40 °C in a preheated oil bath. For analysis, 10 μL aliquot of the reaction mixture was taken, diluted with 500 μL H_2_O, and analyzed by HPLC and HPLC-HRMS.

#### 4-Phenyl-1*H*-1,2,3-triazole (**3a**)

Mp: 144–146 °C. Mp (lit.): 145–146 °C.^23^ IR (cm^–1^): 3152, 3116, 2956, 2850, 1454, 1082, 971, 873, 763, 691. ^1^H NMR (500 MHz, CDCl_3_) δ 7.99 (s, 1H), 7.85–7.81 (m, 2H), 7.49–7.44 (m, 2H), 7.41–7.37 (m, 1H).

#### 4-(4-(Trifluoromethyl)phenyl)-1*H*-1,2,3-triazole (**3b**)

Mp: 188.5–189.5 °C. IR (cm^–1^): 3141, 2967, 2866, 1424, 1320, 1169, 1130, 1063, 973, 840. ^1^H NMR (500 MHz, CDCl_3_) δ 11.83 (br, 1H), 8.04 (s, 1H), 7.98–7.93 (m, 2H), 7.74–7.70 (m, 2H). NMR data are in agreement with the literature.^12d^

#### 4-(4-Methylphenyl)-1*H*-1,2,3-triazole (**3c**)

Mp: 152–154.5 °C. Mp (lit.): 150–152 °C.^23^ IR (cm^–1^): 3155, 3123, 2858, 1477, 1075, 999, 972, 874, 820, 723. ^1^H NMR (500 MHz, CDCl_3_) δ 11.67 (br, 1H), 7.96 (s, 1H), 7.74–7.70 (m, 2H), 7.27 (d, *J* = 7.4 Hz, 2H), 2.40 (s, 3H).

#### 4-(4-Methoxyphenyl)-1*H*-1,2,3-triazole (**3d**)

Mp: 167–168.5 °C. Mp (lit.): 168–169 °C.^12e^ IR (cm^–1^): 3153, 3114, 2835, 1613, 1533, 1465, 1247, 972, 873, 827. ^1^H NMR (500 MHz, CDCl_3_) δ 7.90 (s, 1H), 7.77–7.73 (m, 2H), 7.01–6.96 (m, 2H), 3.86 (s, 3H).

#### 4-(4-Cianophenyl)-1*H*-1,2,3-triazole (**3e**)

Mp: 172.8–174.5 °C. Mp (lit.): 170–172 °C.^23^ IR (cm^–1^): 3125, 2857, 2227, 1612, 1131, 1079, 997, 969, 869, 850. ^1^H NMR (500 MHz, CDCl_3_) δ 11.96 (br, 1H), 8.04 (s, 1H), 7.97–7.94 (m, 2H), 7.77–7.73 (m, 2H).

#### 4-(4-Bromophenyl)-1*H*-1,2,3-triazole (**3f**)

Mp: 170.2–173 °C. Mp (lit.): 172–173 °C.^41^ IR (cm^–1^): 3149, 3120, 2837, 1131, 1068, 1001, 969, 874, 830, 722. ^1^H NMR (500 MHz, CDCl_3_) δ 7.96 (s, 1H), 7.72–7.68 (m, 2H), 7.61–7.57 (m, 2H).

#### 4-(4-Nitrophenyl)-1*H*-1,2,3-triazole (**3g**)

Mp: 199.5–201 °C. Mp (lit.): 198–199 °C.^42^ IR (cm^–1^): 3104, 2896, 1603, 1509, 1333, 1107, 854, 819, 755, 711. ^1^H NMR (500 MHz, DMSO-*d*_6_) δ 8.63 (br, 1H), 8.34–8.30 (m, 2H), 8.18–8.12 (m, 2H).

#### 4-(2-Fluorophenyl)-1*H*-1,2,3-triazole (**3h**)

Mp: 83.5–85.5 °C. IR (cm^–1^): 3126, 2855, 1471, 1217, 1077, 1001, 975, 860, 817, 753. ^1^H NMR (500 MHz, CDCl_3_) δ 8.14 (d, *J* = 3.5 Hz, 1H), 8.10–8.06 (m, 1H), 7.39–7.34 (m, 1H), 7.29–7.24 (m, 1H), 7.22–7.16 (m, 1H). NMR data are in agreement with the literature.^12d^

#### 4-(3-Ethoxy-4-Methoxyphenyl)-1*H*-1,2,3-triazole (**3i**)

Mp: 133–134.5 °C. IR (cm^–1^): 3131, 2836, 1493, 1348, 1251, 1234, 1142, 993, 852, 826. ^1^H NMR (500 MHz, CDCl_3_) δ 7.91 (s, 1H), 7.40 (d, *J* = 2.1 Hz, 1H), 7.33 (dd, *J* = 8.3, 2.1 Hz, 1H), 6.94 (d, *J* = 8.3 Hz, 1H), 4.19 (q, *J* = 7.0 Hz, 2H), 3.92 (s, 3H), 1.49 (t, *J* = 7.0 Hz, 3H). ^13^C{^1^H} NMR (126 MHz, CDCl_3_) δ 150, 148.9, 147.3, 129.4, 122.8, 118.9, 111.8, 110.6, 64.7, 56.2, 15. HRMS–ESI (*m/z*): [M + H]^+^ calcd for C_11_H_14_N_3_O_2_^+^, 220.1081; found, 220.1081.

#### 2-(1*H*-1,2,3-triazol-4-yl)pyridine (**3k**)

Mp: 153.5–155.4 °C. IR (cm^–1^): 3118, 2724, 1703, 1601, 1510, 1005, 960, 890, 781, 740. ^1^H NMR (500 MHz, CDCl_3_) δ 8.71 (d, *J* =4.8 Hz, 1H), 8.33 (s, 1H), 8.02–7.97 (m, 1H), 7.85–7.79 (m, 1H), 7.33–7.28 (m, 1H). NMR data are in agreement with the literature.^4c^

#### 4-(Tiophen-2-yl)-1*H*-1,2,3-triazole (**3l**)

Mp: 90.5–92.5 °C. IR (cm^–1^): 3117, 2973, 2843, 1416, 1123, 1033, 997, 934, 847, 692. ^1^H NMR (500 MHz, CDCl_3_) δ 7.92 (s, 1H), 7.43 (dd, *J* = 3.6, 1.1 Hz, 1H), 7.34 (dd, *J* = 5.2, 1.1 Hz, 1H), 7.10 (dd, *J* = 5.2, 3.6 Hz, 1H). ^13^C{^1^H} NMR (126 MHz, CDCl_3_) δ 142.6, 132.1, 129.1, 128, 126.1, 125.4. HRMS–ESI (*m/z*): [M + H]^+^ calcd for C_6_H_6_N_3_S^+^, 152.0277; found, 152.028.

#### 4-((Phenylthio)methyl)-1*H*-1,2,3-triazole (**3m**)

Mp: 74.7–76.2 °C. IR (cm^–1^): 3150, 3115, 2841, 1478, 1435, 1223, 1023, 1001, 728, 688. ^1^H NMR (500 MHz, CDCl_3_) δ 7.55 (s, 1H), 7.36–7.31 (m, 2H), 7.28–7.23 (m, 2H), 7.22–7.17 (m, 1H), 4.23 (s, 2H). ^13^C{^1^H} NMR (126 MHz, CDCl_3_) δ 144.3, 135.1, 132.9, 131.1, 130.3, 129.2, 127.0, 126.5, 28.9. HRMS–ESI (*m/z*): [M + H]^+^ calcd for C_9_H_10_N_3_S^+^, 192.0590; found, 192.0595.

#### 4-(Triisopropylsilyl)-1*H*-1,2,3-triazole (**3n**)

Mp: 103.8–105.5 °C. IR (cm^–1^): 3098, 2940, 2863, 1460, 1179, 1105, 1017, 881, 679, 655. ^1^H NMR (500 MHz, CDCl_3_) δ 12.55 (br, 1H), 7.83 (s, 1H), 1.38 (sept, *J* = 7.4 Hz, 3H), 1.10 (d, *J* = 7.5 Hz, 18H). ^13^C{^1^H} NMR (126 MHz, CDCl_3_) δ 139.9, 132.9, 18.6, 11.2. HRMS–ESI (*m/z*): [M + H]^+^ calcd for C_11_H_24_N_3_Si^+^, 226.1734; found, 226.1734.

#### 4-Dodecyl-1*H*-1,2,3-triazole (**3o**)

Mp: 64–67 °C. IR (cm^–1^): 3159, 3113, 2913, 2848, 1470, 1022, 999, 875, 844, 718. ^1^H NMR (500 MHz, CDCl_3_) δ 7.51 (s, 1H), 2.76–2.71 (m, 2H), 1.72–1.64 (m, 2H), 1.40–1.19 (m, 18H), 0.87 (t, *J* = 6.9 Hz, 3H). ^13^C{^1^H} NMR (126 MHz, CDCl_3_) δ 147.2, 131.5, 32.1, 29.84, 29.82, 29.7, 29.54, 29.52, 29.39, 29.35, 25.2, 22.9, 14.3. HRMS–ESI (*m/z*): [M + H]^+^ calcd for C_14_H_28_N_3_^+^, 238.2278; found, 238.2275.

#### 4-(Cyclohex-1-ene-1-yl)-1*H*-1,2,3-triazole (**3p**)

Mp: 90–93 °C. IR (cm^–1^): 3139, 2933, 2860, 1651, 1040, 993, 982, 917, 864, 845. ^1^H NMR (500 MHz, CDCl_3_) δ 7.68 (s, 1H), 6.43–6.39 (m, 1H), 2.46–2.41 (m, 2H), 2.24–2.19 (m, 2H), 1.81–1.75 (m, 2H), 1.71–1.65 (m, 2H). NMR data are in agreement with the literature.^12e^

#### 4-(Tert-butyl)-1*H*-1,2,3-triazole (**3q**)

IR (cm^–1^): 3136, 2962, 2904, 2870, 1707, 1462, 1366, 1206, 1111, 975, 851. ^1^H NMR (500 MHz, CDCl_3_) δ 7.55 (s, 1H), 1.37 (s, 9H). ^13^C{^1^H} NMR (126 MHz, CDCl_3_) δ 155.6, 128.9, 30.7, 30.5. HRMS–ESI (*m/z*): [M + H]^+^ calcd for C_6_H_12_N_3_^+^, 126.1026; found, 126.1029.

#### 4-Propyl-1*H*-1,2,3-triazole (**3r**)

IR (cm^–1^): 3135, 2961, 2933, 2873, 1706, 1460, 1200, 1110, 973, 801. ^1^H NMR (300 MHz, CDCl_3_) δ 9.22 (br, 1H), 7.59 (s, 1H), 2.73 (t, *J* = 7.6 Hz, 2H), 1.79–1.65 (m, 2H), 0.98 (t, *J* = 7.4 Hz, 3H). ^13^C{^1^H} NMR (126 MHz, CDCl_3_) δ 146.2, 130.9, 27.0, 22.6, 13.9. HRMS–ESI (*m/z*): [M + H]^+^ calcd for C_5_H_10_N_3_^+^, 112.0869; found, 112.0870.

#### Androst-4-en-3-one-17*α*-hydroxy-17*β*-(1*H*-1,2,3-triazol-4-yl) (**3s**)

Mp: 233–236 °C. IR (cm^–1^): 3125, 2945, 2904, 22848, 1659, 1607, 1232, 988, 871, 857. ^1^H NMR (500 MHz CDCl_3_) δ 7.55 (s, 1H), 5.72 (s, 1H), 2.44–2.22 (m, 5H), 2.18–2.10 (m, 1H), 1.95–1.80 (m, 3H), 1.66–1.50 (m, 4H), 1.45–1.23 (m, 4H), 1.15 (s, 3H), 1.06 (s, 3H), 1.06–0.95 (m, 2H), 0.72–0.65 (m, 1H), 0.45–0.37 (m, 1H). ^13^C{^1^H} NMR (126 MHz CDCl_3_) δ 199.9, 171.4, 124.1, 82.5, 53.4, 49.1, 47.1, 38.7, 38.3, 36.4, 35.7, 34.1, 33.0, 32.8, 31.8, 23.9, 20.7, 17.6, 14.3. HRMS–ESI (*m/z*): [M + H]^+^ calcd for C_21_H_30_N_3_O_2_^+^, 356.2333; found, 356.2331.

#### Androsta-4,16-dien-3-one-17-(1*H*-1,2,3-triazol-4-yl) (**3t**)

Mp: 235–238 °C. IR (cm^–1^): 3158, 2927, 2859, 1650, 1611, 1234, 957, 872, 841, 780. ^1^H NMR (500 MHz, CDCl_3_) δ 7.71 (s, 1H), 6.21–6.19 (m, 1H), 5.76 (s, 1H), 2.50–1.00 (m, 17H), 1.24 (s, 3H), 1.04 (s, 3H). ^13^C{^1^H} NMR (126 MHz, CDCl_3_) δ 199.9, 171.5, 143.6, 129.2, 124.2, 56.4, 54.3, 47.2, 38.9, 35.7, 35.2, 34.2, 34.1, 33.0, 32.0, 31.9, 21.1, 17.4, 16.4. HRMS–ESI (*m/z*): [M + H]^+^ calcd for C_21_H_28_N_3_O^+^, 338.2227; found, 338.2224.

#### Losartan Analogue **3u**

Mp: 95–97 °C. IR (cm^–1^): 3138, 2955, 2929, 2869, 1575, 1464, 1255, 1005, 965, 765. ^1^H NMR (500 MHz, CDCl_3_) δ 7.82 (br, 1H), 7.48–7.39 (m, 2H), 7.36–7.30 (m, 2H), 7.16 (d, *J* = 8.1 Hz, 2H), 6.97 (d, *J* = 8.1 Hz, 2H), 6.95 (br, 1H), 5.23 (s, 2H), 4.52 (s, 2H), 2.60–2.52 (m, 2H), 1.66–1.57 (m, 2H), 1.35–1.27 (m, 2H), 0.84 (t, *J* = 7.4 Hz, 3H). ^13^C{^1^H} NMR (126 MHz, CDCl_3_) δ 148.8, 141.2, 140.2, 135.4, 130.5, 130.2, 129.5, 128.8, 128.2, 127.4, 126.2, 125.2, 53.0, 47.6, 29.9, 26.9, 22.5, 13.9. HRMS–ESI (*m*/*z*): [M + H]^+^ calcd for C_23_H_25_ClN_5_O^+^, 422.1742; found, 422.1732.

#### Fmoc-L-azahistidine **3w**

Mp: 137–140 °C. IR (cm^–1^): 3066, 2944, 2911, 1696, 1523, 1448, 1233, 1043, 758, 737. ^1^H NMR (500 MHz, DMSO-*d*_*6*_) δ 7.88 (d, *J* = 7.5 Hz, 2H), 7.68–7.63 (m, 2H), 7.54 (s, 1H), 7.41 (t, *J* = 7.5 Hz, 2H), 7.34–7.28 (m, 3H), 4.25–4.16 (m, 3H), 4.13–4.07 (m, 1H), 3.18–3.12 (m, 1H), 3.04–2.99 (m, 1H). ^13^C{^1^H} NMR (126 MHz, DMSO-*d*_6_) δ 173.0, 155.7, 143.87, 143.82, 140.7, 127.6, 127.1, 125.32, 125.26 120.1, 65.6, 54.4, 46.6, 27.0. HRMS–ESI (*m*/*z*): [M + H]^+^ calcd for C_20_H_19_N_4_O_4_^+^, 379.1401; found, 379.1399.

#### Fmoc-*L*-azahistidine-*L*-TrpOMe **3x**

Mp: 202–205 °C. IR (cm^–1^): 3417, 3238, 1730, 1719, 1660, 1530, 1441, 1286, 1219, 1042, 737. ^1^H NMR (500 MHz, DMSO-*d*_*6*_) δ 10.88 (s, 1H), 8.48 (d, *J* = 6.4 Hz, 1H), 7.88 (d, *J* = 7.6 Hz, 2H), 7.66 (t, *J* = 7.3 Hz, 3H), 7.48 (d, *J* = 7.9 Hz, 1H), 7.41 (t, *J* = 7.4 Hz, 2H), 7.35–7.28 (m, 3H), 7.17 (d, *J* = 1.9 Hz, 1H), 7.06 (t, 7.3 Hz, 1H), 6.98 (t, *J* = 7.3 Hz, 1H), 4.56–4.50 (m, 1H), 4.41–4.34 (m, 1H), 4.27–4.20 (m, 1H), 4.20–4.14 (m, 2H), 3.56 (s, 3H), 3.19–3.13 (m, 1H), 3.12–3.04 (m 2H), 2.95–2.88 (m, 1H). ^13^C{^1^H} NMR (126 MHz, DMSO-*d*_*6*_) δ 172.1, 171.3, 155.7, 143.8, 143.7, 140.7, 136.1, 127.7, 127.10, 127.09, 127.05, 125.4, 125.3, 123.8, 121.0, 120.1, 118.5, 118.0, 111.4, 109.2, 65.7, 54.2, 53.2, 51.9, 46.6, 28.0, 26.9. HRMS–ESI (*m*/*z*): [M + H]^+^ calcd for C_32_H_31_N_6_O_5_^+^, 579.2350; found, 579.2343.

## Supporting Information Available

Detailed description of experimental procedures and optimization protocols, compound characterization data, and copies of ^1^H and ^13^C NMR spectra (PDF)
