# Correction to “Copper-Catalyzed Azide–Alkyne Cycloaddition of Hydrazoic Acid Formed *In Situ* from Sodium Azide Affords 4-Monosubstituted-1,2,3-triazoles”

**DOI:** 10.1021/acs.joc.2c01150

**Published:** 2022-06-03

**Authors:** Dominik Jankovič, Miha Virant, Martin Gazvoda

Subsequent to our publication, we received correspondence reinforcing to us the extreme care required in performing chemistry in which HN_3_ is employed. In particular, our assertions about the relative safety of dilute solutions of HN_3_ should not be taken as qualitatively or quantitatively authoritative in all circumstances. Moreover, our statement concerning reference 40b, that “hydrazoic acid was recently used for the large-scale synthesis of an early aryltetrazole intermediate in the synthesis of a drug candidate, giving the developed method the potential for scaling-up,” does not provide sufficient context: We wish to clarify this statement by emphasizing that the publication cited in reference 40b was in fact focused on reducing hydrazoic acid concentrations to <200 ppm. In addition, the procedure presented in reference 40b is dissimilar to the conditions in our manuscript in question, particularly with respect to reaction pH and the strict avoidance of metals. The scalability presented in reference 40b does not imply that our newly published conditions are safe to scale up without extreme care and precautions appropriately attached to HN_3_ and transition metal azides. At the time of submission (May 17, 2022) of this Correction, there were no citations of the article.

