# Supplementary material for: Copper-Catalyzed Azide–Alkyne Cycloaddition of Hydrazoic Acid Formed In Situ from Sodium Azide Affords 4-Monosubstituted-1,2,3-Triazoles
Source: J Org Chem. 2022 Feb 11;87(6):4018–28. doi: 10.1021/acs.joc.1c02775 (PMC8938953; doi:10.1021/acs.joc.1c02775)
Supplement: Supplementary file 1 — jo1c02775_si_001.pdf [file jo1c02775_si_001.pdf]

SUPPORTING INFORMATION

Copper-Catalyzed Azide-Alkyne Cycloaddition of Hydrazoic Acid Formed *In Situ* from Sodium Azide Affords 4-Monosubstituted-1,2,3-Triazoles

Dominik Jankovič, Miha Virant, Martin Gazvoda*

Faculty of Chemistry and Chemical Technology, University of Ljubljana
Večna pot 113, SI-1000 Ljubljana
Slovenia
e-mail: martin.gazvoda@fkkt.uni-lj.si

TABLE OF CONTENT

1. GENERAL INFORMATION 1

1.1 Hydrazoic acid caution..... 1

1.2. General reagent information 1

1.3. General experimental information 2

1.4. General analytical information..... 2

2. EXPERIMENTAL PROCEDURES AND CHARACTERIZATION DATA 4

2.1. Optimization of reaction parameters (Supporting Tables S1-S9) 4

2.2. Monitoring conversion over time on model reaction with phenylacetylene for Method C 13

2.3. Preparation of hydrazoic acid solution by distillation..... 15

2.4. Reactions with distilled hydrazoic acid solution (Supporting Table S10) 16

2.5. Synthesis and characterization of 4-monosubstituted-1,2,3-triazoles (Fig. 3) 17

2.6. Preparation of Tris((1-benzyl-4-triazolyl)methyl)amine (TBTA) 33

2.7. Synthesis and characterization of Fmoc-L-propargylglycine-methyl-L-tryptophanate (methyl ((S)-2-((((9H-fluoren-9-yl)methoxy)carbonyl)amino)pent-4-ynoyl)-L-tryptophanate) (**1x**)..... 34

2.8. Preparation of (2-butyl-4-chloro-1-((2'-ethynyl-[1,1'-biphenyl]-4-yl)methyl)-1*H*-imidazol-5-yl)methanol (**1u**) 35

2.9. Competitive ketone-forming reaction (Fig. 4) 38

2.10. Reactions of azahistidine and peptides (Fig. 5) 42

2.11. Calculation of hydrazoic acid concentration in solution for Method C 60

3. COPIES OF NMR SPECTRA..... 62

4. PHOTOS OF EQUIPMENT USED FOR EXPERIMENTAL WORK 86

5. REFERENCES..... 91

1. GENERAL INFORMATION

1.1 Hydrazoic acid caution

Caution! Hydrazoic acid (HN_3) is toxic if inhaled,[1] and can be explosive if concentrated. It was reported that hydrazoic acid at concertation above 20% (w/w) can be explosive,[2] although ignition source (e.g. spark) is required for detonation of its vapours.[3] Hydrazoic acid solutions with concentration under 10% (w/w) are safe to store and handle.[4] Therefore, to minimize its potential hazards, reactions were performed on low scale (0.5–1.5 mmol) and at low concentration of NaN_3 / hydrazoic acid (max. 6% (w/w)), in closed systems inside the fume hood.

1.2. General reagent information

Copper(II) sulfate pentahydrate, copper(I) bromide, copper(I) iodide, copper(II) acetate, *N*-(3-dimethylaminopropyl)-*N′*-ethylcarbodiimide hydrochloride (EDC×HCl) and methyl-L-tryptophanate were purchased from Fluka .

Bromotris(triphenylphosphine)copper(I), copper(I) chloride, 2,2'-bipyridyl (bipy), (1,4-diazabicyclo[2.2.2]octane) (DABCO), tris(2-carboxyethyl)phosphine (TCEP), triphenylphosphine, nitrilotriacetic acid (NTA), tripropargylamine, sodium azide, sodium ascorbate, 1,3,5-trimethoxybenzene, sodium hydroxide, 1,2-phenylenediamine, ammonium chloride, anhydrous sodium sulfate, *p*-toluensulfonic acid, acetic acid, methanoic acid, tris((1-hydroxy-propyl-1*H*-1,2,3-triazol-4-yl)methyl)amine (THPTA), 2-bromoiodobenzene, triisopropyl borate, *n*-butyllithium, 2-butyl-5-chloro-1*H*-imidazole-4-carboxaldehyde, 4-bromobenzyl bromide, potassium carbonate, sodium borohydrate, 2,4-lutidine and phosphorus(V) oxychloride, phenyl propargyl sulfide (**1m**), (triisopropylsilyl)acetylene (**1n**), 1-tetradecyne (**1o**), 1-ethynylcyclohexene (**1p**), 1-pentyne (**1r**), diphenylacetylene (**1v**), ethisterone (**1s**) were purchased from Sigma-Aldrich.

1,10'-Phenanthroline, trimethylsilylacetylene, palladium acetate and tetra-*n*-butylammonium fluoride (TBAF), phenylacetylene (**1a**), 1-ethynyl-4-(trifluoromethyl)benzene (**1b**), 4-ethynylbenzonitrile (**1e**), 1-bromo-4-ethynylbenzene (**1f**), 1-ethynyl-4-nitrobenzene (**1g**), 4-ethynylaniline (**1j**), 2-ethynylpyridine (**1k**), 2-ethynyltiophene (**1l**), and Fmoc-L-propargylglycine (**1w**) were purchased from FluoroChem.

Benzyl azide, 1-ethynyl-4-methylbenzene (**1c**), 1-ethynyl-4-moethoxybenzene (**1d**), 1-ethynyl-2-fluorobenzene (**1h**) and 3,3-dimethyl-1-butyne (**1q**) were purchased from Alfa Aesar.

2-Ethoxy-4-ethynyl-1-methoxybenzene (**1i**) was purchased from Apollo Scientific.

Custome made peptides **1aa** and **1y** were purchased from NovoPep Limited. Peptide **1z** was purchased from KS-V Peptide Biological Technology Co. Ltd.

Solvents were purchased from Honeywell, Fluorochem, Carlo Erba, J. T. Baker and Fisher Scientific. Solvents used were not pre-dried or distilled. All deuterated solvents were purchased from Euriso-top and were used without further purification.

Tris(2-benzimidazolylmethyl)amine ($(BimH)_3$) was prepared according to procedure from literature.[5]

Pregna-4,16-dien-20-yn-3-one (**1t**) was prepared according to literature.[6]

Fmoc-L-propargylglycine-methyl-L-tryptophanate (methyl ((S)-2-((((9H-fluoren-9-yl)methoxy)carbonyl)amino)pent-4-ynoyl)-L-tryptophanate) (**1x**) was prepared to slightly modified procedure from the literature.[7] See section 2.7.

Tris((1-benzyl-4-triazolyl)methyl)amine (TBTA) was prepared to slightly modified procedure from the literature.[8] See section 2.6.

Synthesis of acetylene substrate (2-butyl-4-chloro-1-((2'-ethynyl-[1,1'-biphenyl]-4-yl)methyl)-1*H*-imidazol-5-yl)methanol (**1u**) is described in section 2.8.

1.3. General experimental information

All reactions were performed under ambient atmosphere (unless noted otherwise). Reactions at elevated temperatures were conducted in ~8 mL or ~21 mL Merck's Ace pressure tubes (Cat. No. Z564621 and Z564583) with a screw cap in a heating block (see Supporting Photo S4), whereas reactions at room temperature were performed in 5 mL round bottom flasks.

1.4. General analytical information

NMR spectra were recorded with Bruker Avance III 500 MHz NMR instrument at 300 K operating at 500 MHz (^{1}H) and 126 MHz (^{13}C). Proton spectra were referenced to residual $CHCl_3$ in deuterated chloroform (δ = 7.26 ppm) and DMSO-d_5 in DMSO-d_6 (δ = 2.50 ppm). Carbon spectra were referenced to the central line of ^{13}C signal of $CDCl_3$ (δ = 77.2 ppm) in deuterated chloroform and $(CD_3)SO$ (δ = 39.52 ppm) in DMSO-d_6. Chemical shifts (δ) are given in ppm. Coupling constants are given in Hz. Multiplicities are indicated as: s (singlet), d (doublet), t (triplet), q (quartet), quint (quintet), sept (septet), m (multiplet) and br (broadened).

IR spectra were recorded with Bruker ALPHA FT-IR spectrometer with Platinum ATR module.

Melting points were determined with Kofler microscope with hot bench Leica Galen III. Melting points are given as obtained and are not corrected.

High resolution mass spectra (HRMS) were recorded on time-of-flight Agilent Accurate Mass TOF LC/MS spectrometer (Agilent 6224) equipped with a double orthogonal electrospray source at atmospheric pressure ionization (ESI+).

HPLC analyses of peptide reactions were performed with Agilent Infinity II 1260 LC system. Atlantis® dC18, 5 μm, 4.6 x 250 mm column was used. Mobile phases A: H_2O + 0.1% trifluoroacetic acid, B: Acetonitrile + 0.1% trifluoroacetic acid. Elution program as follows: 00:00–25:00 gradient elution; 100% A → 100% B, 25:00–30:00 isocratic elution; 100% B. System was purged with 100% A for 5 minutes between runs. Flow rate: 1.0 mL/min. 10 μL of sample was injected, UV detection at 220 nm.

HPLC-HRMS analyses of peptide reactions were done with Agilent 1260 Infinity HPLC instrument coupled with Agilent Accurate Mass TOF LC/MS HRMS instrument. Atlantis® dC18, 5 μm, 4.6 x 250 mm column was used. Mobile phases A: H_2O + 0.1% methanoic acid, B: acetonitrile + 0.1% methanoic acid. Elution program as follows: 00:00–25:00 gradient elution; 100% A → 100% B, 25:00–30:00 isocratic elution; 100% B. System was purged with 100% A for 5 minutes between runs. Flow rate: 1.0 mL/min, 1 μL of sample was injected. Detection with HRMS TOF detector equipped with a double orthogonal electrospray source at atmospheric pressure ionization (ESI+) in range 100–1100 m/z.

Fluka analytical TLC plates were used for thin-layer chromatography. UV-light at 254 nm was used for detection. Silica gel column chromatography was carried out on Fluka Silica gel 60N, mesh 220-240 and Interchim puriFlash® XS520 using various silica columns (specifics are given at each example).

2. EXPERIMENTAL PROCEDURES AND CHARACTERIZATION DATA

2.1. Optimization of reaction parameters (Supporting Tables S1-S9)

Development of reaction conditions for Method A:

Supporting Table S1. Results of acid-screening experiments.

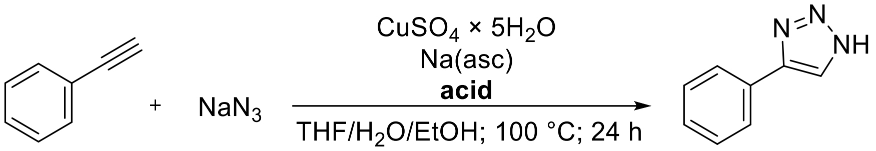

Entry	Acid	Yield [%]
1	HCl	62
2	H_2SO_4	76
3	*p*-TsOH	38
4	-	25

Reaction conditions: phenylacetylene (1.0 mmol), NaN_3 (1.5 mmol), acid (1.6 mmol), $CuSO_4 \times 5H_2O$ (20 mol%), sodium ascorbate (1.0 mmol), THF:H_2O:EtOH 2:2:1 (v/v/v, 2.5 mL). Yield of purified material after column chromatography is given.

Comment on Supporting Table S1: Sulfuric acid turned out to be most applicable for investigated system (entry 2).

Supporting Table S2. Results of temperature-screening experiments.

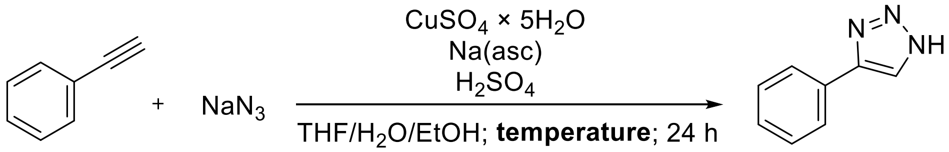

Entry	Temperature [°C]	Yield [%]
1	60	35
2	80	49
3	100	76
4[a]	r.t.	5[b]
5[a]	40	35[b]
6[a]	60	67[b]
7[a]	100	79[b]

Reaction conditions: phenylacetylene (1.0 mmol), NaN_3 (1.5 mmol), acid (1.6 mmol), $CuSO_4 \times 5H_2O$ (20 mol%), sodium ascorbate (1.0 mmol), THF:H_2O:EtOH 2:2:1 (v/v/v, 2.5 mL). Yield of purified material after column chromatography is given. [a]Reaction with 5 equiv. of NaN_3: phenylacetylene (0.15 mmol), NaN_3 (0.75 mmol), H_2SO_4 (0.825 mmol), $CuSO_4 \times 5H_2O$ (20 mol%), Na(asc) (0.15 mmol),

THF:H_2O:EtOH 2:2:1 (v/v/v, 1.25 mL). [b]Conversion was determined by ^{1}H NMR spectroscopy by using 1,3,5-trimethoxybenzene (TMB) as internal standard.

Comment on Supporting Table S2: Although CuAAC reactions usually proceed at low temperatures, it turned out high temperature is needed for our investigated system to achieve high conversion (entry 3). Increasing amount of NaN_3 (5 equiv.) did not increase the conversion into triazole product (entry 7).

Supporting Table S3. Results of catalyst-screening experiments.

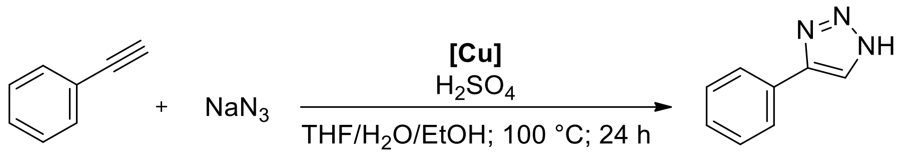

Entry	[Cu]	Yield [%]
1	$CuSO_4 \times 5H_2O$ + Na(asc)	76
2	$(CH_3COO)_2Cu$ + Na(asc)	61
3	$Cu(PPh_3)_3Br$	32
4	CuCl	39
5	CuBr	34
6	CuCl + Na(asc)	64
7	-	14[a]

Reaction conditions: phenylacetylene (1.0 mmol), NaN_3 (1.5 mmol), acid (1.6 mmol), [Cu] (20 mol%), sodium ascorbate (1.0 mmol), THF:H_2O:EtOH 2:2:1 (v/v/v, 2.5 mL). Yield of purified material after column chromatography is given. [a]Crude yield after extraction with EtOAc and aqueous NH_4Cl solution.

Comment on Supporting Table S3: $CuSO_4 \times 5H_2O$ + reducing agent (sodium ascorbate) system turned out to be the most appropriate for optimal conversions (entry 1). Cu(I) tends to oxidize to Cu(II) at ambient atmosphere, thus lowering catalytic activity (compare entries 4, 6).

Supporting Table S4. Results of catalyst-loading expleriments.

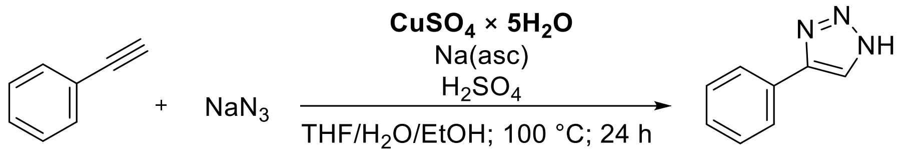

Entry	mol% of $CuSO_4 \times 5H_2O$	Yield [%]
1	5	42
2	10	64
3	20	76
4	30	64

Reaction conditions: phenylacetylene (1.0 mmol), NaN_3 (1.5 mmol), acid (1.6 mmol), $CuSO_4$, sodium ascorbate (1.0 mmol), THF:H_2O:EtOH 2:2:1 (v/v/v, 2.5 mL). Yield of purified material after column chromatography is given.

Supporting Table S5. Results of reaction time screening experiments.

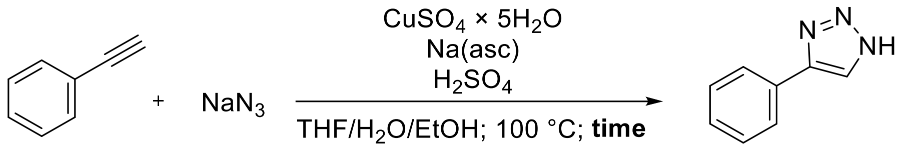

Entry	Time [h]	Yield [%]
1	3	36
2	6	53
3	14	72
4	24	76
5	48	74

Reaction conditions: phenylacetylene (1.0 mmol), NaN_3 (1.5 mmol), acid (1.6 mmol), $CuSO_4 \times 5H_2O$ (20 mol%), sodium ascorbate (1.0 mmol), THF:H_2O:EtOH 2:2:1 (v/v/v, 2.5 mL). Yield of purified material after column chromatography is given.

Comment on Supporting Table S5: Although reaction does not proceed further after 14 hours (entry 3), 24 hours reaction time was selected (entry 4) for furhter screenings. Prolonged reaction times did not result better conversions (entry 5).

Supporting Table S6. Results of solvent-screening experiments.

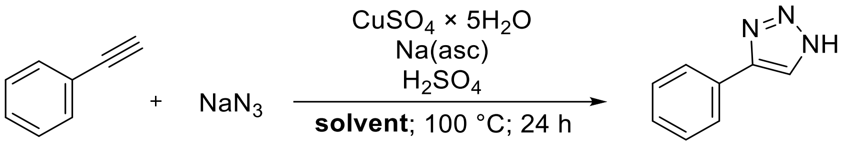

Entry	Solvent[a] [volume ratio]	Conversion [%]
1	H_2O	19
2	H_2O/EtOH [1/1]	48
3	THF/EtOH [1/1]	41
4	THF/H_2O [1/1]	43
5	THF/H_2O/EtOH [2/2/1]	76[b]
6	*t*-BuOH/H_2O/EtOH [1/1/2]	72
7	CH_3CN/H_2O [1/1]	74
8	CH_3CN/H_2O/EtOH [2/2/1]	74
9[c]	DMF/MeOH [5/1]	84[b]

Reaction conditions: phenylacetylene (1.0 mmol), NaN_3 (1.5 mmol), acid (1.6 mmol), $CuSO_4 \times 5H_2O$ (20 mol%), sodium ascorbate (1.0 mmol). [a]2.5 mL of solvent in given ratio. Conversion is determined by ^1H NMR spectroscopy using 1,3,5-trimethoxybenzene (TMB) as internal standard. [b]Yield of purified material after column chromatography. [c]20 mol% of CuI as precatalyst.

Comment on Supporting Table S6: Multiple solvent mixtures were examined. Reaction proceeded the best in DMF/MeOH solvent system with CuI as catalyst (entry 9) and enviromentally friendlier THF/H_2O/EtOH solvent system with $CuSO_4$ catalyst (entry 5). These conditions were selected for substrate scope screening and we described them as Method A.

Development of reaction conditions for Method B:

Supporting Table S7. Results of tris[(1-benzyl-1*H*-1,2,3-triazol-4-yl)methyl]amine (TBTA)-accelerated experiments.

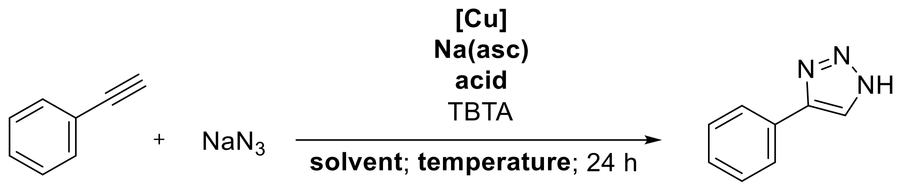

Entry	Cu [mol%]	Equiv. of Na(asc)	Acid [equiv.]	Mol% TBTA	Solvent [mL]	Temp. [°C]	Conversion [%]
1	CuI [20]	1	H_2SO_4 [1.6]	10	DMF [1.25]	60	96
2	CuI [20]	1	H_2SO_4 [1.6]	10	DMF [1.25]	r.t.	51/73[b]/88[c]
3	CuI [20]	-	H_2SO_4 [1.6]	10	DMF [1.25]	60	8
4	$CuSO_4 \times 5H_2O$ [5]	1	H_2SO_4 [1.6]	10	THF/H_2O/EtOH [1.25][a]	60	68
5	CuI [20]	-	H_2SO_4 [1.6]	10	THF/H_2O/EtOH [1.25][a]	60	32
6	CuI [20]	-	HCOOH [5]	10	DMF [1.25]	60	79
7	CuI [10]	1	H_2SO_4 [1.6]	5	DMF [1.25]	60	89/89[c]/88[d]
8	CuI [5]	1	H_2SO_4 [1.6]	2.5	DMF [1.25]	60	89/89[c]
9	CuI [10]	1	-	5	DMF [1.25]	60	6
10	CuI [20]	1	H_2SO_4 [1.6]	10	DMF [0.6]	r.t.	85/85[c]
11	CuI [5]	1	HCl [1.6]	2.5	DMF [0.6]	60	85
12	CuI [5]	-	HCl [1.6]	2.5	DMF [0.6]	60	40
13	CuI [5]	-	HCOOH [5]	2.5	DMF [0.6]	60	77
14	CuI [5]	1	H_2SO_4 [1.6]	2.5	DMF [0.6]	r.t.	35/70[b]
15	CuI [5]	-	HCOOH [5]	2.5	DMF [0.6]	r.t.	0
16	CuI [5]	-	HCOOH [5]	2.5	DMF [0.6]	40	43[c]

17	CuI [10]	-	HCOOH [5]	5	DMF [0.6]	40	50[c]
18	CuI [5]	0.2	HCOOH [5]	2.5	DMF [0.6]	r.t	75[c]
19	CuI [5]	0.2	HCOOH [5]	2.5	DMF [0.6]	40	90/89[d]

Reaction conditions: phenylacetylene (0.5 mmol), NaN_3 (0.75 mmol), TBTA: Tris[(1-benzyl-1*H*-1,2,3-triazol-4-yl)methyl]amine. [a]Volume ratio [2/2/1]. Conversion is determined by ^{1}H NMR spectroscopy using 1,3,5-trimethoxybenzene (TMB) as internal standard. [b]Conversion after 48 hours. [c]Conversion after 72 hours. [d]Yield after purification with column chromatography.

Comment on Supporting Table S7: By using tris[(1-benzyl-1*H*-1,2,3-triazol-4-yl)methyl]amine (TBTA) milder overall reaction conditions were achieved (acid and temperature). Developed conditions (entry 19) were selected for substrate scope screening and we described them as Method B. We aimed to develop synthetic protocol that would be operate under ambient atmospehere and even with strong affinity of TBTA towards Cu(I) we still needed to add reducing agent (sodium ascorbate) to regenerate Cu(I) from Cu(II) (compare entries 1 and 3). We found that methanoic acid (HCOOH) can also acts as a reducing agent of Cu(II), enabeling the reaction without sodium ascorbate. At 60 °C good conversions were obtained without sodium ascorbate (entries 6 and 13), whereas at 40 °C and room temperature methanoic acid is not sufficient reducing agent (entries 15, 16, 17). By employing TBTA we were able to lower Cu-catalyst loading to 5 mol%.

Development of reaction conditions for Method C:

Supporting Table S8. Results of tris(2-benzimidazolylmethyl)amine ((BimH)$_3$)-accelerated experiments.

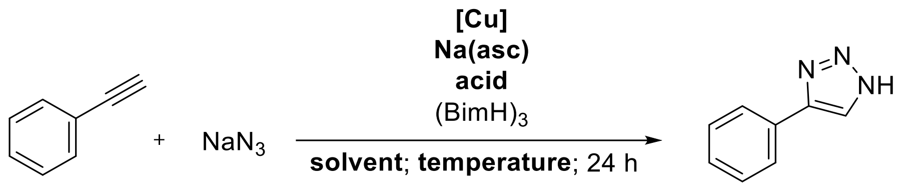

Entry	Cu	Equiv. of Na(asc)	Acid [equiv.]	Solvent [volume ratio]	Temp. [°C]	Conversion [%]
1	CuI	0.2	Acetic acid [3]	DMF	40	61
2	CuI	0.2	Acetic acid [3]	DMF	r.t.	24
3	CuBr	0.2	Acetic acid [3]	EtOH	40	83
4	$CuSO_4 \times 5H_2O$	0.25	HCOOH [2]	$MeOH/H_2O$ [3/1]	40	97
5	$CuSO_4 \times 5H_2O$	0.25	HCOOH [3]	$MeOH/H_2O$ [3/1]	r.t.	99
6	$CuSO_4 \times 5H_2O$	1	Acetic acid [3]	$MeOH/H_2O$ [3/1]	40	80/91[a]
7	$CuSO_4 \times 5H_2O$	0.25	Acetic acid [2]	$MeOH/H_2O$ [3/1]	40	74
8	$CuSO_4 \times 5H_2O$	0.25	Acetic acid [6.6]	$MeOH/H_2O$ [3/1]	r.t.	100/99[c]
9	$CuSO_4 \times 5H_2O$	0.25	Lactic acid [2]	$MeOH/H_2O$ [3/1]	r.t.	90
10	$CuSO_4 \times 5H_2O$	0.25	Lactic acid [5]	$MeOH/H_2O$ [3/1]	r.t.	83
11	$CuSO_4 \times 5H_2O$	0.25	TFA [2]	$MeOH/H_2O$ [3/1]	r.t.	95
12	$CuSO_4 \times 5H_2O$	0.25	Acetate buffer pH 3.7	$MeOH/H_2O$[d] [2/1]	r.t.	39
13	CuBr	0.25	HCOOH [3]	DMF/H_2O [5/1]	r.t.	95[b]
14	$CuSO_4 \times 5H_2O$	0[e]	Acetic acid [6.6]	$MeOH/H_2O$ [4/1]	r.t.	<1%

Reaction conditions: phenylacetylene (0.5 mmol), NaN_3 (0.75 mmol), [Cu] (5 mol%), tris(2-benzimidazolylmethyl)amine (BimH)$_3$ (5 mol%), TFA: trifluoroacetic acid. 0.6 mL of solvent in given ratio. Conversion was determined by ^{1}H NMR spectroscopy using 1,3,5-trimethoxybenzene (TMB) as internal standard. [a]Conversion after 48 hours. [b]Conversion after 72 hours. [c]Yield after purification by column chromatography. [d]0.2 mL AcOH/NaOAc buffer. [e]25 mol% of tris(2-carboxyethyl)phosphine (TCEP) was used instead of sodium ascorbate.

Comment on Supporting Table S8: By using tris(2-benzimidazolylmethyl)amine $(BimH)_3$ we achieved milder reaction conditions regarding employed acids and reaction temperature. Developed conditions (entry 8) were selected for substrate scope screening and we described them as Method C. By using $(BimH)_3$ we were able to lower reaction temperature to room temperature. We explored mild organic acids and wanted to use solvent mixture that provided easier work-up and purification process, and would follow recommendations of CHEM21 selection guide of classical- and less classical-solvents.[9] Notewhorty, by using acetic acid (6.6 equiv.), methanoic acid (3 equiv.) and lactic (2 equiv.) or trifluoroacetic acid (2 equiv.) we were able to obtain quantitative or almost quantitative conversions at room temperature (entries 5, 8, 9, 11).

Supporting Table S9. Results from other ligand-accelerated experiments.

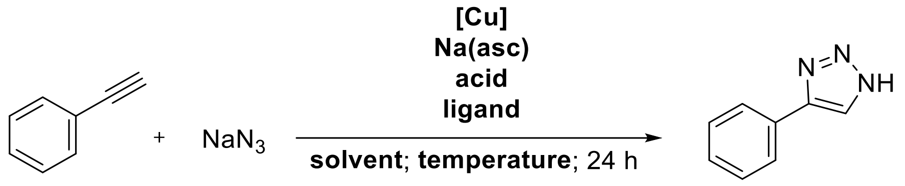

Entry	Cu [mol%]	Equiv. of Na(asc)	Acid [equiv.]	Ligand [mol%]	Solvent [volume ratio]	Temp. [°C]	Conversion [%]
1	CuI [5]	0.2	Acetic acid [2]	DABCO [5]	DMF	40	63
2	CuI [5]	0.2	Acetic acid [3]	Bipy [10]	DMF	40	67
3	CuI [5]	-	Oxalic acid [1.5]	PPh_3 [2.5]	DMF	40	0
4	CuI [5]	0.2	Acetic acid [3]	PPh_3 [10]	DMF	40	43
5	CuI [5]	-	Oxalic acid [1.5]	Phen [2.5]	DMF	40	0
6	CuI [5]	0.2	Acetic acid [3]	Phen [2.5]	DMF	r.t.	32
7	CuI [5]	0.2	Acetic acid [3]	Phen [2.5]	DMF	40	71[b]
8	CuI [5]	0.2	Acetic acid [3]	Phen [5]	DMF	40	73/72[b]
9	CuI [5]	0.2	Acetic acid [3]	Phen [5]	DMF	r.t.	70[a]
10	CuI [5]	0.5	Acetic acid [3]	Phen [10]	DMF	40	72
11	$CuSO_4 \times 5H_2O$ [5]	1	Acetic acid [3]	Phen [10]	THF/H_2O/EtOH [2/2/1]	40	80
12	$CuSO_4 \times 5H_2O$ [10]	-	-	TCEP [200]	MeOH/H_2O [3/1]	60	0
13	$CuSO_4 \times 5H_2O$ [10]	-	Acetic acid [6.6]	TCEP [100]	MeOH/H_2O [3/1]	60	0
14	$CuSO_4 \times 5H_2O$ [10]	-	NTA [3]	-	MeOH/H_2O [3/1]	60	0

Reaction conditions: phenylacetylene (0.5 mmol), NaN_3 (1.5 mmol), TCEP: (tris(2-carboxyethyl)phosphine), NTA: nitrilotriacetic acid, 0.6 mL of solvent in given ratio. Conversion was determined by ^{1}H NMR spectroscopy using 1,3,5-trimethoxybenzene (TMB) as internal standard. [a]Conversion after 72 hours. [b]Yield after purification with column chromatography.

Comment on Supporting Table S9: Although tris[(1-benzyl-1*H*-1,2,3-triazol-4-yl)methyl]amine (TBTA) and tris(2-benzimidazolylmethyl)amine $(BimH)_3$ provided good results, we also investigated other standard ligands for copper-catalysed reactions, including CuAAC, as well as miscellaneous multipurpose aditives. Aditives that would have the potential to act simultaneously as acid, reducing agent, and ligand, such as TCEP, were also investigated. Among examined ligands, besides TBTA and $(BimH)_3$, 1,10'-Phenathroline also provided good results (entries 7–11). When using potential multipurpose aditives such as (tris(2-carboxyethyl)phosphine) (TCEP) and nitrilotriacetic acid (NTA) we did not observe any conversion into triazole product (entries 12–14).

2.2. Monitoring conversion over time on model reaction with phenylacetylene for Method C

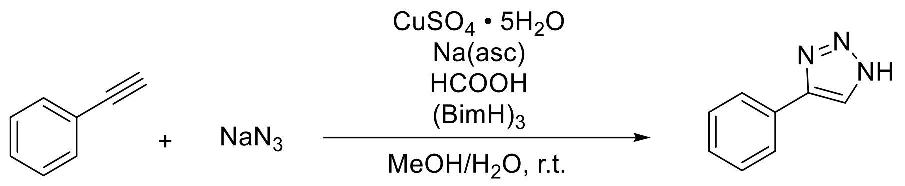

Procedure: Phenylacetylene (51 mg, 0.5 mmol), $CuSO_4\times5H_2O$ (6.3 mg, 0.025 mmol, 5 mol%), tris(2-benzimidazolylmethyl)amine ($(BimH)_3$) (10.2 mg, 0.025 mmol, 5 mol%), Na(asc) (25 mg, 0.125 mmol, 25 mol%), NaN_3 (49 mg, 0.75 mmol, 1.5 equiv.) and 1,3,5-trimethoxybenzene (TMB) internal standard (15.8 mg, 0.09394 mmol) were added to 5 mL round bottom flask containing 0.450 mL of MeOH and 0.150 mL of H_2O. HCOOH (57 μL, 70 mg, 1.5 mmol, 3 equiv.) was added and the flask was sealed with septum. Aliquotes of reaction mixture were taken by syringe (25 μL), diluted with $CDCl_3$ (0.6 mL) and conversion into triazole product was determined by ^{1}H NMR by comparing integrals of indicative ArC*H* resonance of 4-phenyl-1,2,3-triazole at δ 7.77 ppm (d, 2H) and of internal TMB standard at δ 6.06 ppm (s, 3H).

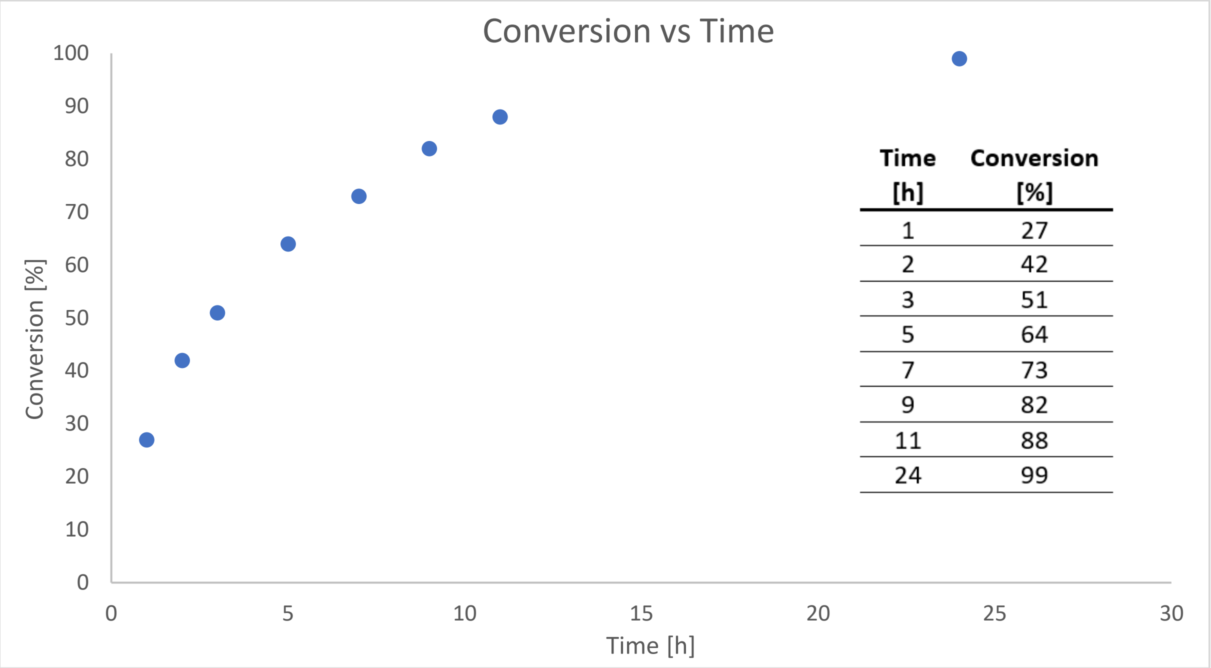

Supporting Figure S1. Conversion of phenylacetylene into 4-phenyl-1,2,3-triazole over time.

2.3. Preparation of hydrazoic acid solution by distillation

CAUTION! Pure hydrazoic acid is explosive and its vapours are toxic (bp. 37 °C). Preparation must be done carefully in a closed system under the fume hood.

Procedure: Sodium hydroxide (250 mg, 6.25 mmol) was dissolved in 7.5 mL of water in 10 mL two necked round bottom distilling flask and sodium azide (747 mg, 11.5 mmol) was added. *Note: Sodium hydroxide is added to prevent cold formation of hydrazoic acid*. Distilling flask was fitted with septum and a condenser with a short distilling path. At the end of condenser 25 mL recieving flask containing 5 mL of cold water was attached and was cooled on ice-bath during distillation process. Solution in the distilling flask was brought to boil. As first drops of distilling water passed trough condenser 4.5 mL of 40% sulfuric acid (23.9 mmol) was added dropwise. After the addition of sulfuric acid, distillation was continued until about 2.5 mL of solution was left in the distilling flask. Approximatelly 10 mL total volume of hydrazoic acid solution was obtained. Concentration of hydrazoic acid solution was determined by titration with 0.05 M NaOH solution using phenolphtalein as indicator. This procedure was repeated twice, determined concentrations were: 0.504 M (first run), 0.623 M (second run).

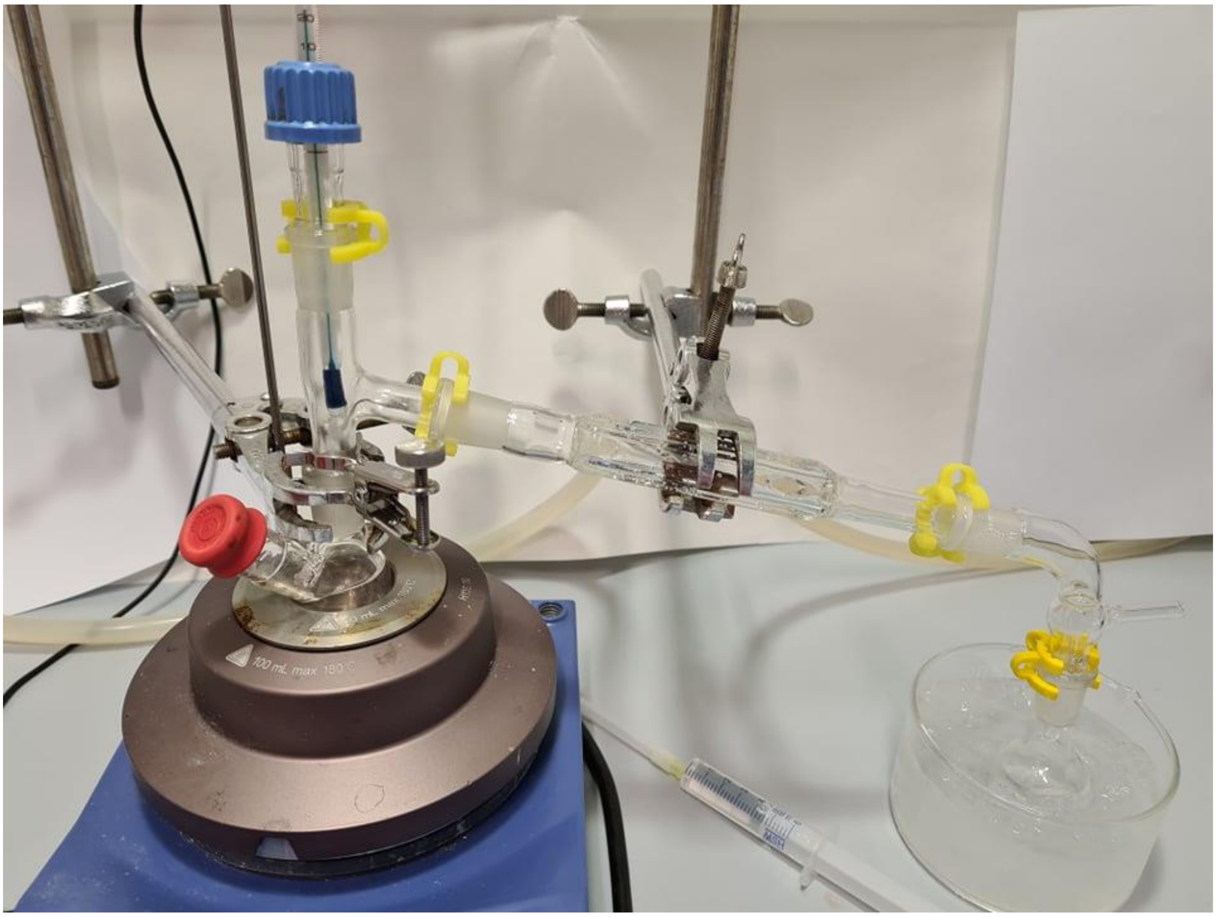

Supporting Photo S1. Apparatus for distillation of hydrazoic acid.

2.4. Reactions with distilled hydrazoic acid solution (Supporting Table S10)

Supporting Table S10. Results of reactions with distilled hydrazoic acid solution.

Hydrazoic acid was introduced into reaction as aqueous solution.

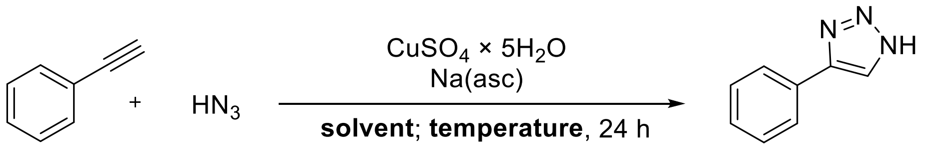

Entry	Solvent [volume ratio]	Temperature [°C]	Conversion [%]
1	THF/H_2O/EtOH [2/2/1]	r.t.	1
2	THF/H_2O/EtOH [2/2/1]	40	34
3	THF/H_2O/EtOH [2/2/1]	40	34[a]
4	THF/H_2O/EtOH [2/2/1]	60	38
5	THF/H_2O/EtOH [2/2/1]	80	50
6	THF/H_2O/EtOH [2/2/1]	100	78
7[b]	THF/H_2O/EtOH [2/2/1]	100	14
8[c]	THF/H_2O/EtOH [2/2/1]	100	72/70[d]
9[e]	MeOH/H_2O [3/1]	r.t.	92

Reaction conditions: HN_3 (0.5 mmol, 1 mL 0.5 M aqueous solution), phenylacetylene (1 mmol), $CuSO_4$×$5H_2O$ (20 mol%), sodium ascorbate (1 mmol), THF:H_2O:EtOH 2:2:1 (v/v/v, 2.5 mL). Conversion was determined by ^1H NMR spectroscopy using 1,3,5-trimethoxybenzene (TMB) as internal standard. [a]Reaction time 72 h. [b]Reaction without copper catalyst and sodium ascorbate. [c]Reaction conditions; excess HN_3: phenylacetylene (0.17 mmol), HN_3 (0.5 mmol, 3 equiv., 1 mL 0.5 M aqueous solution), $CuSO_4$×$5H_2O$ (20 mol%), sodium ascorbate (0.17 mmol). [d]Yield after purification by column chromatography. [e]Reaction conditions with excess of HN_3: phenylacetylene (0.16 mmol), HN_3 (0.31 mmol, 1.9 equiv., 0.5 mL 0.62 M aqueous solution), $CuSO_4$×$5H_2O$ (5 mol%), $(BimH)_3$ (5 mol%), sodium ascorbate (0.04 mmol, 0.25 equiv.), MeOH:H_2O 3:1 (v/v, 2 mL).

2.5. Synthesis and characterization of 4-monosubstituted-1,2,3-triazoles (Fig. 3)

General procedure for Method A:

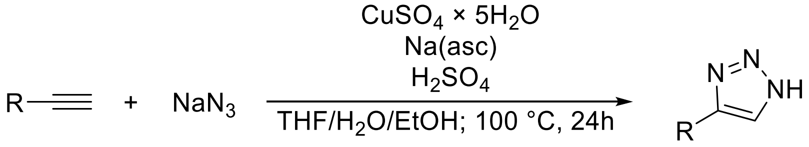

Into a 8 or 21 mL ACE pressure tube stirring bar, alkyne (1 mmol, 1 equiv.), $CuSO_4 \times 5H_2O$ (50 mg, 0.2 mmol, 20 mol%), sodium ascorbate (198 mg, 1 mmol), NaN_3 (98 mg, 1.5 mmol), solvent mixture THF:H_2O:EtOH 2:2:1 (v/v/v) and 96% H_2SO_4 (89 μL, 164 mg, 1.6 mmol), were added in that order. After addition of sulfuric acid, ACE tube was sealed with a screw cap and reaction mixture was stirred for 24 hours at 100 °C in aluminum heating block (see Supporting picture S4). After that time, the reaction mixture was allowed to cool to room temperature in a closed ACE pressure tube. Prior to work-up, ACE tube was uncapped and left open for a few minutes in the fume hood to allow any excess hydrazoic acid in vapor phase to evaporate. To reaction mixture were added ethyl acetate or dichloromethane (30 mL) and ammonium chloride solution (20 mL sat. NH_4Cl + 20 mL H_2O), and phases were separated in separatory funnel. Aqueous phase was additionally extracted with ethyl acetate (2 × 30 mL). Organic phases were combined and dried over anhydrous Na_2SO_4, filtered and the solvent was removed with the aid of rotary evaporator. Crude product was purified by silica column chromatography (mobile phase CH_2Cl_2:acetone 5:1) unless otherwise noted.

General procedure for Method A with CuI catalyst and DMF/MeOH solvent system:

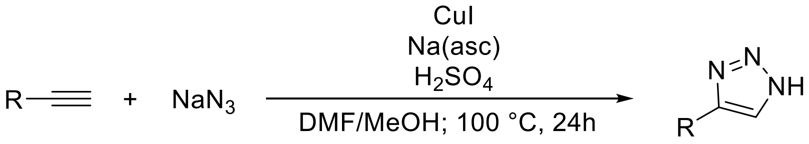

Into a 8 or 21 mL ACE pressure tube stirring bar, alkyne (1 mmol or 0.5 mmol scale, 1 equiv.), CuI (20 mol%), sodium ascorbate (1 equiv.), NaN_3 (1.5 equiv.), solvent mixture DMF:MeOH 5:1 (v/v) and 96% H_2SO_4 (1.6 equiv.), were added in that order. After addition of sulfuric acid, ACE tube was sealed with a screw cap and reaction mixture was stirred for 24 hours at 100 °C in aluminum heating block. After that time, the reaction mixture was allowed to cool to room temperature in a closed ACE pressure tube. Prior to work-up, ACE tube was uncapped and left open for a few minutes in the fume hood to allow any excess hydrazoic acid in vapor phase to evaporate. Reaction mixture was filtered trough a short pad of silica gel by using ethyl acetate as elunet. Resulting filtrate was concentrated with the aid of rotary evaporator and crude product was purified by silica gel column chromatography using Interchim puriFlash® XS520 (Column: Interchim PF-50SIHC-F0004 (4 g, 50 μm SiO_2), gradient elution: 00:00–

20:00: 100% petroleum ether → 100% ethyl acetate; 20:00–25:00: 100% ethyl acetate, flow rate: 5 mL/min).

General procedure for Method B:

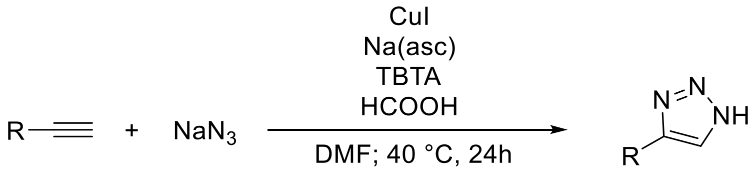

Into a 8 mL ACE pressure tube stirring bar, alkyne (0.5 mmol, 1 equiv.), CuI (4.8 mg, 0.025 mmol, 5 mol%), sodium ascorbate (20 mg, 0.1 mmol, 0.2 equiv.), tris[(1-benzyl-1*H*-1,2,3-triazol-4-yl)methyl]amine (TBTA) (6.6 mg, 0.0125 mmol, 2.5 mol%), NaN_3 (49 mg, 0.75 mmol, 1.5 equiv), DMF (0.6 mL) and HCOOH (94 µL, 115 mg, 2.5 mmol, 5 equiv.) were added in that order. After addition of methanoic acid, ACE tube was sealed with a screw cap and reaction mixture stirred for 24 hours at 40 °C in aluminum heating block. Then, the reaction tube was uncapped and the reaction mixture was filtered trough a short pad of silica gel using ethyl acetate as eluent. Resulting filtrate was concentrated with the aid of rotary evaporator and the crude product was purified by silica gel column chromatography using Interchim puriFlash® XS520, unless noted otherwise. (Column: Interchim PF-50SIHC-F0004 (4 g, 50 µm SiO_2), gradient elution: 00:00–20:00: 100% petroleum ether → 100% ethyl acetate; 20:00–25:00: 100% ethyl acetate, flow rate: 5 mL/min).

General procedure for Method C:

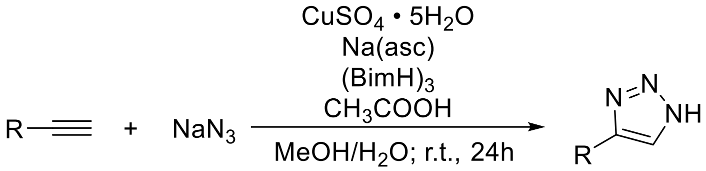

Into a 5 mL round bottom flask stirring bar, alkyne (0.5 mmol, 1 equiv.), $CuSO_4{\times}5H_2O$ (6.2 mg, 0.025 mmol, 5 mol%), sodium ascorbate (25 mg, 0.125 mmol, 0.25 equiv.), tris(2-benzimidazolylmethyl)amine ($(BimH)_3$) (10 mg, 0.025 mmol, 5 mol%), NaN_3 (49 mg, 0.75 mmol, 1.5 equiv.), solvent MeOH:H_2O 3:1 (v/v, 0.6 mL) and CH_3COOH (189 µL, 198 mg, 3.3 mmol, 6.6 equiv) were added in that order. After the addition of acetic acid flask was capped with glass stopper and the reaction mixture was stirred for 24 hours at room temperature. Then, the reaction flask was uncapped and the reaction mixture was filtered trough a short pad of silica gel using ethyl acetate as eluent. Resulting filtrate was concentrated with the aid of rotary evaporator and the crude product was purified by silica gel column chromatography using Interchim puriFlash® XS520 system, unless noted otherwise.

(Column: Interchim PF-50SIHC-F0004 (4 g, 50 μm SiO_2), gradient elution: 00:00–20:00: 100% petroleum ether → 100% ethyl acetate; 20:00–25:00: 100% ethyl acetate, flow rate: 5 mL/min).

4-Phenyl-1*H*-1,2,3-triazole (3a, Fig. 3)

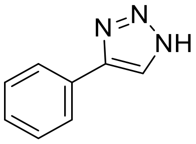

Prepared according to *Method A*: Phenylacetylene (**1a**) (104 mg, 1.02 mmol, 1 equiv.), $CuSO_4{\times}5H_2O$ (51 mg, 0.20 mmol, 20 mol%), Na(asc) (200 mg, 1.01 mmol, 1 equiv.), NaN_3 (104 mg, 1.60 mmol, 1.5 equiv.), 2.5 mL of solvent mixture, 96% H_2SO_4 (89 μL, 1.6 mmol, 1.6 equiv.). Yellowish-white crystalline solid, 113 mg (76%).

Prepared according to *Method A (with CuI catalyst and DMF/MeOH solvent)*: Phenylacetylene (**1a**) (49 mg, 0.50 mmol, 1 equiv.), CuI (19 mg, 0.10 mmol, 20 mol%), Na(asc) (100 mg, 0.50 mmol, 1 equiv.), NaN_3 (51 mg, 0.78 mmol, 1.5 equiv.), 1.25 mL of solvent mixture, 96% H_2SO_4 (45 μL, 79 mg, 0.81 mmol, 1.6 equiv.). White crystalline solid, 61 mg (84%).

Prepared according to *Method B*: Phenylacetylene (**1a**) (47 mg, 0.46 mmol, 1 equiv.), CuI (4.8 mg, 0.025 mmol, 5 mol%), Na(asc) (20 mg, 0.1 mmol, 0.2 equiv.), TBTA (6.6 mg, 0.0125 mmol, 2.5 mol%), NaN_3 (47 mg, 0.72 mmol, 1.5 equiv.), 0.6 mL DMF, HCOOH (94 μL, 2.5 mmol, 5 equiv.). White crystalline solid, 59 mg (89%)

Prepared according to *Method C*: Phenylacetylene (**1a**) (52 mg, 0.51 mmol, 1 equiv.), $CuSO_4{\times}5H_2O$ (6.3 mg, 0.025 mmol, 5 mol%), Na(asc) (25 mg, 0.126 mmol, 0.25 equiv), $(BimH)_3$ (10.2 mg, 0.025 mmol, 5 mol%), NaN_3 (52 mg, 0.80 mmol, 1.5 equiv.), 0.6 mL of solvent mixture, CH_3COOH (190 μL, 3.3 mmol, 6.6 equiv.). White crystalline solid, 73 mg (99%).

Mp.: 144–146 °C. Mp. (lit.): 145–146 °C.[10]

IR (cm^{-1}): 3152, 3116, 2956, 2850, 1454, 1082, 971, 873, 763, 691.

^{1}H NMR (500 MHz, $CDCl_3$) δ 7.99 (s, 1H), 7.85–7.81 (m, 2H), 7.49–7.44 (m, 2H), 7.41–7.37 (m, 1H).

Prepared according to *Method C with extraction and recrystallization:* Phenylacetylene (**1a**) (54 mg, 0.53 mmol, 1 equiv.), $CuSO_4{\times}5H_2O$ (6.3 mg, 0.025 mmol, 5 mol%), Na(asc) (25 mg, 0.126 mmol, 0.25 equiv), $(BimH)_3$ (10.2 mg, 0.025 mmol, 5 mol%), NaN_3 (52 mg, 0.80 mmol, 1.5 equiv.), 0.6 mL of solvent mixture, CH_3COOH (190 μL, 3.3 mmol, 6.6 equiv.). After 24 hours product was extracted with

ethyl acetate from ammonium acetate solution (20 mL sat. NH_4Cl + 20 mL H_2O). Ethyl acetate was dried over anhydrous Na_2SO_4 and evaporated.

^{1}H NMR spectrum of crude product with Method C after extraction:

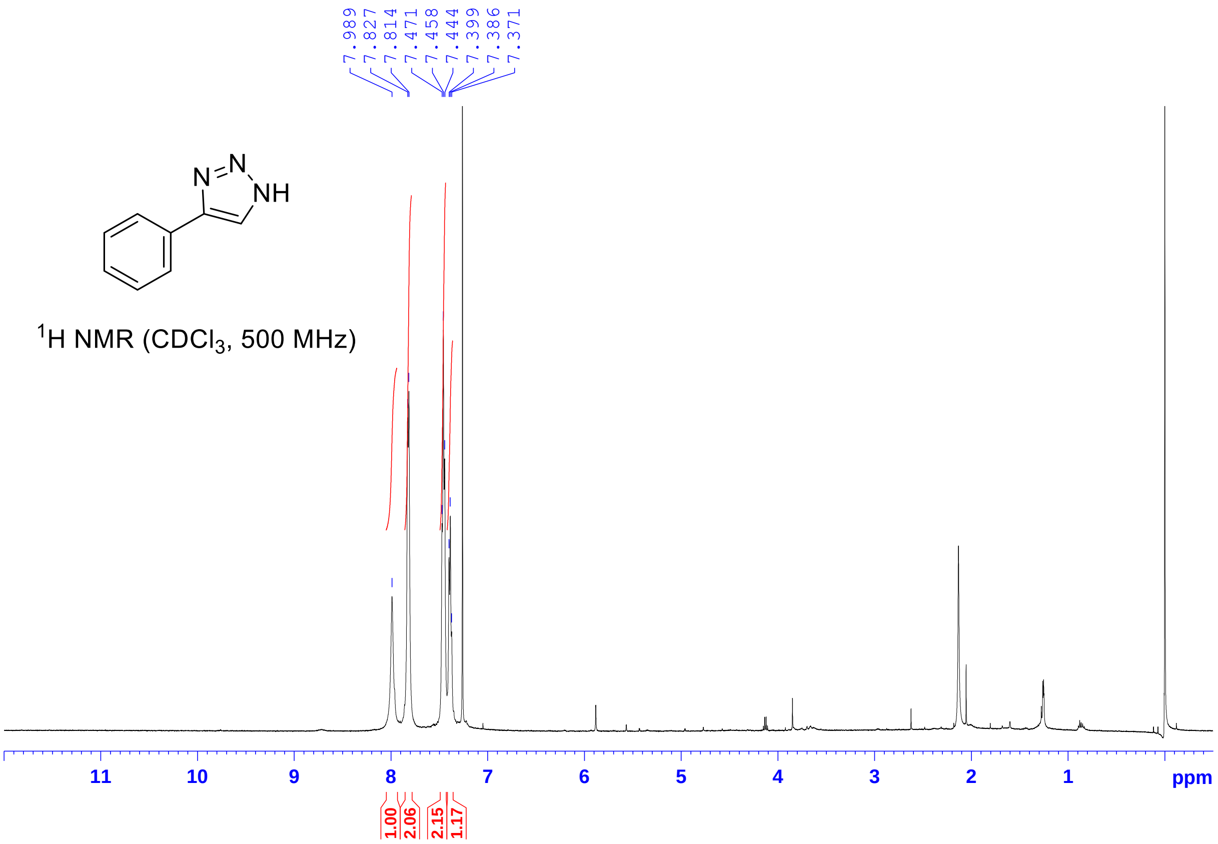

Supporting Figure S2. Crude product **3a** after extraction using Method C conditions.

In the crude product **3a** was almost already in pure form (see Supp. Fig. S2) and could be easily further purified by crystallization. For example, crude product was recrystallized by using ethyl acetate/petroleum ether mixture to obtain product **3a** in completely pure form (43 mg, 56%). Crystallization protocol was not optimized.

4-(4-(Trifluoromethyl)phenyl)-1*H*-1,2,3-triazole (3b, Fig. 3)

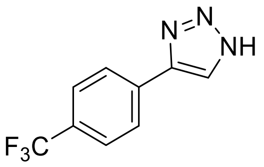

Prepared according to *Method A*: 1-ethynyl-4-(trifluoromethyl)benzene (**1b**) (174 mg, 1.02 mmol, 1 equiv.), $CuSO_4 \times 5H_2O$ (52 mg, 0.21 mmol, 20 mol%), Na(asc) (205 mg, 1.03 mmol, 1 equiv.), NaN_3 (103 mg, 1.58 mmol, 1.5 equiv.), 2.5 mL of solvent mixture, 96% H_2SO_4 (89 µL, 1.6 mmol, 1.6 equiv.). Yellowish white crystalline solid, 145 mg (67%).

Mp.: 188.5–189.5 °C.

IR (cm^{-1}): 3141, 2967, 2866, 1424, 1320, 1169, 1130, 1063, 973, 840.

^{1}H NMR (500 MHz, $CDCl_3$) δ 11.83 (br, 1H), 8.04 (s, 1H), 7.98–7.93 (m, 2H), 7.74–7.70 (m, 2H). NMR data are in agreement with those from the literature.[11]

4-(4-Methylphenyl)-1*H*-1,2,3-triazole (3c, Fig. 3)

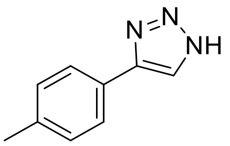

Prepared according to *Method A*: 1-ethynyl-4-methylbenzene (**1c**) (117 mg, 1.01 mmol, 1 equiv.) $CuSO_4 \times 5H_2O$ (51 mg, 0.20 mmol, 20 mol%), Na(asc) (200 mg, 1.01 mmol, 1 equiv.), NaN_3 (99 mg, 1.52 mmol, 1.5 equiv.), 2.5 mL of solvent mixture, 96% H_2SO_4 (89 μL, 1.6 mmol, 1.6 equiv.). Off-white crystalline solid, 100 mg (62%).

Prepared according to *Method A (with CuI catalyst and DMF/MeOH solvent)*: 1-ethynyl-4-methylbenzene (**1c**) (58 mg, 0.50 mmol, 1 equiv.) CuI (19 mg, 0.10 mmol, 20 mol%), Na(asc) (99 mg, 0.50 mmol, 1 equiv.), NaN_3 (51 mg, 0.78 mmol, 1.5 equiv.), 1.25 mL of solvent mixture, 96% H_2SO_4 (45 μL, 0.81 mmol, 1.6 equiv.). White crystalline solid, 62 mg (78%).

Mp.: 152–154.5 °C. Mp. (lit.): 150–152 °C.[10]

IR (cm^{-1}): 3155, 3123, 2858, 1477, 1075, 999, 972, 874, 820, 723.

^{1}H NMR (500 MHz, $CDCl_3$) δ 11.67 (br, 1H), 7.96 (s, 1H), 7.74–7.70 (m, 2H), 7.27 (d, J = 7.4 Hz, 2H), 2.40 (s, 3H).

4-(4-Methoxyphenyl)- 1*H*-1,2,3-triazole (3d, Fig. 3)

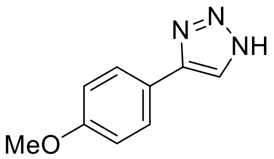

Prepared according to *Method A*: 1-ethynyl-4-methoxybenzene (**1d**) (133 mg, 1.01 mmol, 1 equiv.), $CuSO_4 \times 5H_2O$ (51 mg, 0.20 mmol, 20 mol%), Na(asc) (200 mg, 1.01 mmol, 1 equiv.), NaN_3 (99 mg, 1.52 mmol, 1.5 equiv.), 2.5 mL of solvent mixture, 96% H_2SO_4 (89 μL, 1.6 mmol, 1.6 equiv.). Off-white crystalline solid, 98 mg (56%).

Prepared according to *Method A (with CuI catalyst and DMF/MeOH solvent)*: 1-ethynyl-4-methoxybenzene (**1d**) (74 mg, 0.56 mmol, 1 equiv.), CuI (19 mg, 0.10 mmol, 20 mol%), Na(asc) (98 mg, 0.49 mmol, 1 equiv.), NaN_3 (53 mg, 0.82 mmol, 1.5 equiv.), 1.25 mL of solvent mixture, 96% H_2SO_4 (45 μL, 0.81 mmol, 1.6 equiv.). Brownish white crystalline solid, 74 mg (75%).

Prepared according to *Method B*: 1-ethynyl-4-methoxybenzene (**1d**) (66 mg, 0.50 mmol, 1 equiv.), CuI (4.8 mg, 0.025 mmol, 5 mol%), Na(asc) (20 mg, 0.1 mmol, 0.2 equiv.), TBTA (6.6 mg, 0.0125 mmol, 2.5 mol%), NaN_3 (51 mg, 0.78 mmol, 1.5 equiv.), 0.6 mL DMF, HCOOH (115 mg, 94 μL, 2.5 mmol, 5 equiv.). White crystalline solid, 61 mg (70%)

Prepared according to *Method C*: 1-ethynyl-4-methoxybenzene (**1d**) (65 mg, 0.49 mmol, 1 equiv.), $CuSO_4 \times 5H_2O$ (6.3 mg, 0.025 mmol, 5 mol%), Na(asc) (25 mg, 0.126 mmol, 0.25 equiv.), $(BimH)_3$ (10.2 mg, 0.025 mmol, 5 mol%), NaN_3 (49 mg, 0.75 mmol, 1.5 equiv.), 0.6 mL of solvent mixture, CH_3COOH (190 μL, 3.3 mmol, 6.6 equiv.). White crystalline solid, 82 mg (95%).

Mp.: 167–168.5 °C. Mp. (lit.): 168 –169 °C.[12]

IR (cm^{-1}): 3153, 3114, 2835, 1613, 1533, 1465, 1247, 972, 873, 827.

^1H NMR (500 MHz, $CDCl_3$) δ 7.90 (s, 1H), 7.77–7.73 (m, 2H), 7.01–6.96 (m, 2H), 3.86 (s, 3H).

4-(4-Cianophenyl)-1*H*-1,2,3-triazole (3e, Fig. 3)

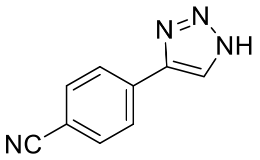

Prepared according to *Method A*: 1-ethynyl-4-cianobenzene (**1e**) (120 mg, 0.94 mmol, 1 equiv.), $CuSO_4 \times 5H_2O$ (51 mg, 0.20 mmol, 20 mol%), Na(asc) (200 mg, 1.01 mmol, 1 equiv.), NaN_3 (97 mg, 1.49 mmol, 1.5 equiv.), 2.5 mL of solvent mixture, 96% H_2SO_4 (89 μL, 1.6 mmol, 1.6 equiv.). Off-white crystalline solid, 115 mg (72%).

Mp.: 172.8–174.5 °C. Mp. (lit.): 170 –172 °C.[10]

IR (cm^{-1}): 3125, 2857, 2227, 1612, 1131, 1079, 997, 969, 869, 850.

^1H NMR (500 MHz, $CDCl_3$) δ 11.96 (br, 1H), 8.04 (s, 1H), 7.97–7.94 (m, 2H), 7.77–7.73 (m, 2H).

4-(4-Bromophenyl)-1*H*-1,2,3-triazole (3f, Fig. 3)

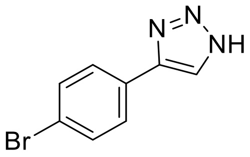

Prepared according to *Method A*: 4-bromo-1-ethynylbenzene (**1f**) (53 mg, 0.29 mmol, 1 equiv.), $CuSO_4 \times 5H_2O$ (15 mg, 0.06 mmol, 20 mol%), Na(asc) (60 mg, 0.30 mmol, 1 equiv.), NaN_3 (30 mg, 0.46 mmol, 1.5 equiv.), 1 mL of solvent mixture, 96% H_2SO_4 (27 µL, 0.48 mmol, 1.6 equiv.). Off-white crystalline solid, 43 mg (65%).

Mp.: 170.2–173 °C. Mp. (lit.): 172–173 °C.[13]

IR (cm^{-1}): 3149, 3120, 2837, 1131, 1068, 1001, 969, 874, 830, 722.

^{1}H NMR (500 MHz, $CDCl_3$) δ 7.96 (s, 1H), 7.72–7.68 (m, 2H), 7.61–7.57 (m, 2H).

Prepared according to *Method B*: 4-bromo-1-ethynylbenzene (**1f**) (91 mg, 0.50 mmol, 1 equiv.), CuI (4.8 mg, 0.025 mmol, 5 mol%), Na(asc) (20 mg, 0.1 mmol, 0.2 equiv.), TBTA (6.6 mg, 0.0125 mmol, 2.5 mol%), NaN_3 (51 mg, 0.78 mmol, 1.5 equiv.), 0.6 mL DMF, HCOOH (94 µL, 2.5 mmol, 5 equiv.). White crystalline solid, 83 mg (74%)

4-(4-Nitrophenyl)-1*H*-1,2,3-triazole (3g, Fig. 3)

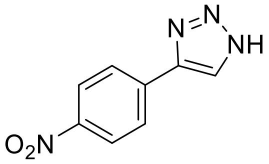

Prepared according to *Method A*: 1-ethynyl-4-nitrobenzene (**1g**) (143 mg, 0.97 mmol, 1 equiv.), $CuSO_4 \times 5H_2O$ (51 mg, 0.20 mmol, 20 mol%), Na(asc) (203 mg, 1.03 mmol, 1 equiv.), NaN_3 (101 mg, 1.55 mmol, 1.5 equiv.), 2.5 mL of solvent mixture, 96% H_2SO_4 (89 µL, 1.6 mmol, 1.6 equiv.). Brownish white crystalline solid, 103 mg (56%).

Prepared according to *Method B*: 1-ethynyl-4-nitrobenzene (**1g**) (78 mg, 0.53 mmol, 1 equiv.), CuI (4.8 mg, 0.025 mmol, 5 mol%), Na(asc) (20 mg, 0.1 mmol, 0.2 equiv.), TBTA (6.6 mg, 0.0125 mmol, 2.5 mol%), NaN_3 (54 mg, 0.83 mmol, 1.5 equiv.), 0.6 mL DMF, HCOOH (94 µL, 2.5 mmol, 5 equiv.). White crystalline solid, 86 mg (85%)

Prepared according to *Method C*: 1-ethynyl-4-nitrobenzene (**1g**) (74 mg, 0.50 mmol, 1 equiv.), $CuSO_4 \times 5H_2O$ (6.3 mg, 0.025 mmol, 5 mol%), Na(asc) (25 mg, 0.126 mmol, 0.25 equiv.), $(BimH)_3$ (10.2

mg, 0.025 mmol, 5 mol%), NaN_3 (50 mg, 0.77 mmol, 1.5 equiv.), 0.6 mL of solvent mixture, CH_3COOH (190 µL, 3.3 mmol, 6.6 equiv.). Brownish white crystalline solid, 65 mg (68%).

Mp.: 199.5–201 °C. Mp. (lit.): 198–199 °C.[14]

IR (cm^{-1}): 3104, 2896, 1603, 1509, 1333, 1107, 854, 819, 755, 711.

^1H NMR (500 MHz, DMSO-d_6) δ 8.63 (br, 1H), 8.34–8.30 (m, 2H), 8.18–8.12 (m, 2H).

4-(2-Fluorophenyl)-1*H*-1,2,3-triazole (3h, Fig. 3)

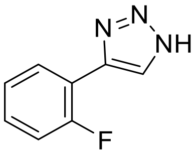

Prepared according to *Method A*: 1-ethynyl-2-fluorobenzene (**1h**) (123 mg, 1.02 mmol, 1 equiv.), $CuSO_4 \times 5H_2O$ (50 mg, 0.20 mmol, 20 mol%), Na(asc) (200 mg, 1.01 mmol, 1 equiv.), NaN_3 (98 mg, 1.51 mmol, 1.5 equiv.), 2.5 mL of solvent mixture, 96% H_2SO_4 (89 µL, 1.6 mmol, 1.6 equiv.). Brownish white crystalline solid, 103 mg (62%).

Mp.: 83.5–85.5 °C.

IR (cm^{-1}): 3126, 2855, 1471, 1217, 1077, 1001, 975, 860, 817, 753.

^1H NMR (500 MHz, $CDCl_3$) δ 8.14 (d, J = 3.5 Hz, 1H), 8.10–8.06 (m, 1H), 7.39–7.34 (m, 1H), 7.29–7.24 (m, 1H), 7.22–7.16 (m, 1H). NMR data are in agreement with those from the literature.[11]

4-(3-Ethoxy-4-Methoxyphenyl)-1*H*-1,2,3-triazole (3i, Fig. 3)

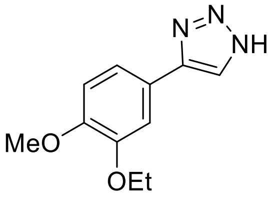

Prepared according to *Method A*: 1-ethynyl-3-ethoxy-4-methoxybenzene (**1i**) (86 mg, 0.49 mmol, 1 equiv.), $CuSO_4 \times 5H_2O$ (26 mg, 0.10 mmol, 20 mol%), Na(asc) (101 mg, 0.51 mmol, 1 equiv.), NaN_3 (50 mg, 0.77mmol, 1.5 equiv.), 1.25 mL of solvent mixture, 96% H_2SO_4 (45 µL, 0.81 mmol, 1.6 equiv.). Off-white crystalline solid, 57 mg (53%).

Mp.: 133–134.5 °C.

IR (cm^{-1}): 3131, 2836, 1493, 1348, 1251, 1234, 1142, 993, 852, 826.

^{1}H NMR (500 MHz, $CDCl_3$) δ 7.91 (s, 1H), 7.40 (d, *J* = 2.1 Hz, 1H), 7.33 (dd, *J* = 8.3, 2.1 Hz, 1H), 6.94 (d, *J* = 8.3 Hz, 1H), 4.19 (q, *J* = 7.0 Hz, 2H), 3.92 (s, 3H), 1.49 (t, *J* = 7.0 Hz, 3H).

$^{13}C\{^{1}H\}$ NMR (126 MHz, $CDCl_3$) δ 150, 148.9, 147.3, 129.4, 122.8, 118.9, 111.8, 110.6, 64.7, 56.2, 15.

HRMS–ESI (*m/z*): $[M + H]^+$ calcd for $C_{11}H_{14}N_3O_2^+$, 220.1081; found, 220.1081.

2-(1*H*-1,2,3-Triazol-4-yl)pyridine (3k, Fig. 3)

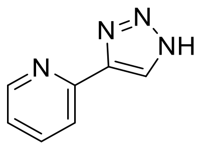

Prepared according to *Method A*: 2-ethynylpyridine (**1k**) (111 mg, 1.08 mmol, 1 equiv.), $CuSO_4 \times 5H_2O$ (50 mg, 0.20 mmol, 20 mol%), Na(asc) (201 mg, 1.01 mmol, 1 equiv.), NaN_3 (100 mg, 1.54 mmol, 1.5 equiv.), 2.5 mL of solvent mixture, 96% H_2SO_4 (89 µL, 1.6 mmol, 1.6 equiv.). Column chromatography with mobile phase CH_2Cl_2:acetone; 1:1. Brown crystalline solid, 89 mg (57%).

Mp.: 153.5–155.4 °C.

IR (cm^{-1}): 3118, 2724, 1703, 1601, 1510, 1005, 960, 890, 781, 740.

^{1}H NMR (500 MHz, $CDCl_3$) δ 8.71 (d, *J* =4.8 Hz, 1H), 8.33 (s, 1H), 8.02–7.97 (m, 1H), 7.85–7.79 (m, 1H), 7.33–7.28 (m, 1H). NMR data are in agreement with those from the literature.[15]

4-(Tiophen-2-yl)-1*H*-1,2,3-triazole (3l, Fig. 3)

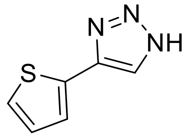

Prepared according to *Method A*: 2-ethynyltiophene (**1l**) (113 mg, 1.04 mmol, 1 equiv.), $CuSO_4 \times 5H_2O$ (51 mg, 0.20 mmol, 20 mol%), Na(asc) (202 mg, 1.02 mmol, 1 equiv.), NaN_3 (106 mg, 1.63 mmol, 1.5 equiv.), 2.5 mL of solvent mixture, 96% H_2SO_4 (89 µL, 1.6 mmol, 1.6 equiv.). Brown crystalline solid, 67 mg (43%).

Mp.: 90.5–92.5 °C.

IR (cm^{-1}): 3117, 2973, 2843, 1416, 1123, 1033, 997, 934, 847, 692.

^{1}H NMR (500 MHz, $CDCl_3$) δ 7.92 (s, 1H), 7.43 (dd, *J* = 3.6, 1.1 Hz, 1H), 7.34 (dd, *J* = 5.2, 1.1 Hz, 1H), 7.10 (dd, *J* = 5.2, 3.6 Hz, 1H).

$^{13}C\{^{1}H\}$ NMR (126 MHz, $CDCl_3$) δ 142.6, 132.1, 129.1, 128, 126.1, 125.4.

HRMS–ESI (*m/z*): $[M + H]^+$ calcd for $C_6H_6N_3S^+$, 152.0277; found, 152.028.

4-((Phenylthio)methyl)-1*H*-1,2,3-triazole (3m, Fig. 3)

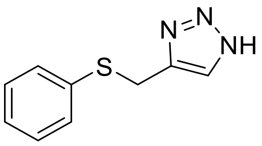

Prepared according to *Method A*: Phenyl propargyl sulfide (**1m**) (142 mg, 0.96 mmol, 1 equiv.). $CuSO_4$×$5H_2O$ (50 mg, 0.20 mmol, 20 mol%), Na(asc) (200 mg, 1.01 mmol, 1 equiv.), NaN_3 (99 mg, 1.52 mmol, 1.5 equiv.), 2.5 mL of solvent mixture, 96% H_2SO_4 (89 μL, 1.6 mmol, 1.6 equiv.). Brown crystalline solid, 95 mg (52%).

Mp.: 74.7–76.2 °C.

IR (cm^{-1}): 3150, 3115, 2841, 1478, 1435, 1223, 1023, 1001, 728, 688.

^{1}H NMR (500 MHz, $CDCl_3$) δ 7.55 (s, 1H), 7.36–7.31 (m, 2H), 7.28–7.23 (m, 2H), 7.22–7.17 (m, 1H), 4.23 (s, 2H).

$^{13}C\{^{1}H\}$ NMR (126 MHz, $CDCl_3$) δ 144.3, 135.1, 132.9, 131.1, 130.3, 129.2, 127.0, 126.5, 28.9.

HRMS–ESI (*m/z*): $[M + H]^+$ calcd for $C_9H_{10}N_3S^+$, 192.0590; found, 192.0595.

4-(Triisopropylsilyl)-1*H*-1,2,3-triazole (3n, Fig. 3)

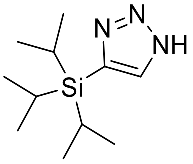

Prepared according to *Method A*: (Triisopropylsilyl)acetylene (**1n**) (181 mg, 0.99 mmol, 1 equiv.), $CuSO_4$×$5H_2O$ (50 mg, 0.20 mmol, 20 mol%), Na(asc) (201 mg, 1.01 mmol, 1 equiv.), NaN_3 (99 mg, 1.52 mmol, 1.5 equiv.), 2.5 mL of solvent mixture, 96% H_2SO_4 (89 μL, 1.6 mmol, 1.6 equiv.). White crystalline solid, 140 mg (63%).

Mp.: 103.8–105.5 °C.

IR (cm^{-1}): 3098, 2940, 2863, 1460, 1179, 1105, 1017, 881, 679, 655.

^{1}H NMR (500 MHz, $CDCl_3$) δ 12.55 (br, 1H), 7.83 (s, 1H), 1.38 (sept, *J* = 7.4 Hz, 3H), 1.10 (d, *J* = 7.5 Hz, 18H).

$^{13}C\{^{1}H\}$ NMR (126 MHz, $CDCl_3$) δ 139.9, 132.9, 18.6, 11.2.

HRMS–ESI (*m/z*): $[M + H]^+$ calcd for $C_{11}H_{24}N_3Si^+$, 226.1734; found, 226.1734.

4-Dodecyl-1*H*-1,2,3-triazole (3o, Fig. 3)

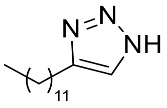

Prepared according to *Method A*: 1-tetradecyne (**1o**) (197 mg, 1.01 mmol, 1 equiv.), $CuSO_4{\times}5H_2O$ (50 mg, 0.20 mmol, 20 mol%), Na(asc) (200 mg, 1.01 mmol, 1 equiv.), NaN_3 (99 mg, 1.52 mmol, 1.5 equiv.), 2.5 mL of solvent mixture, 96% H_2SO_4 (89 µL, 1.6 mmol, 1.6 equiv.). Extraction with CH_2Cl_2. White crystalline product, 96 mg (40%).

Prepared according to *Method A (with CuI catalyst and DMF/MeOH solvent)*: 1-tetradecyne (**1o**) (99 mg, 0.51 mmol, 1 equiv.), CuI (19 mg, 0.10 mmol, 20 mol%), Na(asc) (98 mg, 0.49 mmol, 1 equiv.), NaN_3 (51 mg, 0.78 mmol, 1.5 equiv.), 1.25 mL of solvent mixture, 96% H_2SO_4 (45 µL, 0.81 mmol, 1.6 equiv.). White crystalline solid, 76 mg (63%).

Mp.: 64–67 °C.

IR (cm^{-1}): 3159, 3113, 2913, 2848, 1470, 1022, 999, 875, 844, 718.

^{1}H NMR (500 MHz, $CDCl_3$) δ 7.51 (s, 1H), 2.76–2.71 (m, 2H), 1.72–1.64 (m, 2H), 1.40–1.19 (m, 18H), 0.87 (t, *J* = 6.9 Hz, 3H).

$^{13}C\{^{1}H\}$ NMR (126 MHz, $CDCl_3$) δ 147.2, 131.5, 32.1, 29.84, 29.82, 29.7, 29.54, 29.52, 29.39, 29.35, 25.2, 22.9, 14.3.

HRMS–ESI (*m/z*): $[M + H]^+$ calcd for $C_{14}H_{28}N_3^+$, 238.2278; found, 238.2275.

4-(Cyclohex-1-ene-1-yl)-1*H*-1,2,3-triazole (3p, Fig. 3)

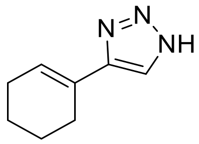

Prepared according to *Method A*: 1-ethynylcyclohex-1-ene (**1p**) (107 mg, 1.01 mmol, 1 equiv.), $CuSO_4{\times}5H_2O$ (51 mg, 0.20 mmol, 20 mol%), Na(asc) (201 mg, 1.01 mmol, 1 equiv.), NaN_3 (98 mg, 1.50 mmol, 1.5 equiv.), 2.5 mL of solvent mixture, 96% H_2SO_4 (89 µL, 1.6 mmol, 1.6 equiv.). Brownish white crystalline solid, 103 mg (69%).

Prepared according to *Method B*: 1-ethynylcyclohex-1-ene (**1p**) (53 mg, 0.50 mmol, 1 equiv.), CuI (4.8 mg, 0.025 mmol, 5 mol%), Na(asc) (20 mg, 0.1 mmol, 0.2 equiv.), TBTA (6.6 mg, 0.0125 mmol, 2.5 mol%), NaN_3 (50 mg, 0.77 mmol, 1.5 equiv.), 0.6 mL DMF, HCOOH (94 µL, 2.5 mmol, 5 equiv.). White crystalline solid, 43 mg (57%).

Mp.: 90–93 °C.

IR (cm^{-1}): 3139, 2933, 2860, 1651, 1040, 993, 982, 917, 864, 845.

^1H NMR (500 MHz, $CDCl_3$) δ 7.68 (s, 1H), 6.43–6.39 (m, 1H), 2.46–2.41 (m, 2H), 2.24–2.19 (m, 2H), 1.81–1.75 (m, 2H), 1.71–1.65 (m, 2H). NMR data are in agreement with those from the literature.[12]

4-(*Tert*-butyl)-1*H*-1,2,3-triazole (3q, Fig. 3)

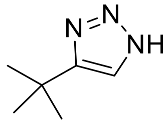

Prepared according to *Method A*: 3,3-dimethylbut-1-yn (**1q**) (82 mg, 1.00 mmol, 1 equiv.), $CuSO_4{\times}5H_2O$ (51 mg, 0.20 mmol, 20 mol%), Na(asc) (200 mg, 1.01 mmol, 1 equiv.), NaN_3 (101 mg, 1.55 mmol, 1.5 equiv.), 2.5 mL of solvent mixture, 96% H_2SO_4 (89 µL, 1.6 mmol, 1.6 equiv.). No extraction. Crude reaction mixture was concentrated under reduced pressure and purified with silica gel column chromatography. Yellowish oil, 63 mg (50%).

IR (cm^{-1}): 3136, 2962, 2904, 2870, 1707, 1462, 1366, 1206, 1111, 975, 851.

^1H NMR (500 MHz, $CDCl_3$) δ 7.55 (s, 1H), 1.37 (s, 9H).

$^{13}C\{^1H\}$ NMR (126 MHz, $CDCl_3$) δ 155.6, 128.9, 30.7, 30.5.

HRMS–ESI (*m/z*): $[M + H]^+$ calcd for $C_6H_{12}N_3^+$, 126.1026; found, 126.1029.

4-Propyl-1*H*-1,2,3-triazole (3r, Fig. 3)

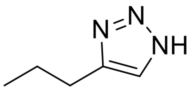

Prepared according to *Method A*: 1-pentyne (**1r**) (68 mg, 1.00 mmol, 1 equiv.), $CuSO_4 \times 5H_2O$ (52 mg, 0.21 mmol, 20 mol%), Na(asc) (200 mg, 1.01 mmol, 1 equiv.), NaN_3 (100 mg, 1.54 mmol, 1.5 equiv.), 2.5 mL of solvent mixture, 96% H_2SO_4 (89 µL, 1.6 mmol, 1.6 equiv.). Yellowish oil, 36 mg, (32%).

Prepared according to *Method A (with CuI catalyst and DMF/MeOH solvent)*: 1-pentyne (**1r**) (34 mg, 0.50 mmol, 1 equiv.), CuI (19 mg, 0.10 mmol, 20 mol%), Na(asc) (100 mg, 0.50 mmol, 1 equiv.), NaN_3 (49 mg, 0.75 mmol, 1.5 equiv.), 1.25 mL of solvent mixture, 96% H_2SO_4 (45 µL, 0.81 mmol, 1.6 equiv.). Yellowish oil, 29 mg (52%).

Prepared according to *Method C*: 1-pentyne (**1r**) (34 mg, 0.50 mmol, 1 equiv.), $CuSO_4 \times 5H_2O$ (6.3 mg, 0.025 mmol, 5 mol%), Na(asc) (25 mg, 0.126 mmol, 0.25 equiv.), $(BimH)_3$ (10.2 mg, 0.025 mmol, 5 mol%), NaN_3 (50 mg, 0.77 mmol, 1.5 equiv.), 0.6 mL of solvent mixture, CH_3COOH (190 µL, 3.3 mmol, 6.6 equiv.). Yellowish oil, 30 mg (53%).

IR (cm^{-1}): 3135, 2961, 2933, 2873, 1706, 1460, 1200, 1110, 973, 801.

^{1}H NMR (300 MHz, $CDCl_3$) δ 9.22 (br, 1H), 7.59 (s, 1H), 2.73 (t, *J* = 7.6 Hz, 2H), 1.79–1.65 (m, 2H), 0.98 (t, *J* = 7.4 Hz, 3H).

$^{13}C\{^1H\}$ NMR (126 MHz, $CDCl_3$) δ 146.2, 130.9, 27.0, 22.6, 13.9.

HRMS–ESI (*m/z*): $[M + H]^+$ calcd for $C_5H_{10}N_3^+$, 112.0869; found, 112.0870.

Androst-4-en-3-one-17*α*-hydroxy-17*β*-(1*H*-1,2,3-triazol-4-yl) (3s, Fig. 3)

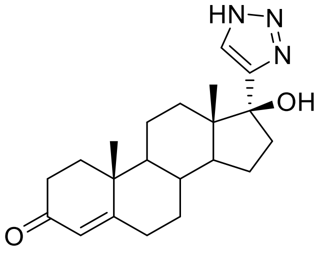

Prepared according to slightly modified *Method B*, reaction time: 120 h at 60 °C in aluminum heating block (due to low solubility of ethisterone **1s**). Ethisterone (**1s**) (156 mg, 0.50 mmol, 1 equiv.), CuI (4.8 mg, 0.025 mmol, 5 mol%), Na(asc) (20 mg, 0.1 mmol, 0.2 equiv.), TBTA (6.6 mg, 0.0125 mmol, 2.5 mol%), NaN_3 (49 mg, 0.75 mmol, 1.5 equiv.), 2 mL DMF, HCOOH (94 µL, 2.5 mmol, 5 equiv.). Product was purified with silica column chromatography using Interchim puriFlash® XS520 (Column: Interchim

PF-25SIHC-F0040 (40 g, 25 µm SiO_2), gradient elution (petroleum ether–ethyl acetate): 00:00–6:30: 82% petroleum ether → 74% petroleum ether; 6:30–21:00: 74% petroleum ether → 0% petroleum ether; 21:00–35:00: 100% ethyl acetate, flow rate: 26 mL/min). Yellow crystalline solid, 114 mg (64%).

Mp.: 233–236 °C.

IR (cm^{-1}): 3125, 2945, 2904, 22848, 1659, 1607, 1232, 988, 871, 857.

^{1}H NMR (500 MHz $CDCl_3$) δ 7.55 (s, 1H), 5.72 (s, 1H), 2.44–2.22 (m, 5H), 2.18–2.10 (m, 1H), 1.95–1.80 (m, 3H), 1.66–1.50 (m, 4H), 1.45–1.23 (m, 4H), 1.15 (s, 3H), 1.06 (s, 3H), 1.06–0.95 (m, 2H), 0.72–0.65 (m, 1H), 0.45–0.37 (m, 1H).

^{13}C{^{1}H} NMR (126 MHz $CDCl_3$) δ 199.9, 171.4, 124.1, 82.5, 53.4, 49.1, 47.1, 38.7, 38.3, 36.4, 35.7, 34.1, 33.0, 32.8, 31.8, 23.9, 20.7, 17.6, 14.3.

HRMS–ESI (*m/z*): $[M + H]^+$ calcd for $C_{21}H_{30}N_3O_2^+$, 356.2333; found, 356.2331.

Androsta-4,16-dien-3-one-17-(1*H*-1,2,3-triazol-4-yl) (3t, Fig. 3)

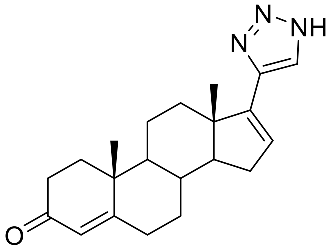

Prepared according to slightly modified *Method C*: Pregna-4,16-dien-20-yn-3-one (**1t**) (147 mg, 0.50 mmol, 1 equiv.), $CuSO_4 \times 5H_2O$ (6.3 mg, 0.025 mmol, 5 mol%), Na(asc) (25 mg, 0.126 mmol, 0.25 equiv.), $(BimH)_3$ (10.2 mg, 0.025 mmol, 5 mol%), NaN_3 (49 mg, 0.75 mmol, 1.5 equiv.), 1.2 mL MeOH, CH_3COOH (190 µL, 3.3 mmol, 6.6 equiv.). White crystalline solid, 95 mg (56%). Product was purified with silica column chromatography using Interchim puriFlash® XS520 (Column: Interchim PF-25SIHC-F0040 (40 g, 25 µm SiO_2), gradient elution (petroleum ether–ethyl acetate): 00:00–6:30: 82% petroleum ether → 74% petroleum ether; 6:30–21:00: 74% petroleum ether → 0% petroleum ether; 21:00–35:00: 100% ethyl acetate, flow rate: 26 mL/min). White crystalline solid, 95 mg (56%).

Mp.: 235–238 °C.

IR (cm^{-1}): 3158, 2927, 2859, 1650, 1611, 1234, 957, 872, 841, 780.

^{1}H NMR (500 MHz, $CDCl_3$) δ 7.71 (s, 1H), 6.21–6.19 (m, 1H), 5.76 (s, 1H), 2.50–1.00 (m, 17H), 1.24 (s, 3H), 1.04 (s, 3H).

$^{13}C\{^1H\}$ NMR (126 MHz, $CDCl_3$) δ 199.9, 171.5, 143.6, 129.2, 124.2, 56.4, 54.3, 47.2, 38.9, 35.7, 35.2, 34.2, 34.1, 33.0, 32.0, 31.9, 21.1, 17.4, 16.4.

HRMS–ESI (*m/z*): $[M + H]^+$ calcd for $C_{21}H_{28}N_3O^+$, 338.2227; found, 338.2224.

(1-((2'-(1*H*-1,2,3-triazol-4-yl)-[1,1'-biphenyl]-4-yl)methyl)-2-butyl-4-chloro-1H-imidazol-5-yl)methanol – Losartan analogue (3u, Fig. 3)

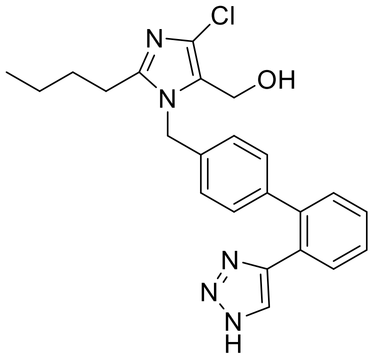

Prepared according to slightly modified *Method C*. Into a 20 mL ACE pressure tube **1u** (190 mg, 0.50 mmol, 1 equiv.), $CuSO_4 \times 5H_2O$ (25 mg, 0.1 mmol, 20 mol%), $(BimH)_3$ (40 mg, 0.1 mmol, 20 mol%), sodium ascorbate (50 mg, 0.25 mmol, 0.5 equiv.), sodium azide (65 mg, 1 mmol, 2 equiv.), 1 mL of MeOH and CH_3COOH (190 µL, 3.3 mmol, 6.6 equiv.) were added in that order. After addition of acetic acid ACE pressure tube was sealed with a screw cap and reaction was left to stir for 72 hours at 40 °C in aluminum heating block. After 72 hours the reaction tube was ucapped and ethyl acetate (30 mL) and NH_4Cl solution (20 mL sat. NH_4Cl, 20 mL H_2O) were added to the reaction mixture. The phases were separated in separatory funnel and aqueous layer was additionally extracted with EtOAc (3 × 30 mL). Organic phases were combined and dried over anhydrous sodium sulfate, filtered and solvent was removed under reduced pressure by the aid of rotary evaporator. Crude product was purified by column chromatography using Interchim puriFlash® XS520 (Column: Interchim PF-25SIHC-F0040 (40 g, 25 µm SiO_2), gradient elution: petroleum ether → ethyl acetate; 00:00–06:00; 88% PE → 82% PE, 06:00–27:00; 82% PE → 7% PE, 27:00–38:00; 7% PE, flow rate: 26 mL/min.) to obtain white crystalline solid, 172 mg (82%).

Mp.: 95–97 °C.

IR (cm^{-1}): 3138, 2955, 2929, 2869, 1575, 1464, 1255, 1005, 965, 765.

^1H NMR (500 MHz, $CDCl_3$) δ 7.82 (br, 1H), 7.48–7.39 (m, 2H), 7.36–7.30 (m, 2H), 7.16 (d, *J* = 8.1 Hz, 2H), 6.97 (d, *J* = 8.1 Hz, 2H), 6.95 (br, 1H), 5.23 (s, 2H), 4.52 (s, 2H), 2.60–2.52 (m, 2H), 1.66–1.57 (m, 2H), 1.35–1.27 (m, 2H), 0.84 (t, *J* = 7.4 Hz, 3H).

$^{13}C\{^1H\}$ NMR (126 MHz, $CDCl_3$) δ 148.8, 141.2, 140.2, 135.4, 130.5, 130.2, 129.5, 128.8, 128.2, 127.4, 126.2, 125.2, 53.0, 47.6, 29.9, 26.9, 22.5, 13.9.

HRMS–ESI (*m/z*): $[M + H]^+$ calcd for $C_{23}H_{25}ClN_5O^+$, 422.1742; found, 422.1732.

2.6. Preparation of Tris((1-benzyl-4-triazolyl)methyl)amine (TBTA)

Tris((1-benzyl-4-triazolyl)methyl)amine (TBTA) was synthesized following a slightly modified literature procedure.[8]

A mixture of benzyl azide (400 mg, 3 mmol, 1 equiv.), tripropargylamine (131 mg, 1 mmol, 1 equiv.), and $Cu(PPh_3)_3Br$ (0.06 mmol, 55 mg, 2 mol %) in 4 mL of chloroform was stirred at room temperature overnight. The reaction mixture was dissolved in hot ethyl acetate and the product was precipitated by addition of light petroleum. After filtration and drying, pure product was isolated as a white solid (497 mg, 94%).

^1H NMR (500 MHz, $CDCl_3$) δ 7.65 (s, 1H), 7.38–7.31 (m, 9H), 7.27–7.23 (m, 6H). ^1H NMR data of is in agreement with the literature reports.[16]

2.7. Synthesis and characterization of Fmoc-L-propargylglycine-methyl-L-tryptophanate (methyl ((S)-2-((((9H-fluoren-9-yl)methoxy)carbonyl)amino)pent-4-ynoyl)-L-tryptophanate) (1x)

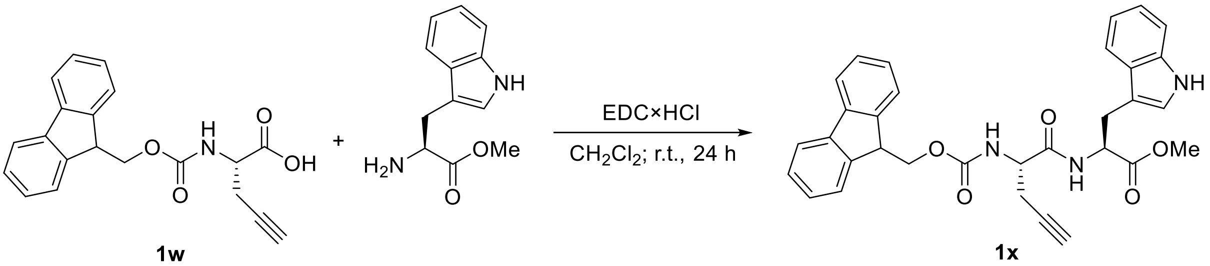

Procedure: 1x was synthesized according to the modified literature procedure.[7]

Reaction was performed in 100 mL round-bottomed flask. Fmoc-L-propargylglycine (**1w**) (1006 mg, 3 mmol, 1 equiv.) and methyl-L-tryptophanate (655 mg, 3 mmol, 1 equiv.) were added followed by CH_2Cl_2 (25 mL). EDC×HCl (*N*-(3-dimethylaminopropyl)-*N'*-ethylcarbodiimide hydrochloride) (575 mg, 3 mmol, 1 equiv.) was added to this solution and the resulting solution was left to stirr overnight at room temperature. Then, CH_2Cl_2 (20 mL) was added and the resulting solution was washed with water (2 × 30 mL) in separatory funnel. Combined organic layers were dried over anhydrous Na_2SO_4, filtered and the solvent was evaporated with the aid of rotary evaporator. Crude product was purified by silica column chromatography using Interchim puriFlash® XS520 (Column: Interchim PF-25SIHC-F0040 (40 g, 25 µm SiO_2), isocratic elution: CH_2Cl_2/MeOH; 99/1, flow rate: 26 mL/min) to afford white crystalline solid (1100 mg, 68%).

^{1}H NMR (500 MHz, $CDCl_3$) δ 8.06 (s, 1H), 7.78 (d, *J* = 7.5 Hz, 2H), 7.56 (dd, *J* = 16.5, 7.3 Hz, 2H), 7.51 (d, *J* = 7.9 Hz, 1H), 7.41 (t, *J* = 6.7 Hz, 2H), 7.34–7.27 (m, 3H), 7.14 (t, *J* = 7.4 Hz, 1H), 7.08 (t, *J* = 7.4 Hz, 1H), 6.92 (d, *J* = 1.6 Hz, 1H), 6.83 (d, *J* = 7.3 Hz, 1H), 5.58 (d, *J* = 7.9 Hz, 1H), 4.97–4.89 (m, 1H), 4.44–4.33 (m, 2H), 4.29–4.22 (m, 1H), 4.19 (t, *J* = 7.0 Hz, 1H), 3.67 (s, 3H), 3.32 (d, *J* = 5.4 Hz, 2H), 2.75–2.65 (m, 1H), 2.56 (dd, *J* = 16.7 Hz, 5.1 Hz, 1H), 1.90 (s, 1H).

^{13}C{^{1}H} NMR (126 MHz, $CDCl_3$) δ 171.9, 169.1, 156.0, 143.9, 141.4, 136.2, 128.0, 127.7, 127.29, 127.26, 125.3, 125.2, 123.1, 122.4, 120.2, 119.9, 118.6, 111.5, 109.8, 79.2, 72.0, 67.4, 53.6, 53.4, 53.3, 52.6, 47.1, 27.6, 22.7.

HRMS–ESI (*m/z*): $[M + H]^+$ calcd for $C_{32}H_{30}N_3O_5^+$, 536.2180; found, 536.2179.

2.8. Preparation of (2-butyl-4-chloro-1-((2'-ethynyl-[1,1'-biphenyl]-4-yl)methyl)-1H-imidazol-5-yl)methanol (1u)

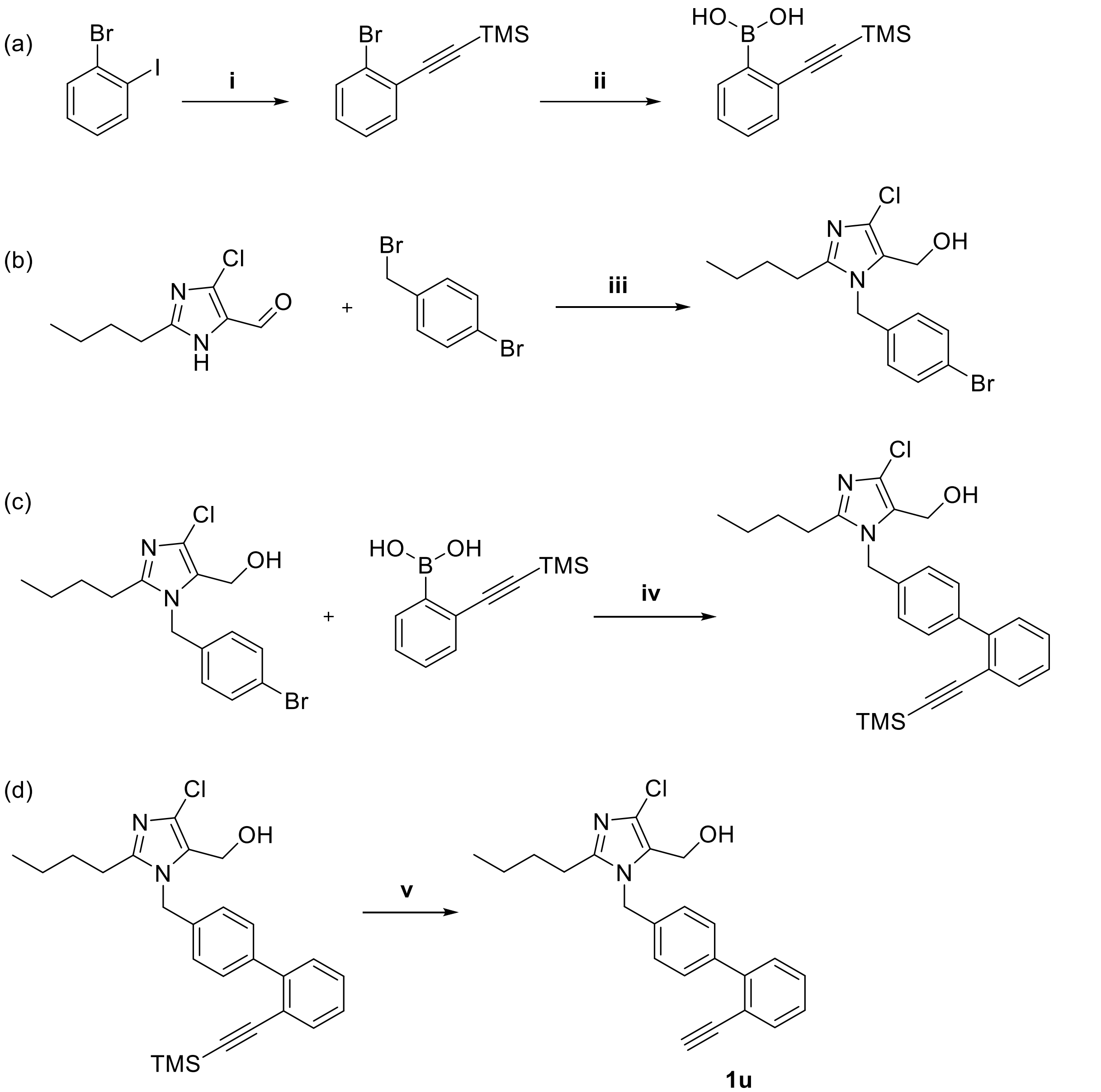

1u

i: Following literature procedure.[17]

ii: Following literature procedure.[18]

iii: Following literature procedure.[19]

iv: Following slightly modified literature procedure which reported reaction on similar substrates.[19] Reaction was performed using Schlenk-line techniques in a 100 mL round-bottomed Schlenk flask (29/32) with rubber septum and appropriate reflux condenser.

To a degassed of solvent mixture of THF:diethoxymethane 1:4 (v/v, 25 mL), were added PPh_3 (46 mg, 0.18 mmol, 4 mol%) and $Pd(OAc)_2$ (20 mg, 0.09 mmol, 2 mol%). Resulting mixture was stirred for 30 minutes at room temperature under nitrogen atmosphere. Then, boronic acid (1000 mg, 4.6 mmol, 1.05 equiv.) was added and the mixture was stirred at room temperature for 30 minutes under nitrogen atmosphere. After that time, water (183 μL, 10.12 mmol, 2.3 equiv.) was added and reaction mixture was again let to stirr at room temperature for 30 minutes, followed by addition of K_2CO_3 (1520 mg, 11 mmol, 2.5 equiv.) and arylbromide (1580 mg, 4.4 mmol, 1 equiv.). The reaction mixture was refluxed using aluminum heating mantle under inert nitrogen atmosphere for 6 hours. The reaction mixture was allowed to cool to 50 °C and of THF (6.3 mL) and water (7.5 mL) were added. Two phases were formed and were separated in separatory funnel. Organic phase was collected and aqueous phase was additionally extracted with EtOAc (2 x 30 mL). Organic phases were combined, dried over anhydrous sodium sulfate, filtered and evaporated under reduced pressure with the aid of rotary evaporator. Crude product was purified by column chromatography using Interchim puriFlash® XS520 (Column: Interchim PF-25SIHC-F0080 (80 g, 25 μm SiO_2), gradient elution: petroleum ether → ethyl acetate; 00:00 – 08:00; 94% PE → 92% PE, 08:00 – 20:00; 92% PE → 83% PE, 20:00 – 39:00; 83% PE → 46% PE, 39:00 – 50:00; 46% PE → 7% PE, 50:00 – 1:05:00 7% PE, flow rate: 34 mL/min) to afford desired product (1221 mg, 61%) as a yellow waxy solid (61%).

IR (cm^{-1}): 2957, 2871, 2156, 1575, 1442, 1248, 1004, 862, 839, 756.

^1H NMR (500 MHz, $CDCl_3$) δ 7.60–7.55 (m, 3H), 7.39–7.31 (m, 2H), 7.30–7.26 (m, 1H), 7.02 (d, J = 8.1 Hz, 2H), 5.27 (s, 2H), 2.63–2.57 (m, 2H), 1.74–1.66 (m, 2H), 1.41–1.31 (m, 2H), 0.89 (t, J = 7.3 Hz, 3H), 0.11 (s, 9H).

$^{13}C\{^1H\}$ NMR (126 MHz, $CDCl_3$) δ 148.9, 143.4, 140.2, 135.3, 133.5, 130.2, 129.4, 129.0, 127.8, 127.4, 125.4, 124.9, 121.5, 104.7, 97.9, 53.5, 47.5, 30.0, 27.0, 22.7, 13.9, 0.19.

HRMS–ESI (m/z): $[M + H]^+$ calcd for $C_{26}H_{32}ClN_2OSi^+$, 451.1967; found, 451.1963.

v: Reaction was performed using Schlenk-line techniques in an oven dried 25 mL round-bottom Schlenk flask capped with septum. To a solution of TMS-protected acetylene (614 mg, 1.36 mmol, 1 equiv.) in dry THF (4 mL), tetra-*n*-butylammonium fluoride (TBAF) (4 mL of 1M solution in THF, 4 mmol, 3 equiv. of TBAF) was added dropwise at 0 °C under nitrogen atmosphere. The reaction mixture was allowed to warm to room temperature and was stirred for 3 hours. Progress of the reaction was monitored by TLC. After consumption of starting material THF was removed under reduced pressure with the aid

of rotary evaporator. The crude product was purified using Interchim puriFlash® XS520 (Column: Interchim PF-25SIHC-F0040 (40 g, 25 μm SiO_2), gradient elution: petroleum ether → ethyl acetate; 00:00 – 05:00 94% PE → 92% PE, 05:00 – 13:00 92% PE → 83% PE, 13:00 – 24:00 83% PE → 46% PE, 24:00 – 31:00 47% PE → 7% PE, 31:00 – 40:00 7% PE, flow rate: 26 mL/min) to afford product (**1u**) as a yellow solid (444 mg, 86%).

^{1}H NMR (500 MHz, $CDCl_3$) δ 7.61 (dd, *J* = 7.7, 1.4 Hz, 1H), 7.58–7.54 (m, 2H), 7.41 (td, *J* = 7.6, 1.4 Hz, 1H), 7.36–7.29 (m, 2H), 5.27 (s, 2H), 4.54 (d, *J* = 5.7 Hz, 2H), 3.03 (s, 1H), 2.63–2.56 (m, 2H), 1.77 (t, *J* = 5.8 Hz, 1H), 1.71–1.63 (m, 2H), 1.39–1.30 (m, 2H), 0.87 (t, *J* = 7.4 Hz, 3H) .

^{13}C{^{1}H} NMR (126 MHz, $CDCl_3$) δ 148.9, 143.6, 140.1, 135.5, 134.1, 160.1, 129.6, 129.3, 127.7, 127.5, 125.7, 124.9, 120.5, 83.1, 80.6, 53.5, 47.5, 29.9, 27.0, 22.6, 13.9.

HRMS–ESI (*m/z*): $[M + H]^+$ calcd for $C_{23}H_{24}ClN_2O^+$, 379.1572; found, 379.1565.

2.9. Competitive ketone-forming reaction (Fig. 4)

Reaction by using developed Method A:

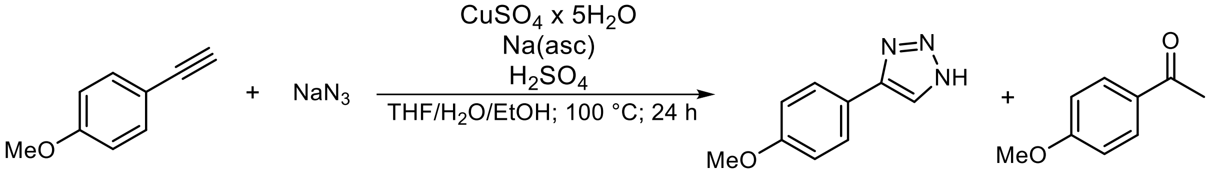

1d **2** **3d** **5d**

Into a 21 mL ACE pressure tube stirring bar, 1-ethynyl-4-methoxybenzene (**1d**) (132 mg, 1.00 mmol, 1 equiv.), $CuSO_4 \times 5H_2O$ (51 mg, 0.20 mmol, 20 mol%), Na(asc) (198 mg, 1.00 mmol, 1 equiv.), NaN_3 (98 mg, 1.50 mmol, 1.5 equiv.), 2.5 mL of solvent mixture THF:H_2O:EtOH 2:2:1 (v/v/v), 96% H_2SO_4 (89 µL, 1.6 mmol, 1.6 equiv.) were added in that order. After addition of sulfuric acid, ACE tube was sealed with a screw cap and reaction mixture was stirred for 24 hours at 100 °C in aluminum heating block. After that time, the reaction mixture was allowed to cool to room temperature in a closed ACE pressure tube. Prior to work-up, ACE tube was uncapped and left open for a few minutes in the fume hood to allow any excess hydrazoic acid in vapor phase to evaporate. Ethyl acetate (30 mL) and ammonium chloride solution (20 mL sat. NH_4Cl + 20 mL H_2O) were added to the raction mixture, and phases were separated in separatory funnel. Aqueous phase was additionally extracted with ethyl acetate (2 × 30 mL). To combined organic phases 41.2 mg (0.2450 mmol) of 1,3,5-trimethoxybenzene (TMB) was added. Aliquote of organic solution was taken and evaporated with the aid of rotary evaporator and ^{1}H NMR spectrum of an aliquote was recorded. Conversion of **1d** into **3d** and **5d** was 58% and 40% respectively.

Ketone product **5d** was isolated using silica column chromatography and characterized by ^{1}H NMR and HRMS.

^{1}H NMR (500 MHz, $CDCl_3$) δ 7.99–7.89 (m, 2H), 6.96–6.91 (m, 2H), 3.87 (s, 3H), 2.56 (s, 3H).

HRMS–ESI (*m/z*): [M + H]$^+$ calcd for $C_9H_{11}O_2^+$, 151.0754; found, 151.0751.

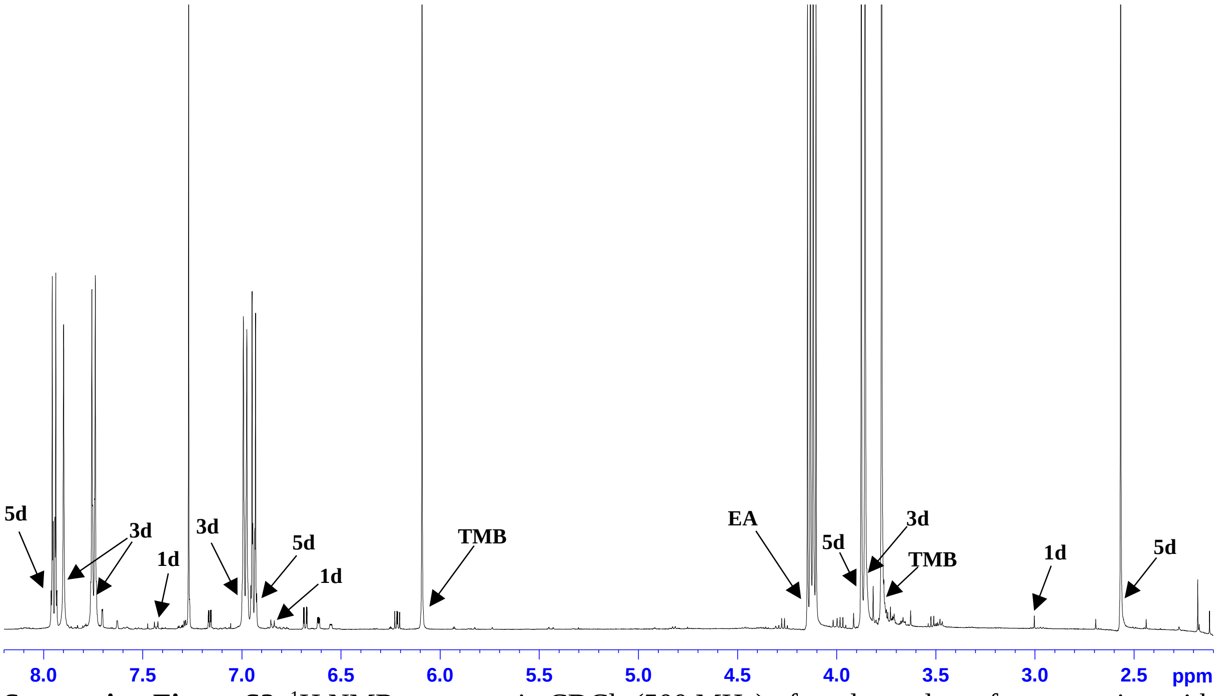

Supporting Figure S3. ^{1}H NMR spectrum in $CDCl_3$ (500 MHz) of crude product after extraction with ethyl acetate. Representative signals for acetylene (**1d**) triazole (**3d**), ketone (**5d**), residual ethyl acetate (EA) and 1,3,5-trimethoxybenzene (TMB) are labeled.

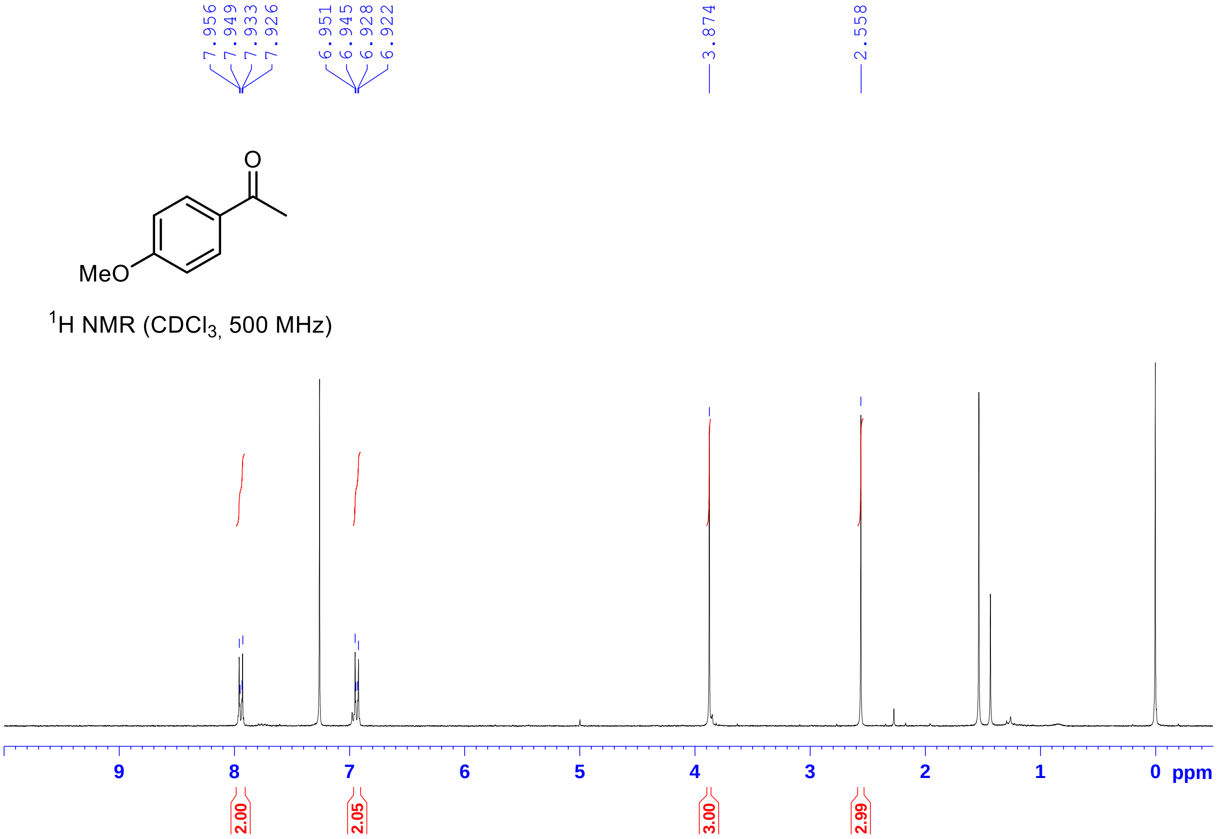

Supporting Figure S4. ^{1}H NMR spectrum of ketone **5d**. Signals at δ 1.54 and 1.43 ppm correspond to water and residual cyclohexane.

Reaction by using developed Method C:

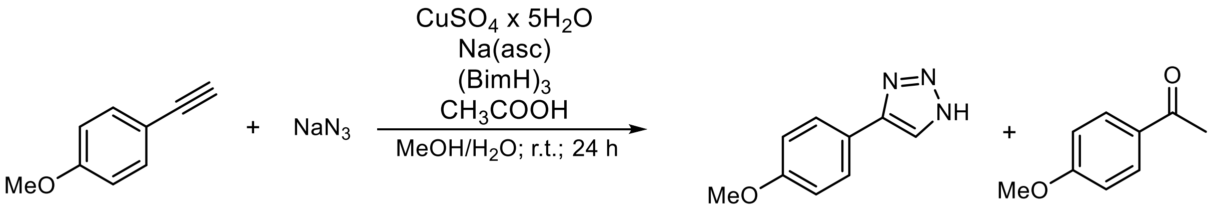

1d 2 3d 5d

Into a 5 mL round bottom flask stirring bar, 1-ethynyl-4-methoxybenzene (**1d**) (65 mg, 0.49 mmol, 1 equiv.), $CuSO_4 \times 5H_2O$ (6.3 mg, 0.025 mmol, 5 mol%), Na(asc) (25 mg, 0.126 mmol, 0.25 equiv.), $(BimH)_3$ (10.2 mg, 0.025 mmol, 5 mol%), NaN_3 (49 mg, 0.75 mmol, 1.5 equiv.), 0.6 mL of solvent mixture in volume ratio MeOH:H_2O 3:1 (v/v), CH_3COOH (190 µL, 3.3 mmol, 6.6 equiv.) were added in that order. After the addition of acetic acid flask was capped and the reaction mixture was stirred for 24 hours at room temperature. Then, the reaction flask was uncapped and the reaction mixture was filtered trough a short pad of silica gel using ethyl acetate as eluent. Into resulting filtrate 1,3,5-trimethoxybenzene (TMB) (17.9 mg, 0.1064 mmol) was added. Aliquote was taken and solvent was removed by the aid of rotatry evaporator. ^{1}H NMR spectrum of collected aliquote was recorded. Conversion of **1d** into **3d** and **5d** was determined to be 95% and 5% respectively, by comparing integrals in ^{1}H NMR spectrum (see Supporting Figure S5).

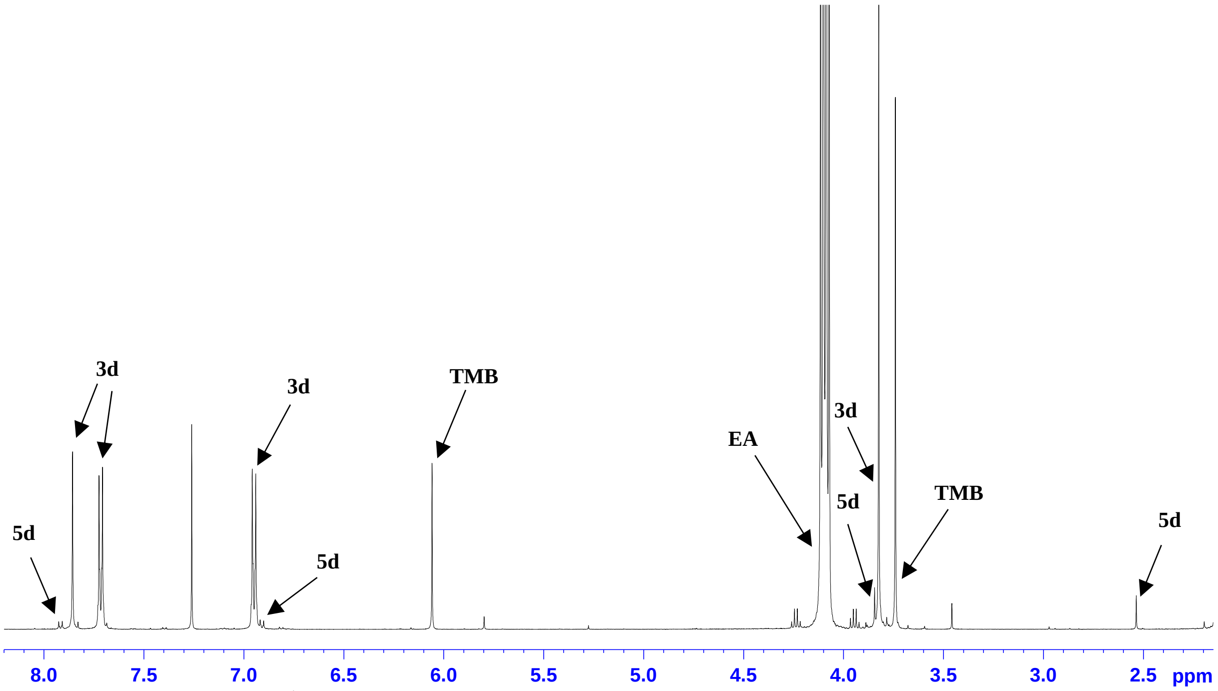

Supporting Figure S5. ^{1}H NMR spectrum of crude product in $CDCl_3$ (500 MHz) after filtration trough silica with ethyl acetate. Representative signals for triazole (**3d**), ketone (**5d**), residual ethyl acetate (EA) and 1,3,5-trimethoxybenzene (TMB) are labeled.

Reaction by using developed Method C at 100 °C:

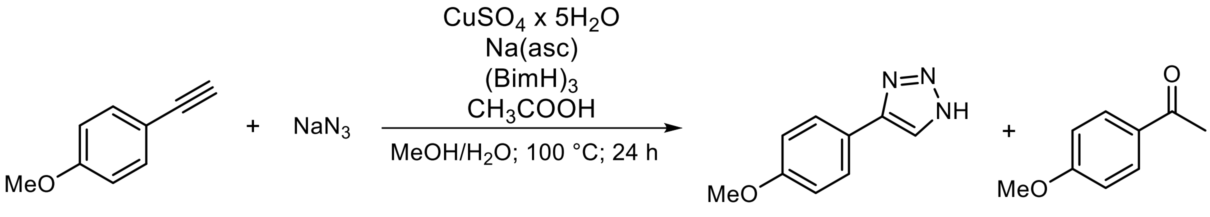

Into a 8 mL ACE pressure tube were added stirring bar, 1-ethynyl-4-methoxybenzene (**1d**) (65 mg, 0.49 mmol, 1 equiv.), $CuSO_4 \times 5H_2O$ (6.3 mg, 0.025 mmol, 5 mol%), Na(asc) (25 mg, 0.126 mmol, 0.25 equiv.), $(BimH)_3$ (10.2 mg, 0.025 mmol, 5 mol%), NaN_3 (49 mg, 0.75 mmol, 1.5 equiv.), 0.6 mL of solvent mixture in volume ratio MeOH:H_2O 3:1 (v/v) and CH_3COOH (190 µL, 3.3 mmol, 6.6 equiv.). After the addition of acetic acid ACE pressure tube was capped and the reaction mixture was stirred for 24 hours at 100 °C in aluminum heating block. Then, the reaction mixture was filtered trough a short pad of silica gel using ethyl acetate as eluent. Into resulting filtrate 1,3,5-trimethoxybenzene (TMB) (19.7 mg, 0.1177 mmol) was added. Aliquote was taken and solvent was removed by the aid of rotatry evaporator. ^1H NMR spectrum of collected aliquote was recorded. Conversion of **1d** into **3d** and **5d** was determined to be 96% and 4% respectively, by comparing integrals in ^1H NMR spectrum.

2.10. Reactions of azahistidine and peptides (Fig. 5)

Conditions 1 for reactions of 1w and 1x:

Into an 8- or 21-mL ACE pressure tube were added stirring bar, alkyne (1 equiv.), CuI (0.2 equiv., 20 mol%), TBTA (0.1 equiv., 10 mol%), sodium ascorbate (0.2 equiv.), sodium azide (1.5 equiv.), solvent and HCOOH (5 equiv.) in that order. After addition of methanoic acid ACE tube was sealed with a screw cap and left to stir for 24 hours at 40 °C in aluminum heating block.

Fmoc-L-azahistidine (2*S*-((((9H-fluoren-9-yl)methoxy)carbonyl)amino)-3-(1*H*-1,2,3-triazol-4-yl)propanoic acid) (3w, Fig. 5)

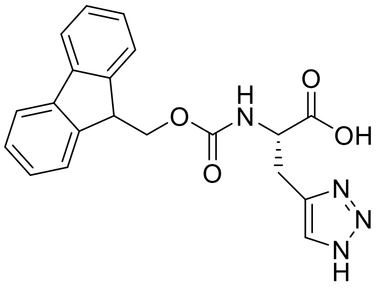

Prepared according to *Conditions 1 for reactions of* ***1w*** *and* ***1x***. Using 20 mL ACE pressure tube, Fmoc-L-propargylglycine (**1w**) (335 mg, 1 mmol), CuI (38 mg, 0.2 mmol, 20 mol%), TBTA (53 mg, 0.1 mmol 10 mol%), sodium ascorbate (40 mg, 0.2 mmol, 0.2 equiv.), NaN_3 (98 mg, 1.5 mmol, 1.5 equiv.), $MeOH:H_2O$ 5:1 (v/v, 1.2 mL), HCOOH (189 µL, 5 mmol, 5 equiv.). Product was extracted with EtOAc (3 × 20 mL) after addition of ammonium chloride solution (20 mL sat. NH_4Cl + 20 mL H_2O). Organic phases were dried over anhydrous Na_2SO_4 and concentrated under reduced pressure with the aid of rotary evaporator. Crude product was purified by silica gel column chromatography using Interchim puriFlash® XS520 (Column: Interchim PF-25SIHC-F0025 (25 g, 25 µm SiO_2), gradient elution (CH_2Cl_2–MeOH): 00:00–20:00: 100% CH_2Cl_2 → 80% CH_2Cl_2; 20:00–30:00: 80% CH_2Cl_2 → 60% CH_2Cl_2; 30:00–35:00: 60% CH_2Cl_2, flow rate: 15 mL/min). Yellow solid, 170 mg (45%).

Mp.: 137–140 °C.

IR (cm^{-1}): 3066, 2944, 2911, 1696, 1523, 1448, 1233, 1043, 758, 737.

^1H NMR (500 MHz, DMSO-d_6) δ 7.88 (d, J = 7.5 Hz, 2H), 7.68–7.63 (m, 2H), 7.54 (s, 1H), 7.41 (t, J = 7.5 Hz, 2H), 7.34–7.28 (m, 3H), 4.25–4.16 (m, 3H), 4.13–4.07 (m, 1H), 3.18–3.12 (m, 1H), 3.04–2.99 (m, 1H).

$^{13}C\{^1H\}$ NMR (126 MHz, DMSO-d_6) δ 173.0, 155.7, 143.87, 143.82, 140.7, 127.6, 127.1, 125.32, 125.26 120.1, 65.6, 54.4, 46.6, 27.0.

HRMS–ESI (*m/z*): [M + H]$^+$ calcd for $C_{20}H_{19}N_4O_4^+$, 379.1401; found, 379.1399.

Synthesis of Fmoc-azahistidine on 3 mmol scale (3w, Fig. 5)

20 mL ACE pressure tube, Fmoc-propargylglycine (**1w**) (1006 mg, 3 mmol), CuI (114 mg, 0.6 mmol, 20 mol%), TBTA (159 mg, 0.3 mmol, 10 mol%), sodium ascorbate (120 mg, 0.6 mmol, 0.2 equiv.), NaN_3 (294 mg, 4.5 mmol, 1.5 equiv.), MeOH:H_2O 5:1 (v/v, 6 mL), HCOOH (567 µL, 15 mmol, 5 equiv.). Product was extracted with EtOAc (3 × 50 mL) after addition of ammonium chloride solution (40 mL sat. NH_4Cl + 40 mL H_2O). Organic phase was dried over anhydrous Na_2SO_4 and concentrated under reduced pressure. Product was purified with silica column chromatography using Interchim puriFlash® XS520 (Column: Interchim PF-25SIHC-F0040 (40 g, 25 µm SiO_2), gradient elution (CH_2Cl_2–MeOH): 00:00–20:00: 100% CH_2Cl_2 → 90% CH_2Cl_2; 20:00–40:00: 90% CH_2Cl_2 → 50% CH_2Cl_2; flow rate: 26 mL/min). Yellow solid, 450 mg (40%).

Fmoc-*L*-azaHis-*L*-TrpOMe (methyl ((S)-2-((((9H-fluoren-9-yl)methoxy)carbonyl)amino)-3-(1*H*-1,2,3-triazol-4-yl)propanoyl)-L-tryptophanate) (3x, Fig. 5)

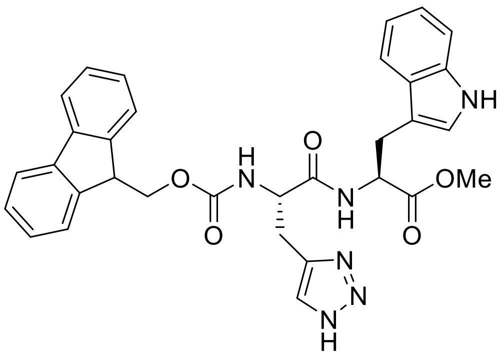

Prepared according to modified *Conditions 1 for reactions of **1w** and **1x***. 5 mL ACE pressure tube, Fmoc-L-propargylglycine-methyl-L-tryptophanate (**1x**) (54 mg, 0.1 mmol), CuI (3.8 mg, 0.02 mmol, 20 mol%), TBTA (5.3 mg, 0.01 mmol, 10 mol%), sodium ascorbate (4 mg, 0.02 mmol, 0.2 equiv.), NaN_3 (9.8 mg, 0.15 mmol, 1.5 equiv.), DMF (0.2 mL), HCOOH (19 µL, 0.5 mmol, 5 equiv.). Reaction mixture was filtered trough a pad of silica using EtOAc. Organic phase was concentrated under reduced pressure with the aid of rotary evaporator and product was purified using Interchim puriFlash® XS520 (Column: Interchim PF-50SIHC-F0004 (4 g, 50 µm SiO_2), gradient elution: 00:00–20:00: 100% petroleum ether → 100% ethyl acetate; 20:00–25:00: 100% ethyl acetate, flow rate: 5 mL/min). Yellow solid, 42 mg (73%).

Mp.: 202–205 °C.

IR (cm^{-1}): 3417, 3238, 1730, 1719, 1660, 1530, 1441, 1286, 1219, 1042, 737.

^{1}H NMR (500 MHz, DMSO-d_6) δ 10.88 (s, 1H), 8.48 (d, J = 6.4 Hz, 1H), 7.88 (d, J = 7.6 Hz, 2H), 7.66 (t, J = 7.3 Hz, 3H), 7.48 (d, J = 7.9 Hz, 1H), 7.41 (t, J = 7.4 Hz, 2H), 7.35–7.28 (m, 3H), 7.17 (d, J = 1.9 Hz, 1H), 7.06 (t, 7.3 Hz, 1H), 6.98 (t, J = 7.3 Hz, 1H), 4.56–4.50 (m, 1H), 4.41–4.34 (m, 1H), 4.27–4.20 (m, 1H), 4.20–4.14 (m, 2H), 3.56 (s, 3H), 3.19–3.13 (m, 1H), 3.12–3.04 (m 2H), 2.95–2.88 (m, 1H).

^{13}C{^{1}H} NMR (126 MHz, DMSO-d_6) δ 172.1, 171.3, 155.7, 143.8, 143.7, 140.7, 136.1, 127.7, 127.10, 127.09, 127.05, 125.4, 125.3, 123.8, 121.0, 120.1, 118.5, 118.0, 111.4, 109.2, 65.7, 54.2, 53.2, 51.9, 46.6, 28.0, 26.9. HRMS–ESI (m/z): [M + H]$^+$ calcd for $C_{32}H_{31}N_6O_5^+$, 579.2350; found, 579.2343.

Structures of peptides in Fig. 5:

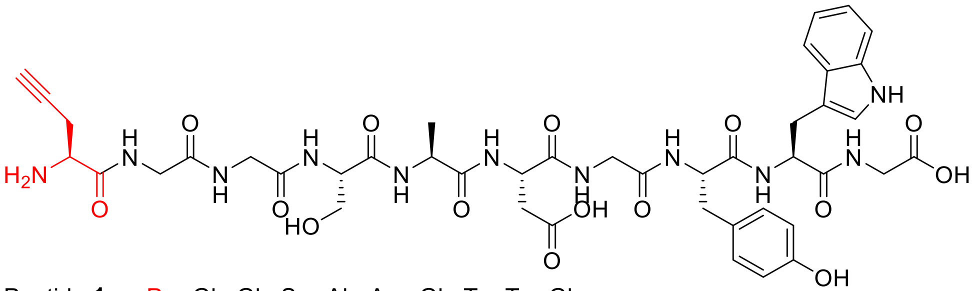

Peptide **1aa**: Pra-Gly-Gly-Ser-Ala-Asp-Gly-Tyr-Trp-Gly
Exact mass: 963.3723

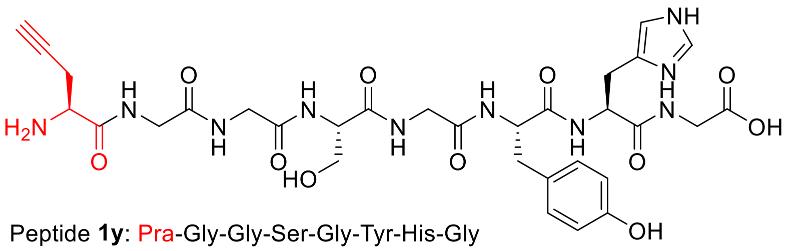

Peptide **1y**: Pra-Gly-Gly-Ser-Gly-Tyr-His-Gly
Exact mass: 728.2878

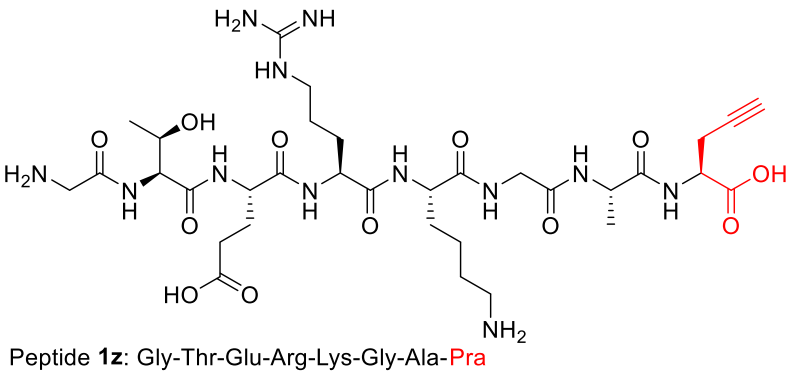

Peptide **1z**: Gly-Thr-Glu-Arg-Lys-Gly-Ala-Pra
Exact mass: 812.4141

Two different reaction conditions were employed for the reactions of peptides: *Conditions 2* were based upon modified literature procedure for CuAAC of biomolecules[20] - $CuSO_4$ and THPTA (tris((1-hydroxy-propyl-1*H*-1,2,3-triazol-4-yl)methyl)amine, water soluble analogue of TBTA) were employed.

Conditions 1: 50 µL of reaction mixture (35 µL DMF and 15 µL H_2O). Temperature: 40 °C.

- 100 mM peptide
- 200 mM NaN_3
- 50 mM CuI
- 100 mM Na(asc)
- 50 mM TBTA
- 500 mM HCOOH
- 50 mM internal standard (protected phenylalanine as internal standard; Fmoc-Phe-OEt)

Conditions 2: 100 µL of reaction mixture (30 µL DMF and 70 µL H_2O). Temperature: r.t.–40 °C.

- 50 mM peptide
- 100 mM NaN_3
- 25 mM $CuSO_4$
- 200 mM Na(asc)
- 125 mM THPTA
- 500 mM HCOOH

For details of HPLC analysis parameters see 1.4. General analytical information.

HPLC analyses of individual reagents:

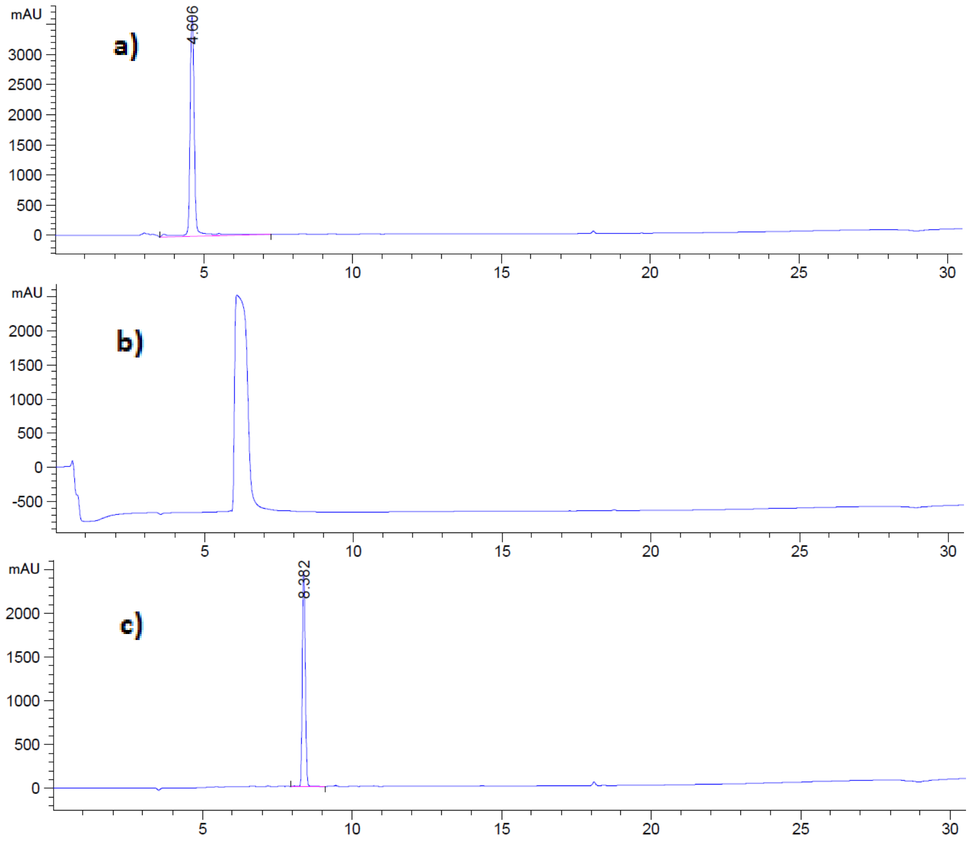

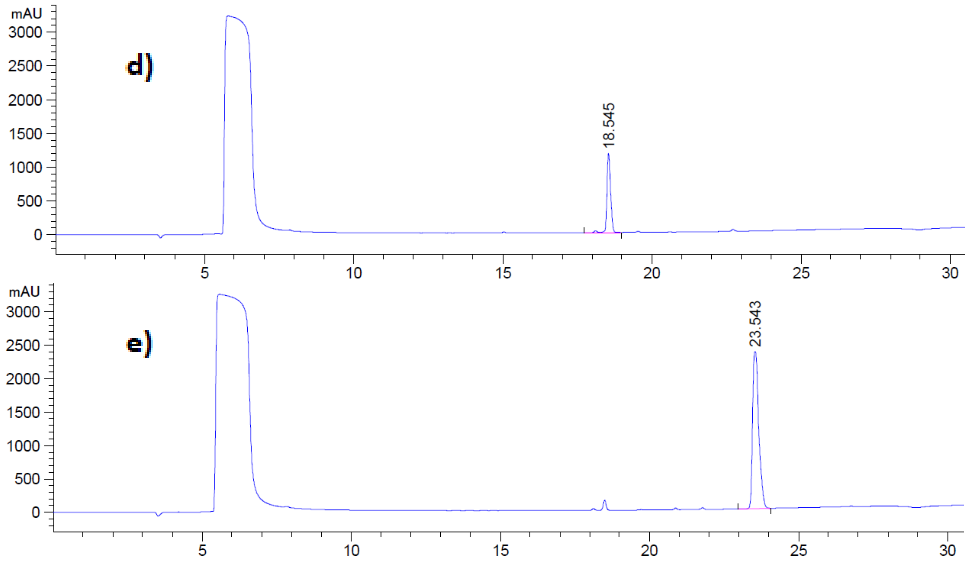

Supporting Figure S6. HPLC chromatograms of individual compounds: **a)** sodium ascorbate, **b)** DMF, **c)** THPTA, **d)** TBTA solution in DMF, **e)** internal standard solution in DMF.

HPLC analyses of starting peptides:

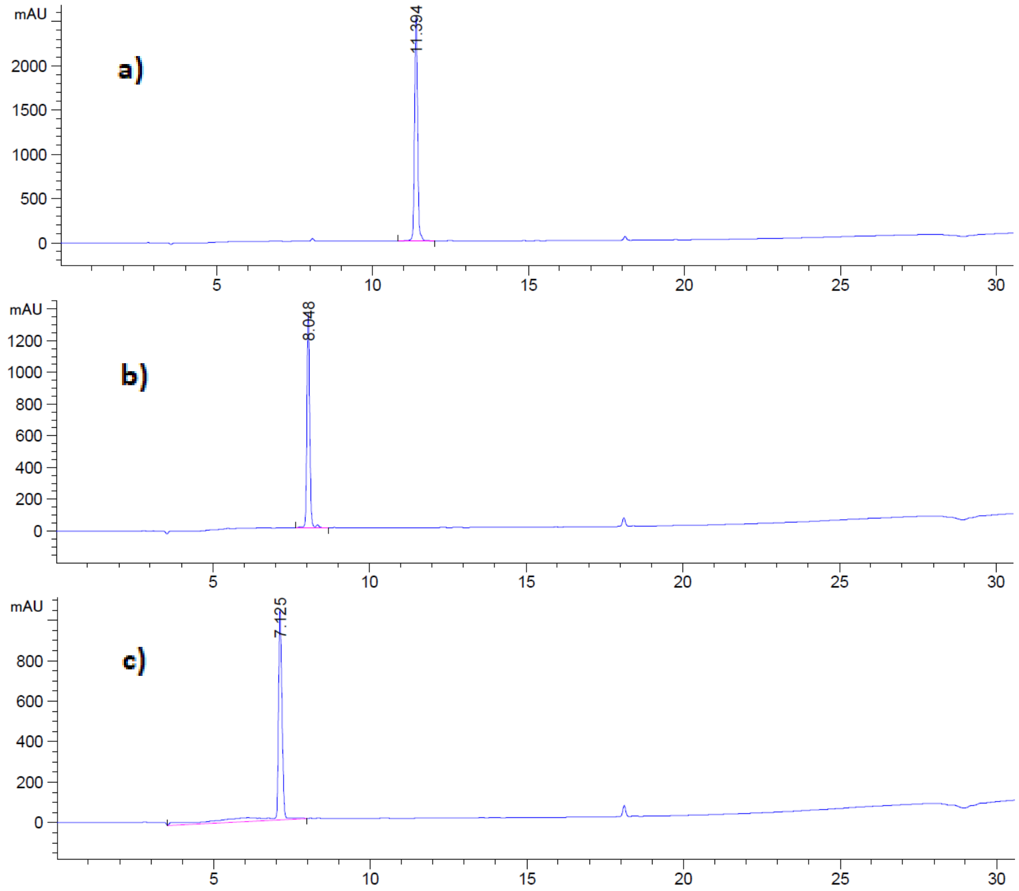

Supporting Figure S7. HPLC chromatograms of individual peptides with alkyne handle: **a)** peptide **1aa**, **b)** peptide **1y**, **c)** peptide **1z** using the same optimized elution parameters.

Reaction on Peptide 1aa, Conditions 2:

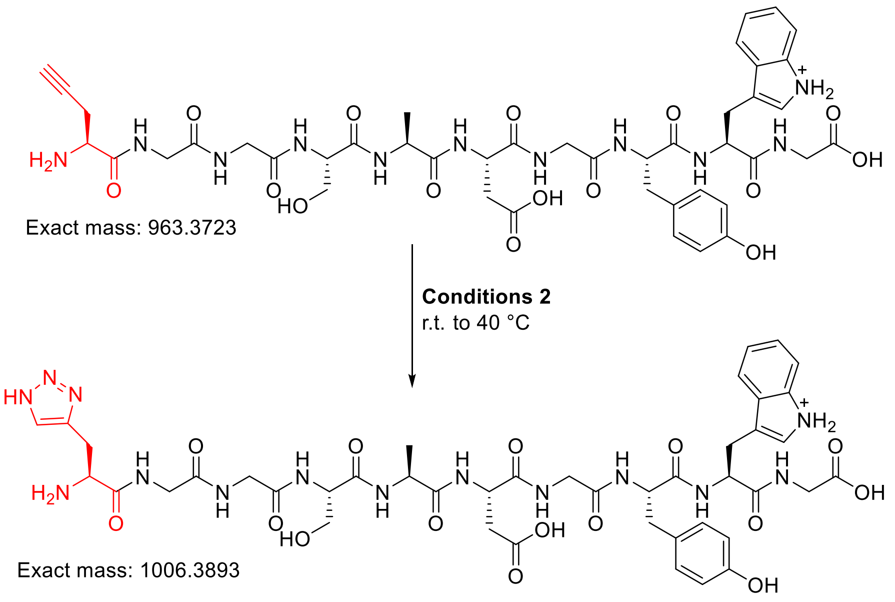

Peptide stock solution (DMF:H_2O 1:1, v/v):

- 125 mM peptide **1aa**

Reagents stock solution (DMF:H_2O 1:4, v/v):

- 250 mM THPTA
- 50 mM $CuSO_4$
- 400 mM Na(asc)
- 1.0 M HCOOH

Sodium azide stock solution (H_2O):

- 1.0 M NaN_3

Reaction was performed in a 200 µL Eppendorf tube. Stock solutions were added in the following order: 40 µL of peptide stock solution, 50 µL Reagents stock solution, 10 µL sodium azide stock solution. After addition of each stock solution reaction mixture was mixed by pipetting up and down 15-times.

Final concentrations of reagents were: peptide **1aa** (50 mM), NaN_3 (100 mM), HCOOH (500 mM), Cu (25 mM), THPTA (125 mM), Na(asc) (200 mM), DMF:H2O 3:7 (v/v).

Reaction was set at room temperature and was raised to 40 °C (Eppendorf tube in oil bath) after 3h since no conversion of **1aa** into **3aa** at room temperature. For analysis 10 µL of reaction mixture was taken, dilluted with 500 µL H_2O and analyzed by HPLC and HPLC-HRMS.

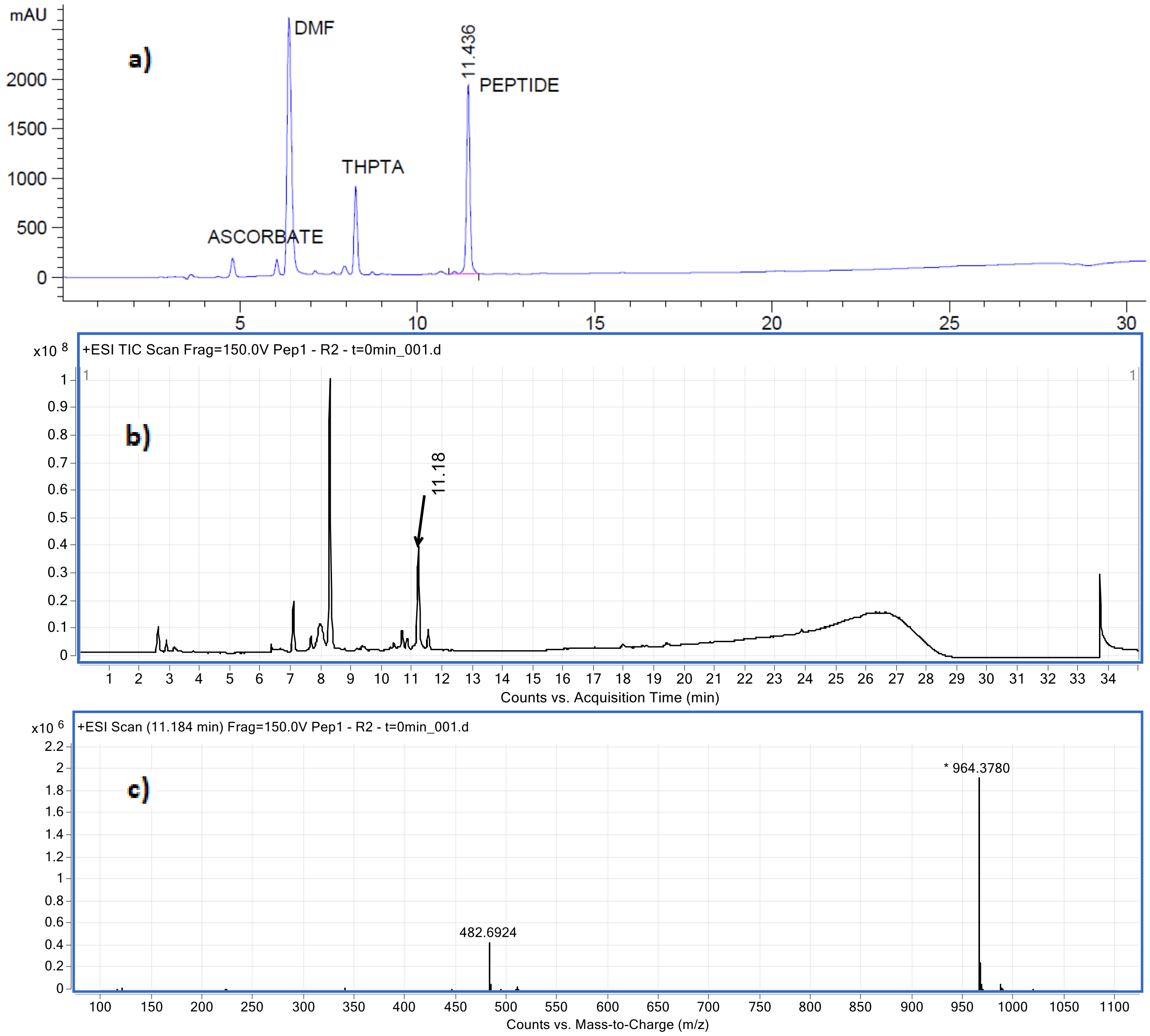

Supporting Figure S8. Reaction on peptide **1aa**, Conditions 2, aliquote was taken at reaction time t=0: **a)** HPLC chromatogram, **b)** TIC chromatogram of HPLC-HRMS measurement, **c)** HRMS spectrum of TIC chromatogram at RT=11.18 min. Signals representative for alkyne peptide **1aa**: found $[M+H]^+$ 964.3780, $[M+2H]^{2+}$ 482.6924; calculated 964.3795 for $C_{43}H_{54}N_{11}O_{15}^+$ $[M+H]^+$.

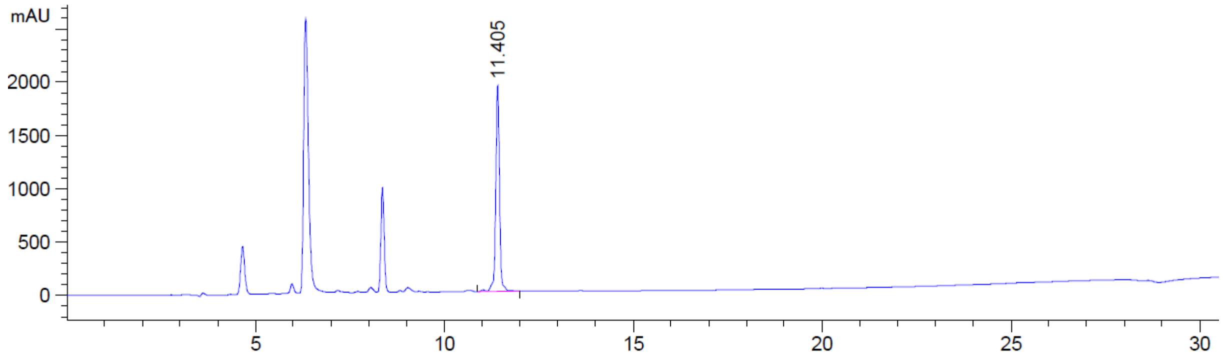

Supporting Figure S9. Reaction on peptide **1aa**, Conditions 2, aliquote was taken at reaction time t=3 h. HPLC chromatogram.

No conversion was observed after 3h. Reaction temperature was raised from RT to 40 °C.

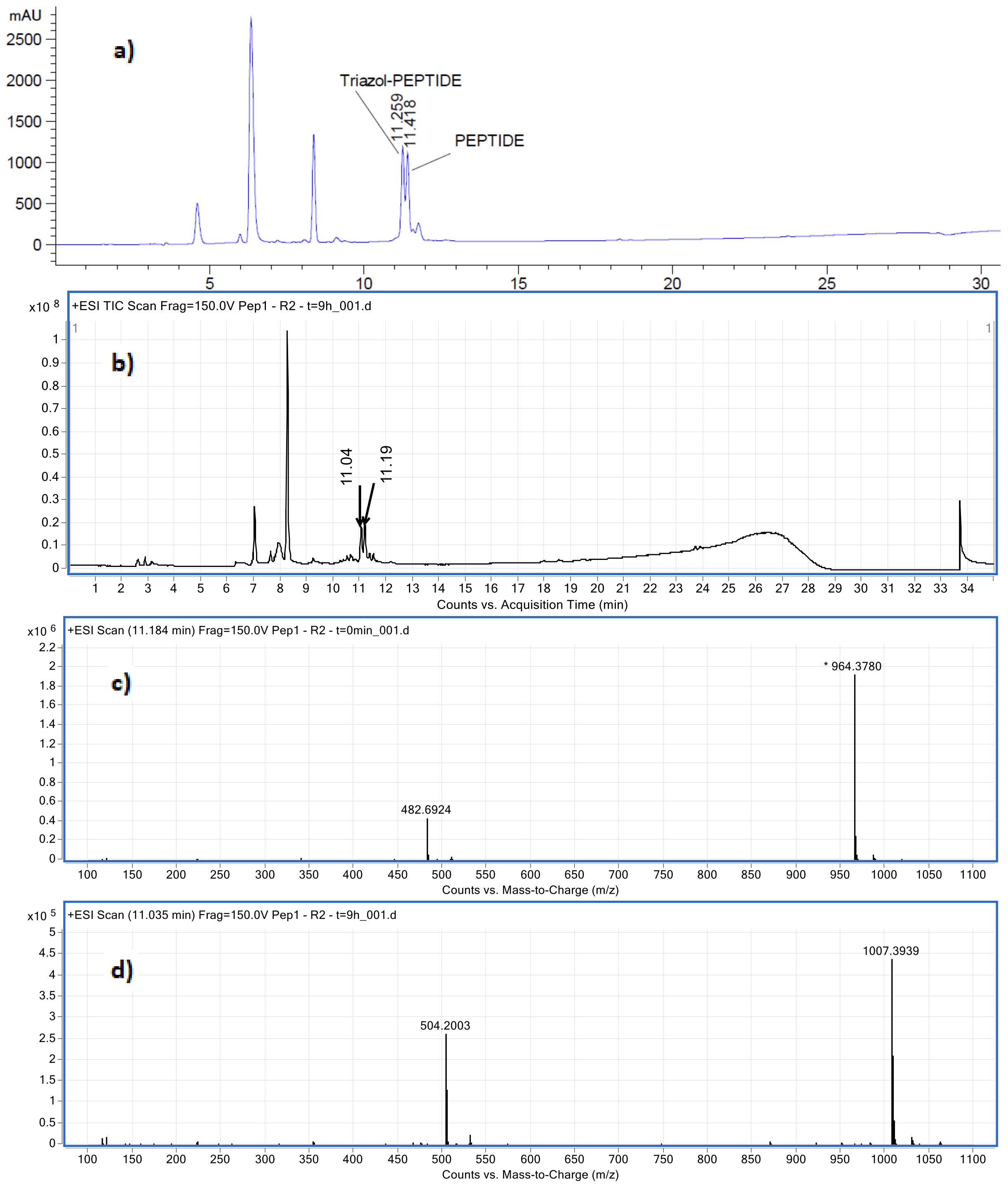

Supporting Figure S10. Reaction on peptide **1aa**, Conditions 2, aliquote was taken at reaction time t=9 h: **a)** HPLC chromatogram, **b)** TIC chromatogram of HPLC-HRMS measurement, **c)** HRMS spectrum of TIC chromatogram at RT=11.19 min. Signals representative for alkyne peptide **1aa**: $[M+H]^+$ 964.3780, $[M+2H]^{2+}$ 482.6924; calculated 964.3795 for $C_{43}H_{54}N_{11}O_{15}^+$ $[M+H]^+$. **d)** HRMS spectrum of TIC chromatogram at RT=11.04 min. Signals representative for triazole peptide **3aa** product: $[M+H]^+$ 1007.3939, $[M+2H]^{2+}$ 504.2003; calculated 1007.3966 for $C_{43}H_{55}N_{14}O_{15}^+$ $[M+H]^+$.

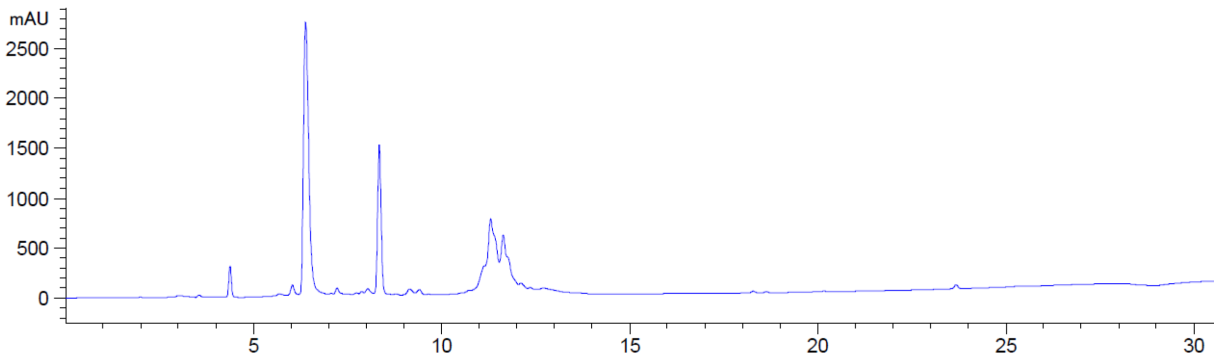

Supporting Figure S11. Reaction on peptide **1aa**, Conditions 2, aliquote was taken at reaction time t=24 h. HPLC chromatogram.

Reaction on Peptide 1aa, Conditions 1:

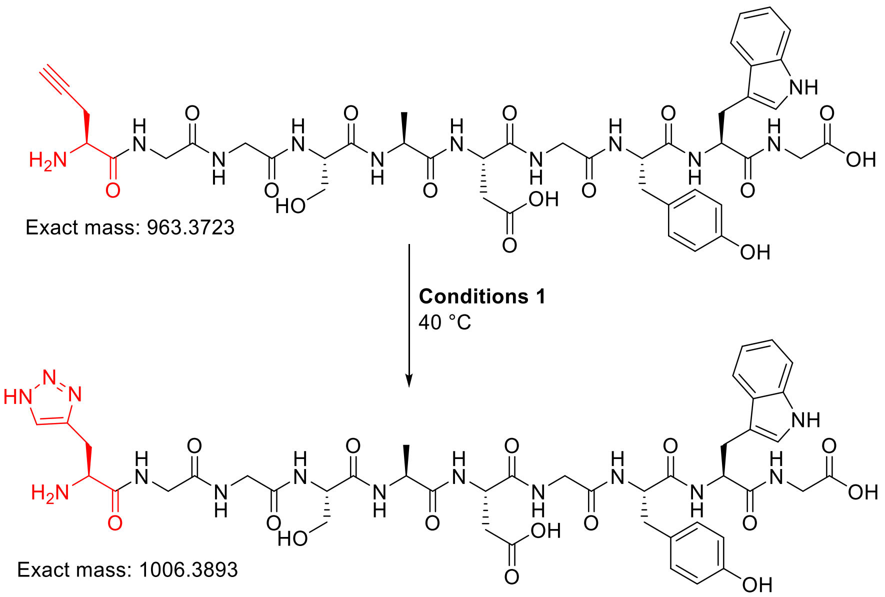

Peptide stock solution (DMF:H_2O, 1:1, v/v):
- 250 mM peptide **1aa**

Reagents stock solution (DMF):
- 100 mM TBTA
- 100 mM CuI
- 200 mM Na(asc)
- 1.0 M HCOOH
- 100 mM internal standard

Sodium azide stock solution (H_2O):
- 2.0 M NaN_3

Reaction was performed in 200 µL Eppendorf tube. Stock solutions were added in the following order: 20 µL peptide stock solution, 25 µL Reagents stock solution, 5 µL sodium azide stock solution. After addition of each stock solution reaction mixture was mixed by pipetting up and down 15-times. Reaction was set at 40 °C (Eppendorf tube in oil bath).

Final concentrations of reagents were: peptide **1aa** (100 mM), NaN_3 (200 mM), HCOOH (500 mM), Cu (50 mM), TBTA (50 mM), Na(asc) (100 mM), DMF:H_2O 7:3 (v/v).

For analysis 5 µL of reaction mixture was taken, dilluted with 250 µL DMF + 250 µL H_2O and analyzed by HPLC and HPLC-HRMS.

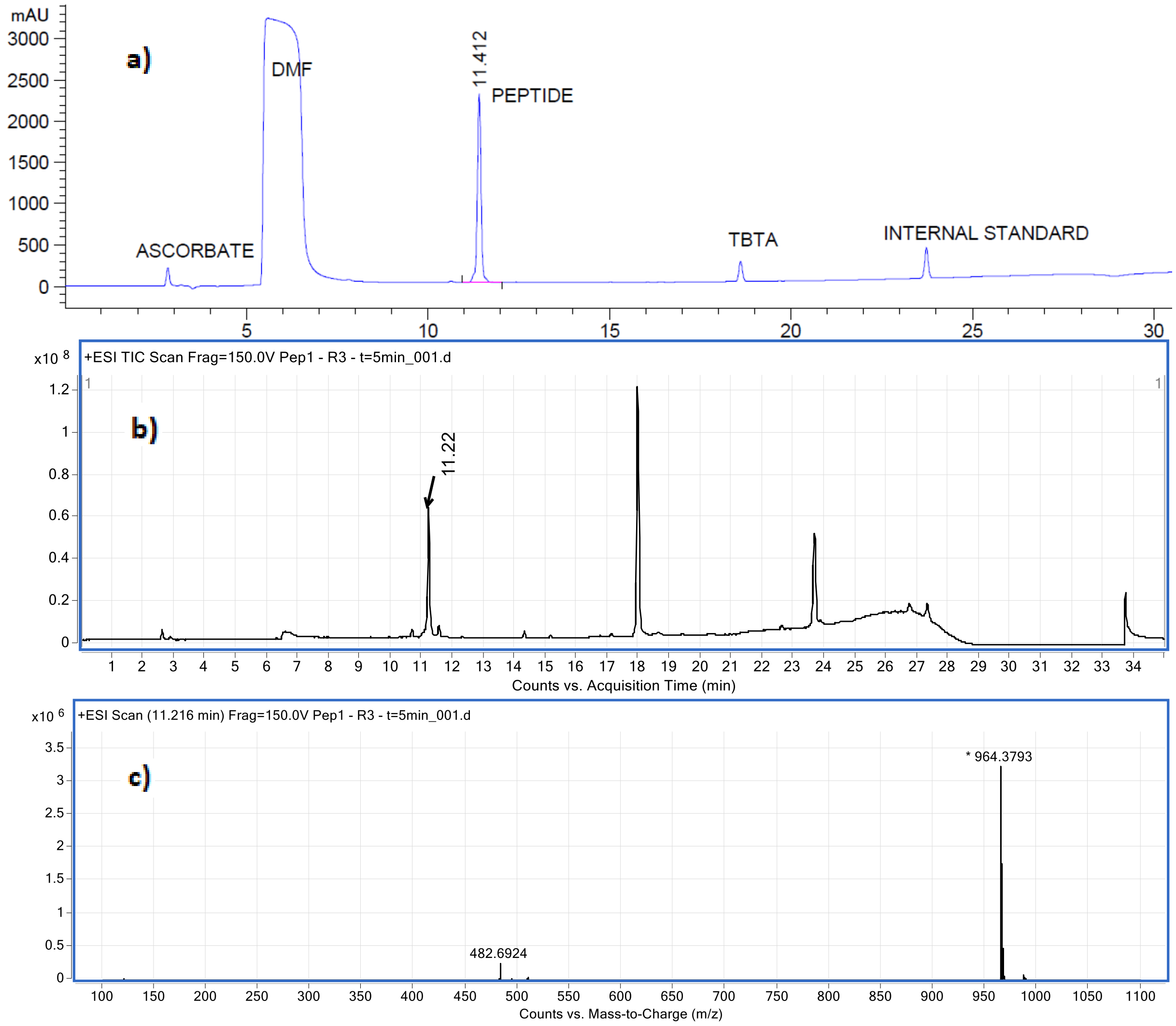

Supporting Figure S12. Reaction on peptide **1aa**, Conditions 1, aliquote was taken at reaction time t=5 min: **a)** HPLC chromatogram, **b)** TIC chromatogram of HPLC-HRMS measurement, **c)** HRMS spectrum of TIC chromatogram at RT=11.22 min. Signals representative for alkyne peptide **1aa**: $[M+H]^+$ 964.3793, $[M+2H]^{2+}$ 482.6924; calculated 964.3795 for $C_{43}H_{54}N_{11}O_{15}^+$ $[M+H]^+$.

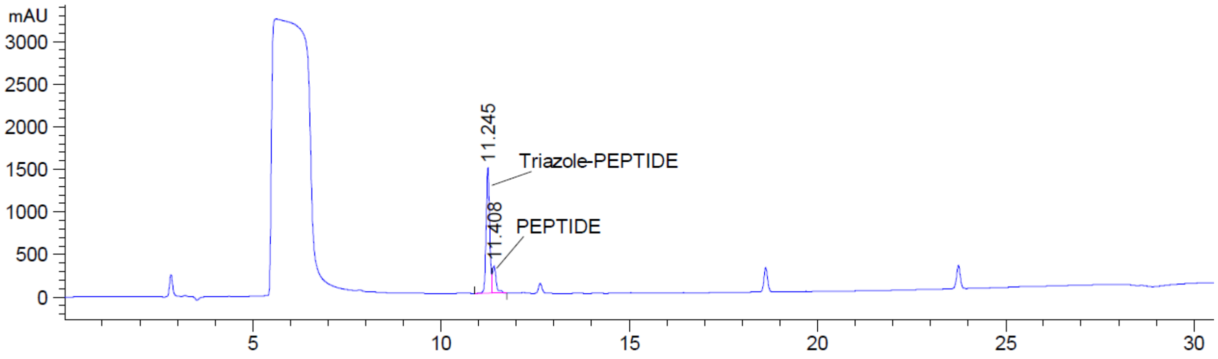

Supporting Figure S13. Reaction on peptide **1aa**, Conditions 1, aliquote was taken at reaction time t=1 h. HPLC chromatogram.

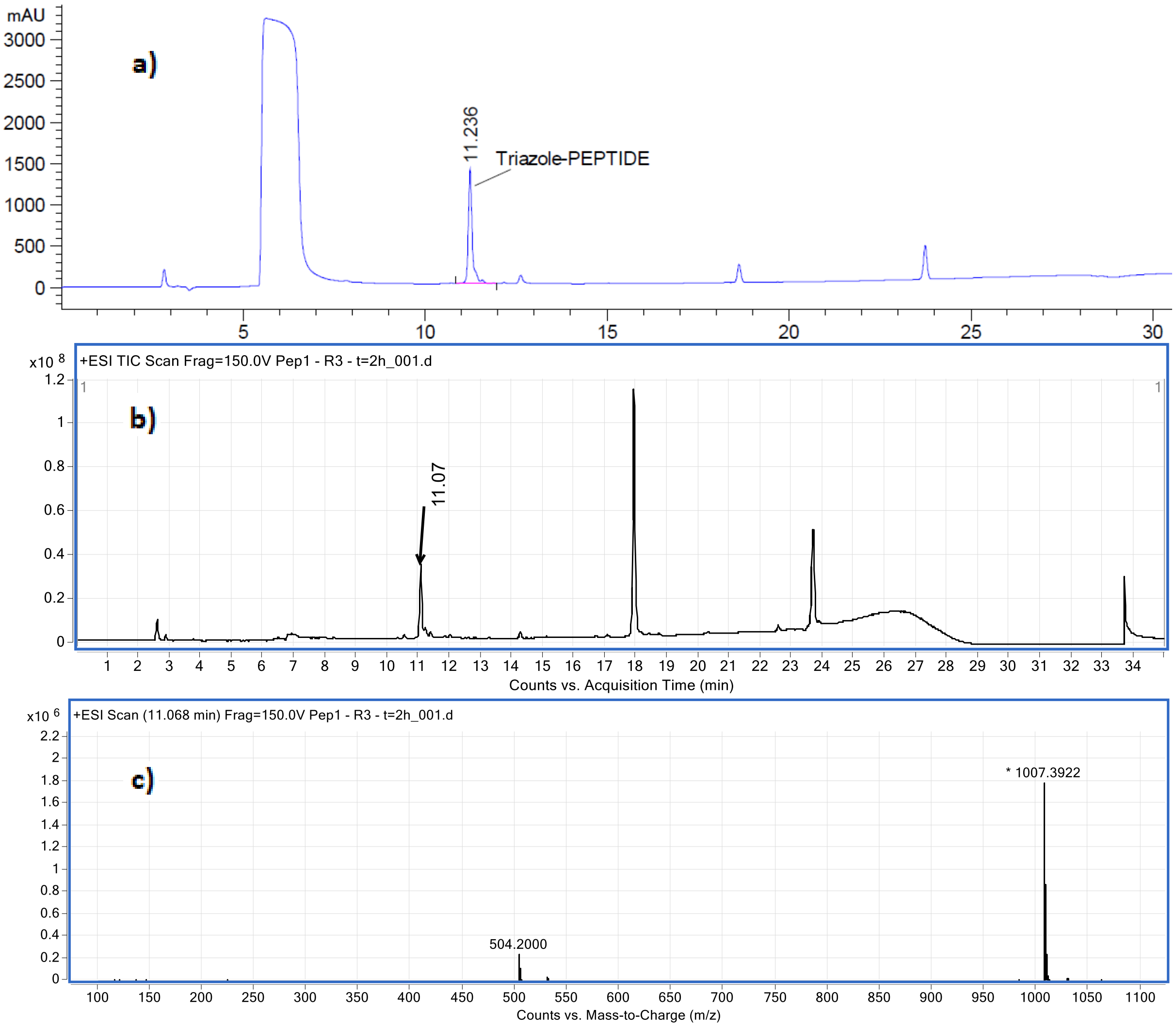

Supporting Figure S14. Reaction on peptide **1aa**, Conditions 1, aliquote was taken at reaction time t=2 h: **a)** HPLC chromatogram, **b)** TIC chromatogram of HPLC-HRMS measurement, **c)** HRMS spectrum of TIC chromatogram at RT=11.07 min. Signals representative for triazole peptide **3aa** product: $[M+H]^+$ 1007.3922, $[M+2H]^{2+}$ 504.2000; calculated 1007.3966 for $C_{43}H_{55}N_{14}O_{15}^+$ $[M+H]^+$.

Reaction on peptide 1y, Conditions 1:

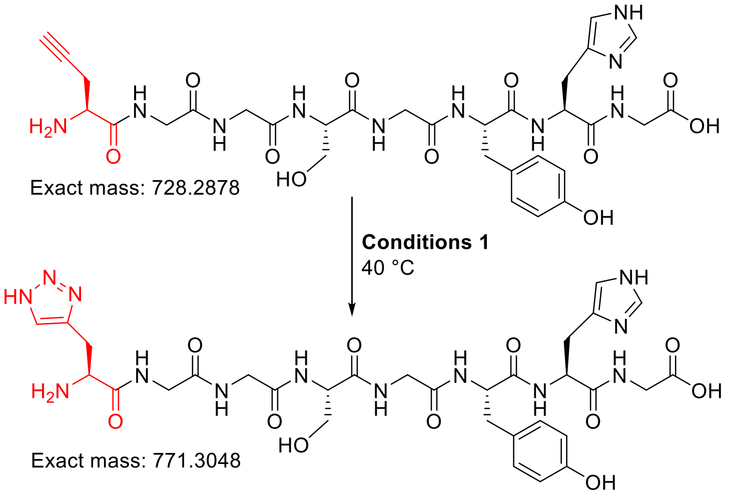

1y

3y

Peptide stock solution (DMF:H_2O 1:1, v/v):
- 250 mM peptide **1y**

Reagents stock solution (DMF):
- 100 mM TBTA
- 100 mM CuI
- 200 mM Na(asc)
- 1.0 M HCOOH
- 100 mM internal standard

Sodium azide stock solution (H_2O):
- 2.0 M NaN_3

Reaction was performed in a 200 µL Eppendorf tube. Stock solutions were added in the following order: 20 µL peptide stock solution, 25 µL Reagents stock solution, 5 µL sodium azide stock solution. After addition of each stock solution reaction mixture was mixed by pipetting up and down 15-times. Reaction was set at 40 °C (Eppendorf tube in oil bath).

Final concentrations of reagents were: peptide **1y** (100 mM), NaN_3 (200 mM), HCOOH (500 mM), Cu (50 mM), TBTA (50 mM), Na(asc) (100 mM), DMF:H_2O 7:3 (v/v).

For analysis 5 µL of reaction mixture were taken, dilluted with 250 µL DMF + 250 µL H_2O and analyzed with HPLC and HPLC-HRMS.

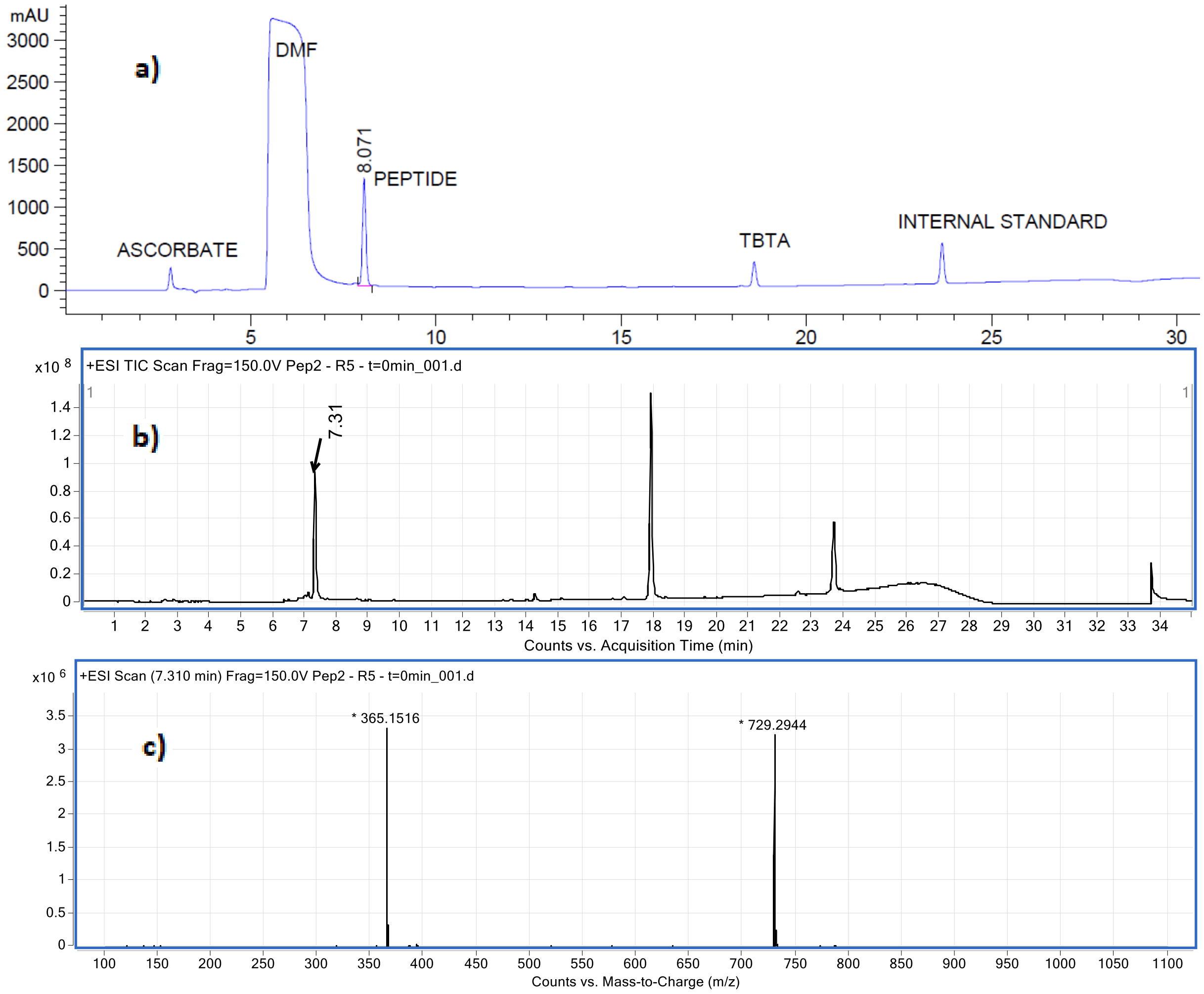

Supporting Figure S15. Reaction on peptide **1y**, Conditions 1, aliquote was taken at reaction time t=0: **a)** HPLC chromatogram, **b)** TIC chromatogram of HPLC-HRMS measurement, **c)** HRMS spectrum of TIC chromatogram at RT=7.31 min. Signals representative for alkyne peptide **1y**: $[M+H]^+$ 729.2944, $[M+2H]^{2+}$ 365.1516; calculated 729.2951 for $C_{31}H_{41}N_{10}O_{11}^+$ $[M+H]^+$.

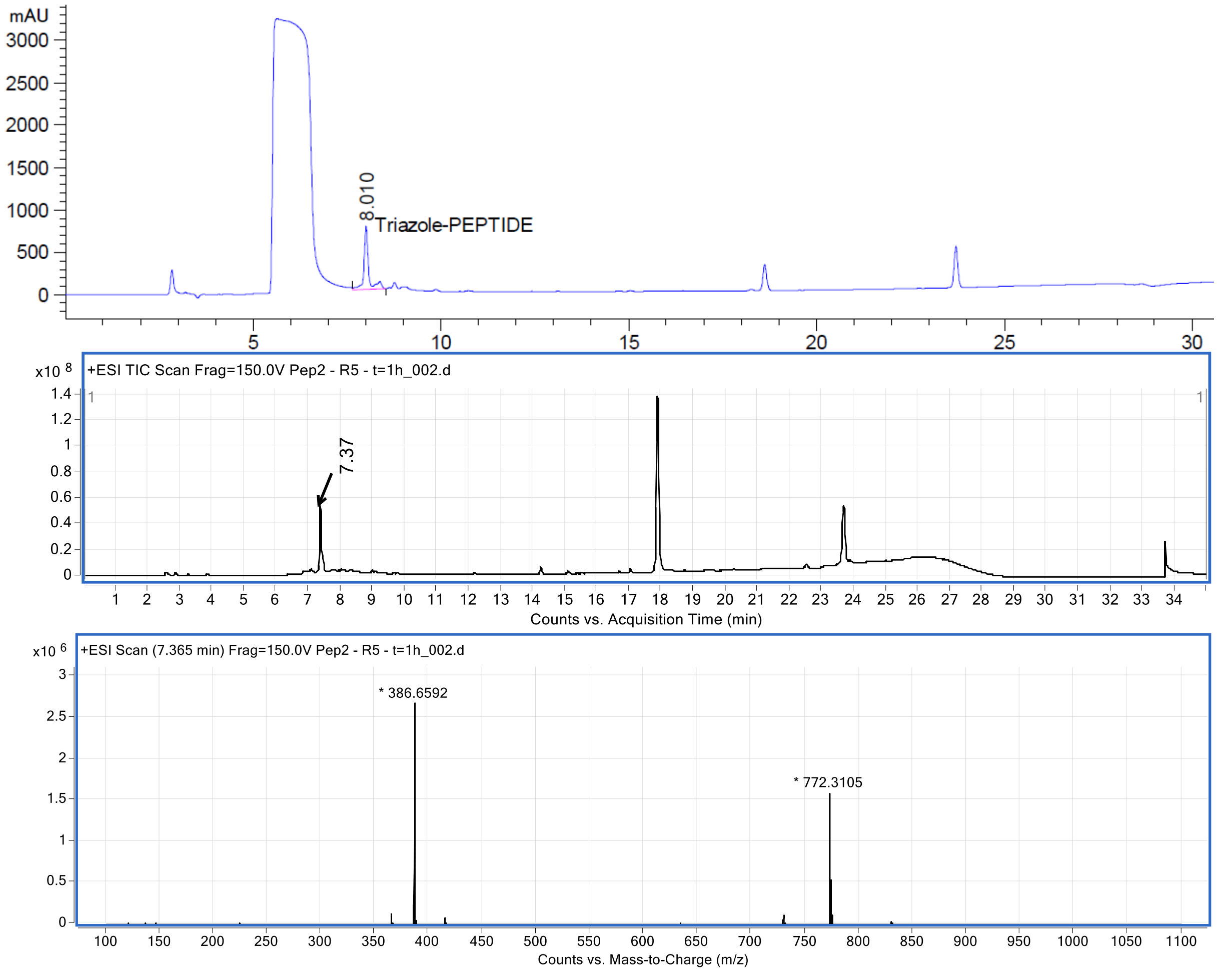

Supporting Figure S16. Reaction on peptide **1y**, conditions 1, aliquote taken at reaction time t=1 h: **a)** HPLC chromatogram, **b)** TIC chromatogram of HPLC-HRMS measurement, **c)** HRMS spectrum of TIC chromatogram at RT=7.37 min. Signals representative for triazole peptide **3y** product: $[M+H]^+$ 772.3105, $[M+2H]^{2+}$ 386.6592; calculated 772.3121 for $C_{31}H_{42}N_{13}O_{11}^+$ $[M+H]^+$.

Reaction on peptide 1z, Conditions 1:

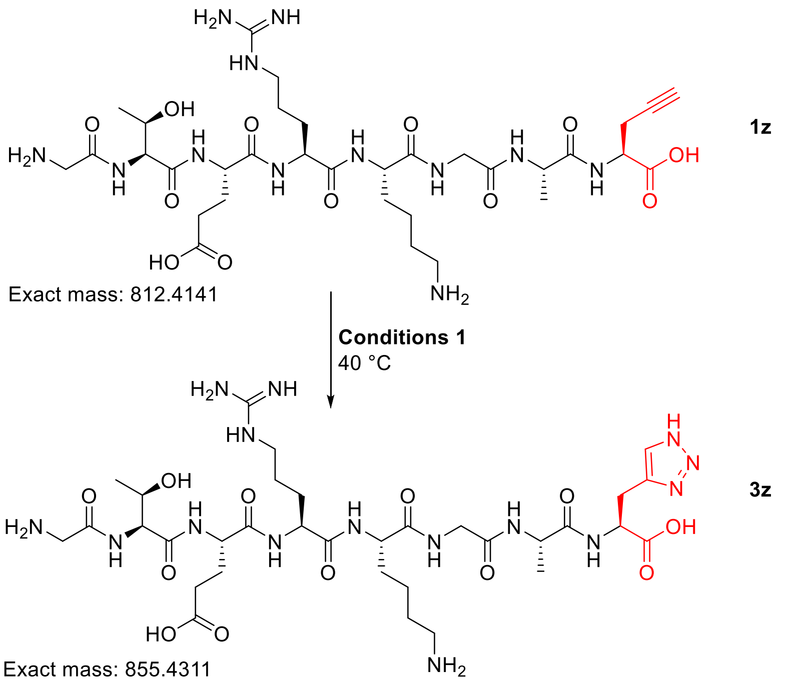

Peptide stock solution (DMF/H_2O; 1/1):
- 250 mM peptide **1z**

Reagents stock solution (DMF):
- 100 mM TBTA
- 100 mM CuI
- 200 mM Na(asc)
- 1.0 M HCOOH
- 100 mM internal standard

Sodium azide stock solution (H_2O):
- 2.0 M NaN_3

Reaction was performed in a 200 µL Eppendorf tube. Stock solutions were added in the following order: 20 µL peptide stock solution, 25 µL Reagents stock solution, 5 µL sodium azide stock solution. After addition of each stock solution reaction mixture was mixed by pipetting up and down 15-times. Reaction was set at 40 °C (Eppendorf tube in oil bath).

Final concentrations of reagents were: peptide **1z** (100 mM), NaN_3 (200 mM), HCOOH (500 mM), Cu (50 mM), TBTA (50 mM), Na(asc) (100 mM), DMF:H_2O 7:3 (v/v).

For analysis 5 µL of reaction mixture were taken, dilluted with 250 µL DMF + 250 µL H_2O and analyzed with HPLC and HPLC-HRMS.

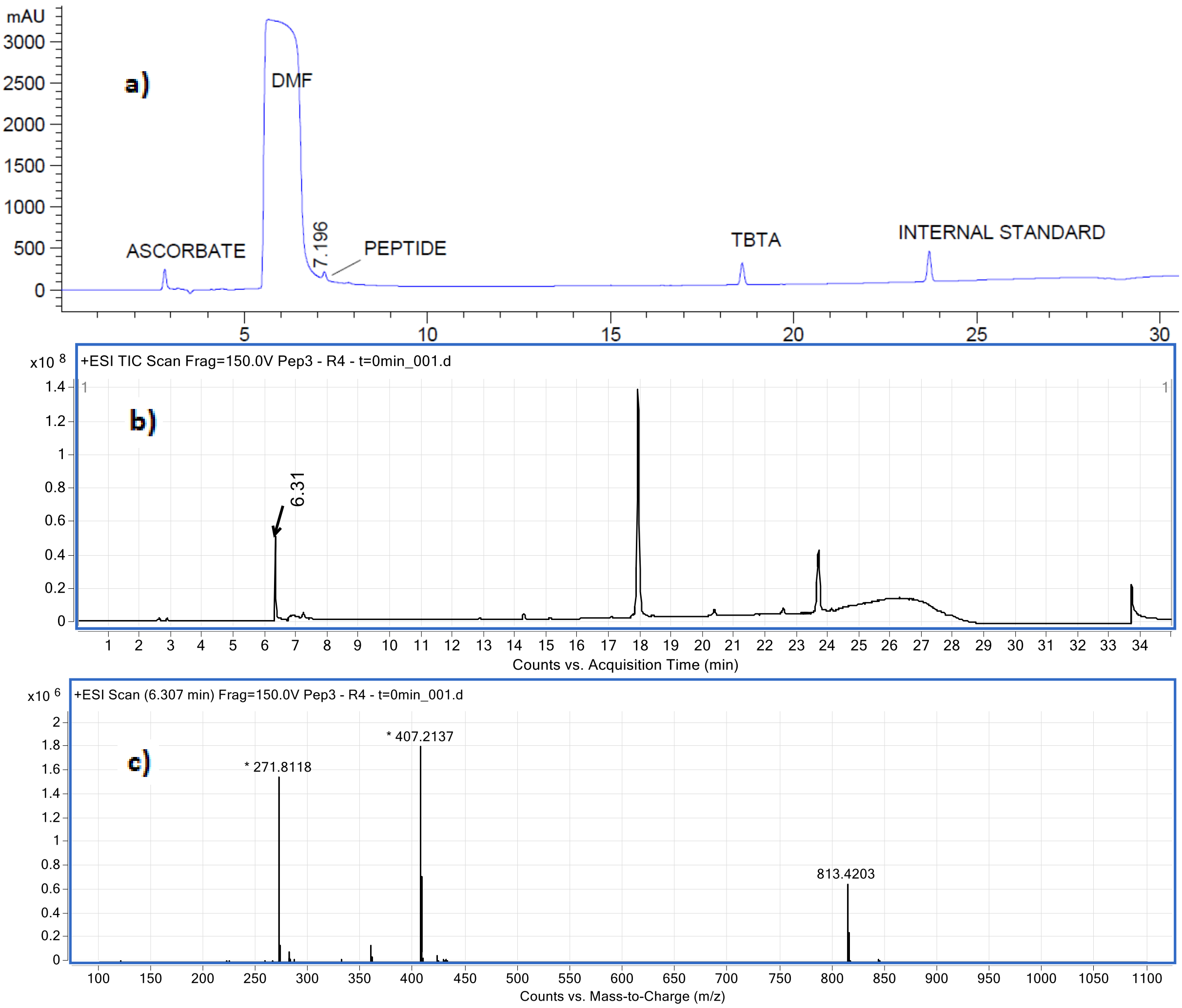

Supporting Figure S17. Reaction on peptide **1z**, Conditions 1, aliquote was taken at reaction time t=0: **a)** HPLC chromatogram, **b)** TIC chromatogram of HPLC-HRMS measurement, **c)** HRMS spectrum of TIC chromatogram at RT=6.31 min. Signals representative for alkyne peptide **1z**: $[M+H]^+$ 813.4203, $[M+2H]^{2+}$ 407.2137, $[M+3H]^{3+}$ 271.8118; calculated 813.4213 for $C_{33}H_{57}N_{12}O_{12}^+$ $[M+H]^+$.

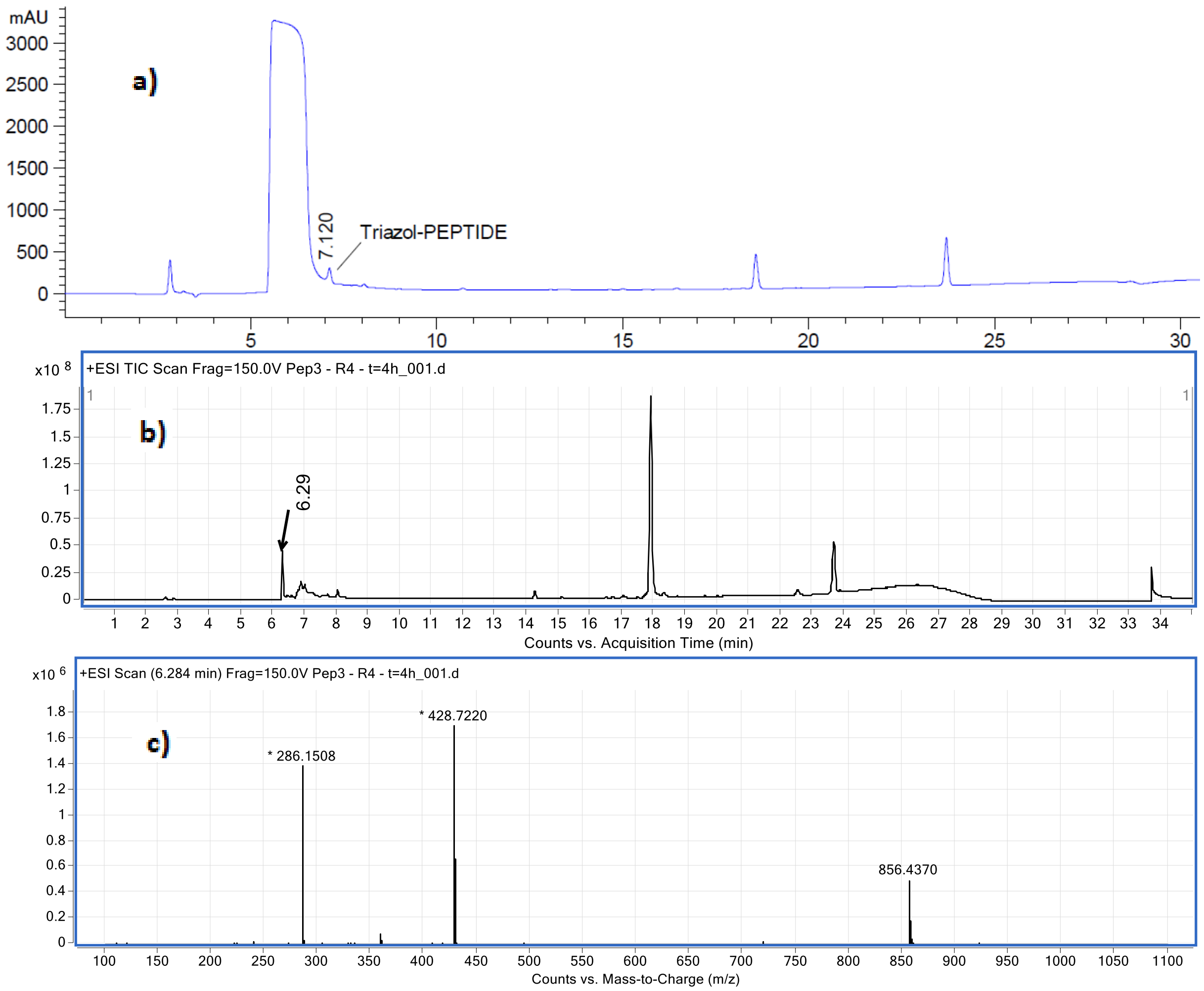

Supporting Figure S18. Reaction on peptide **1z**, Conditions 1, aliquote taken at reaction time t=4 h: **a)** HPLC chromatogram, **b)** TIC chromatogram of HPLC-HRMS measurement, **c)** HRMS spectrum of TIC chromatogram at RT=6.29 min. Signals representative for triazole peptide **3z** product: $[M+H]^+$ 856.4370, $[M+2H]^{2+}$ 428.7220, $[M+3H]^{3+}$ 286.1508; calculated 856.4384 for $C_{33}H_{58}N_{15}O_{12}^+$ $[M+H]^+$.

2.11. Calculation of hydrazoic acid concentration in solution for Method C

Method C: 50 mg PhCCH, **49 mg NaN_3**, 6.3 mg $CuSO_4 \times 5H_2O$, **190 µL CH_3COOH**, 25 mg Na(asc), 10.2 mg $(BimH)_3$, **450 µL MeOH, 150 µL H_2O**.

Assuming reaction solution consists only of NaN_3, CH_3COOH and H_2O:

m(NaN_3) = 49 mg → 0.75 mmol
V(CH_3COOH) = 190 µL → 200 mg → 3.33 mmol
V(H_2O) = 600 µL
pKa(CH_3COOH) = 4.75
pKa(HN_3) = 4.6
$K_w = 10^{-14}$

If we assume additivity of volumes:

$C_0(CH_3COOH)$ = 4.22 M
$C_0(NaN_3)$ = 0.95 M

Equilibriums:

$$HN_3 + H_2O \rightleftharpoons N_3^- + H_3O^+$$

$$CH_3COOH + H_2O \rightleftharpoons CH_3COO^- + H_3O^+$$

$$H_2O \rightleftharpoons H_3O^+ + OH^-$$

Corresponding equilibrium equations:

$$K_{(HN3)} = \frac{[N_3^-][H_3O^+]}{[HN_3]} \qquad (1)$$

$$K_{(CH_3COOH)} = \frac{[CH_3COO^-][H_3O^+]}{[CH_3COOH]} \qquad (2)$$

$$K_w = [H_3O^+][OH^-] \qquad (3)$$

Mass balances and electronegativity equation:

$$C_0(NaN_3) = [N_3^-] + [HN_3] \qquad (4)$$

$$C_0(CH_3COOH) = [CH_3COO^-] + [CH_3COOH] \qquad (5)$$

$$[Na^+] + [H_3O^+] = [CH_3COO^-] + [N_3^-] + [OH^-] \qquad (6)$$

Na^+ does not hydrolize therefore:

$$[Na^+] = C_o(NaN_3) \qquad (7)$$

By combining equations (1–7) we get the following expression:

$$[H_3O^+] = C_0(CH_3COOH) - \frac{C_0(CH_3COOH) \cdot [H_3O^+]}{K_{(CH_3COOH)} + [H_3O^+]} - \frac{C_0(NaN_3) \cdot [H_3O^+]}{K_{(HN3)} + [H_3O^+]} + \frac{K_w}{[H_3O^+]} \quad (8)$$

Using WolframAlpha we solve expression (8) for parameters given above:

$$[H_3O^+] = 8.46 \cdot 10^{-5}\ M$$

Combining equations (1) and (4) we get the following expression:

$$[HN_3] = \frac{C_o(NaN_3) \cdot [H_3O^+]}{K_{(HN3)} + [H_3O^+]} \quad (9)$$

Using equation (9) to calculate equilibrium concentration of hydrazoic acid:

$$[HN_3] = 0.733\ M$$

Which corresponds to:

$$w/w = 3.11\ \%$$

3. COPIES OF NMR SPECTRA

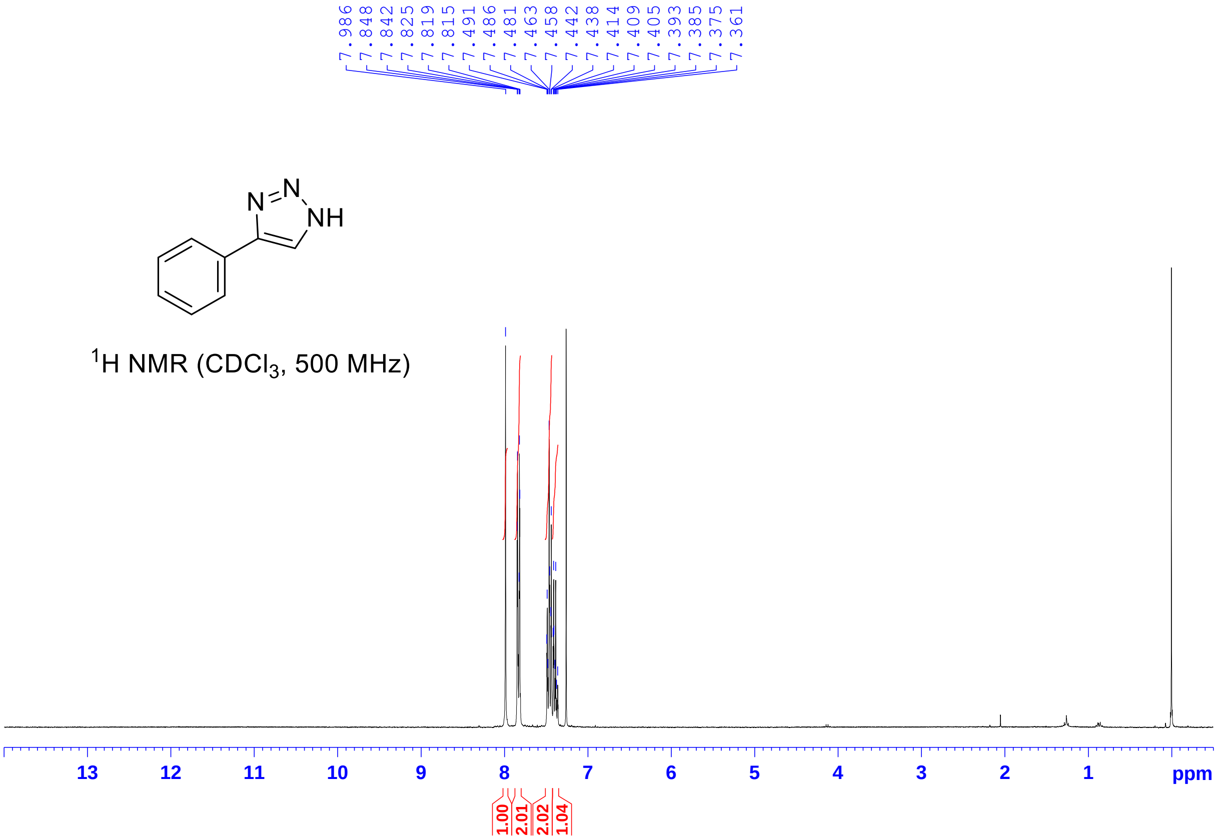

Supporting Figure S19. ^{1}H NMR spectrum of compound **3a** in $CDCl_3$, 500 MHz.

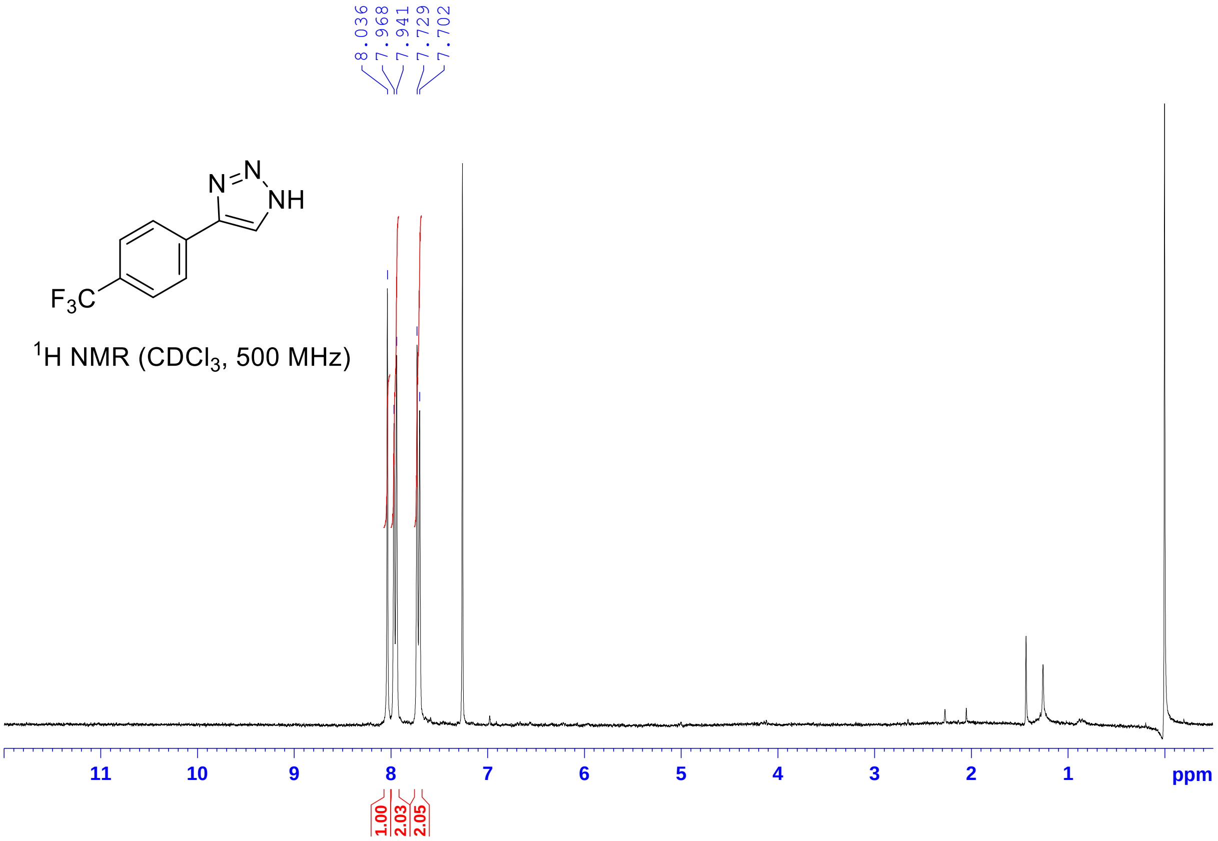

Supporting Figure S20. ^{1}H NMR spectrum of compound **3b** in $CDCl_3$, 500 MHz.

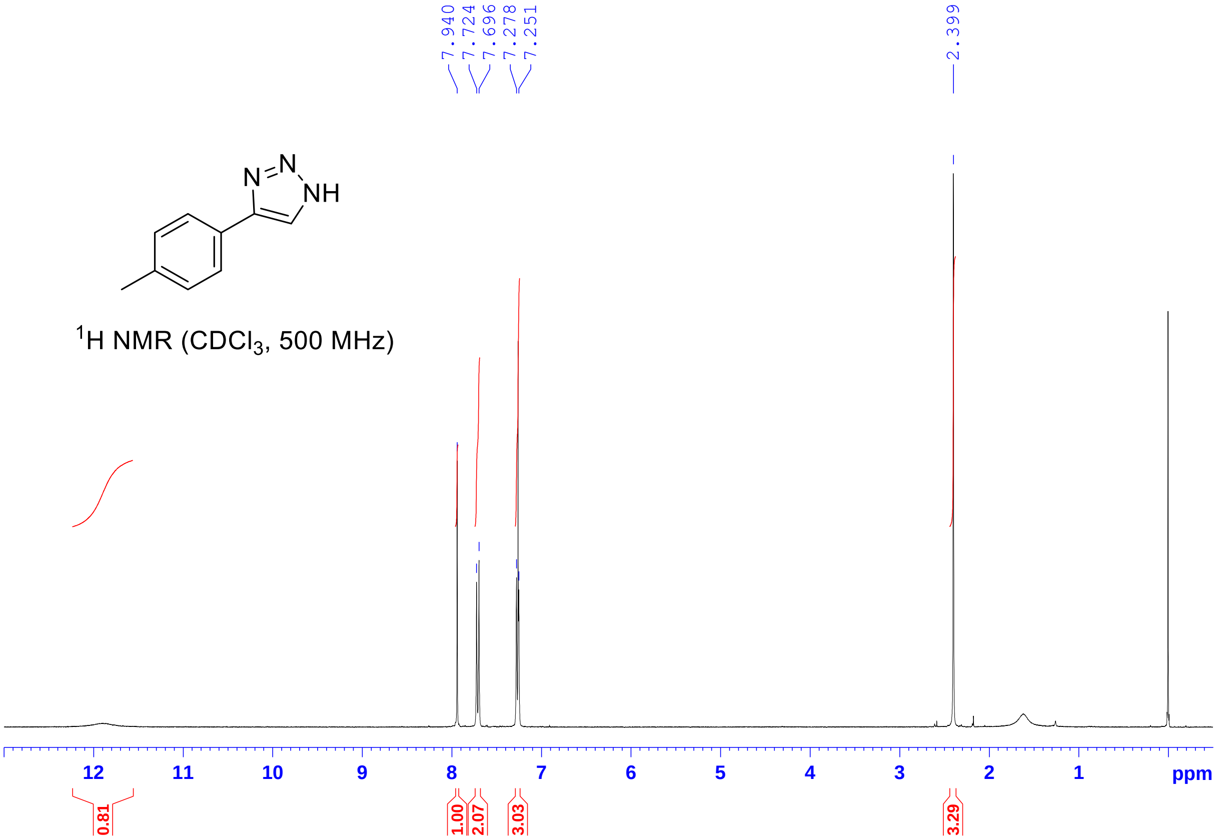

Supporting Figure S21. ^{1}H NMR spectrum of compound **3c** in $CDCl_3$, 500 MHz.

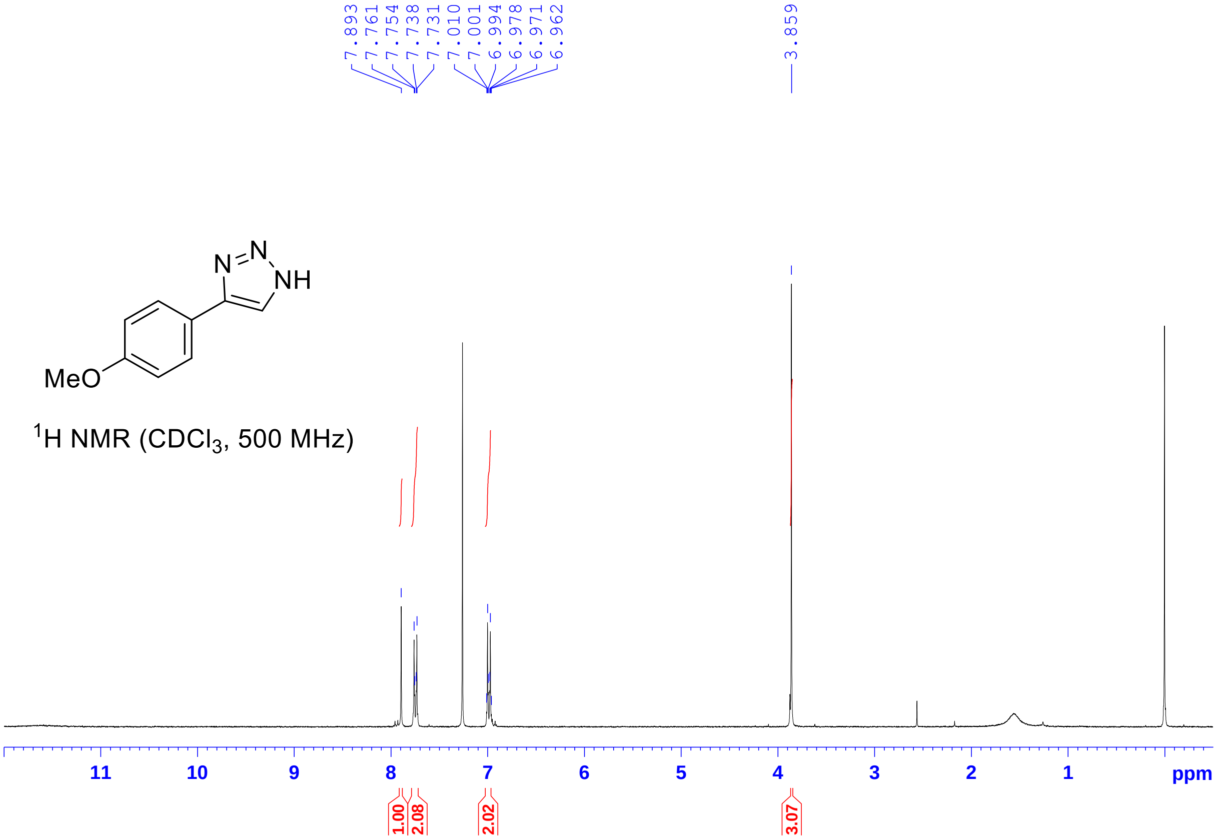

Supporting Figure S22. ^{1}H NMR spectrum of compound **3d** in $CDCl_3$, 500 MHz.

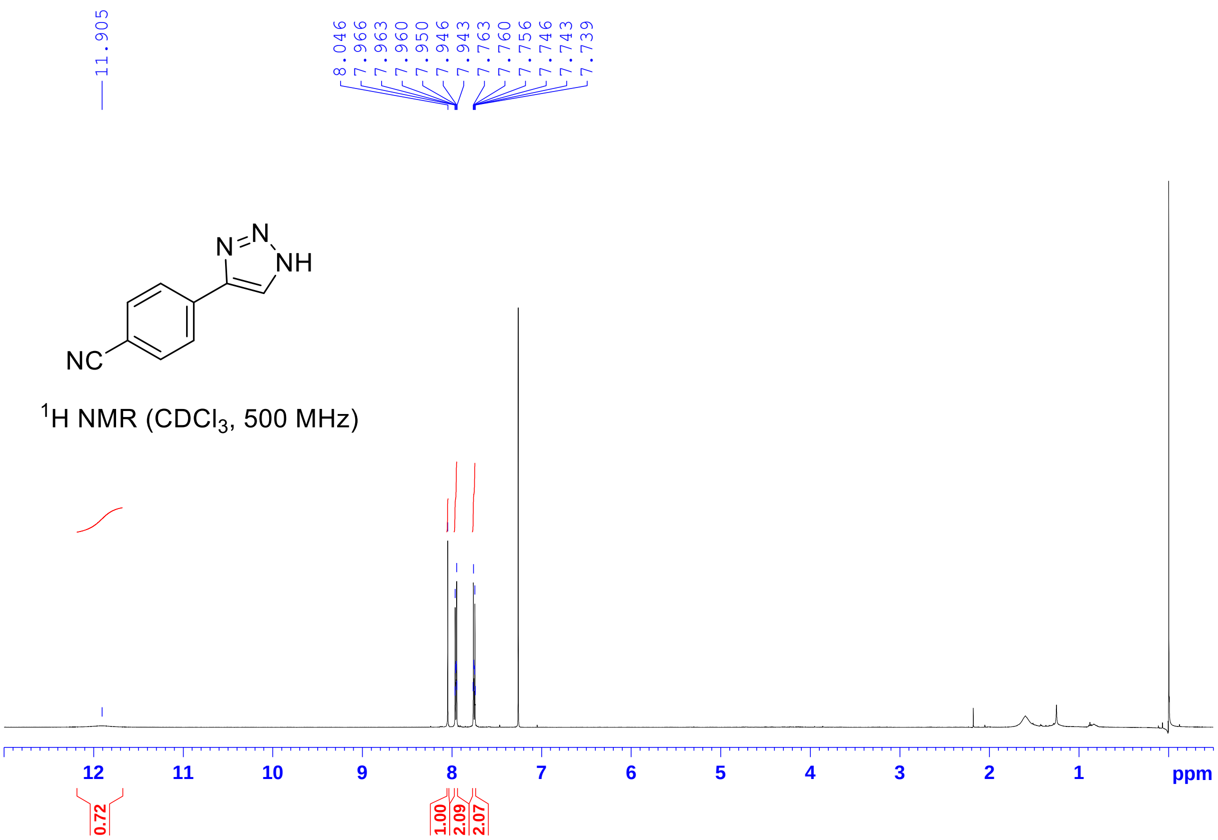

Supporting Figure S23. ^{1}H NMR spectrum of compound **3e** in $CDCl_3$, 500 MHz.

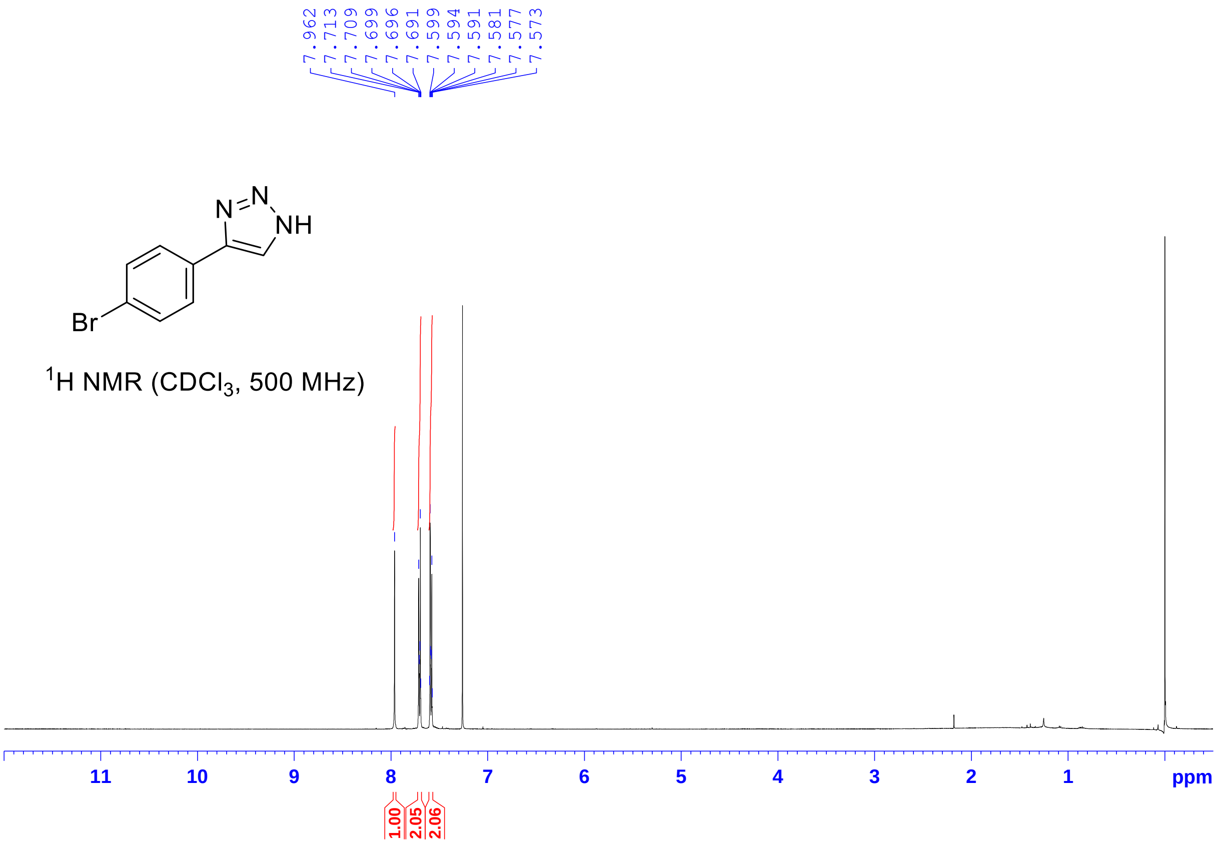

Supporting Figure S24. ^{1}H NMR spectrum of compound **3f** in $CDCl_3$, 500 MHz.

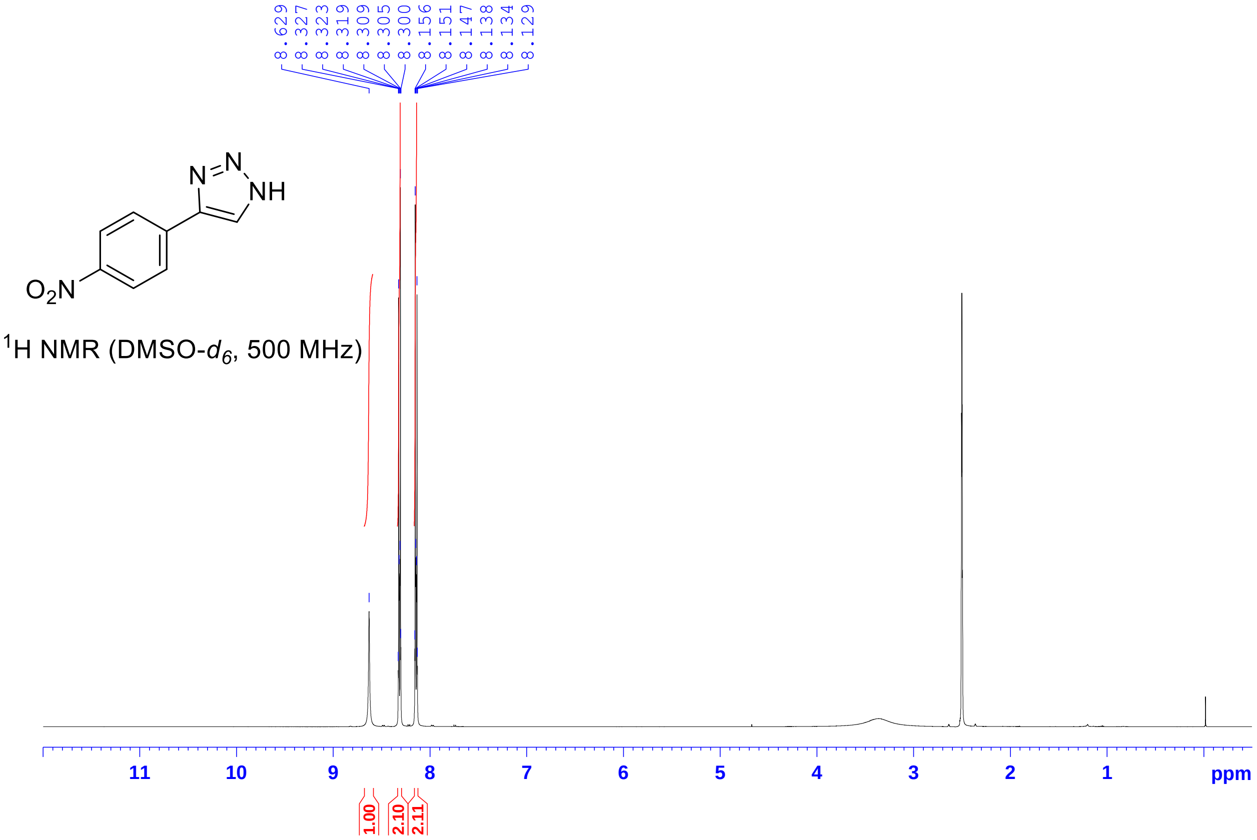

Supporting Figure S25. ^{1}H NMR spectrum of compound **3g** in DMSO-d_6, 500 MHz.

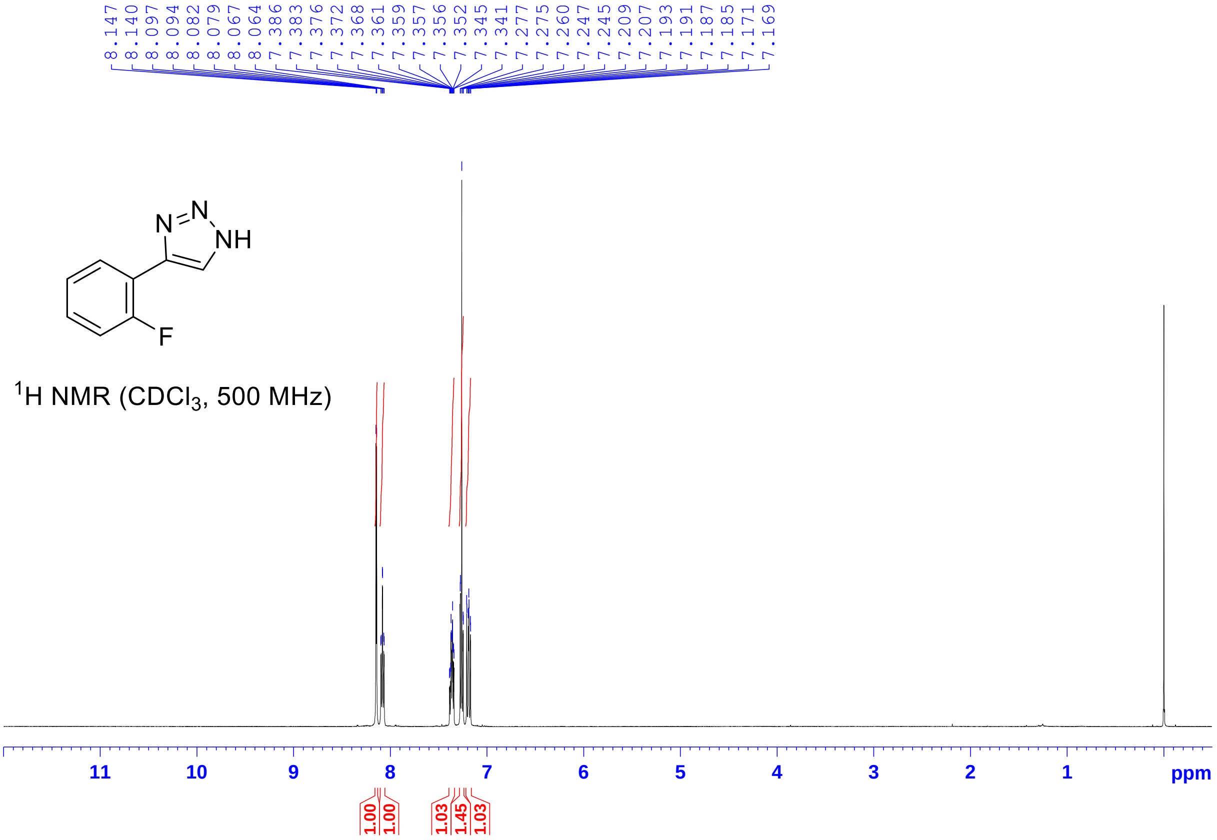

Supporting Figure S26. ^{1}H NMR spectrum of compound **3h** in $CDCl_3$, 500 MHz.

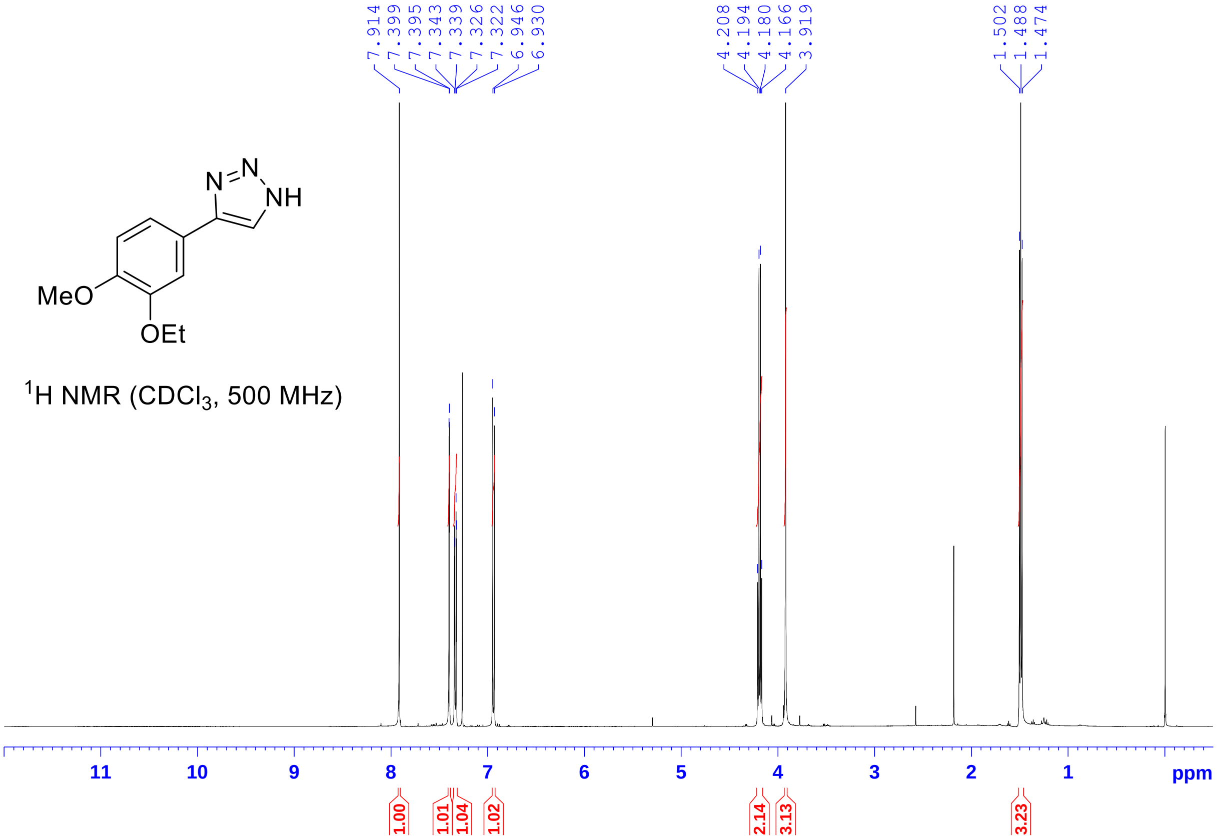

Supporting Figure S27. ^{1}H NMR spectrum of compound **3i** in $CDCl_3$, 500 MHz. Signal at δ 2.18 is from residual acetone.

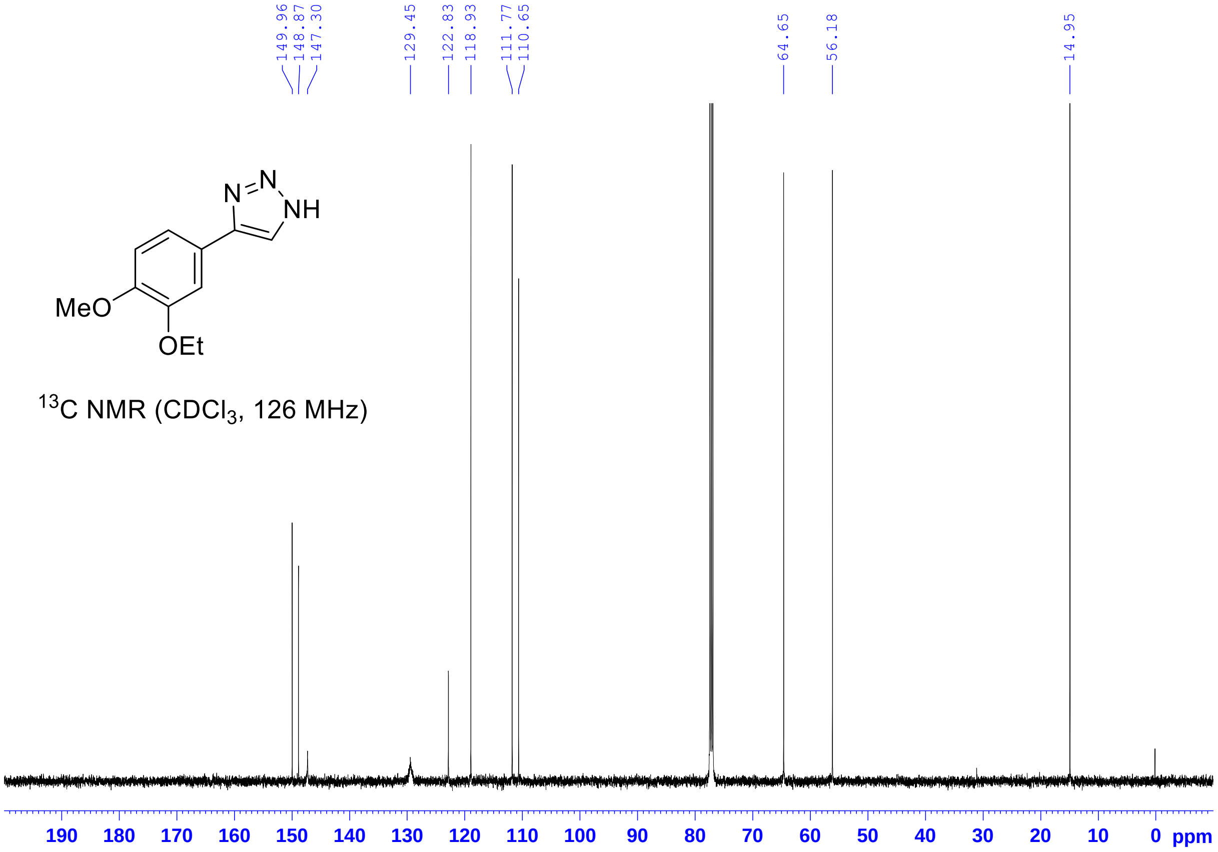

Supporting Figure S28. ^{13}C{^{1}H} NMR spectrum of compound **3i** in $CDCl_3$, 126 MHz.

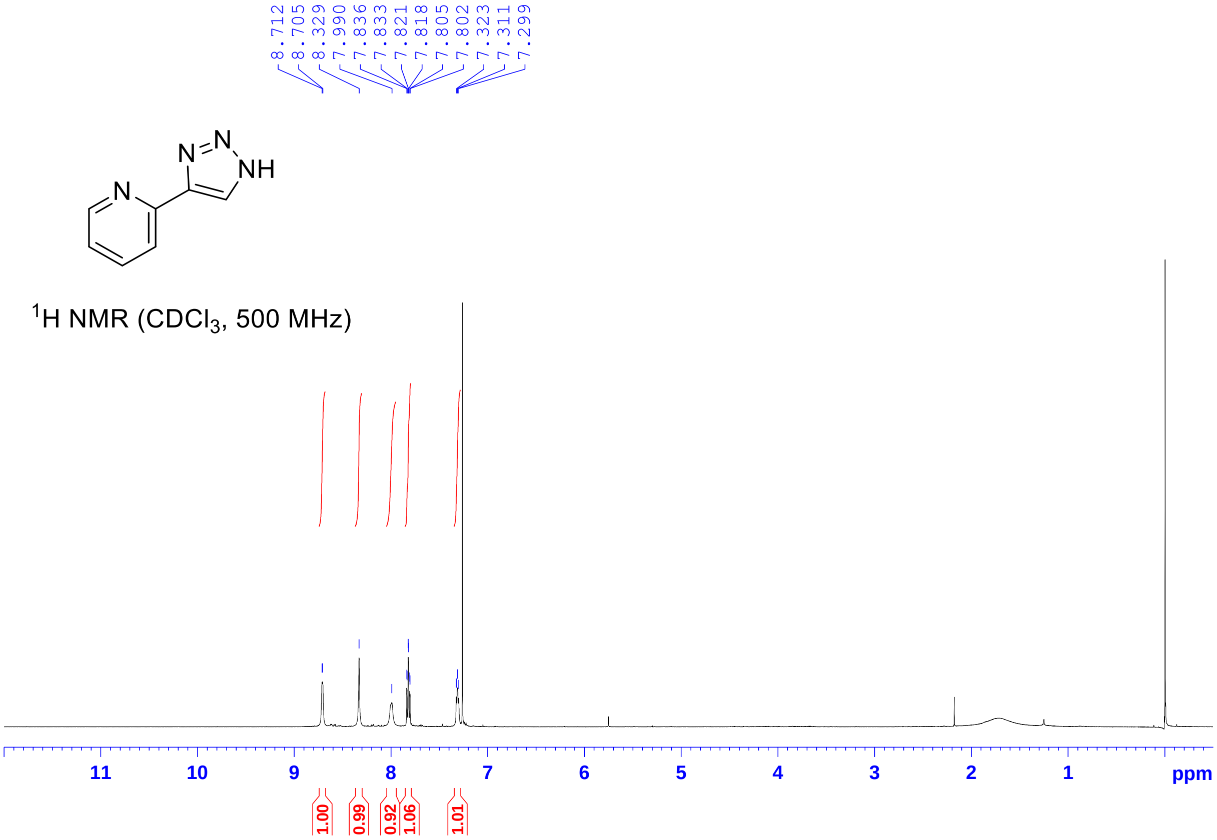

Supporting Figure S29. ^{1}H NMR spectrum of compound **3k** in $CDCl_3$, 500 MHz. Signal at δ 2.18 is from residual acetone.

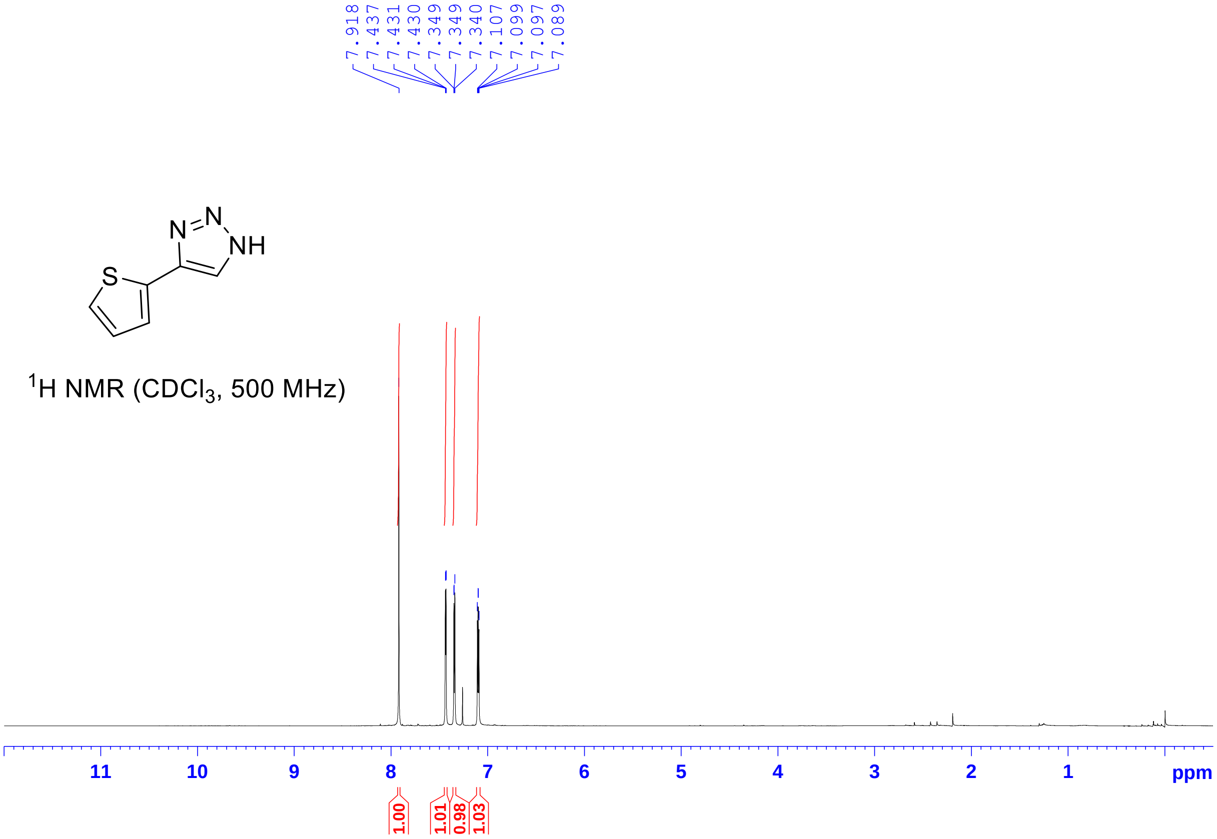

Supporting Figure S30. ^{1}H NMR spectrum of compound **3l** in $CDCl_3$, 500 MHz.

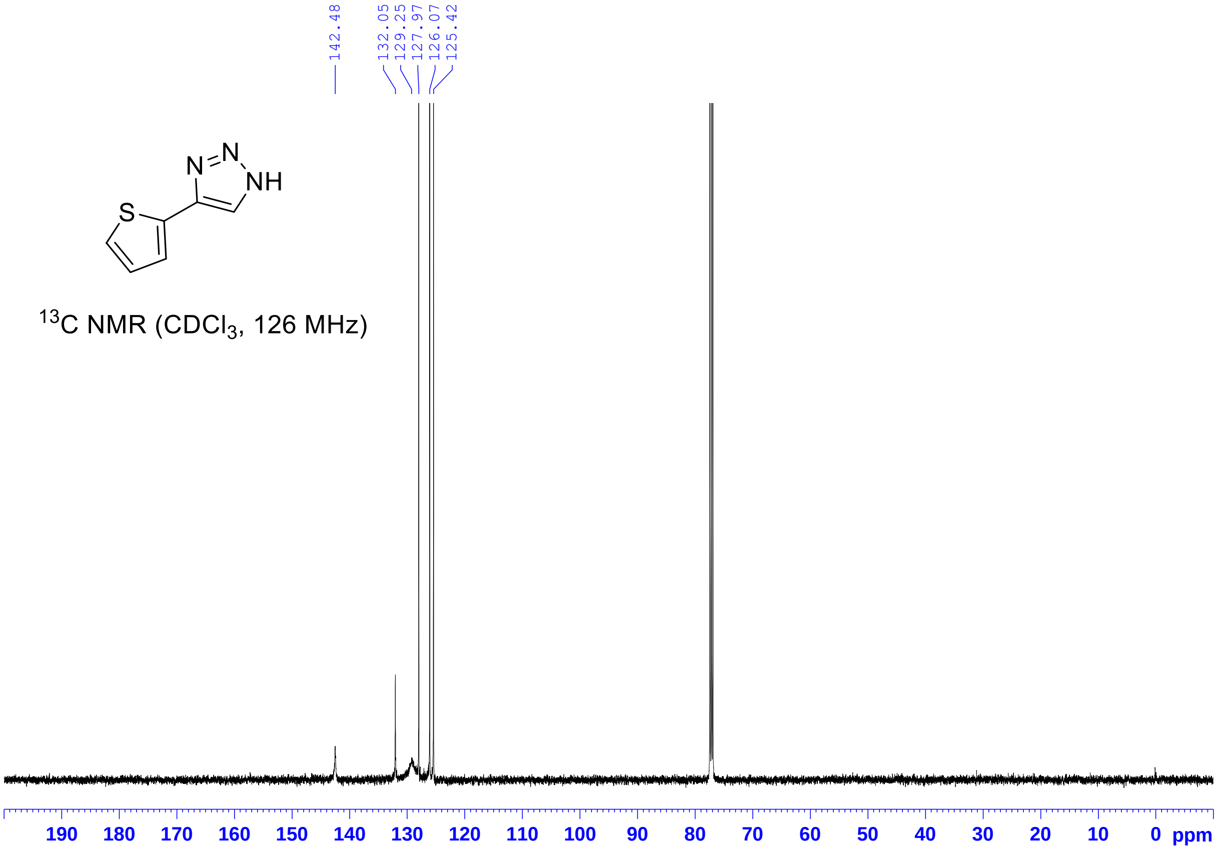

Supporting Figure S31. $^{13}C\{^{1}H\}$ NMR spectrum of compound **3l** in $CDCl_3$, 126 MHz.

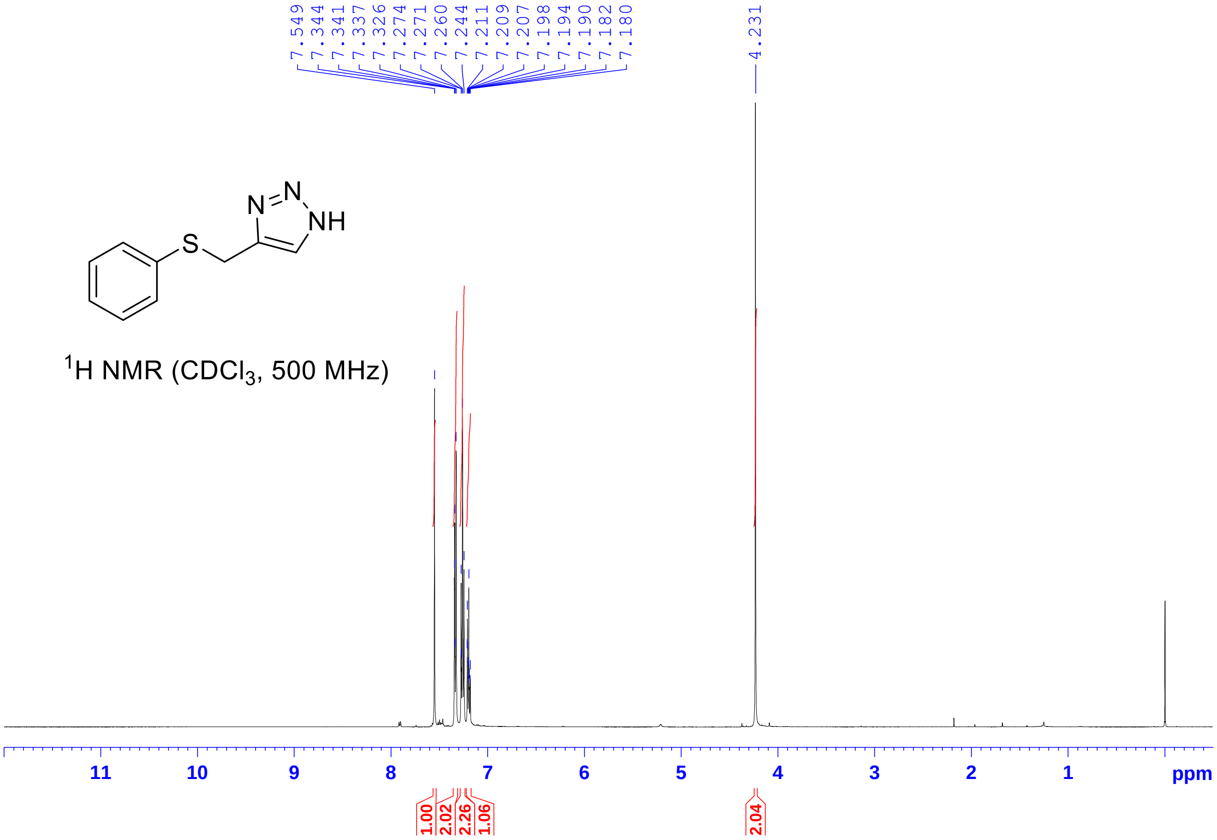

Supporting Figure S32. ^{1}H NMR spectrum of compound **3m** in $CDCl_3$, 500 MHz.

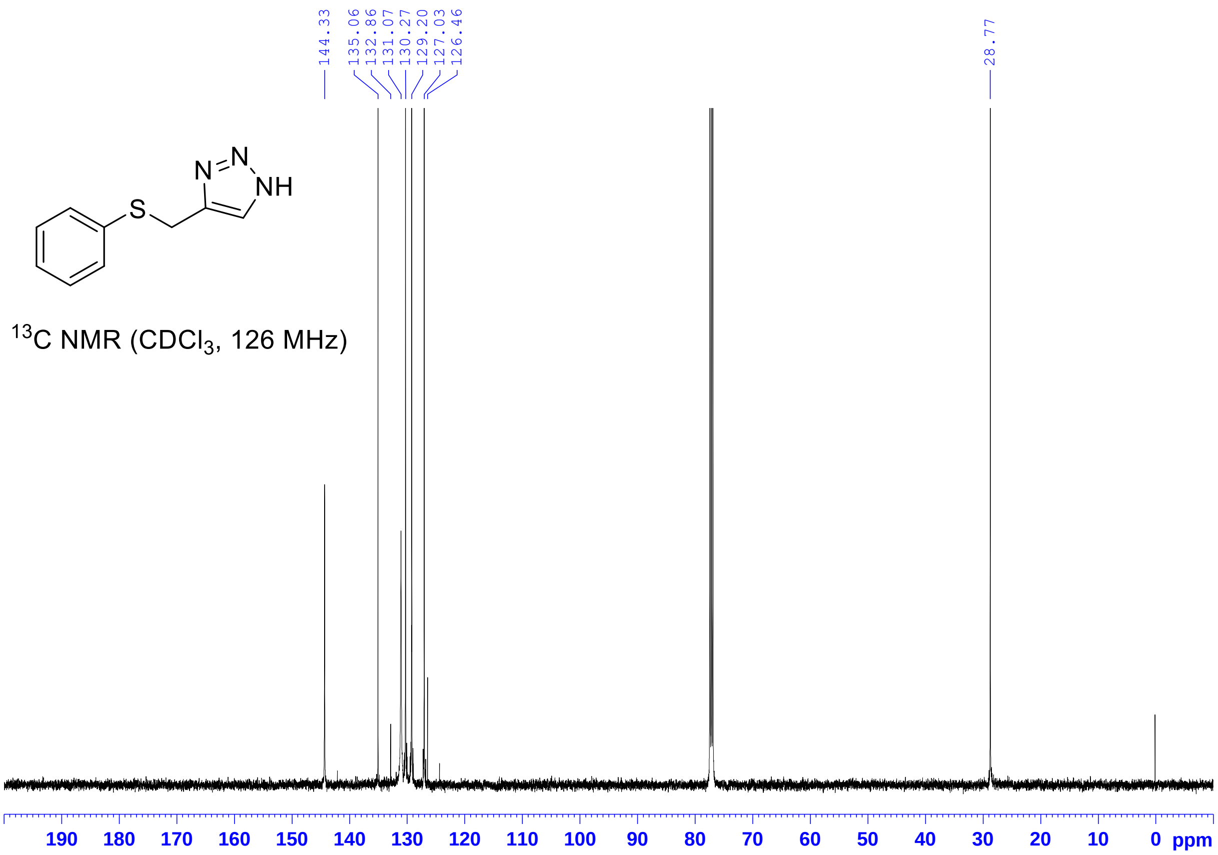

Supporting Figure S33. $^{13}C\{^{1}H\}$ NMR spectrum of compound **3m** in $CDCl_3$, 126 MHz.

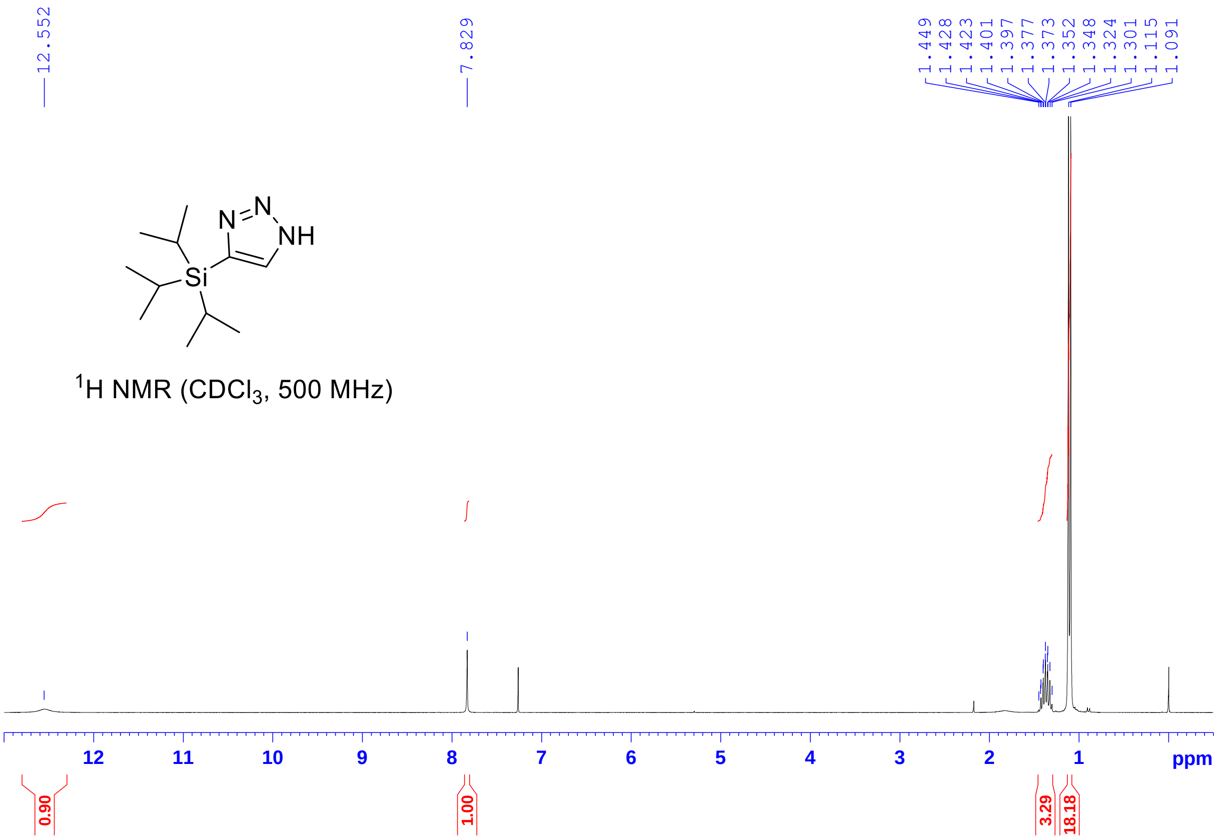

Supporting Figure S34. ^{1}H NMR spectrum of compound **3n** in $CDCl_3$, 500 MHz.

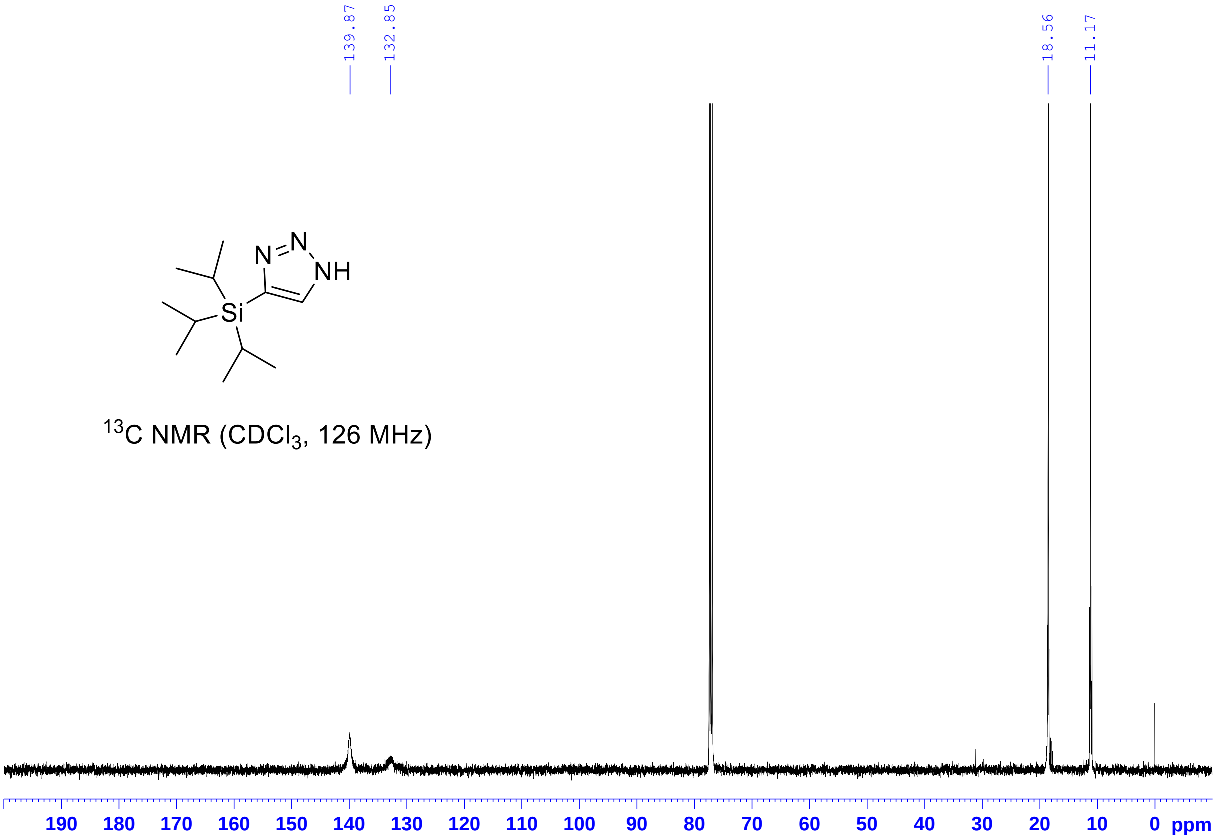

Supporting Figure S35. $^{13}C\{^1H\}$ NMR spectrum of compound **3n** in $CDCl_3$, 126 MHz.

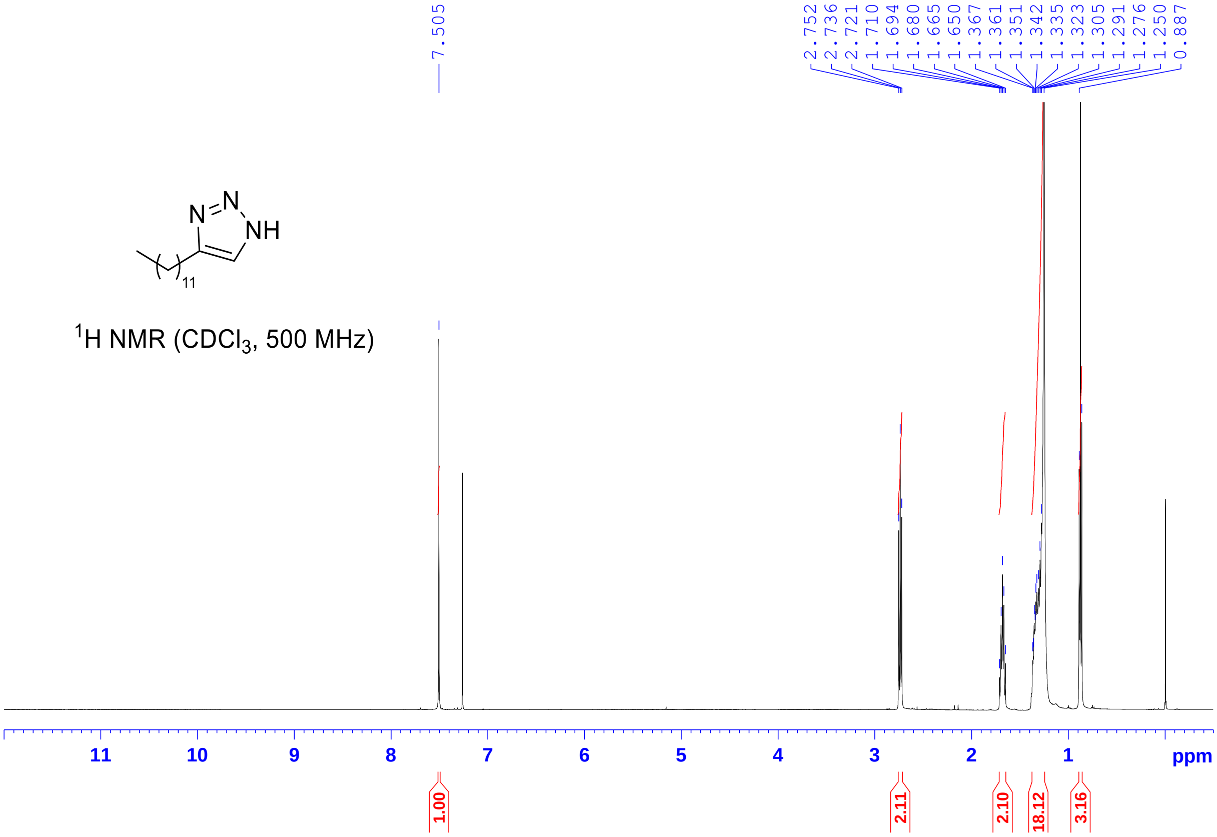

Supporting Figure S36. ^1H NMR spectrum of compound **3o** in $CDCl_3$, 500 MHz.

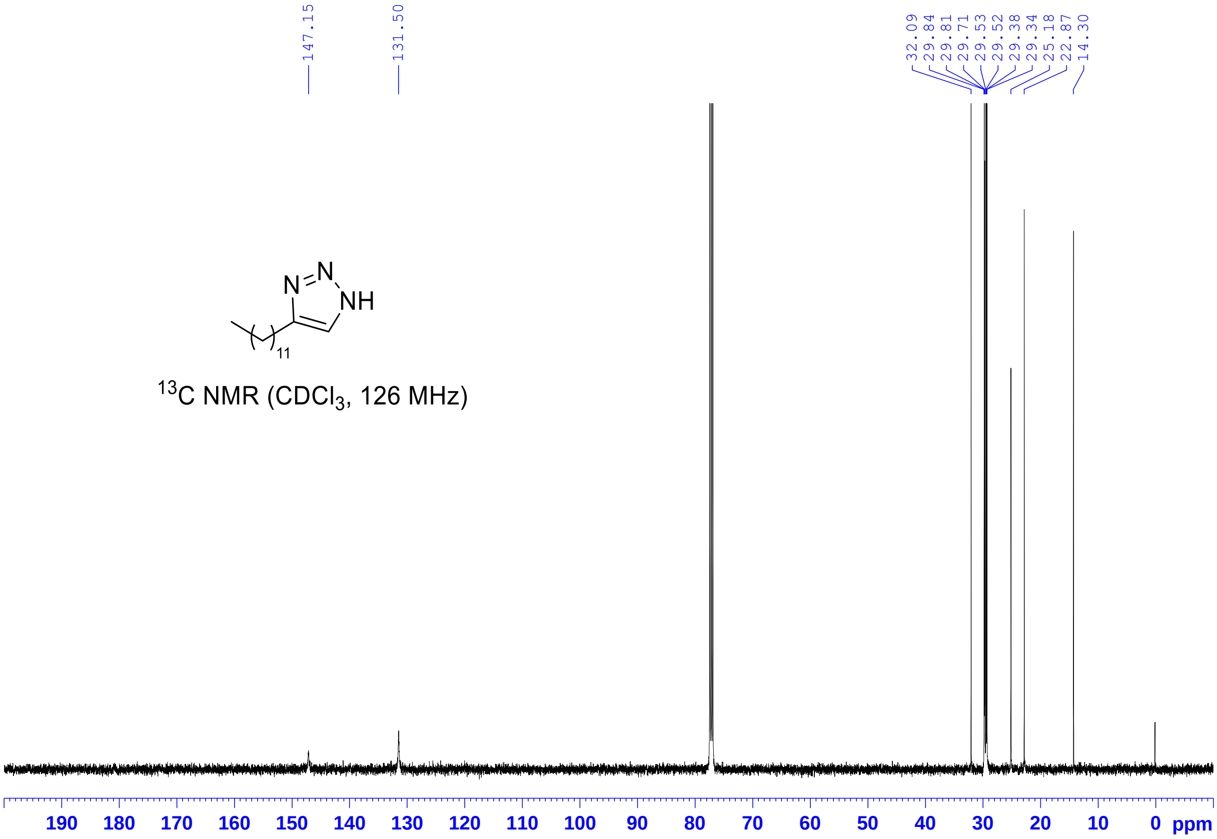

Supporting Figure S37. ^{13}C{^{1}H} NMR spectrum of compound **3o** in CDCl$_3$, 126 MHz.

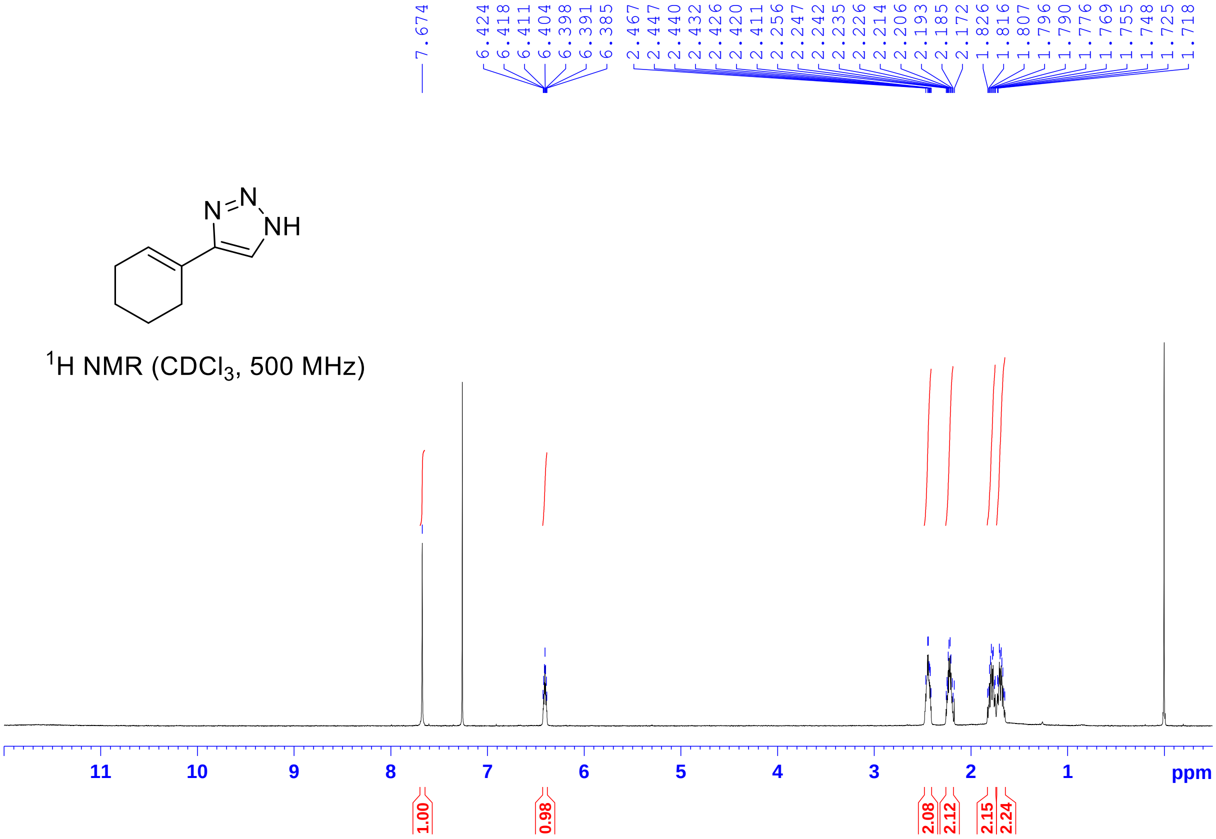

Supporting Figure S38. ^{1}H NMR spectrum of compound **3p** in CDCl$_3$, 500 MHz.

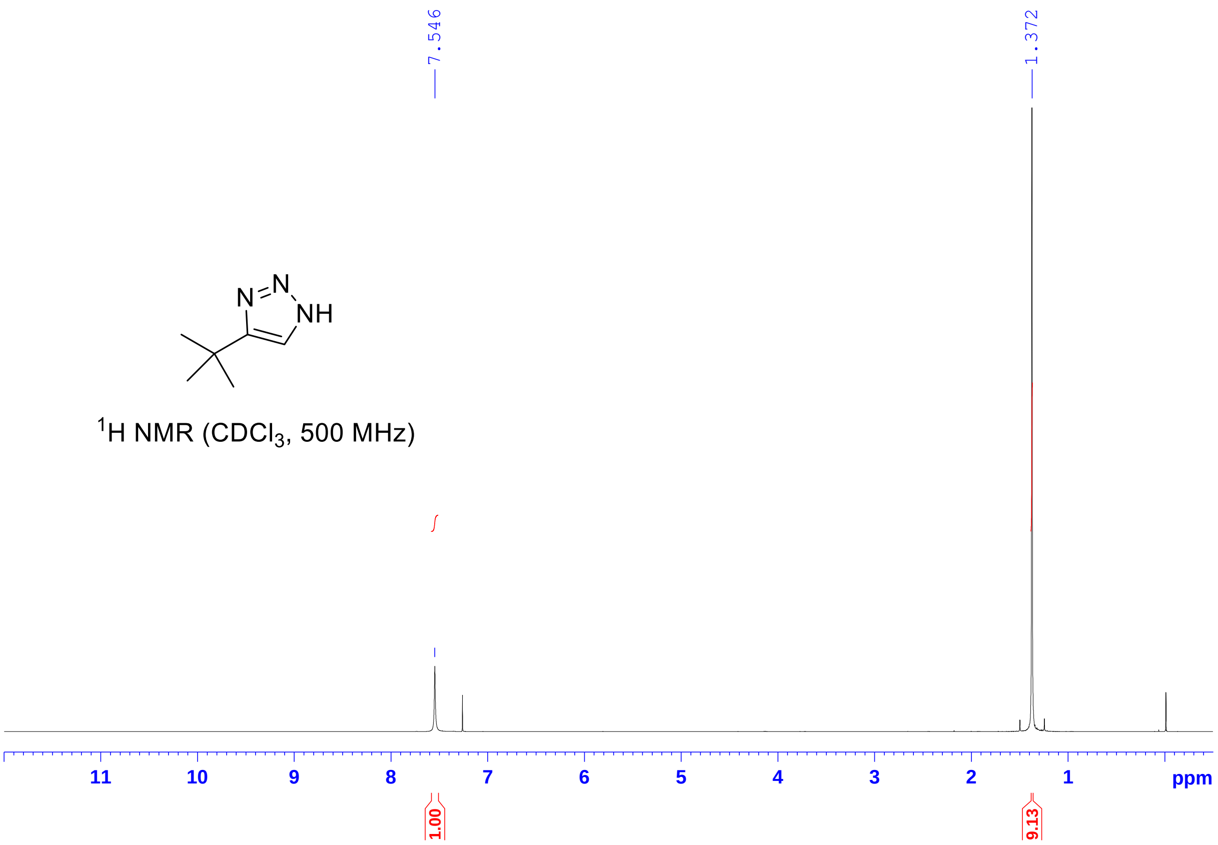

Supporting Figure S39. ^{1}H NMR spectrum of compound **3q** in $CDCl_3$, 500 MHz.

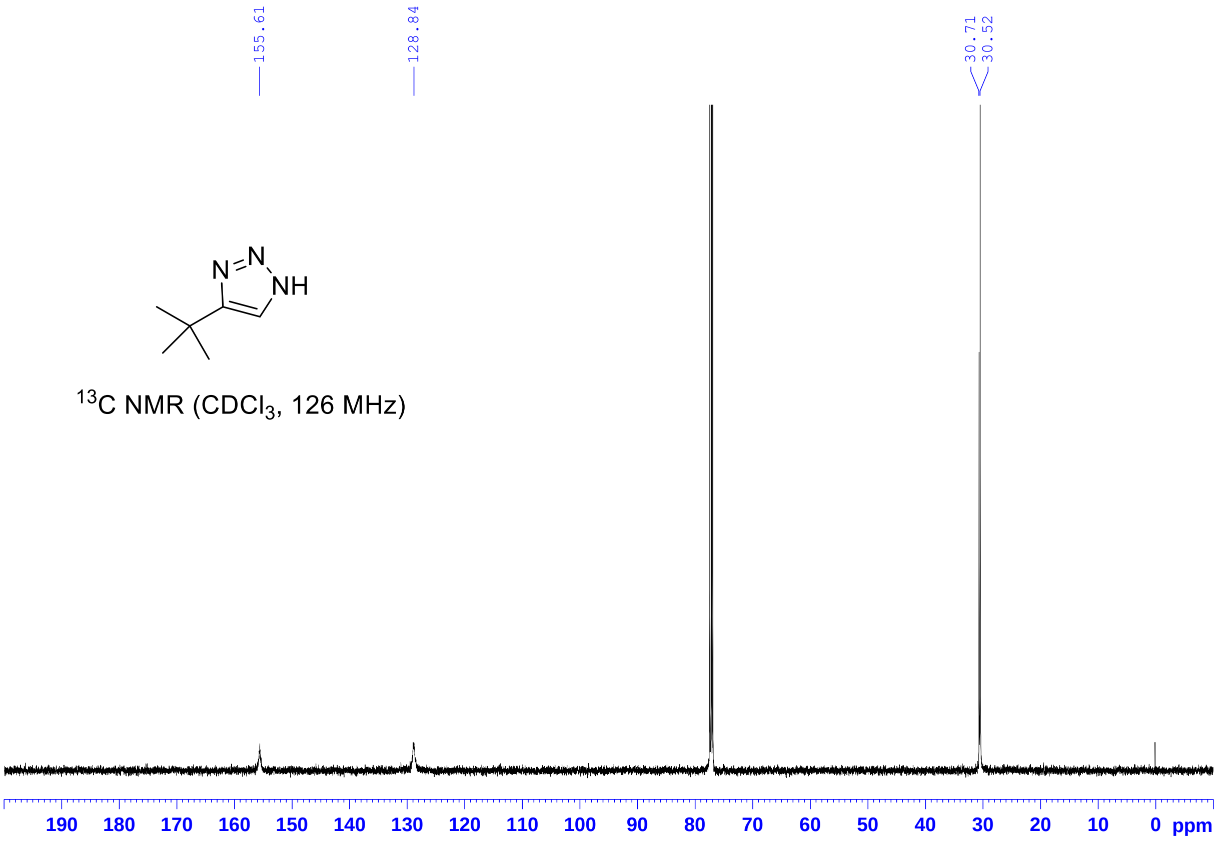

Supporting Figure S40. ^{13}C{^{1}H} NMR spectrum of compound **3q** in $CDCl_3$, 126 MHz.

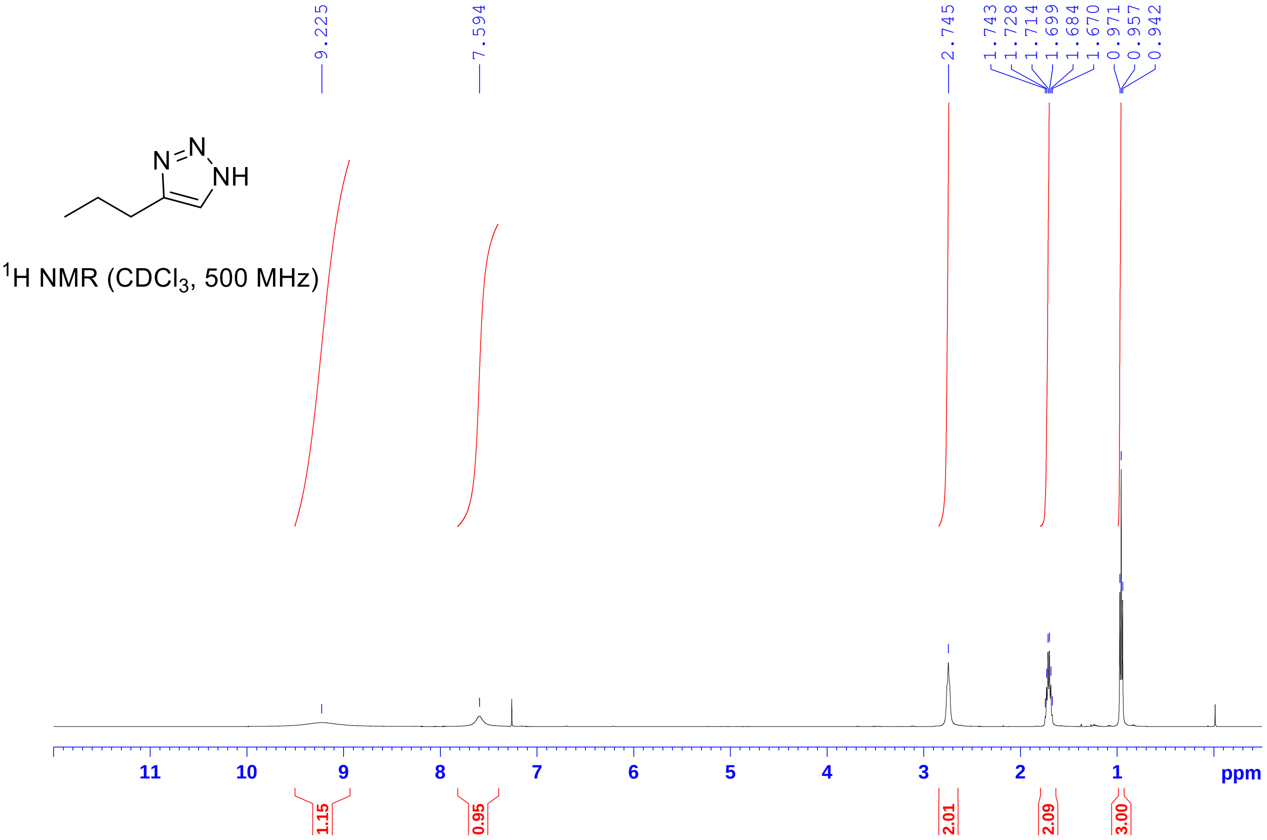

Supporting Figure S41. ^{1}H NMR spectrum of compound **3r** in $CDCl_3$, 500 MHz.

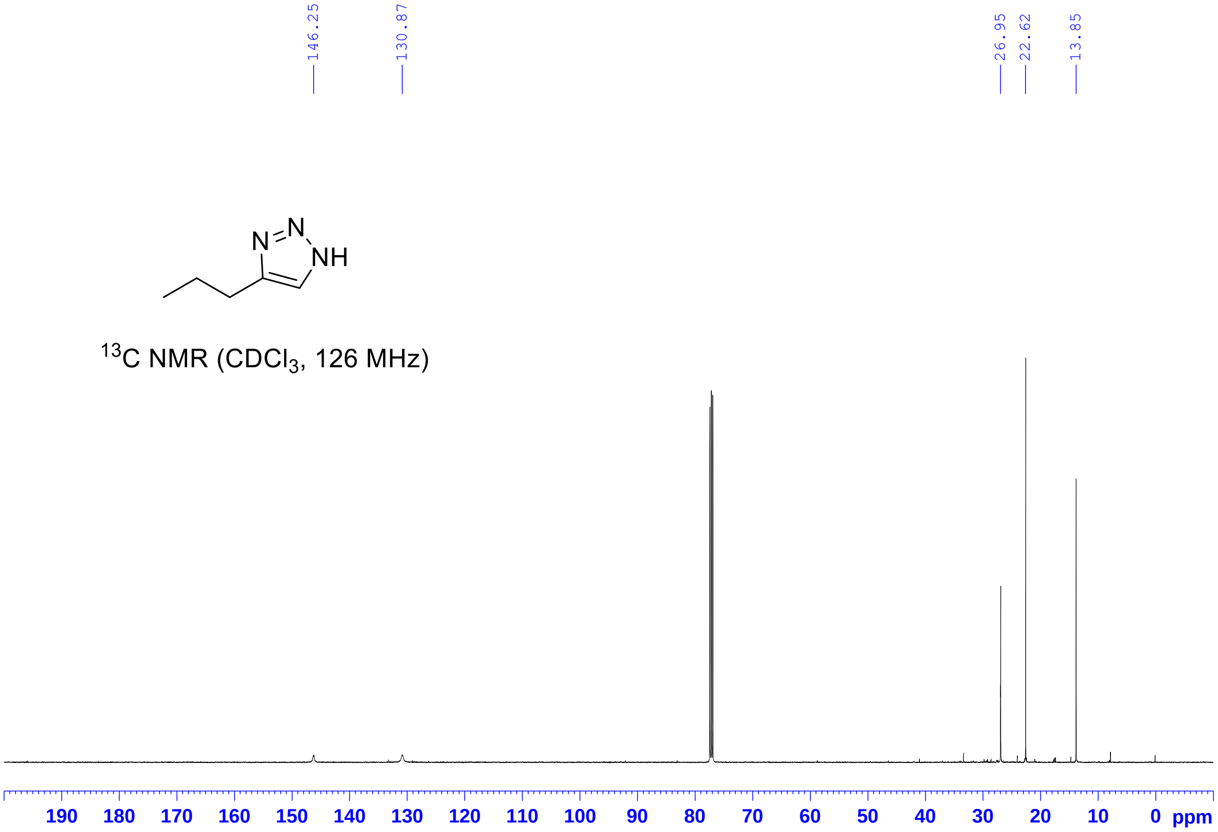

Supporting Figure S42. ^{13}C{^{1}H} NMR spectrum of compound **3r** in $CDCl_3$, 126 MHz.

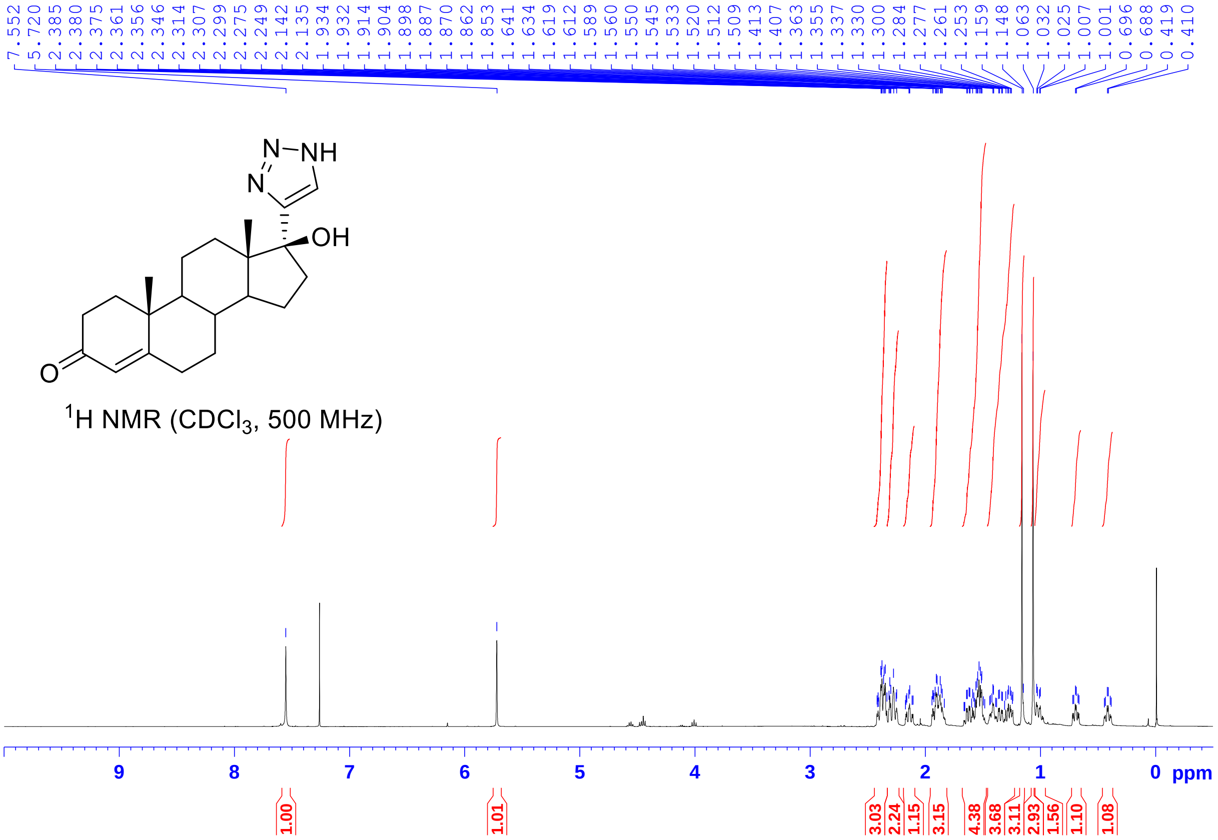

Supporting Figure S43. ^{1}H NMR spectrum of triazole product **3s** in $CDCl_3$, 500 MHz.

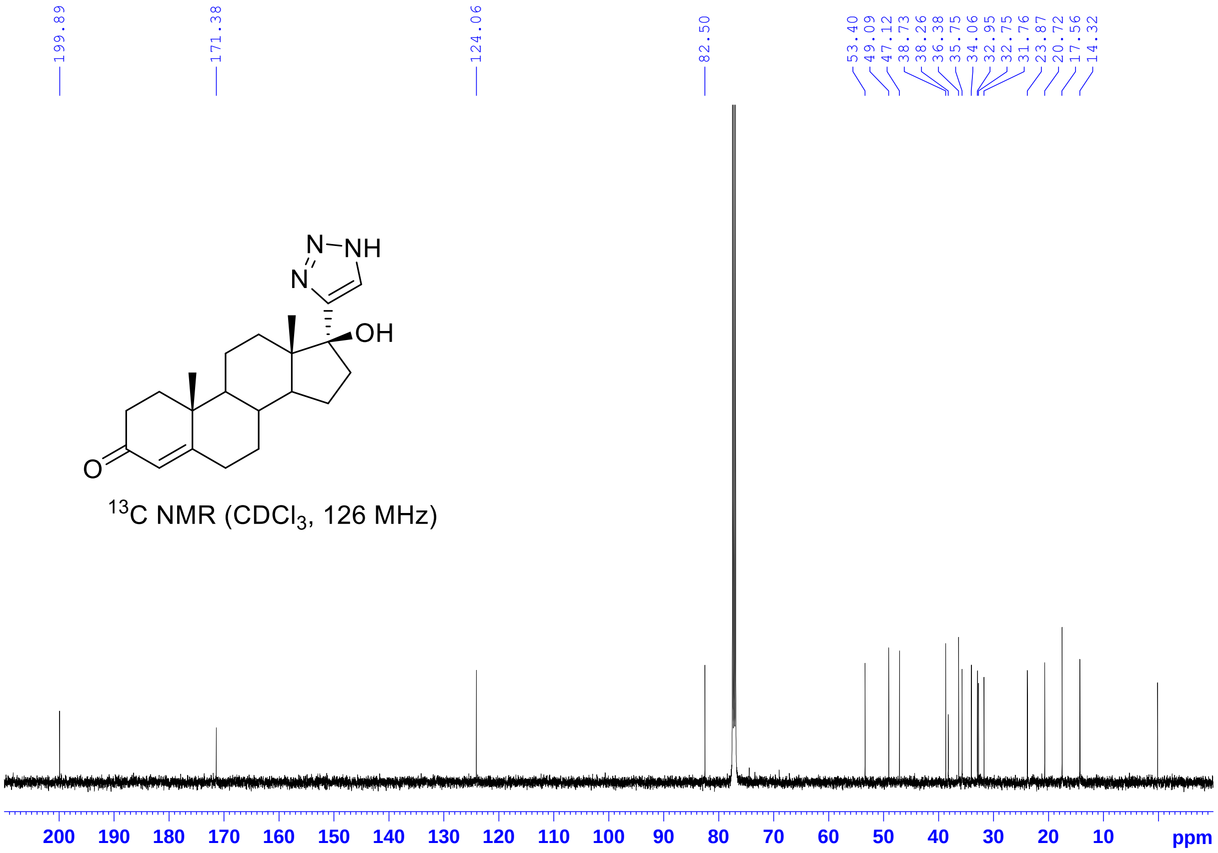

Supporting Figure S44. $^{13}C\{^{1}H\}$ NMR spectrum of triazole product **3s** in $CDCl_3$, 126 MHz. Two carbon resonances are not seen.

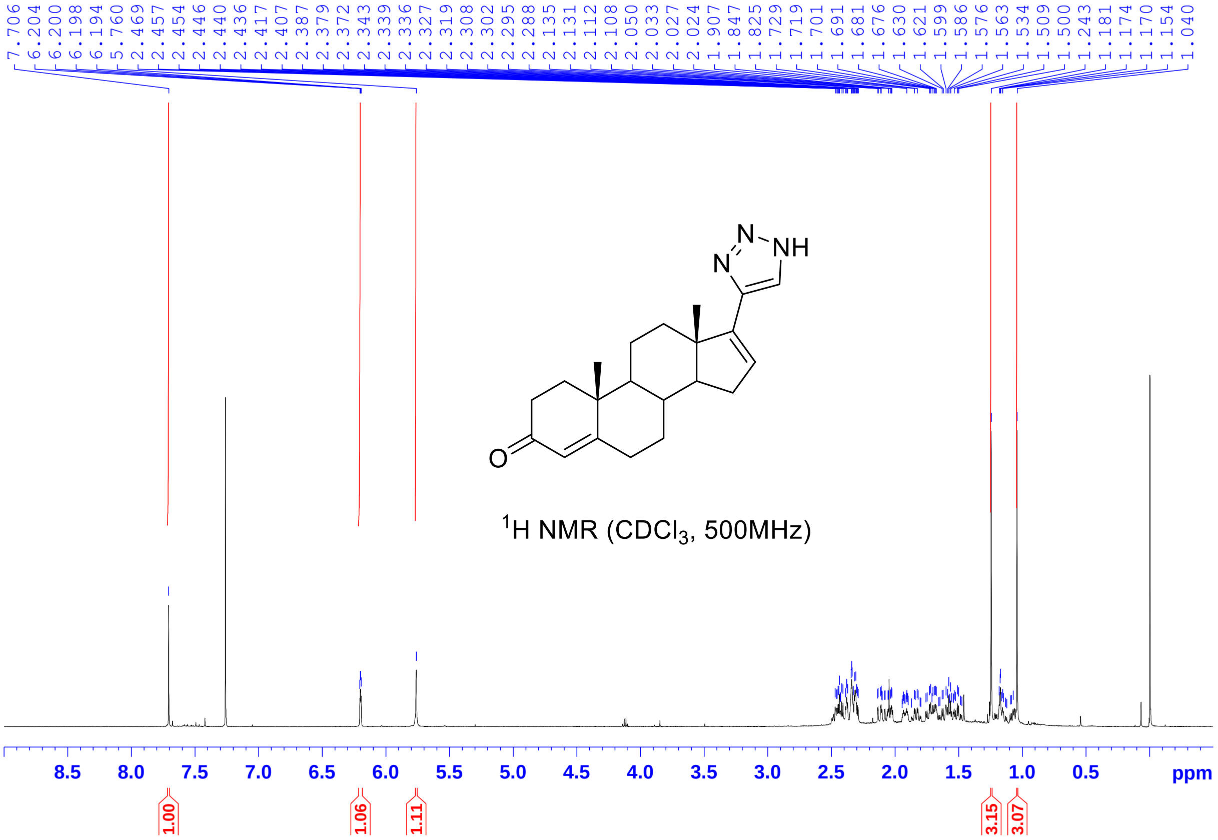

Supporting Figure S45. ^{1}H NMR spectrum of triazole product **3t** in $CDCl_3$, 500 MHz.

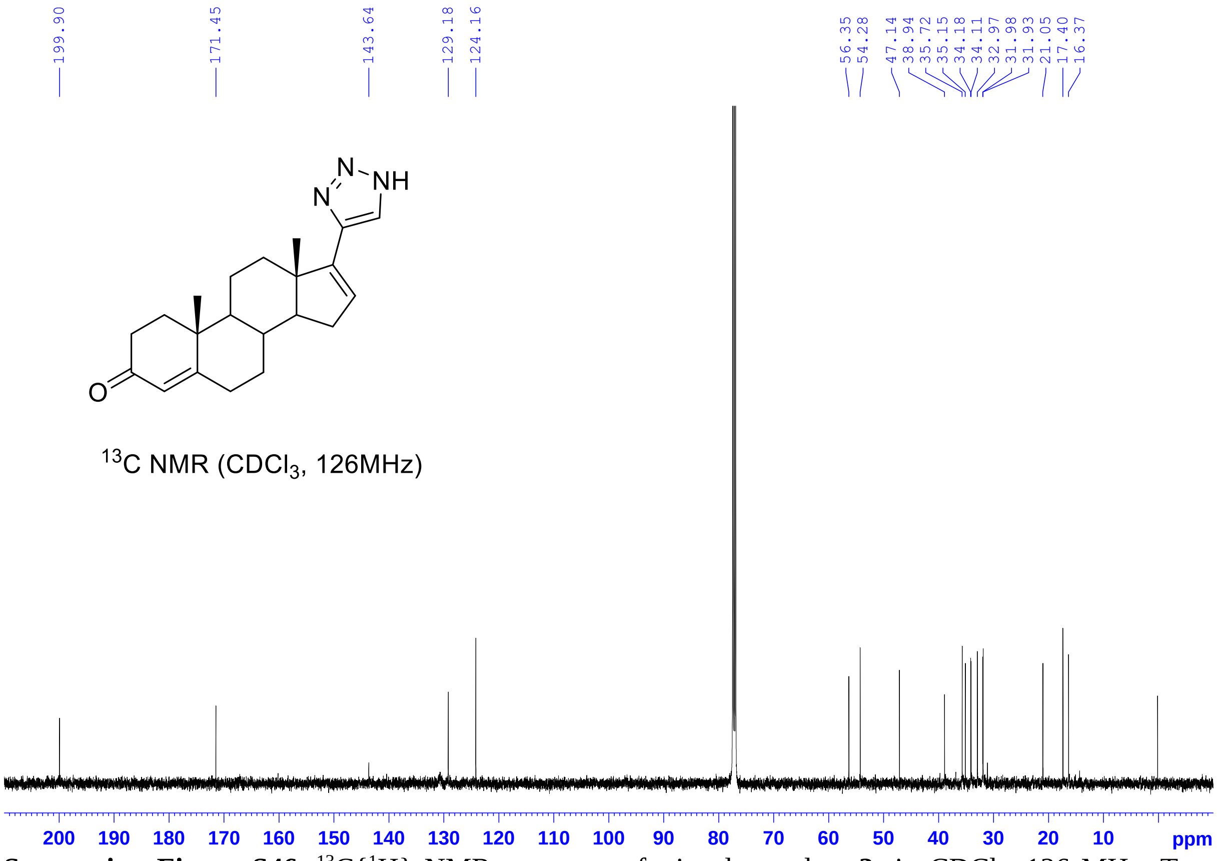

Supporting Figure S46. ^{13}C{^{1}H} NMR spectrum of triazole product **3t** in $CDCl_3$, 126 MHz. Two carbon resonances are not seen.

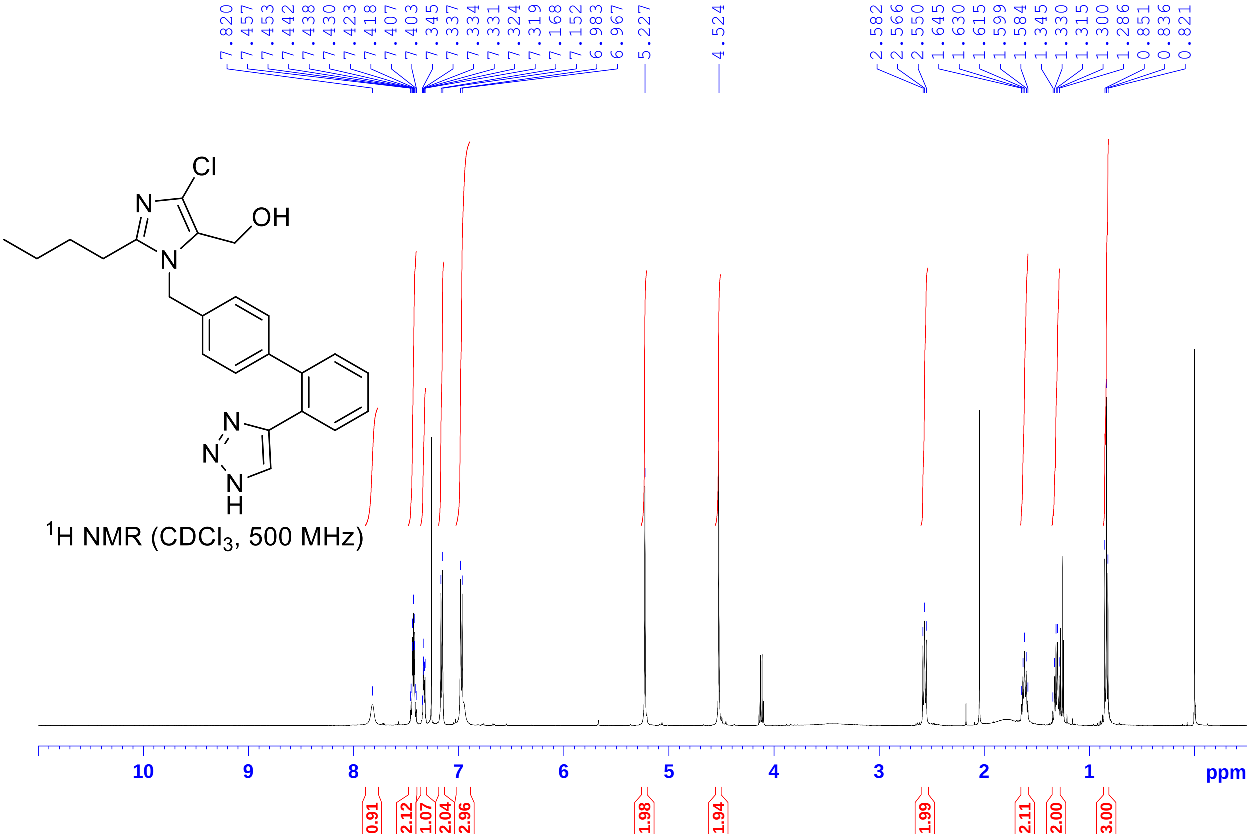

Supporting Figure S47. ^{1}H NMR spectrum of (1-((2'-(1*H*-1,2,3-triazol-4-yl)-[1,1'-biphenyl]-4-yl)methyl)-2-butyl-4-chloro-1H-imidazol-5-yl)methanol (**3u**) in $CDCl_3$, 500 MHz. Signals at δ 4.02, 1.99 and 1.17 are from residual ethyl acetate.

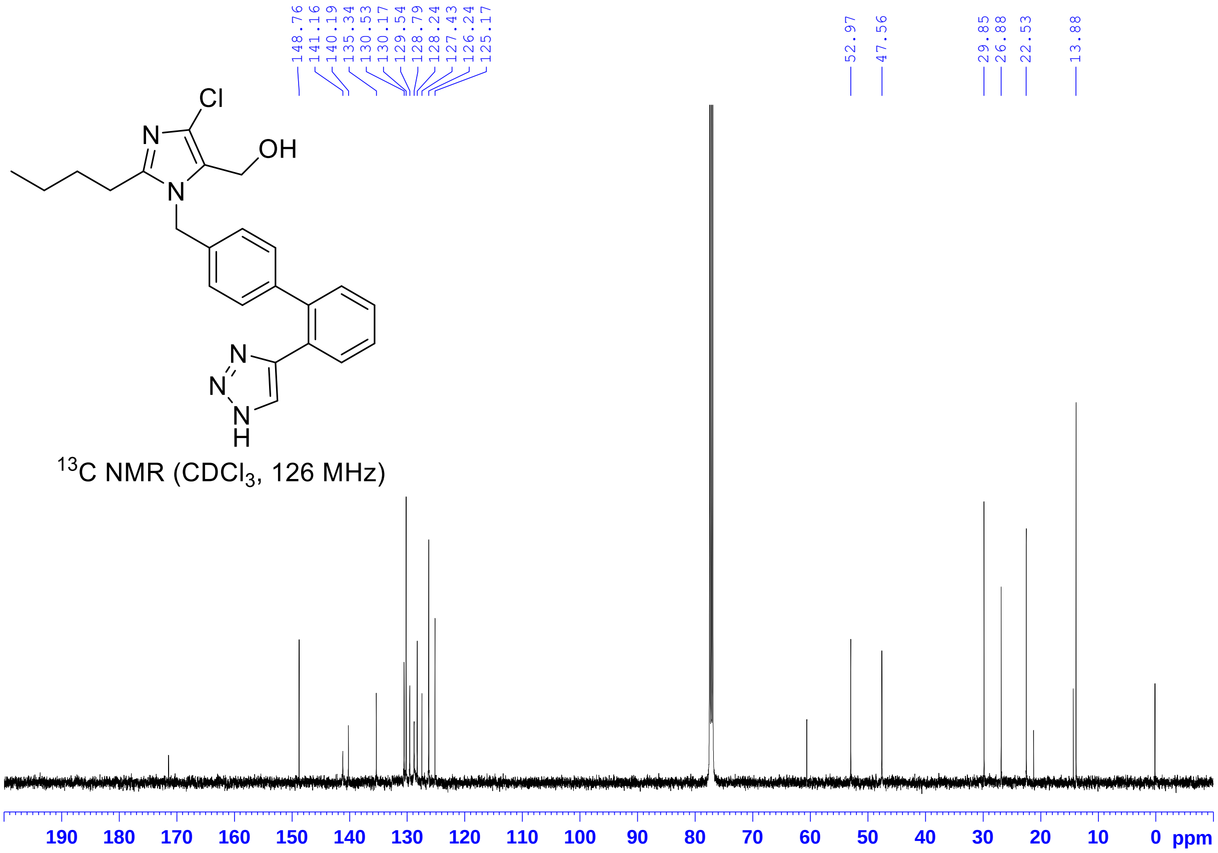

Supporting Figure S48. ^{13}C{^{1}H} NMR spectrum of (1-((2'-(1H-1,2,3-triazol-4-yl)-[1,1'-biphenyl]-4-

yl)methyl)-2-butyl-4-chloro-1H-imidazol-5-yl)methanol (**3u**) in $CDCl_3$, 126 MHz. Signals at δ 170.3, 59.7, 20.7 and 14.4 are from residual ethyl acetate. Three carbon resonances are not seen.

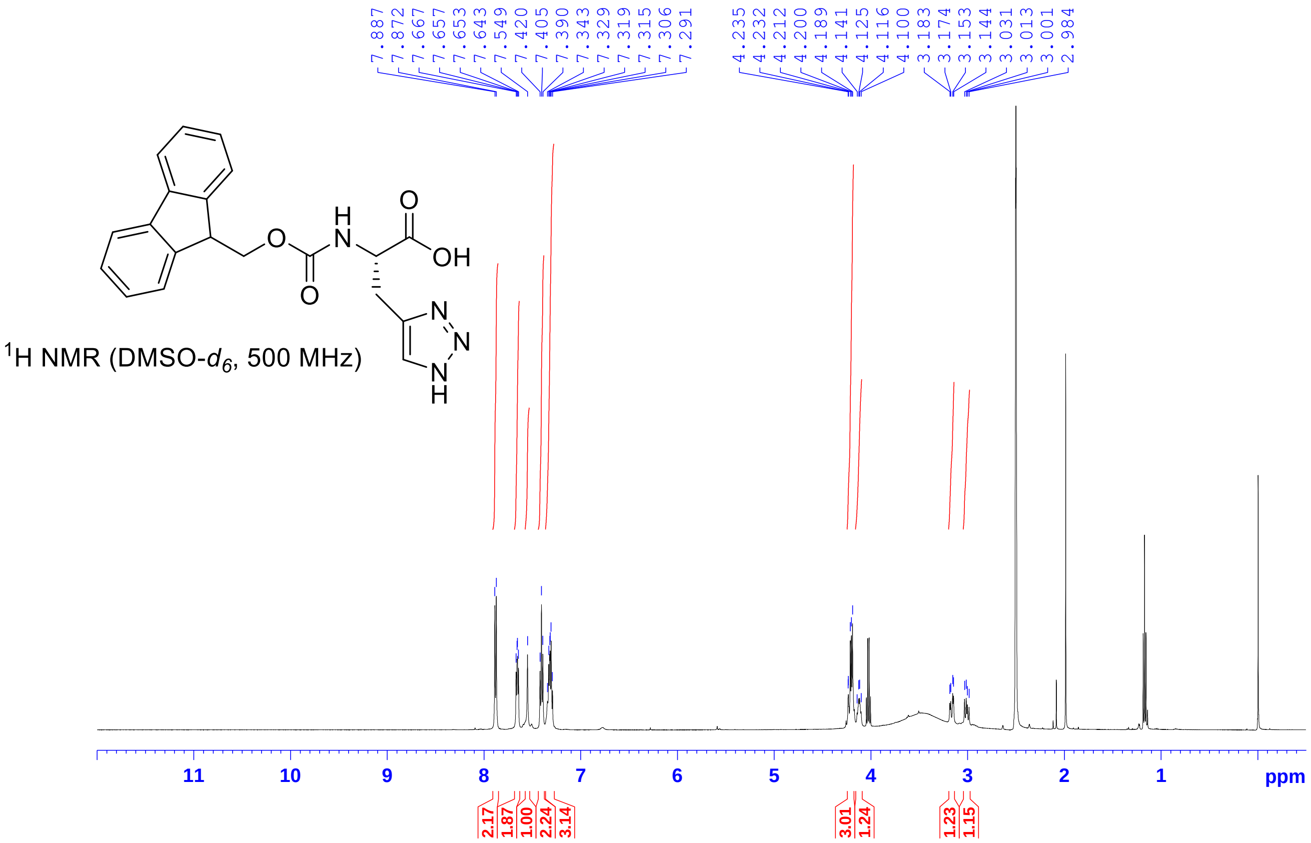

Supporting Figure S49. ^{1}H NMR spectrum of compound **3w** in DMSO-d_6, 500 MHz. Signals at δ 1.17, 1.99, 2.08 and 4.02 are from residual ethyl acetate and acetone.

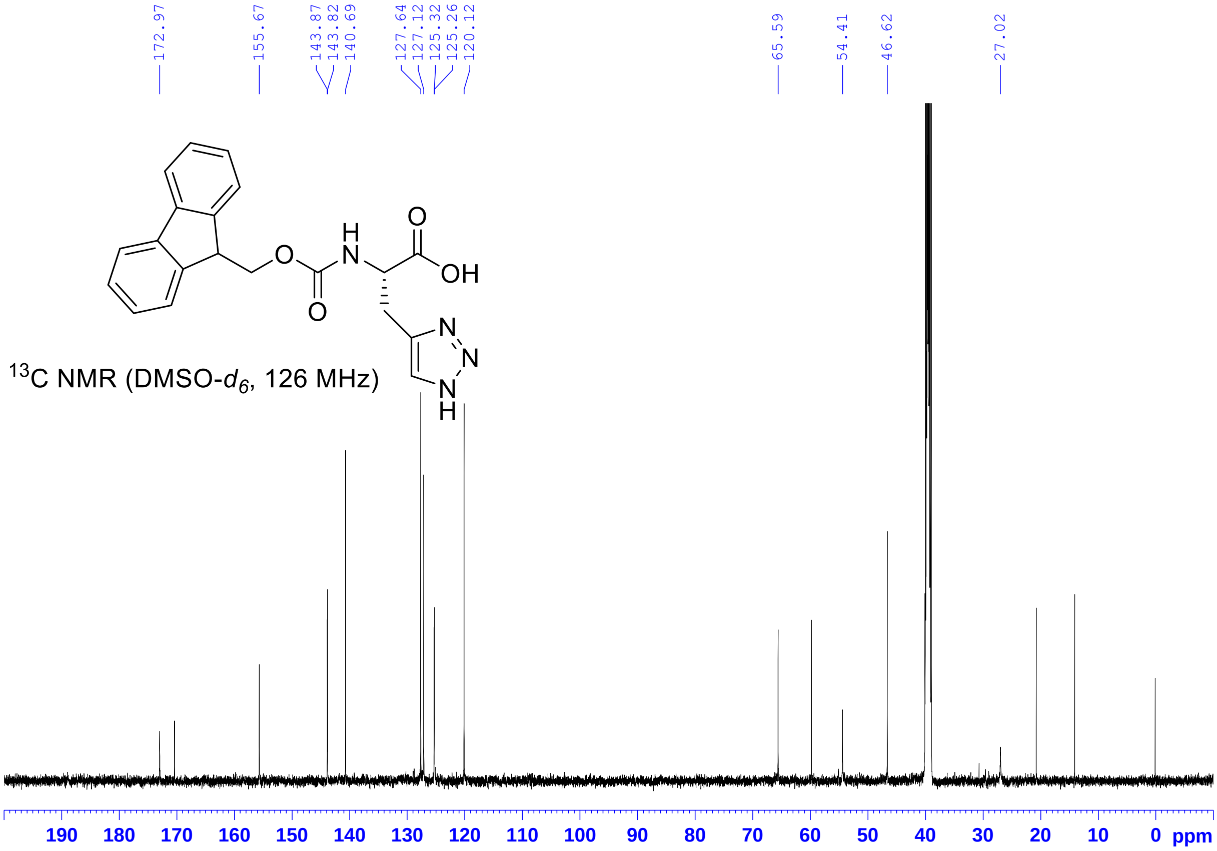

Supporting Figure S50. $^{13}C\{^{1}H\}$ NMR spectrum of compound **3w** in DMSO-d_6, 126 MHz. Signals at δ 170.3, 59.7, 20.7 and 14.4 are from residual ethyl acetate.

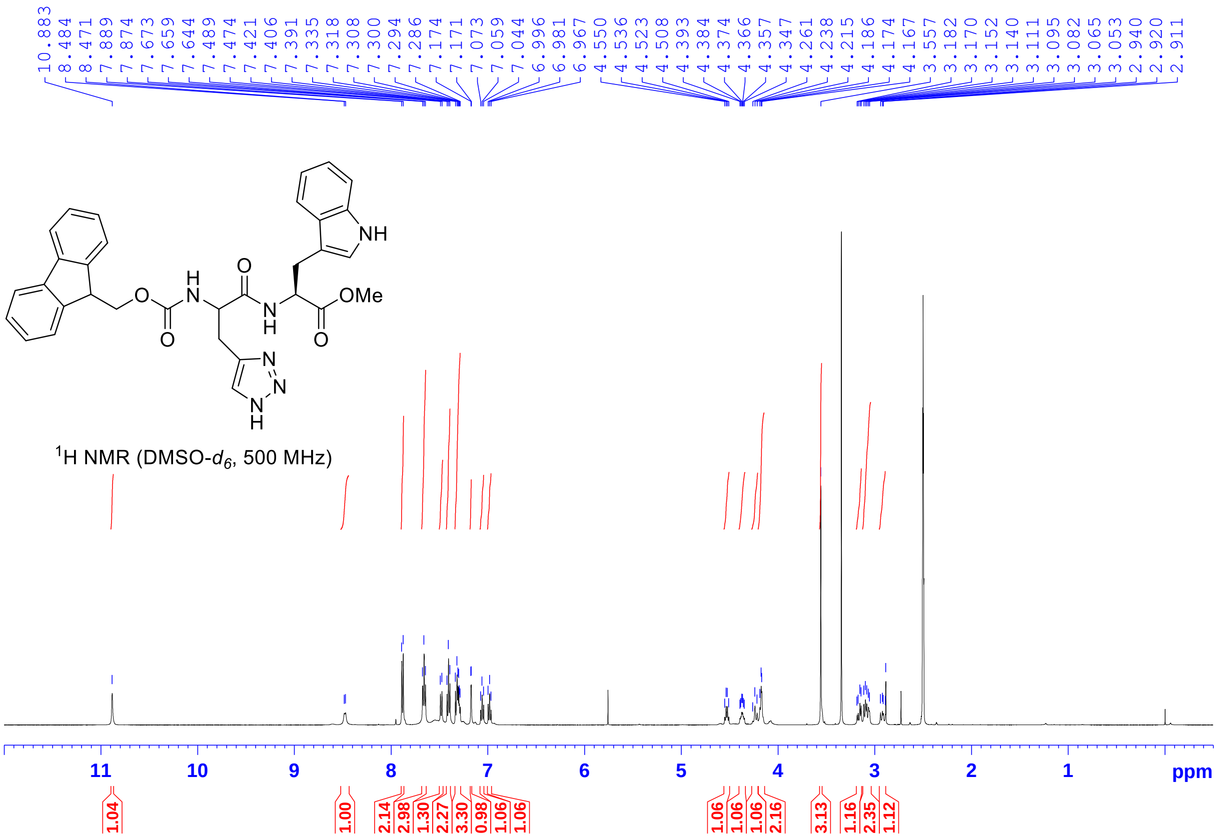

Supporting Figure S51. ^{1}H NMR spectrum of compound **3x** in DMSO-d_6, 500 MHz. Signals at δ 7.95, 5.76, 2.89, 2.73 ppm are from residual dichloromethane and DMF.

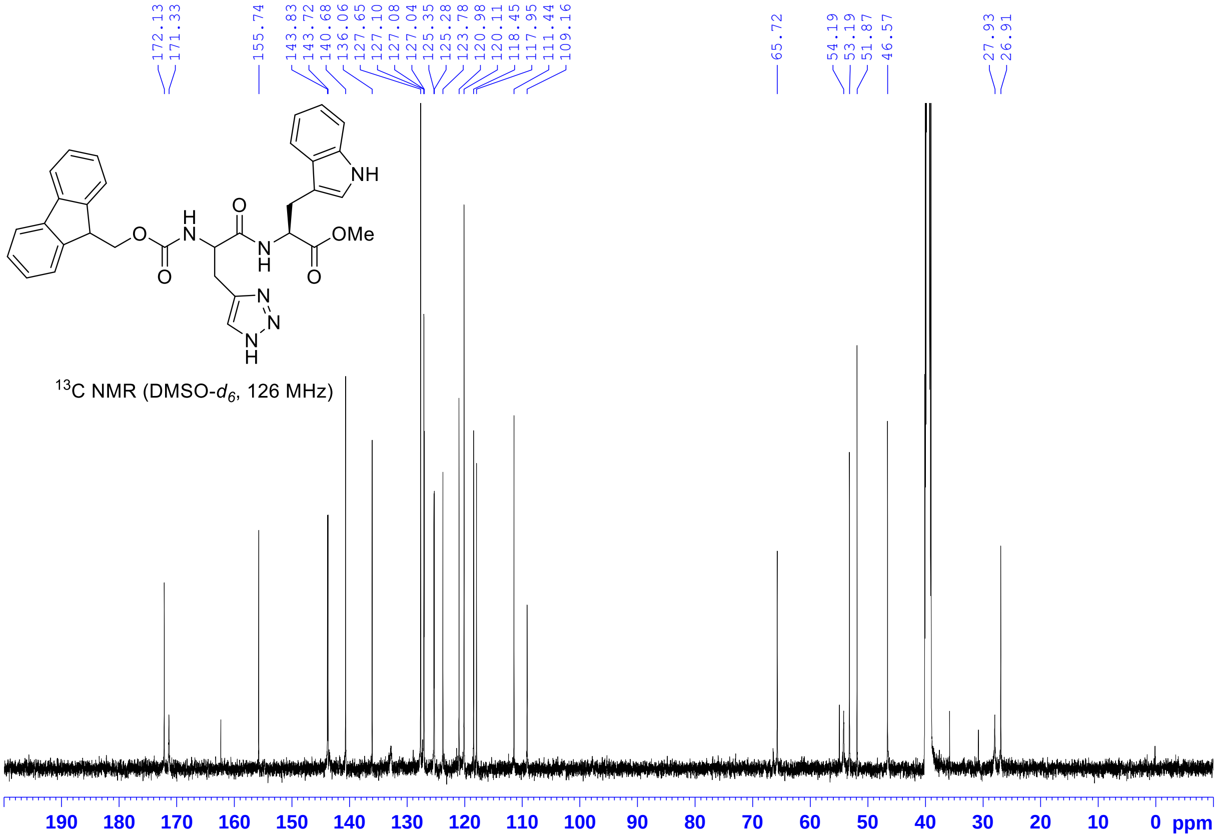

Supporting Figure S52. $^{13}C\{^{1}H\}$ NMR spectrum of compound **3x** in DMSO-*d*$_6$, 126 MHz. Signals at δ 162.3, 54.8, 35.7, 30.7 are from residual dichloromethane and DMF.

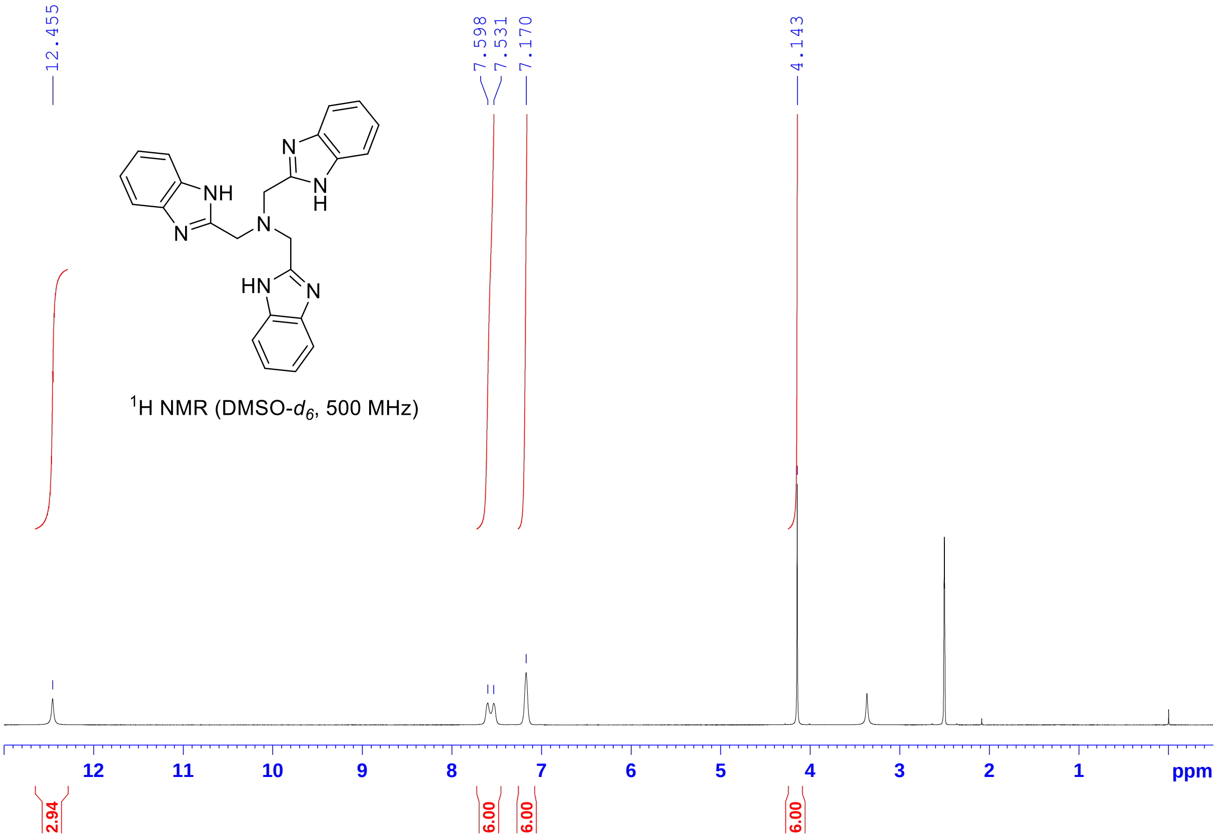

Supporting Figure S53. ^{1}H NMR spectrum of Tris(2-benzimidazolylmethyl)amine ((BimH)$_3$) in DMSO-d_6, 500 MHz.

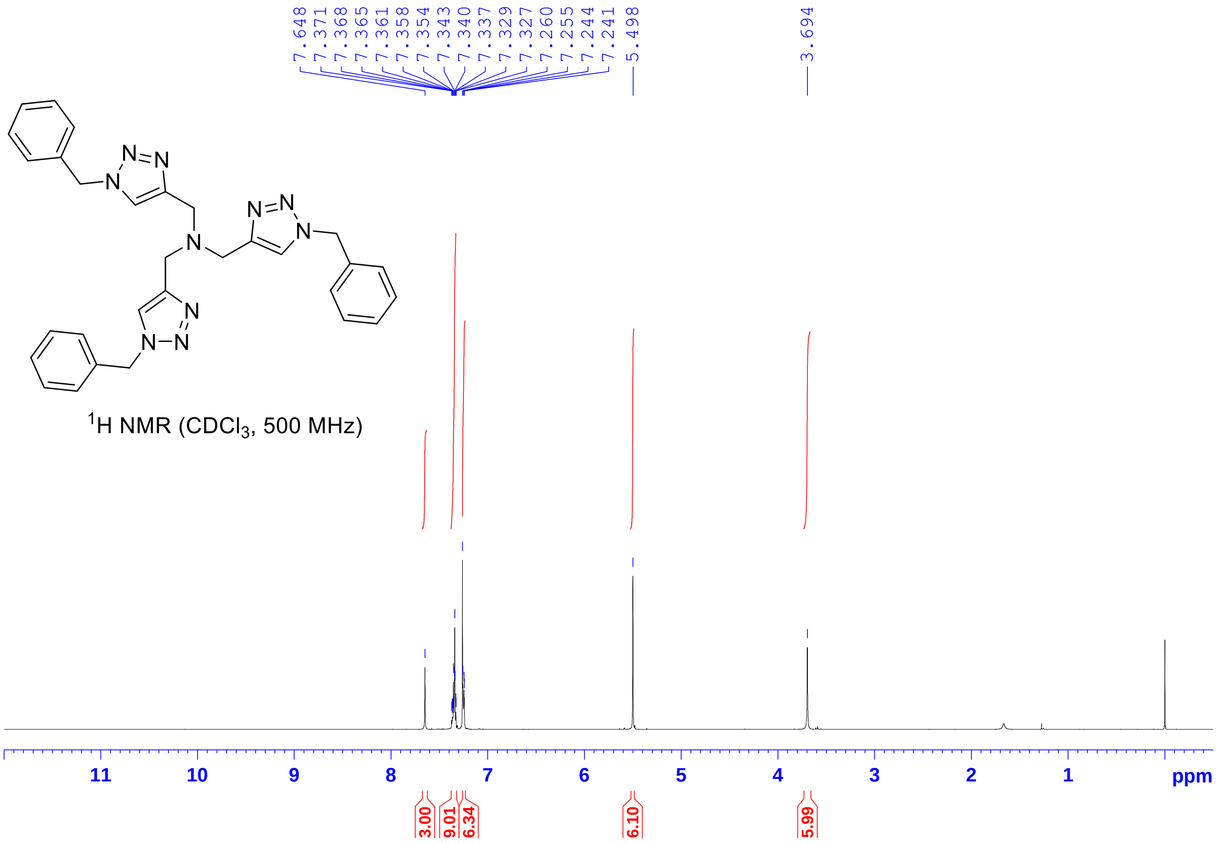

Supporting Figure S54. ^{1}H NMR spectrum of Tris[(1-benzyl-1*H*-1,2,3-triazol-4-yl)methyl]amine (TBTA) in $CDCl_3$, 500 MHz.

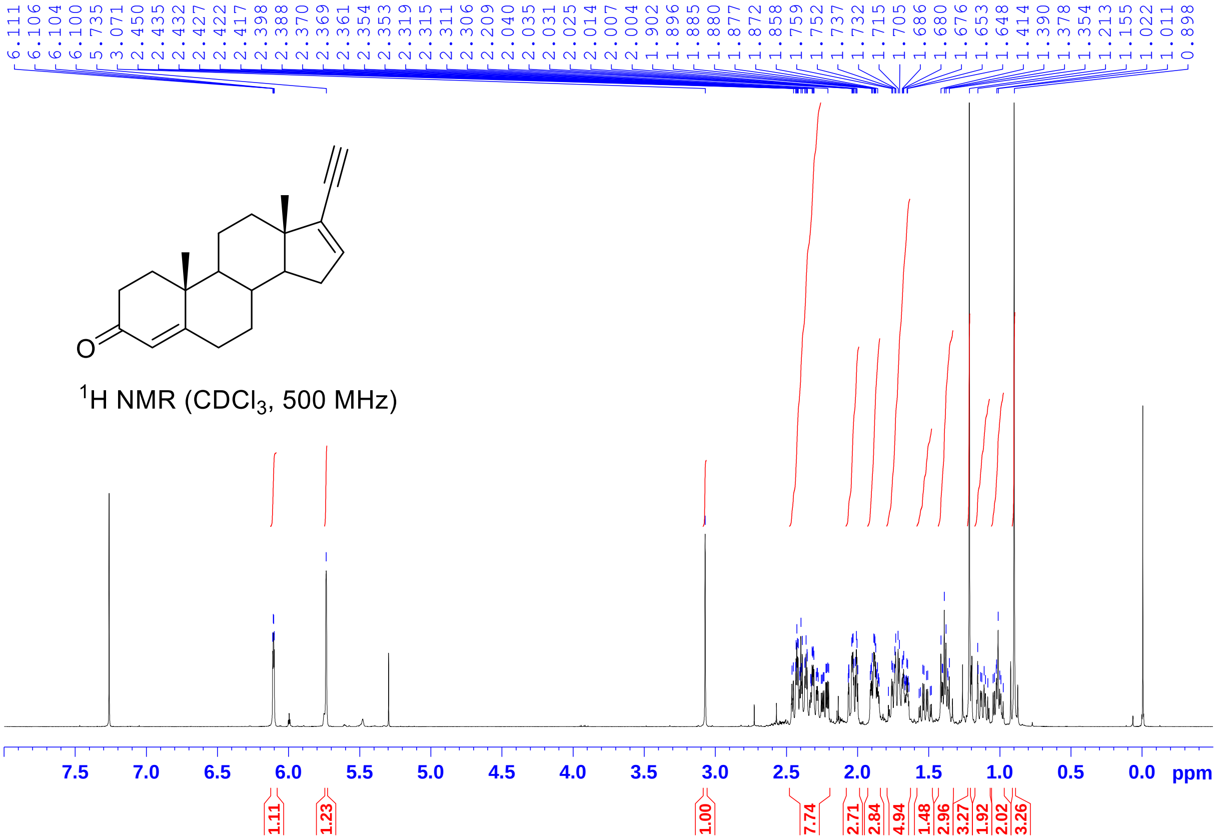

Supporting Figure S55. ^{1}H NMR spectrum of Pregna-4,16-dien-20-yn-3-one (**1t**) in $CDCl_3$, 500 MHz.

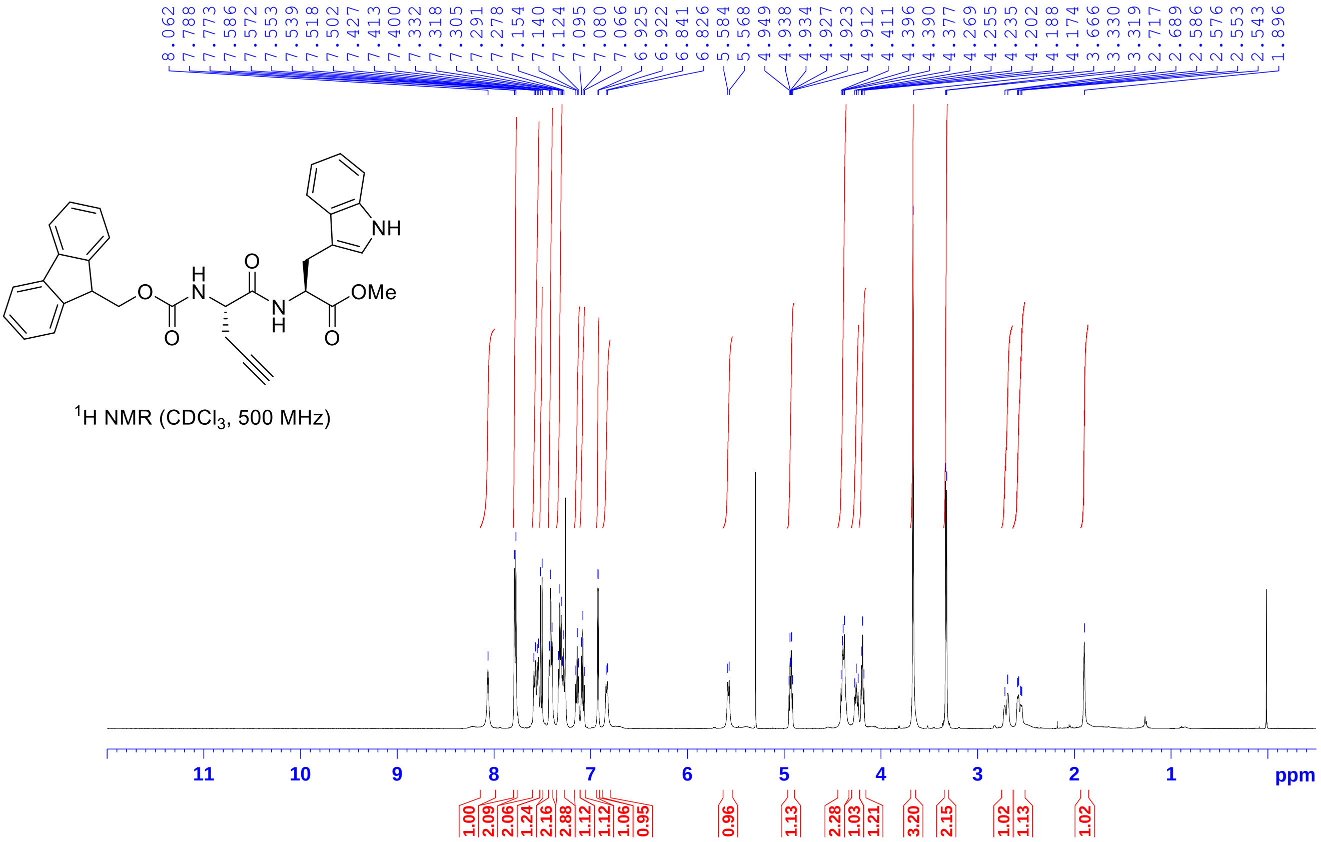

Supporting Figure S56. ^{1}H NMR spectrum of compound **1x** in $CDCl_3$, 500 MHz.

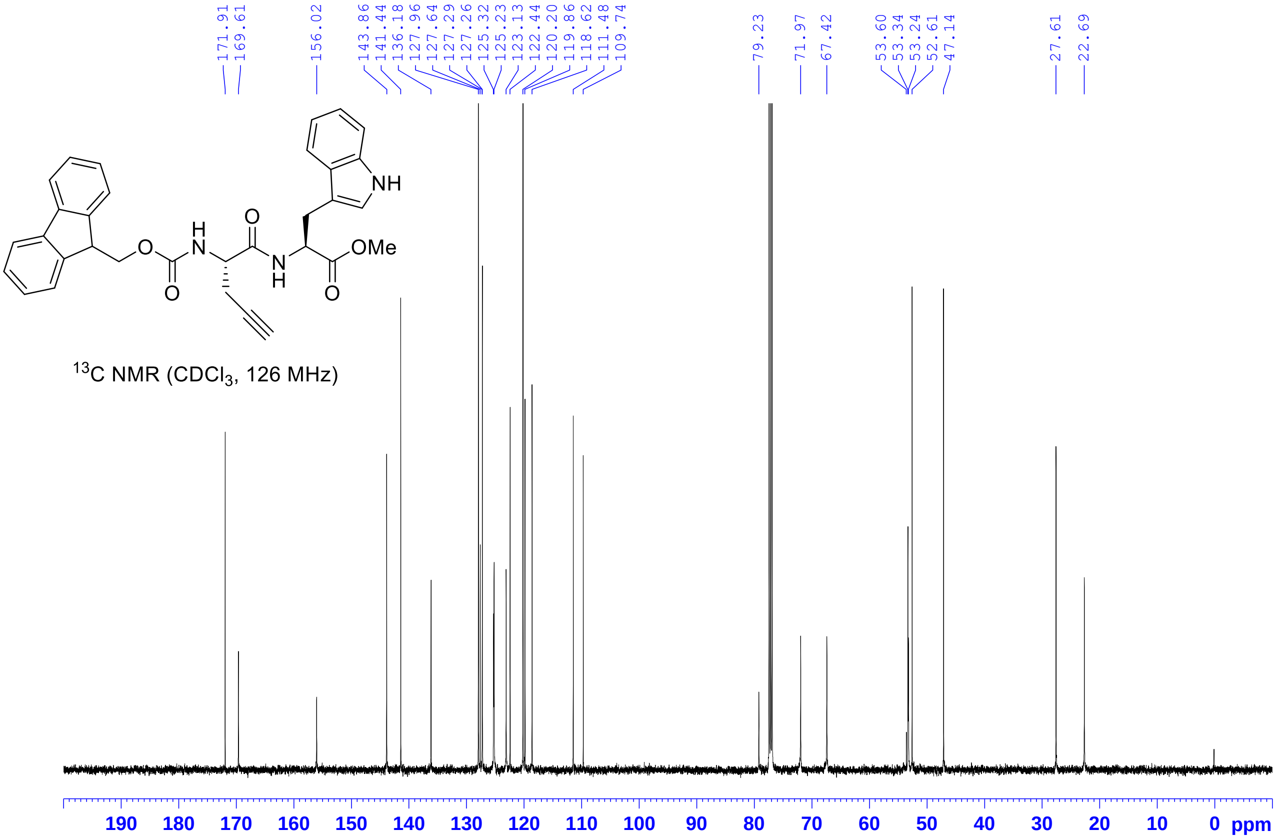

Supporting Figure S57. ^{13}C{^{1}H} NMR spectrum of compound **1x** in $CDCl_3$, 126 MHz.

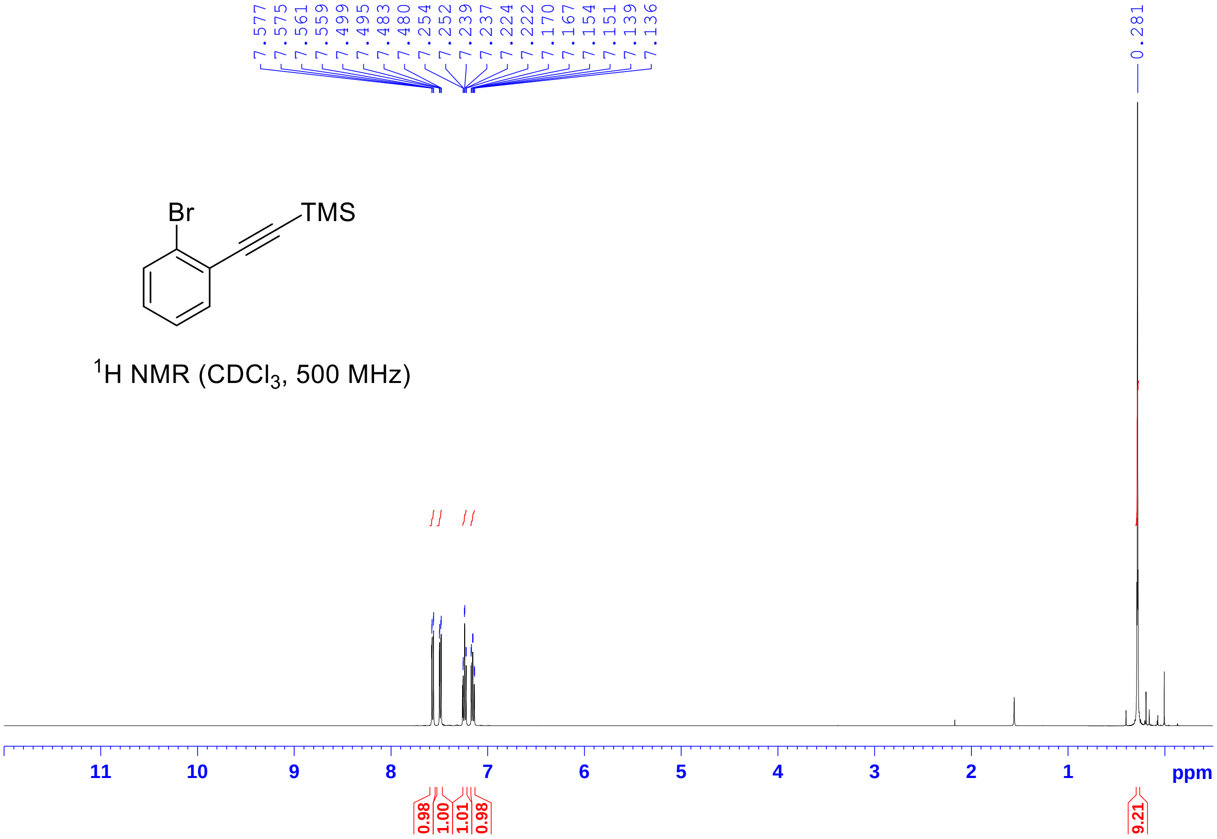

Supporting Figure S58. ^{1}H NMR spectrum of 1-bromo-2-[2-(trimethylsilyl)ethynyl]benzene in $CDCl_3$, 500 MHz.

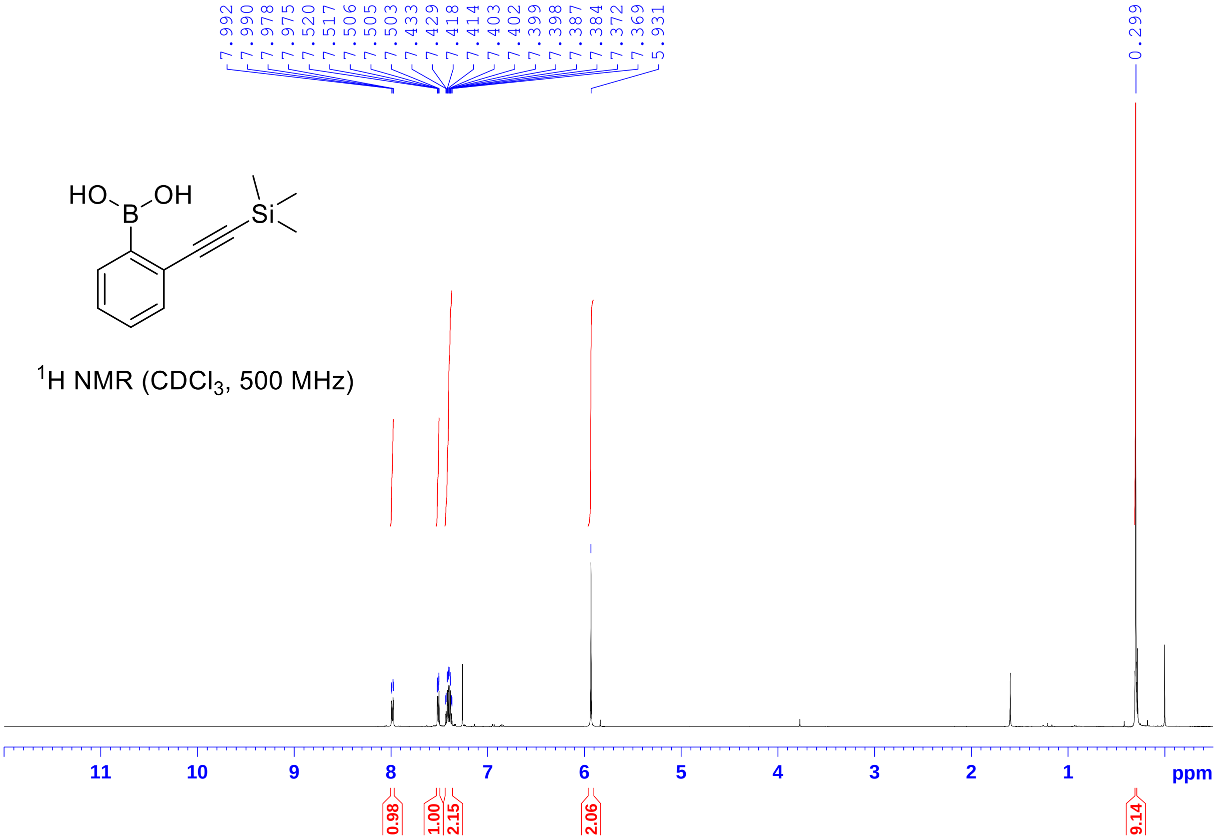

Supporting Figure S59. ^{1}H NMR spectrum of 2-(2-Trimethylsilylethynyl)phenylboronic acid in $CDCl_3$, 500 MHz.

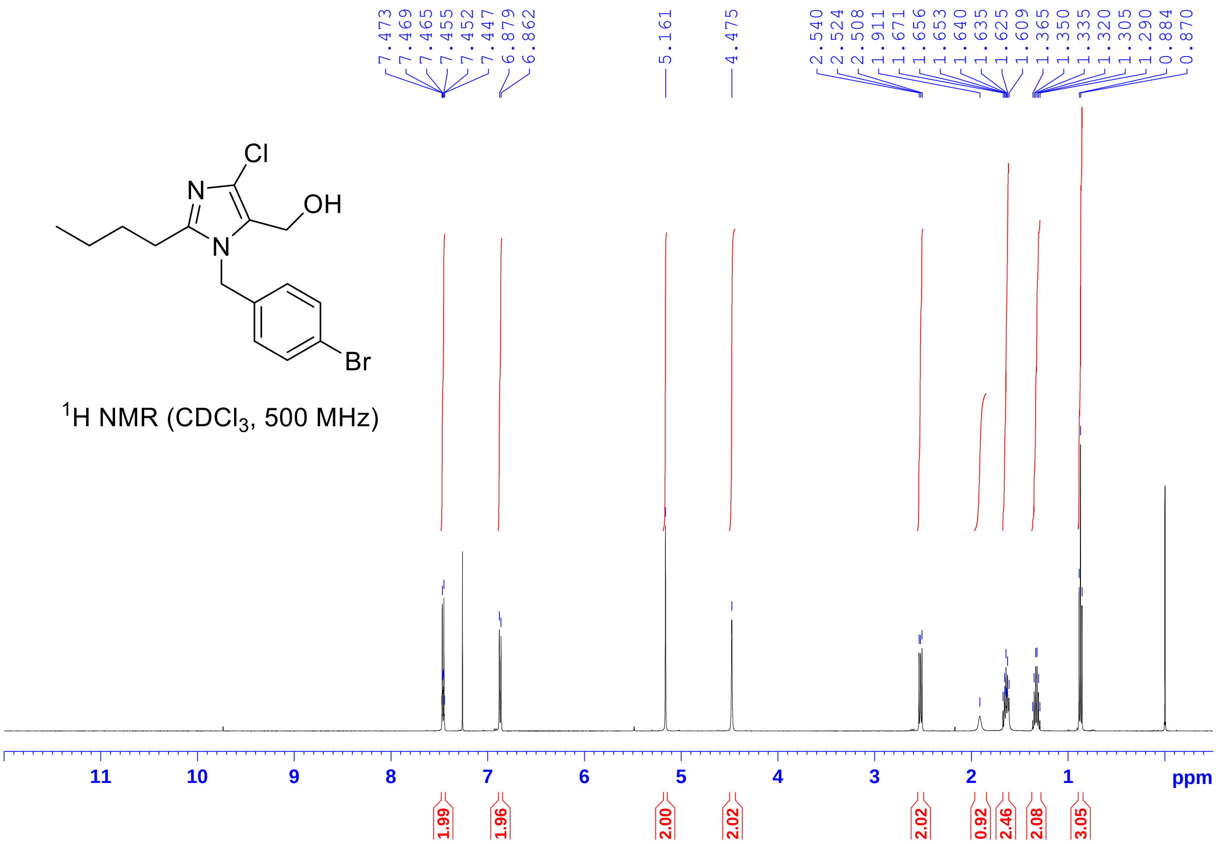

Supporting Figure S60. ^{1}H NMR spectrum of 2-Butyl-1-(4-bromobenzyl)-4-chloro-5-(hydroxymethyl)-1*H*-imidazole in $CDCl_3$, 500 MHz.

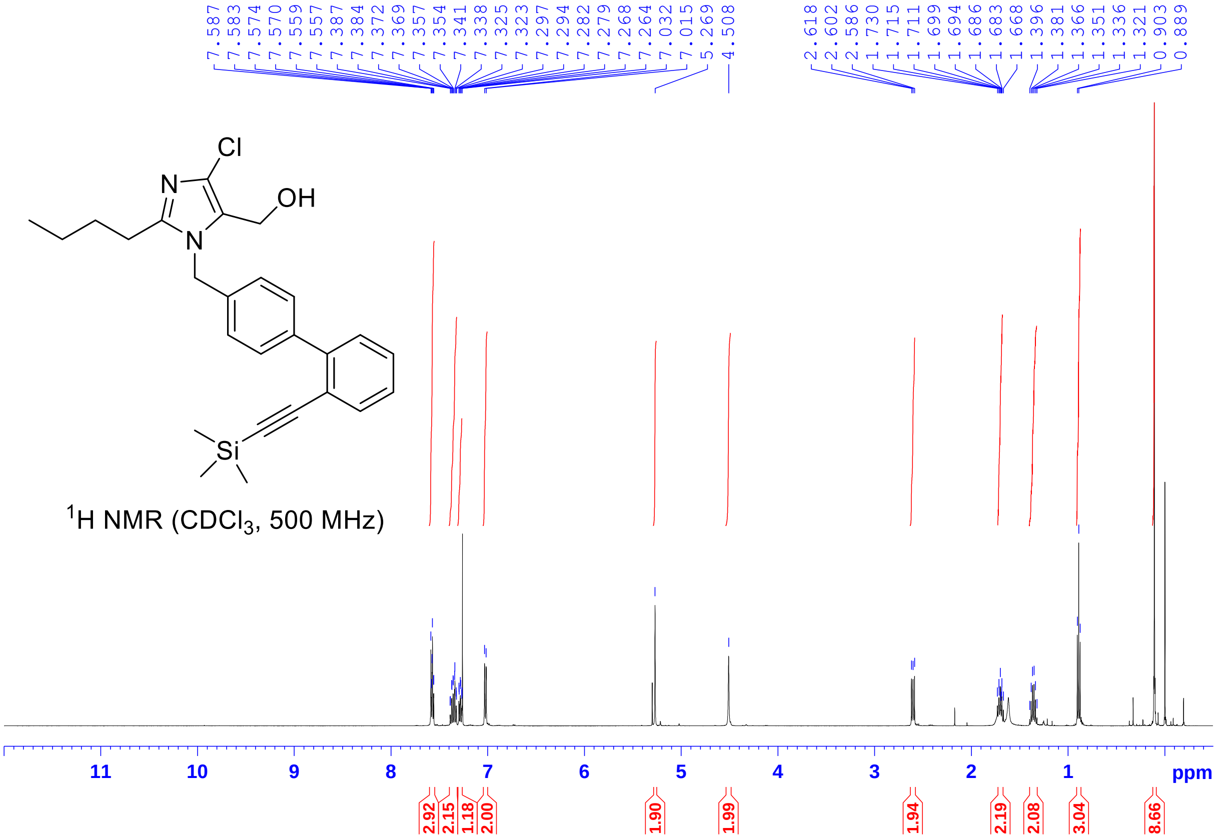

Supporting Figure S61. ^{1}H NMR spectrum of (2-butyl-4-chloro-1-((2'-((trimethylsilyl)ethynyl)-[1,1'-biphenyl]-4-yl)methyl)-1H-imidazol-5-yl)methanol in $CDCl_3$, 500 MHz.

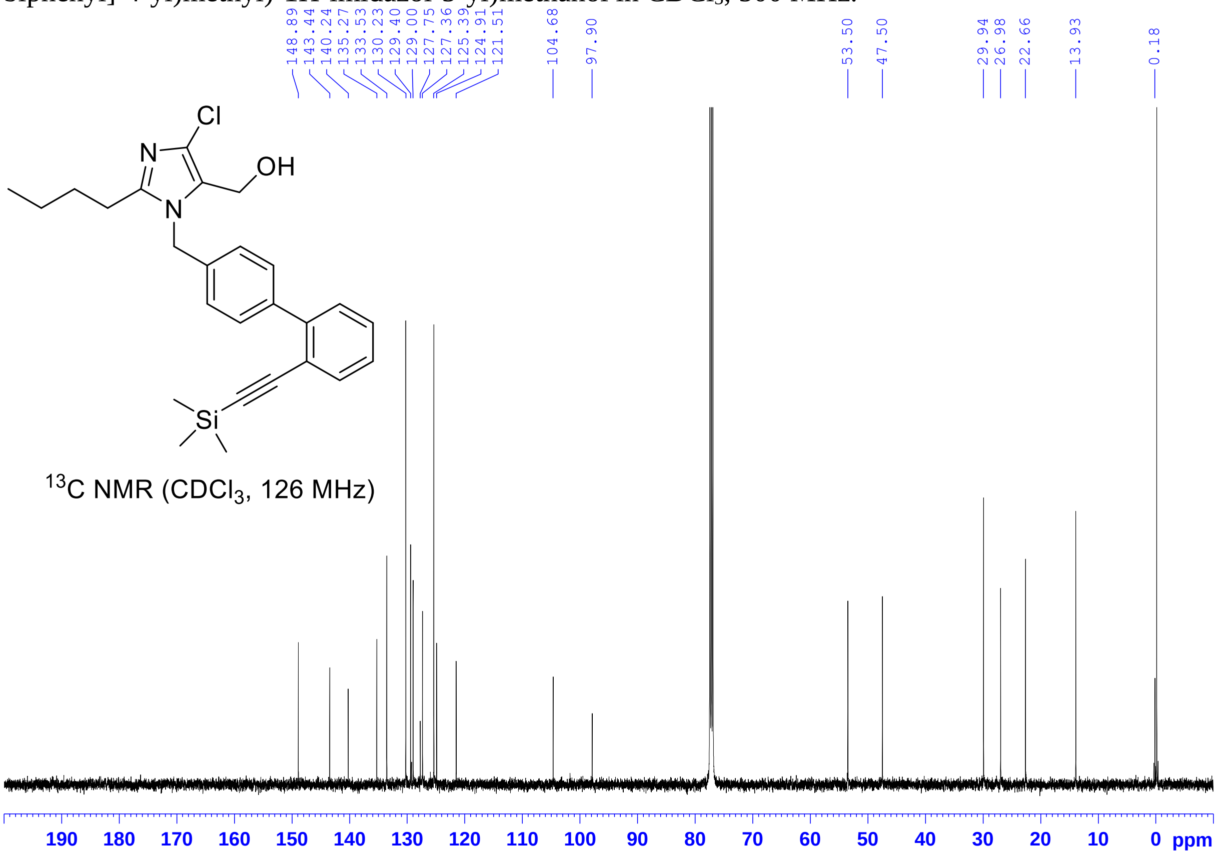

Supporting Figure S62. $^{13}C\{^{1}H\}$ NMR spectrum of (2-butyl-4-chloro-1-((2'-((trimethylsilyl)ethynyl)-[1,1'-biphenyl]-4-yl)methyl)-1H-imidazol-5-yl)methanol in $CDCl_3$, 126 MHz.

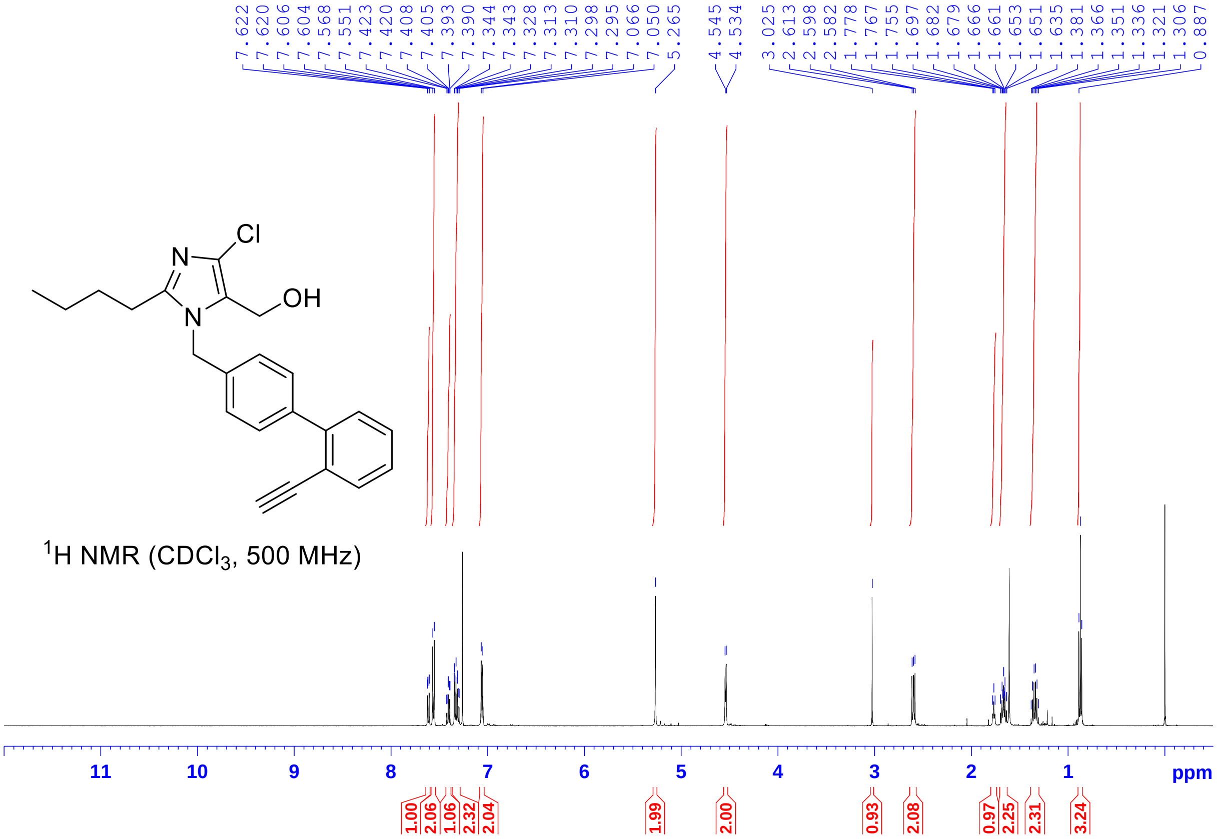

Supporting Figure S63. ^{1}H NMR spectrum of (2-butyl-4-chloro-1-((2'-ethynyl-[1,1'-biphenyl]-4-yl)methyl)-1H-imidazol-5-yl)methanol (**1u**) in $CDCl_3$, 500 MHz.

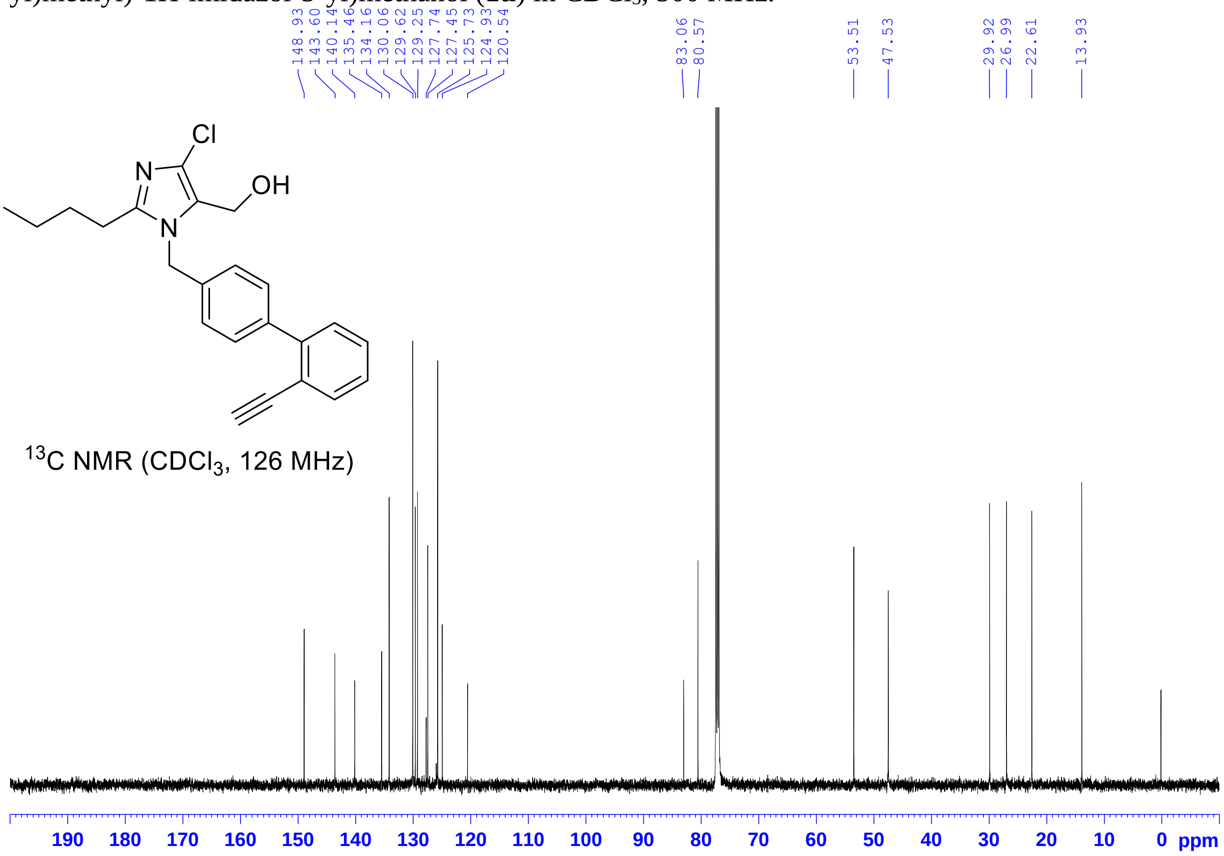

Supporting Figure S64. ^{13}C{^{1}H} NMR spectrum of (2-butyl-4-chloro-1-((2'-ethynyl-[1,1'-biphenyl]-4-yl)methyl)-1H-imidazol-5-yl)methanol (**1u**) in $CDCl_3$, 126 MHz.

4. PHOTOS OF EQUIPMENT USED FOR EXPERIMENTAL WORK

Method A: For reactions at elevated temperatures.

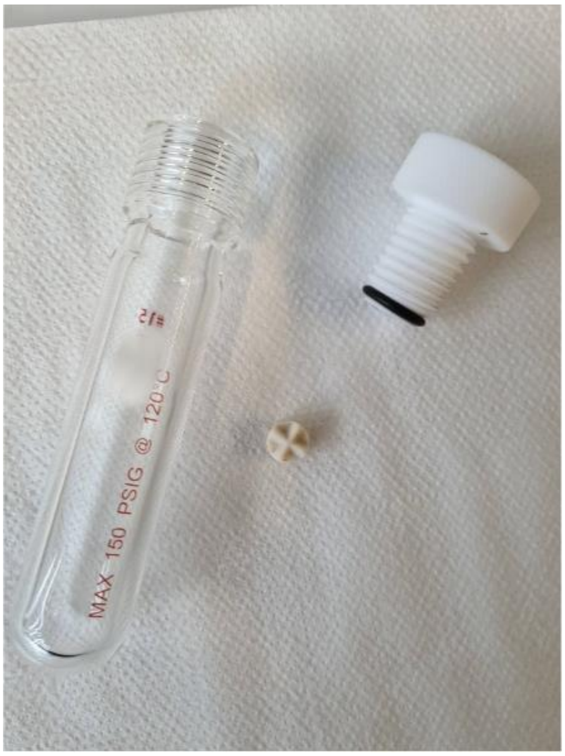

Supporting Photo S2. 21 mL ACE pressure tube, magnetic stirrer and screw cap.

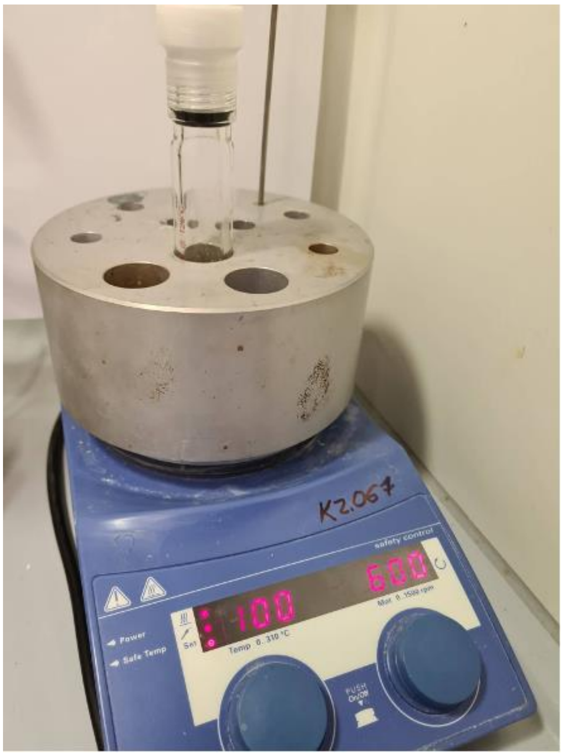

Supporting Photo S4. Reactions heated and stirred in aluminum heating block.

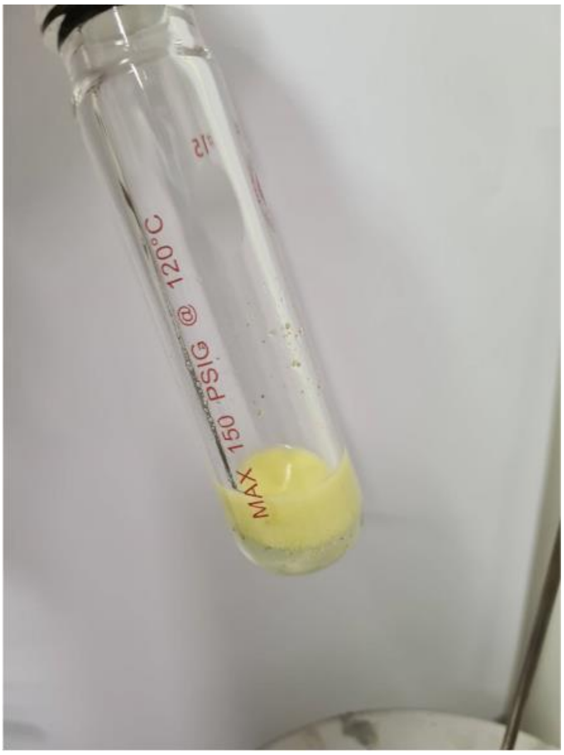

Supporting Photo S3. Reaction mixture (phenylacetylene, $CuSO_4$, sodium ascorbate, NaN_3, H_2SO_4 in THF/H_2O/EtOH) at t=0.

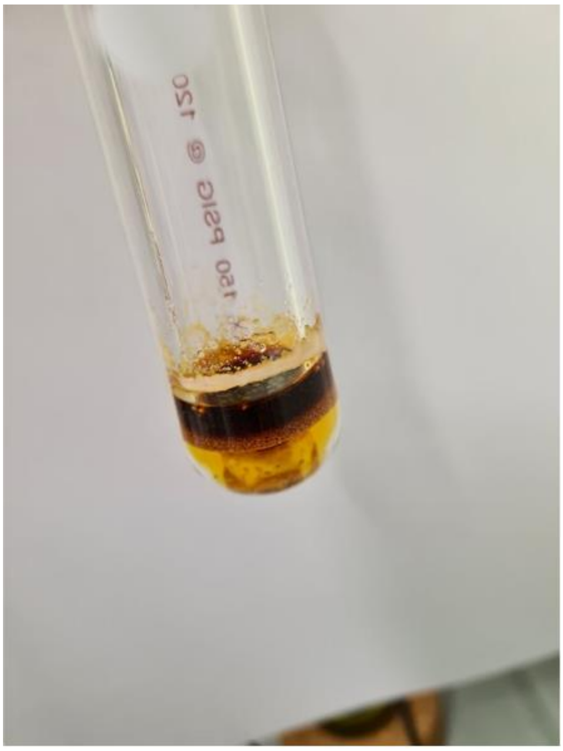

Supporting Photo S5. Reaction mixture (phenylacetylene, $CuSO_4$, sodium ascorbate, NaN_3, H_2SO_4 in THF/H_2O/EtOH) at t=24 h.

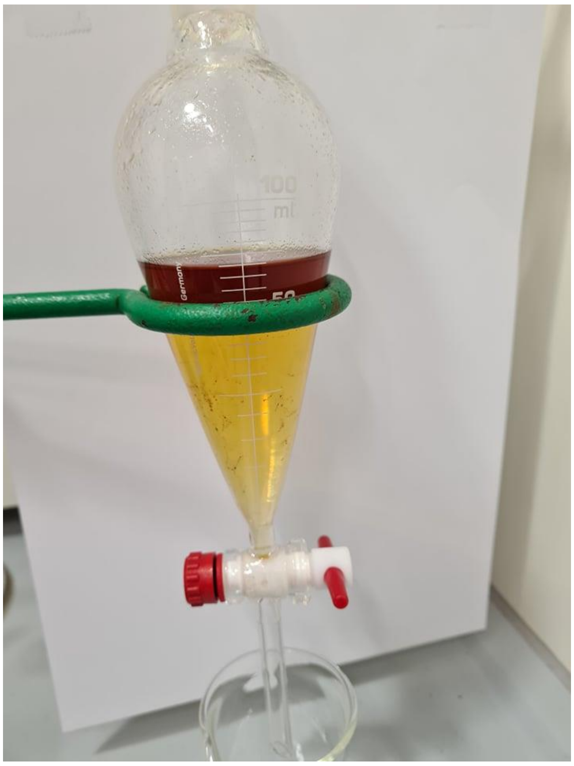

Supporting Photo S6. Extraction with EtOAc and NH_4Cl solution.

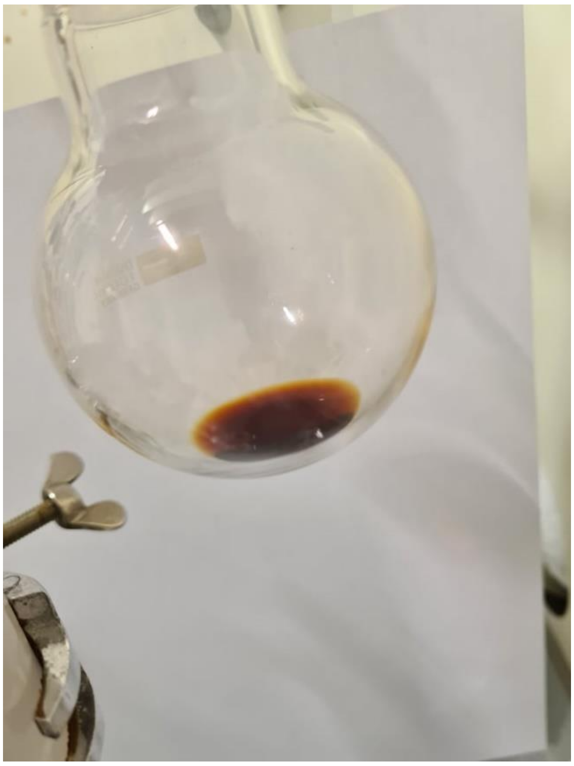

Supporting Photo S7. Dissolving crude product in minimal amount of mobile phase for column chromatography.

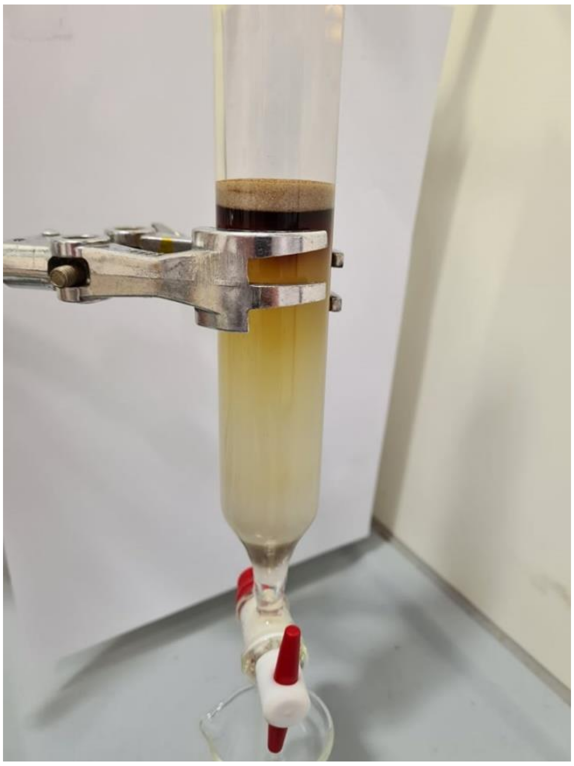

Supporting Photo S8. Silica column chromatography.

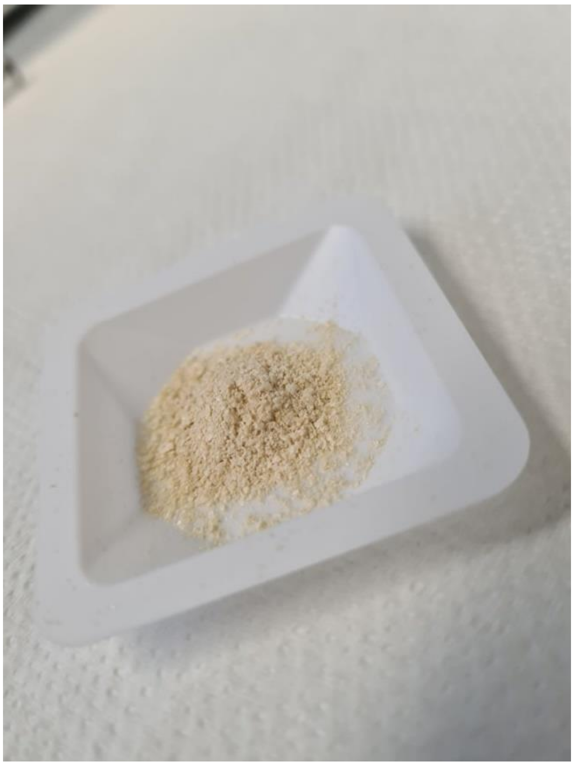

Supporting Photo S9. Pure product.

Method C: For reactions at room temperature.

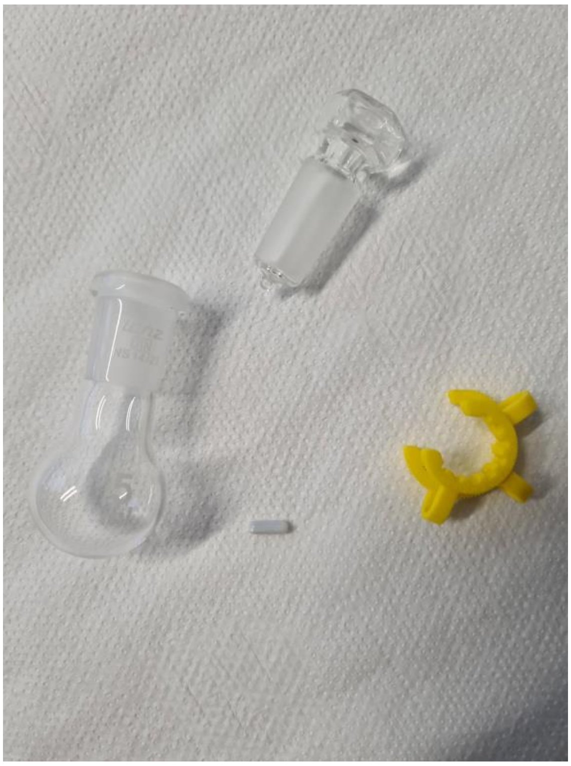

Supporting Photo S10. 5 mL round-bottomed flask, stirring bar, ground glass joint with plastic clamp.

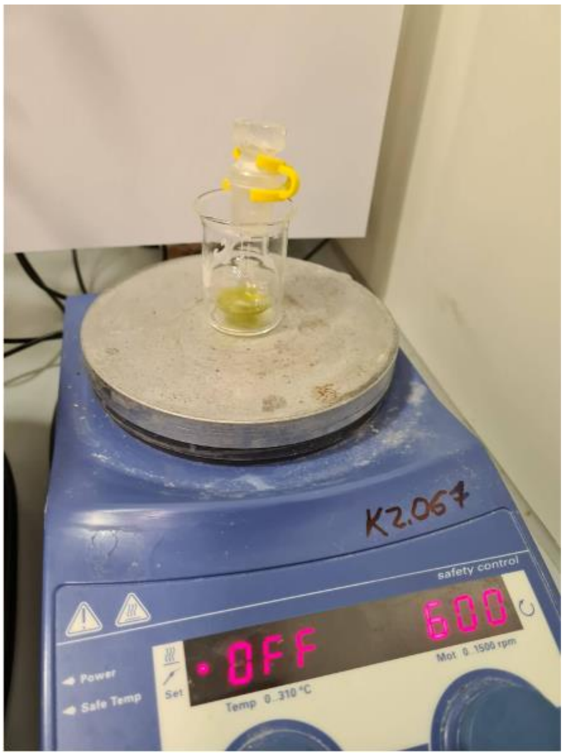

Supporting Photo S12. Stirring at 600 rpm at room temperature.

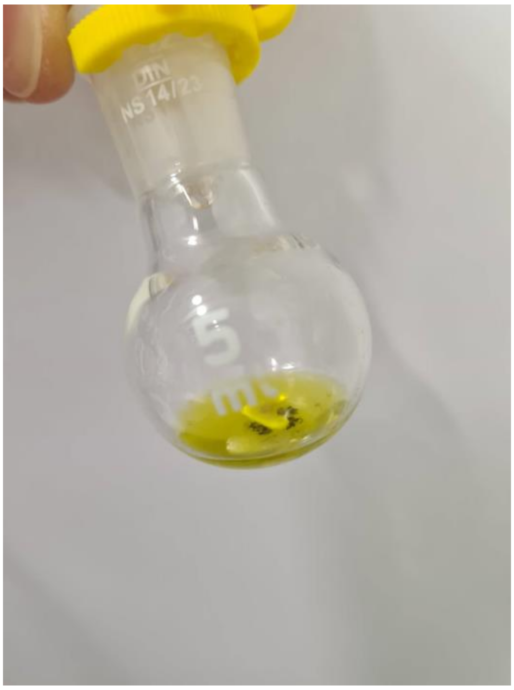

Supporting Photo S11. Reaction mixture (phenylacetylene, $CuSO_4$, $(BimH)_3$, sodium ascorbate, NaN_3, CH_3COOH in $MeOH/H_2O$) at t=0.

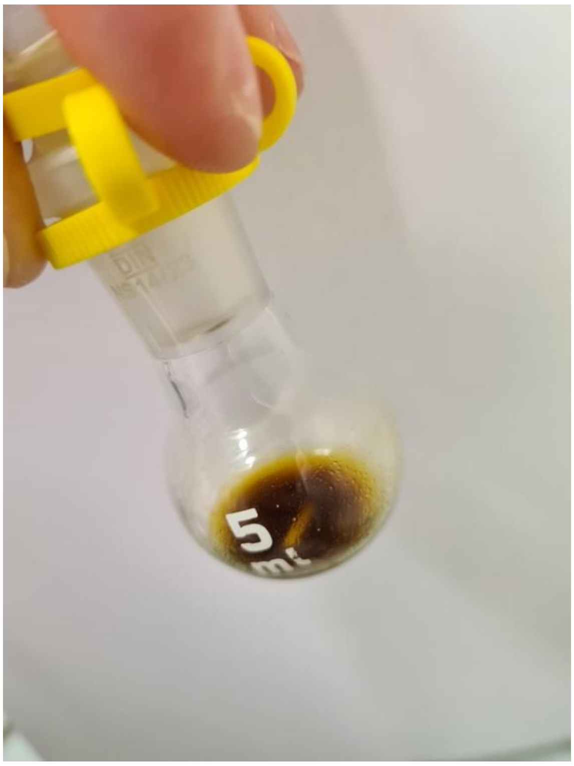

Supporting Photo S13. Reaction mixture (phenylacetylene, $(BimH)_3$, $CuSO_4$, sodium ascorbate, NaN_3, CH_3COOH in $MeOH/H_2O$) at t=24 h.

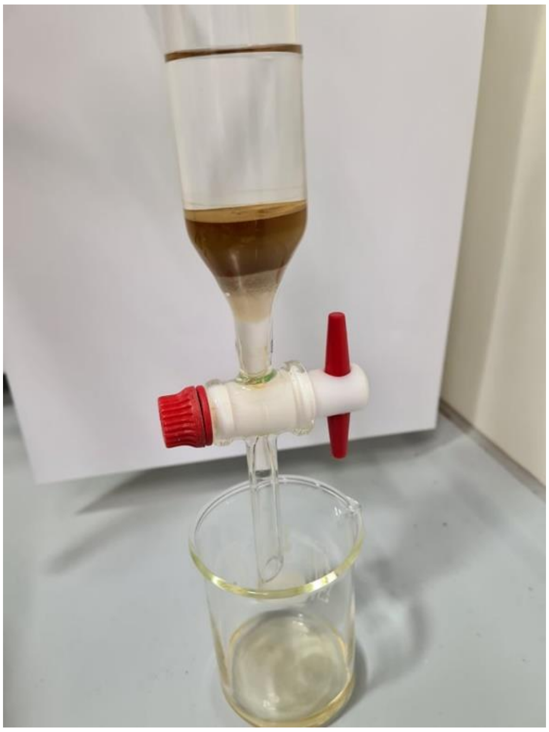

Supporting Photo S14. Filtration trough pad of silica using EtOAc.

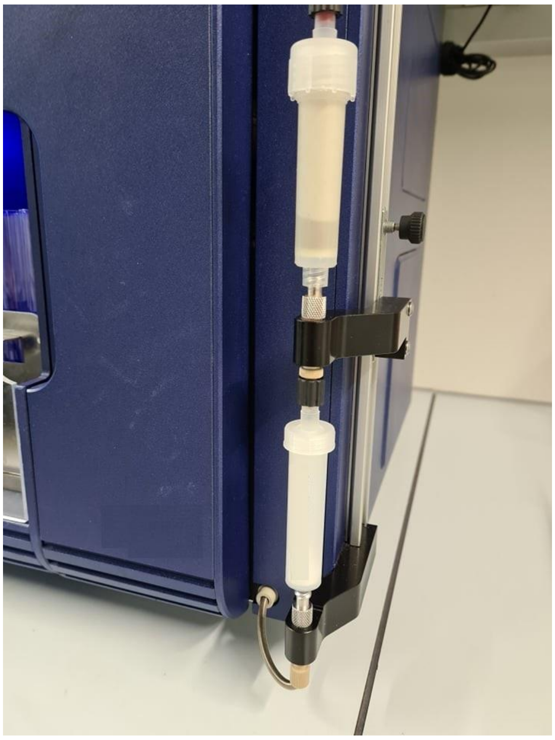

Supporting Photo S15. Purification with dryload silica column chromatography using Interchim puriFlash® XS520.

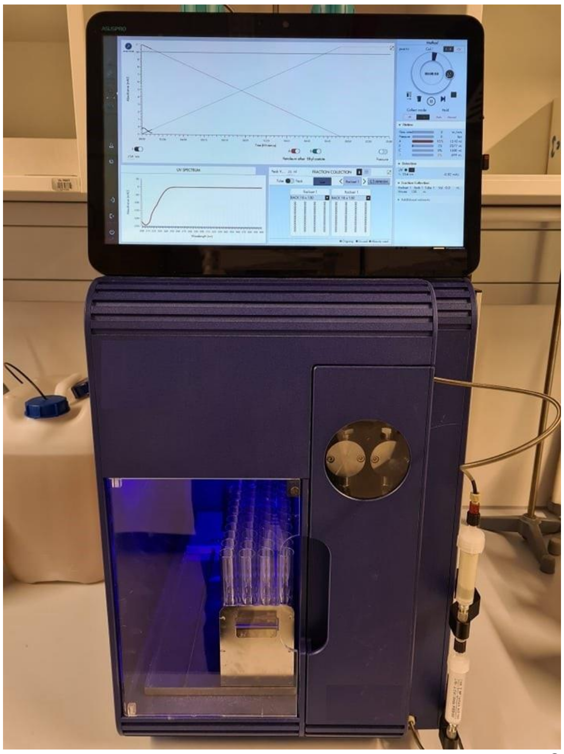

Supporting Photo S16. Interchim puriFlash® XS520.

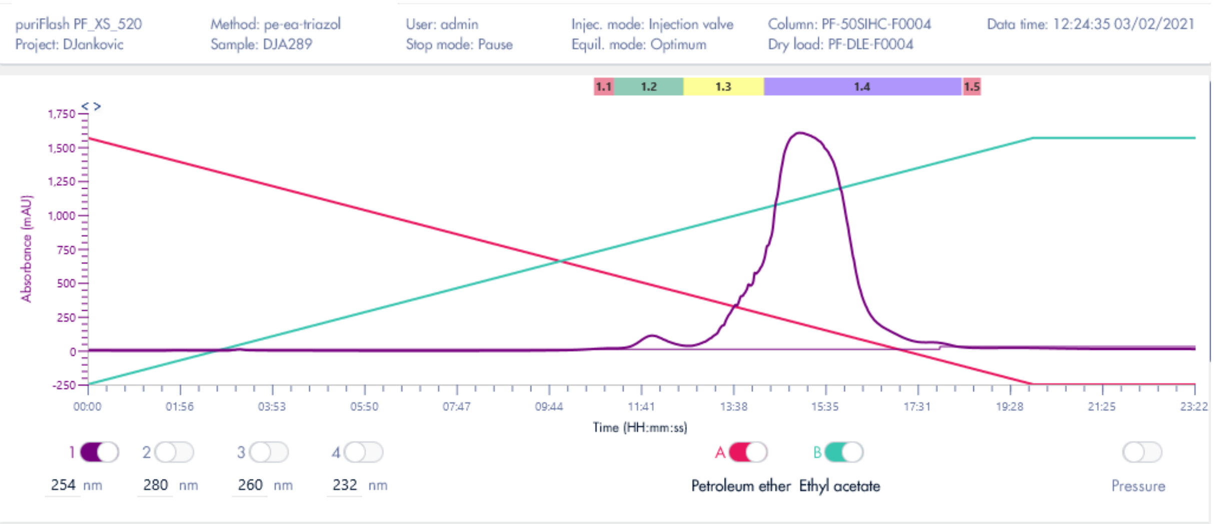

Supporting Photo S17. Purification report on Interchim puriFlash® XS520.

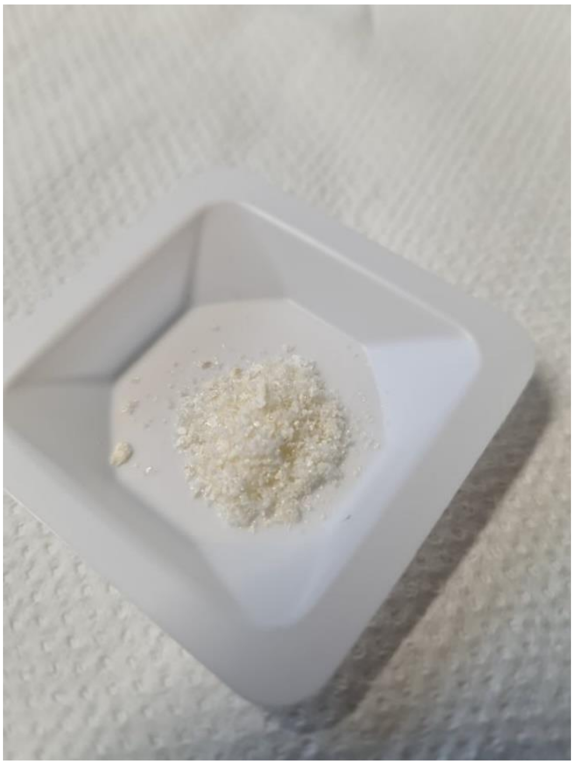

Supporting Photo S18. Pure product.

5. REFERENCES

(1) Graham, J. D. P.; Rogan, J. M.; Robertson, D. G.: Observations on hydrazoic acid. *J. Ind. Hyg. Toxicol.* **1948**, *30*, 98–102.

(2) Hammerl, A.; Klapötke, T. M. in *Encyclopedia of Inorganic Chemistry*. R. B. King (Ed.), Wiley, United States, 2nd Edition, **2005**, Nitrogen: Inorganic Chemistry, p. 25.

(3) Wiss, J.; Fleury, C.; Heuberger, C.; Onken, U.; Glor, M.: Explosion and Decomposition Characteristics of Hydrazoic Acid in the Gas Phase. *Org. Process Res. Dev.* **2007**, *11*, 1096–1103.

(4) McDonald, J. R.; Rabalais, J. W.; McGlynn, S. P.: Electronic Spectra of the Azide Ion, Hydrazoic Acid, and Azido Molecules. *J. Chem. Phys.* **1970**, *52*, 1332–1340.

(5) Woollars-Shore, J. G.; Holland, J. P.; Jones, M. W.; Dilworth, J. R.: Nitrite reduction by copper complexes. *Dalton Trans.* **2010**, *39*, 1576–1585.

(6) Vincze, I.; Lokos, M.; Bakos, T.; Dancsi, A.; Mak, M.: Selective Reduction and Hydrogenation of Unsaturated Steroids. *Steroids* **1993**, *58*, 220–224.

(7) Perez-Balado, C.; de Lera, A. R.: Concise total synthesis and structural revision of (+)-pestalazine B. *Org. Biomol. Chem.* **2010**, *8*, 5179–5186.

(8) Virant, M.; Košmrlj, J.: Arylation of Click Triazoles with Diaryliodonium Salts. *J. Org. Chem.* **2019**, *84*, 14030–14044.

(9) Prat, D.; Wells, A.; Hayler, J.; Sneddon, H.; McElroy, C. R.; Abou-Shehadad, S.; Dunn, P. J.: CHEM21 selection guide of classical- and less classical-solvents. *Green Chem.* **2016**, *18*, 288–296.

(10) Kalisak, J.; Sharpless, K. B.; Fokin, V. V.: Efficient Synthesis of 2-Substituted-1,2,3-triazoles. *Org. Lett.* **2008**, *10*, 3171–3174.

(11) Jiang, Y.; Kuang, C.; Yang, Q.: Copper(I) Iodide-Catalyzed Synthesis of 4-Aryl-1*H*-1,2,3-triazoles from *anti*-3-Aryl-2,3-dibromopropanoic Acids and Sodium Azide. *Synthesis* **2010**, 4256–4260.

(12) Lu, L-H.; Wu, J.-H.; Yang, C-H.: Preparation of 1H-1,2,3-Triazoles by Cuprous Ion Mediated Cycloaddition of Terminyl Alkyne and Sodium Azide. *J. Chin. Chem. Soc.* **2008**, *55*, 414–417.

(13) Finzi, P. V.: Reactions between sulfone azides and arylacetylenes. *Chim. e l'Indus.* **1965**, *47*, 1338–1339.

(14) Harvey, G. R.: The reactions of phosphorus compounds. XII. A new synthesis of 1,2,3-triazoles and diazo esters from phosphorus ylides and azides. *J. Org. Chem.* **1966**, *31*, 1587–1590.

(15) Ranga Prabhath, M. R.; Romanova, J.; Curry, R. J.; Ravi, S.; Silva, P.; Jarowski, P. D.: The Role of Substituent Effects in Tuning Metallophilic Interactions and Emission Energy of Bis-4-(2-pyridyl)-1,2,3-triazolatoplatinum(II) Complexes. *Angew. Chem. Int. Ed.* **2015**, *54*, 7949–7953.

(16) Jlalia, I.; Meganem, F.; Herscovici, J.; Girard, C.: "Flash" Solvent-free Synthesis of Triazoles Using a Supported Catalyst. *Molecules* **2009**, *14*, 528–539.

(17) Sprenger, K.; Golz, C.; Alcarazo, M.: Synthesis of Cycloheptatrienes, Oxepines, Thiepines, and Silepines: A Comparison between Brønsted Acid and Au-Catalysis. *Eur. J. Org. Chem.* **2020**, *39*, 6245–6254.

(18) Benhamou, L.; Walker, D. W.; Bučar, D.-K.; Aliev, A. E.; Sheppard, T. D.: Synthesis of substituted benzooxaborinin-1-ols *via* palladium-catalysed cyclisation of alkenyl- and alkynyl-boronic acids. *Org. Biomol. Chem.* **2016**, *14*, 8039–8043.

(19) Larsen, R. D.; King, A. O.; Chen, C. Y.; Corley, E. G.; Foster, B. S.; Roberts, F. E.; Yang, C.; Lieberman, D. R.; Reamer, R. A.; Tschaen, D. M.; Verhoven, T. R.; Reider, P. J.: Efficient Synthesis of Losartan, A Nonpeptide Angiotensin II Receptor Antagonist. *J. Org. Chem.* **1994**, *59*, 6391–6394.

(20) Hong, V.; Presolski, S. I.; Ma, C.; Finn, M. G.: Analysis and optimization of copper-catalyzed azide-alkyne cycloaddition for bioconjugation. *Angew. Chem., Int. Ed.* **2009**, *48*, 9879–9883.
